# Supplementary material for: Psychometric evaluation of the ‘Attitudes and Beliefs about Cardiovascular Disease (ABCD) Risk Questionnaire’ with validation of a previously untested ‘Intentions and Beliefs around Smoking’ subscale
Source: BMJ Open. 2023 Jan 13;13(1):e054532. doi: 10.1136/bmjopen-2021-054532 (PMC9843199; doi:10.1136/bmjopen-2021-054532)
Supplement: Supplementary data [file bmjopen-2021-054532supp003.pdf]

**Appendix 3**

ABCD subscale and selected INTERHEART variable correlation values from Nottingham study compared with values reported in the original Woringer study.

| | | Knowledge | Perceived Risk | Perceived Benefit | Healthy Intentions | IMD2010 Quintile | BMI/W2Hr | Qrisk2/INTERHEART |
|---|---|---|---|---|---|---|---|---|
| Knowledge | Correlation Coefficient | | -.124/ **.013** | -.148/ **-.021** | -.106/ **-.039** | -.002/ .085 | -.225/ -.084 | -.007/ -.018 |
| | Sig 2 tailed | | .236/ .722 | .175/ .645 | .319/ .400 | .986/ .066 | .021/ .082 | .941/ .714 |
| | N | | 93/462 | 86/462 | 91/462 | 99/466 | 105/433 | 104/436 |
| Perceived Risk | Correlation Coefficient | | | -.195/ -.112 | -.188/ -0.36 | .239/ .039 | .389/ .182 | .220/ .356 |
| | Sig 2 tailed | | | .080/ .016 | .088/ .441 | .025/ .397 | .000/ .000 | .036/ .000 |
| | N | | | 82/462 | 84/462 | 87/466 | 92/433 | 91/436 |
| Perceived Benefits | Correlation Coefficient | | | | .533/ .383 | -.287/ .071 | -.068/ .000 | -.118/ -.164 |
| | Sig 2 tailed | | | | .000/ .000 | .009/ .127 | .538/ .997 | .284/ .001 |
| | N | | | | 83/462 | 81/466 | 85/433 | 84/436 |
| Healthy Intentions | Correlation Coefficient | | | | | -.261/ .098 | .084/ .044 | -.072/ -.079 |
| | Sig 2 tailed | | | | | .016/ .034 | .430/ .365 | .504/ .100 |
| | N | | | | | 85/466 | 90/462 | 89/436 |

**Correlations**

**Correlations**

Correlations

| | | | knowledge score | Risk score | Benefit score | Diet score | Smoke score | total_score |
|---|---|---|---|---|---|---|---|---|
| Spearman's rho | knowledge score | Correlation Coefficient | 1.000 | .118** | .103* | .078 | -.079 | .006 |
| | | Sig. (2-tailed) | . | .009 | .023 | .086 | .082 | .896 |
| | | N | 483 | 483 | 483 | 483 | 483 | 440 |
| | Risk score | Correlation Coefficient | .118** | 1.000 | -.003 | .057 | .107* | .371** |
| | | Sig. (2-tailed) | .009 | . | .950 | .212 | .019 | .000 |
| | | N | 483 | 483 | 483 | 483 | 483 | 440 |
| | Benefit score | Correlation Coefficient | .103* | -.003 | 1.000 | .538** | .009 | -.236** |
| | | Sig. (2-tailed) | .023 | .950 | . | .000 | .851 | .000 |
| | | N | 483 | 483 | 483 | 483 | 483 | 440 |
| | Diet score | Correlation Coefficient | .078 | .057 | .538** | 1.000 | -.022 | -.143** |
| | | Sig. (2-tailed) | .086 | .212 | .000 | . | .635 | .003 |
| | | N | 483 | 483 | 483 | 483 | 483 | 440 |
| | Smoke score | Correlation Coefficient | -.079 | .107* | .009 | -.022 | 1.000 | .240** |
| | | Sig. (2-tailed) | .082 | .019 | .851 | .635 | . | .000 |
| | | N | 483 | 483 | 483 | 483 | 483 | 440 |
| | total_score | Correlation Coefficient | .006 | .371** | -.236** | -.143** | .240** | 1.000 |
| | | Sig. (2-tailed) | .896 | .000 | .000 | .003 | .000 | . |
| | | N | 440 | 440 | 440 | 440 | 440 | 440 |

** Correlation is significant at the 0.01 level (2-tailed).

* Correlation is significant at the 0.05 level (2-tailed).
