# Supplementary material for: Psychometric evaluation of the ‘Attitudes and Beliefs about Cardiovascular Disease (ABCD) Risk Questionnaire’ with validation of a previously untested ‘Intentions and Beliefs around Smoking’ subscale
Source: BMJ Open. 2023 Jan 13;13(1):e054532. doi: 10.1136/bmjopen-2021-054532 (PMC9843199; doi:10.1136/bmjopen-2021-054532)
Supplement: Supplementary data [file bmjopen-2021-054532supp004.pdf]

Appendix 4. Figures and factor result tables

Without smoking items

Non-missing samples: 420

Bartlett's Test of Sphericity (X2 = 4235.007, p-value < 0.001)

The overall KMO is 0.82, which is within the recommended range (0.8 to 1).

EFA results

- The root mean square of the residuals (RMSR) is 0.05
- Tucker Lewis Index of factoring reliability = 0.77
- RMSEA index = 0.121 and the 90 % confidence intervals are 0.113 0.129
- BIC = 165.35

Scree plot

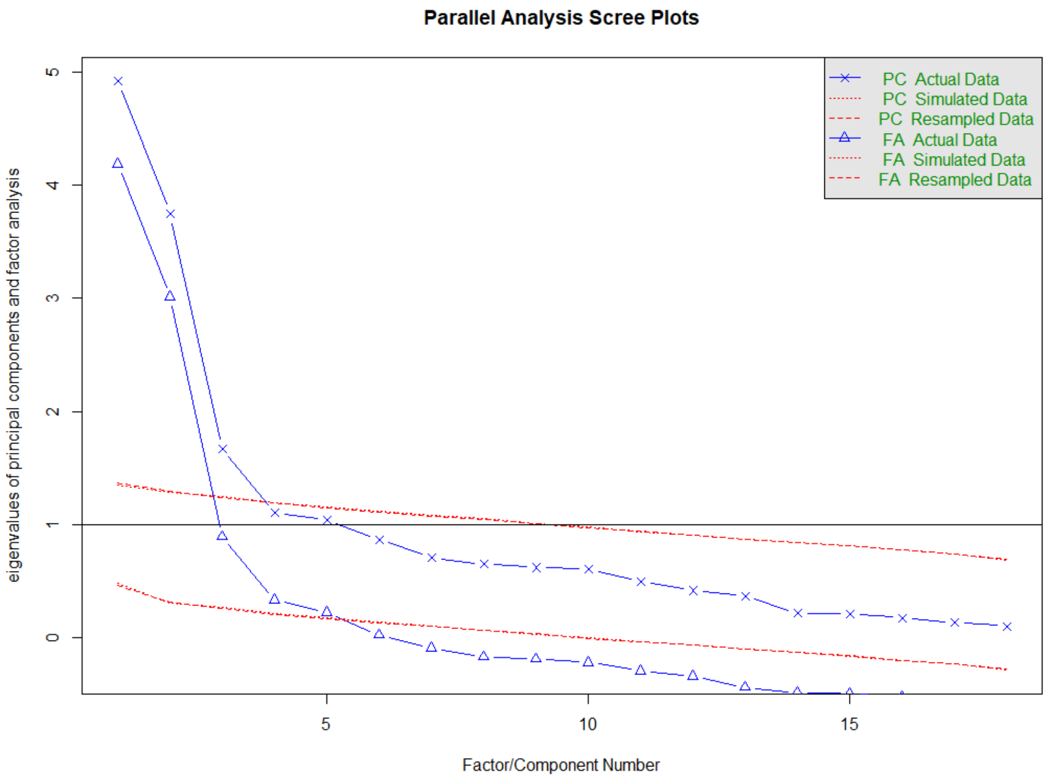

Figure 1. 18-item ABCD Questionnaire results (without smoking items)

Note: Scree plots are a line-plot of the eigenvalues of factors identified by the Principal Components Analysis (PCA) and Exploratory Factor Analysis (EFA). In this analysis, conducted using the independent Nottingham 'SPICES' study dataset, the blue lines indicate eigenvalues calculated for

each factor extracted from the observed data. Eigenvalues of 1 or greater are generally considered significant. The red lines represent eigenvalues generated by the PCA and EFA operations from a random data matrix of the same size as the original. Plotting both lines allow us to observe 1) the number of identified factors with eigenvalues exceeding 1, and 2) the point of inflection (the point at which the gap between resampled data and actual data tends to be minimum). The principle is to retain, at maximum, the number of factors with observed eigenvalues that are larger than those extracted from corresponding factors based on resampled/noise data.

Table A1 (a). Factor loadings of the exploratory factor analysis of the risk scale without the smoking items

**Items**	**Factor2**	**Factor1**	**Factor3**	**communality**	**uniqueness**
I feel I will suffer from a heart attack or stroke sometime during my life	0.86	0.02	-0.03	0.74	0.26
It is likely that I will suffer from a heart attack or stroke in the future	0.91	0.05	0.00	0.82	0.18
It is likely that I will have a heart attack or stroke sometime during my life	0.88	0.01	0.01	0.77	0.23
There is a good chance I will experience a heart attack or stroke in the next 10 years	0.73	-0.07	0.01	0.55	0.45
My chances of suffering from a heart attack or stroke in the next 10 years are great	0.65	-0.10	0.01	0.44	0.56
It is likely I will have a heart attack or stroke because of my past and/or present behaviors	0.56	-0.03	-0.01	0.32	0.68
I am not worried that I might have a heart attack or stroke (Reverse coded)	0.28	-0.11	0.10	0.10	0.90
I am concerned about the likelihood of having a heart attack or stroke in the near future	0.40	-0.02	0.11	0.16	0.84
I am thinking about exercising at least 2.5 hours a week	-0.02	0.87	-0.06	0.73	0.27
I intend or want to exercise at least 2.5 hours a week	-0.01	0.91	-0.04	0.80	0.20
When I exercise for at least 2.5 hours a week I am doing something good for the health of my heart	0.02	0.69	0.10	0.53	0.47
I am confident that I can maintain a healthy weight by exercising at least 2.5 hours a week	-0.05	0.45	0.19	0.31	0.69
I am not thinking about exercising for 2.5 hours a week (Reverse coded)	0.04	0.56	0.05	0.34	0.66
When I eat five portions of fruit and vegetables a day I am doing something good for the health of my heart	0.02	0.37	0.35	0.36	0.64
Increasing my exercise to at least 2.5 hours a week will decrease my chances of having a heart attack or stroke	0.02	0.39	0.27	0.30	0.70
I am confident that I can eat at least five portions of fruit and vegetables a day within the next two months	-0.04	0.07	0.64	0.46	0.54

I am thinking about eating at least five portions of fruit and vegetables a day	0.01	-0.01	0.93	0.85	0.15
I am not thinking about eating at least five portions of fruit and vegetables a day (Reverse coded)	-0.01	-0.03	0.78	0.60	0.40

Table A1 (b): Summary of factor loadings and variance distribution of the risk scale without the smoking items

Measures	Factor 2	Factor 1	Factor 3
SS loadings	3.86	3.04	2.28
Proportion Var	0.21	0.17	0.13
Cumulative Var	0.21	0.38	0.51
Proportion Explained	0.42	0.33	0.25
Cumulative Proportion	0.42	0.75	1.00

With smoking items

Non-missing samples: 88

The overall KMO is 0.78, which is slightly below the recommended range (0.8 to 1).

The Bartlet's test of Sphericity is significant (X2 = 1223.459, p-value < 0.001), indicating the sample adequacy for factor analysis.

EFA results

- The root mean square of the residuals (RMSR) is 0.06
- Tucker Lewis Index of factoring reliability = 0.69
- RMSEA index = 0.129 and the 90 % confidence intervals are 0.124 and 0.136
- BIC = 440.9

Scree plot

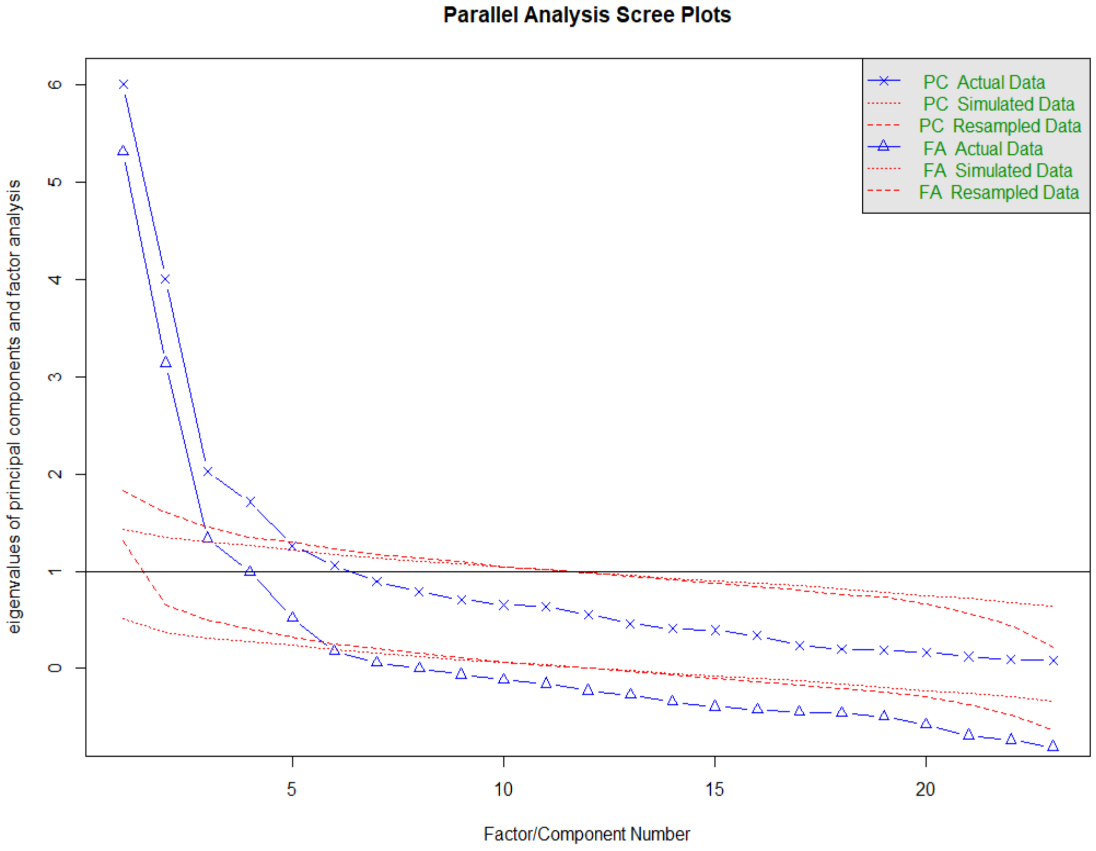

Figure 2. Modified ABCD Questionnaire 23 items with smoking.

Table A2 (a). Factor loadings of the exploratory factor analysis of the risk scale with the smoking items

Items	Factor2	Factor3	Factor1	Factor4	Communality	Uniqueness
I feel I will suffer from a heart attack or stroke sometime during my life	0.86	-0.1	0.05	-0.02	0.76	0.24
It is likely that I will suffer from a heart attack or stroke in the future	0.91	0.06	0.02	-0.01	0.82	0.18
It is likely that I will have a heart attack or stroke sometime during my life	0.88	0.02	0	0	0.77	0.23
There is a good chance I will experience a heart attack or stroke in the next 10 years	0.72	0	-0.09	0.01	0.54	0.46
My chances of suffering from a heart attack or stroke in the next 10 years are great	0.64	-0.03	-0.1	0.01	0.45	0.55
It is likely I will have a heart attack or stroke because of my past and/or present behaviors	0.57	-0.07	0	0	0.33	0.67

I am not worried that I might have a heart attack or stroke (Reverse coded)	0.28	0.02	-0.14	0.1	0.1	0.9
I am concerned about the likelihood of having a heart attack or stroke in the near future	0.41	0.19	-0.12	0.08	0.19	0.81
I am thinking about exercising at least 2.5 hours a week	-0.03	-0.05	0.88	-0.02	0.73	0.27
I intend or want to exercise at least 2.5 hours a week	-0.02	0.05	0.87	-0.02	0.79	0.21
When I exercise for at least 2.5 hours a week I am doing something good for the health of my heart	0.03	0.17	0.62	0.09	0.55	0.45
I am confident that I can maintain a healthy weight by exercising at least 2.5 hours a week	-0.05	0.09	0.42	0.18	0.32	0.68
I am not thinking about exercising for 2.5 hours a week (Reverse coded)	0.02	0	0.53	0.09	0.33	0.67
When I eat five portions of fruit and vegetables a day I am doing something good for the health of my heart	0.04	0.07	0.35	0.35	0.36	0.64
Increasing my exercise to at least 2.5 hours a week will decrease my chances of having a heart attack or stroke	0.04	0.12	0.37	0.24	0.32	0.68
I am confident that I can eat at least five portions of fruit and vegetables a day within the next two months	-0.04	-0.05	0.12	0.64	0.45	0.55
I am thinking about eating at least five portions of fruit and vegetables a day	0.01	0	0.02	0.89	0.8	0.2
I am not thinking about eating at least five portions of fruit and vegetables a day (Reverse coded)	-0.01	0	-0.06	0.83	0.66	0.34
I am thinking of stopping smoking within two months	0.06	0.78	0.12	-0.06	0.67	0.33
I have reduced or stopped smoking	-0.03	0.83	0.02	-0.01	0.71	0.29
I intend or want to stop smoking	-0.05	0.9	-0.02	-0.01	0.8	0.2
If I stop smoking it will reduce my chances of having a heart attack or stroke	0.16	0.58	0.09	0.08	0.43	0.57
I am not thinking about stopping smoking	-0.12	0.56	-0.2	0.17	0.35	0.65

Table A2 (b): Summary of factor loadings and variance distribution of the risk scale with the smoking items

**Measures**	**Factor 2**	**Factor 3**	**Factor 1**	**Factor 4**
SS loadings	3.90	3.00	2.97	2.33
Proportion Var	0.17	0.13	0.13	0.10
Cumulative Var	0.17	0.30	0.43	0.53
Proportion Explained	0.32	0.25	0.24	0.19
Cumulative Proportion	0.32	0.57	0.81	1.00

Modified scale (20-items including the smoking items)

Non-missing samples: 89

The overall KMO is 0.79, which is slightly below the recommended range (0.8 to 1).

The Bartlet's test of Sphericity is significant (X2 = 915.41, p-value < 0.001), indicating the sample adequacy for factor analysis.

EFA results

- The root mean square of the residuals (RMSR) is 0.06
- Tucker Lewis Index of factoring reliability = 0.72
- RMSEA index = 0.118 and the 90 % confidence intervals are 0.111 and 0.126
- BIC = 153.72

Scree plot

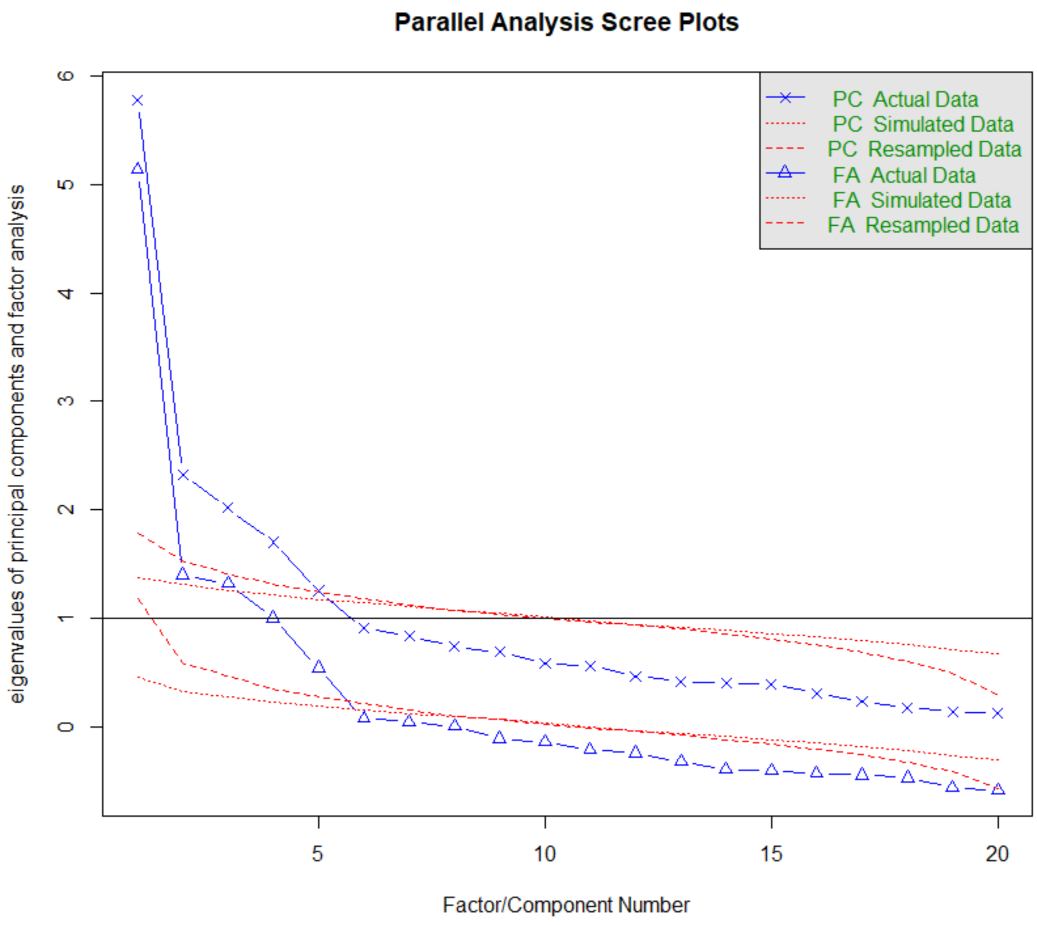

Figure 3. Modified ABCD Questionnaire 20 items with smoking.

Table A3 (a). Factor loadings of the exploratory factor analysis of the modified risk scale (20 items including the smoking items)

Items	Factor3	Factor1	Factor4	Factor2	Communality	Uniqueness
I feel I will suffer from a heart attack or stroke sometime during my life	-0.08	0.04	-0.03	0.76	0.60	0.40
There is a good chance I will experience a heart attack or stroke in the next 10 years	0.02	-0.08	-0.01	0.68	0.48	0.52
It is likely I will have a heart attack or stroke because of my past and/or present behaviors	-0.04	0.01	-0.01	0.61	0.38	0.62
I am not worried that I might have a heart attack or stroke (Reverse coded)	0.04	-0.13	0.10	0.35	0.14	0.86
I am concerned about the likelihood of having a heart attack or stroke in the near future	0.22	-0.11	0.07	0.45	0.23	0.77
I am thinking about exercising at least 2.5 hours a week	-0.06	0.88	-0.02	-0.04	0.74	0.26
I intend or want to exercise at least 2.5 hours a week	0.05	0.87	-0.02	-0.02	0.79	0.21
When I exercise for at least 2.5 hours a week I am doing something good for the health of my heart	0.17	0.62	0.09	0.04	0.55	0.45
I am confident that I can maintain a healthy weight by exercising at least 2.5 hours a week	0.09	0.42	0.18	-0.06	0.32	0.68
I am not thinking about exercising for 2.5 hours a week (Reverse coded)	0.01	0.53	0.09	0.03	0.32	0.68
When I eat five portions of fruit and vegetables a day I am doing something good for the health of my heart	0.08	0.35	0.35	0.07	0.37	0.63
Increasing my exercise to at least 2.5 hours a week will decrease my chances of having a heart attack or stroke	0.13	0.37	0.24	0.06	0.32	0.68

I am confident that I can eat at least five portions of fruit and vegetables a day within the next two months	-0.06	0.12	0.64	-0.05	0.46	0.54
I am thinking about eating at least five portions of fruit and vegetables a day	0.00	0.02	0.89	0.01	0.80	0.20
I am not thinking about eating at least five portions of fruit and vegetables a day (Reverse coded)	0.00	-0.06	0.83	-0.01	0.67	0.33
I am thinking of stopping smoking within two months	0.78	0.12	-0.06	0.04	0.66	0.34
I have reduced or stopped smoking	0.83	0.02	-0.01	-0.03	0.70	0.30
I intend or want to stop smoking	0.89	-0.02	-0.01	-0.07	0.80	0.20
If I stop smoking it will reduce my chances of having a heart attack or stroke	0.59	0.10	0.07	0.18	0.43	0.57
I am not thinking about stopping smoking	0.56	-0.20	0.17	-0.10	0.34	0.66

Table A3 (b): Summary of factor loadings and variance distribution of the modified risk scale (20 items including the smoking items)

**Measures**	**Factor3**	**Factor1**	**Factor4**	**Factor2**
SS loadings	3.00	2.96	2.33	1.80
Proportion Var	0.15	0.15	0.12	0.09
Cumulative Var	0.15	0.30	0.41	0.50
Proportion Explained	0.30	0.29	0.23	0.18
Cumulative Proportion	0.30	0.59	0.82	1.00
