# Supplementary material for: Psychometric evaluation of the ‘Attitudes and Beliefs about Cardiovascular Disease (ABCD) Risk Questionnaire’ with validation of a previously untested ‘Intentions and Beliefs around Smoking’ subscale
Source: BMJ Open. 2023 Jan 13;13(1):e054532. doi: 10.1136/bmjopen-2021-054532 (PMC9843199; doi:10.1136/bmjopen-2021-054532)
Supplement: Supplementary data [file bmjopen-2021-054532supp005.pdf]

Appendix 5.

Item Analysis of published ABCD Risk Questionnaire sub-scales plus 5 unpublished items relating to smoking compared to Item Analysis of recommended edited ABCD Risk Questionnaire sub-scales plus 5 unpublished items relating to smoking.

Table 1. Item Analysis of published ABCD Risk Questionnaire sub-scales plus 5 unpublished items relating to smoking

**Perceived Risk of Heart Attack/ Stroke<br>8 Items<br>Cronbach's Alpha .861<br>(0.84,0.88) 95% CI**	**Inter-item correlation**	**Corrected Item-total correlation**	**Cronbach's alpha if item deleted**
It is likely that I will suffer from a heart attack or stroke in the future	**.832**	.756	.826
It is likely that I will have a heart attack or stroke some time during my life	**.869**	.777	.824
I feel I will suffer a heart attack or stroke some time during my life	**.616**	.784	.824
There is a good chance I will experience a heart attack or stroke in the next 10 years	**.729**	.722	.832
I am not worried that I might have a heart attack or stroke	.403	.624	.843
My chances of suffering a heart attack or stroke in the next 10 years are great	.245	.544	.852
It is likely that I will have a heart attack or stroke because of my past/present behaviours	.266	.319	**.876**
I am concerned about the likelihood of having a heart attack or stroke in the near future	.259	.387	**.870**
**Perceived Benefits and Intentions to Change<br>7 items<br>Cronbach's Alpha .801**	**Inter-item correlation**	**Corrected Item-total correlation**	**Cronbach's alpha if item deleted**
I am thinking about exercising at least 2.5 hours a week	**.727**	**.605**	.760
I intend or want to exercise at least 2.5 hours a week	.442	**.651**	.752
When I exercise for at least 2.5 hours a week I am doing something good for the health of my heart	.426	.593	.769
I am confident that I can maintain a healthy weight by exercising at	.294	.452	.790

least 2.5 hours a week within the next 2 months			
I am not thinking about exercising at least 2.5 hours a week	.264	.508	.781
When I eat at least 5 portions of fruit and vegetables a day I am doing something good for the health of my heart	.483	.483	.783
Increasing my exercise to at least 2.5 hours a week will decrease my chances of having a heart attack or stroke	.326	.474	.786
**Healthy Eating Intentions 3 items Cronbach's Alpha .787 (95% CI**	**Inter-item correlation**	**Corrected Item-total correlation**	**Cronbach's alpha if item deleted**
I am confident that I can eat at least 5 portions of fruit and vegetables a day within the next 2 months	.555	.533	**.812**
I am thinking about eating at least 5 portions of fruit and vegetables a day	**.683**	**.732**	.596
I am not thinking about eating at least 5 portions of fruit and vegetables a day	.424	**.624**	.713
**Perceived Benefits and Intentions to Stop Smoking 5 Items Cronbach's Alpha .943 95% CI**	**Inter-item correlation**	**Corrected item-total correlation**	**Cronbach's alpha if item deleted**
I am thinking of stopping smoking within the next 2 months	**.654**	.848	.932
I have reduced or stopped smoking	**.694**	.751	**.949**
I intend or want to stop smoking	**.829**	.906	.919
If I stop smoking it will reduce my chances of having a heart attack or stroke	**.834**	.886	.922
I am not thinking about stopping smoking	**.789**	.872	.925

Table 2. Item Analysis of edited ABCD Risk Questionnaire sub-scales plus 5 unpublished items relating to smoking.

**Perceived Risk of Heart Attack/ Stroke<br>5 Items<br>Cronbach's Alpha .86 (0.84,0.88) 95% CI<br>Omega 0.85 (0.83, 0.88) 95% CI**	**Inter-item correlation**	**Corrected Item-total correlation**	**Cronbach's alpha if item deleted**
It is likely that I will have a heart attack or stroke some time during my life	**.869**	.777	.824
There is a good chance I will experience a heart attack or stroke in the next 10 years	**.729**	.722	.832
I am not worried that I might have a heart attack or stroke	.403	.624	.843
It is likely that I will have a heart attack or stroke because of my past/present behaviours	.266	.319	**.876**
I am concerned about the likelihood of having a heart attack or stroke in the near future	.259	.387	**.870**
**Perceived Benefits and Intentions to Change<br>6 items<br>Cronbach's Alpha .84 (.81-.86) 95% CI<br>Omega 0.82 (0.78, 0.85) 95% CI**	**Inter-item correlation**	**Corrected Item-total correlation**	**Cronbach's alpha if item deleted**
I am thinking about exercising at least 2.5 hours a week	**.727**	**.605**	.760
I intend or want to exercise at least 2.5 hours a week	.442	**.651**	.752
When I exercise for at least 2.5 hours a week I am doing something good for the health of my heart	.426	.593	.769
I am confident that I can maintain a healthy weight by exercising at least 2.5 hours a week within the next 2 months	.294	.452	.790
I am not thinking about exercising at least 2.5 hours a week	.264	.508	.781
Increasing my exercise to at least 2.5 hours a week will decrease my chances of having a heart attack or stroke	.326	.474	.786
**Healthy Eating Intentions<br>4 items**	**Inter-item correlation**	**Corrected Item-total correlation**	**Cronbach's alpha if item deleted**

**Cronbach's Alpha .84 (.81-.86) 95% CI<br>Omega 0.84 (0.81, 0.88) 95% CI**			
I am confident that I can eat at least 5 portions of fruit and vegetables a day within the next 2 months	.555	.533	**.812**
I am thinking about eating at least 5 portions of fruit and vegetables a day	**.683**	**.732**	.596
I am not thinking about eating at least 5 portions of fruit and vegetables a day	.424	**.624**	.713
When I eat at least 5 portions of fruit and vegetables a day I am doing something good for the health of my heart	.483	.483	.783
**Smoking Intentions<br>5 items<br>Cronbach's Alpha .85 (.83-.87) 95% CI<br>Omega 0.84 (0.81, 0.91) 95% CI**	**Inter-item correlation**	**Corrected Item-total correlation**	**Cronbach's alpha if item deleted**
I am thinking of stopping smoking within the next 2 months	**.654**	.848	.932
I have reduced or stopped smoking	**.694**	.751	**.949**
I intend or want to stop smoking	**.829**	.906	.919
If I stop smoking it will reduce my chances of having a heart attack or stroke	**.834**	.886	.922
I am not thinking about stopping smoking	**.789**	.872	.925
