# Supplementary material for: Psychometric evaluation of the ‘Attitudes and Beliefs about Cardiovascular Disease (ABCD) Risk Questionnaire’ with validation of a previously untested ‘Intentions and Beliefs around Smoking’ subscale
Source: BMJ Open. 2023 Jan 13;13(1):e054532. doi: 10.1136/bmjopen-2021-054532 (PMC9843199; doi:10.1136/bmjopen-2021-054532)
Supplement: Supplementary data [file bmjopen-2021-054532supp006.pdf]

Appendix 6. Characteristics of the sample population

Population Characteristics		N	% total
**Gender**	Male	218	49.8
	Female	220	50.2
**Age Group**	18-30	78	17.8
	30-39	80	18.3
	40-49	82	18.7
	50-59	99	22.6
	60-74	78	17.8
	74+	53	12.1
**Deprivation**	IMD1- least deprived	84	17.98
	IMD2	55	11.77
	IMD3	83	17.77
	IMD4	89	19.05
	IMD5- most deprived	156	33.4
