# Supplementary material for: Psychometric evaluation of the ‘Attitudes and Beliefs about Cardiovascular Disease (ABCD) Risk Questionnaire’ with validation of a previously untested ‘Intentions and Beliefs around Smoking’ subscale
Source: BMJ Open. 2023 Jan 13;13(1):e054532. doi: 10.1136/bmjopen-2021-054532 (PMC9843199; doi:10.1136/bmjopen-2021-054532)
Supplement: Supplementary data [file bmjopen-2021-054532supp007.pdf]

Appendix 7. Modified ABCD Risk Questionnaire

Mark Bowyer, Hamid Hassen

Scale	Items	Coding
**Perceived Risk of Heart Attack or Stroke**	1. It is likely that I will have a heart attack or stroke sometime in my life	4= Strongly disagree, 3= Disagree, 2= Agree, 1= Strongly Agree; N/A= 0
	2. There is a good chance I will experience a heart attack or stroke in the next 10 years	4= Strongly disagree, 3= Disagree, 2= Agree, 1= Strongly Agree; N/A= 0
	3. It is (more) likely I will have a heart attack or stroke because of my past and/or present behaviours	4= Strongly disagree, 3= Disagree, 2= Agree, 1= Strongly Agree; N/A= 0
	4. I am not worried that I might have a heart attack or stroke	**REVERSE CODED** 4= Strongly disagree, 3= Disagree, 2= Agree, 1= Strongly Agree; N/A= 0
	5. I am concerned about the likelihood of having a heart attack or stroke in the near future	4= Strongly disagree, 3= Disagree, 2= Agree, 1= Strongly Agree; N/A= 0
**Perceived Benefits and Intentions to Exercise**	6. I am thinking about exercising at least 2.5 hours a week	4= Strongly disagree, 3= Disagree, 2= Agree, 1= Strongly Agree; N/A= 0
	7. I intend or want to exercise at least 2.5 hours a week	4= Strongly disagree, 3= Disagree, 2= Agree, 1= Strongly Agree; N/A= 0
	8. When I exercise for at least 2.5 hours a week I am doing something good for the health of my heart	4= Strongly disagree, 3= Disagree, 2= Agree, 1= Strongly Agree; N/A= 0
	9. I am confident that I can maintain a healthy weight by exercising at least 2.5 hours a week	4= Strongly disagree, 3= Disagree, 2= Agree, 1= Strongly Agree; N/A= 0
	10. I am not thinking about exercising for 2.5 hours a week	**REVERSE CODED** 4= Strongly disagree, 3= Disagree, 2= Agree, 1= Strongly Agree; N/A= 0
	11. Increasing my exercise to at least 2.5 hours a week will decrease my chances of having a heart attack or stroke	4= Strongly disagree, 3= Disagree, 2= Agree, 1= Strongly Agree; N/A= 0

**Perceived Benefit and Healthy Eating Intentions**	12. I am confident that I can eat at least five portions of fruit and vegetables a day within the next two months	4= Strongly disagree, 3= Disagree, 2= Agree, 1= Strongly Agree; N/A= 0
	13. I am thinking about eating at least five portions of fruit and vegetables a day	4= Strongly disagree, 3= Disagree, 2= Agree, 1= Strongly Agree; N/A= 0
	14. I am not thinking about eating at least five portions of fruit and vegetables a day	**REVERSE CODED** 4= Strongly disagree, 3= Disagree, 2= Agree, 1= Strongly Agree; N/A= 0
	15. When I eat five portions of fruit and vegetables a day I am doing something good for the health of my heart	4= Strongly disagree, 3= Disagree, 2= Agree, 1= Strongly Agree; N/A= 0
**Benefits and Intentions to Stop Smoking**	16. I am thinking of stopping smoking within two months	4= Strongly disagree, 3= Disagree, 2= Agree, 1= Strongly Agree; N/A= 0
	17. I have reduced or stopped smoking	4= Strongly disagree, 3= Disagree, 2= Agree, 1= Strongly Agree; N/A= 0
	18. I intend or want to stop smoking	4= Strongly disagree, 3= Disagree, 2= Agree, 1= Strongly Agree; N/A= 0
	19. If I stop smoking it will reduce my chances of having a heart attack or stroke	4= Strongly disagree, 3= Disagree, 2= Agree, 1= Strongly Agree; N/A= 0
	20. I am not thinking about stopping smoking	**REVERSE CODED** 4= Strongly disagree, 3= Disagree, 2= Agree, 1= Strongly Agree; N/A= 0
