# Supplementary material for: Psychometric evaluation of the ‘Attitudes and Beliefs about Cardiovascular Disease (ABCD) Risk Questionnaire’ with validation of a previously untested ‘Intentions and Beliefs around Smoking’ subscale
Source: BMJ Open. 2023 Jan 13;13(1):e054532. doi: 10.1136/bmjopen-2021-054532 (PMC9843199; doi:10.1136/bmjopen-2021-054532)
Supplement: Supplementary data [file bmjopen-2021-054532supp002.pdf]

A Protocol Paper: Community engagement interventions for Cardiovascular Disorders prevention in socially disadvantaged populations in the UK: An implementation research study
Final 15072019

Target Journal: Journal of Global Health Research and Policy
https://ghrp.biomedcentral.com/?gclid=Cj0KCQiA68bhBRCKARIsABYUGifuKd-xktjcmV7tn3r7G-IEqS5rAb6QmiEl6P9dXGBdNRDhsIPVzA0aAiJWEALw_wcB

Papreen Nahar[1], Harm van Marwijk[1], Linda Gibson[2], Geofrey Musinguzi[3], Sibyl Anthierens[4], Elizabeth Ford [1], Stephen A Bremner [1], Mark Bower [2], Jean Yves Le Reste[5], Tholene Sodi [6], Hilde Bastiaens[4]

Corresponding author: Dr Papreen Nahar, Department of Primary Care and Public Health, Brighton and Sussex Medical School, UK. The University of Sussex. E-mail: P.Nahar@bsms.ac.uk

Abstract:

Cardiovascular disorders (CVD) are the single greatest cause of mortality worldwide. In the UK, the National Health Service (NHS) has launched an initiative of health checks over and above current care to tackle CVD. However, the uptake of Health Checks is poor in disadvantaged communities. This protocol paper sets out a UK-based study aiming to co-produce a community delivered CVD risk assessment and coaching intervention to support community members to reduce their risk of CVD.

The overall aim of the project is to implement a tailored-to-context community engagement (CE) intervention on awareness of CVD risks in vulnerable populations in high, middle and low-income countries. This paper describes the protocol for the UK sites in Sussex and Nottingham. The specific objectives of the study are to enhance stakeholder' engagement; to implement lifestyle interventions for cardiovascular primary prevention, in disadvantaged populations and motivate uptake of NHS health checks.

This study takes a mixed methods approach, combining qualitative and quantitative methods in three phases of evaluation, including pre-, during- and post-implementation. To ensure contextual appropriateness the SPICES project will organize a multi-component community-engagement intervention implementation. For the qualitative component, the pre-implementation phase will involve a contextual assessment and stakeholder mapping, exploring potentials for CVD risk profiling strategies and led by trained Community Health Volunteers (CHV) to identify accessibility and acceptability. The during-implementation phase will involve healthy lifestyle counselling provided by CHVs and evaluation of the outcome to identify fidelity and scalability. The post-implementation phase will involve developing sustainable community-based strategies for CVD risk reduction. All three components will include a process evaluation. The theory of the socio-ecological framework will be applied to analyse the community engagement approach.

A stepped wedge quantitative evaluation of the roll out will focus on implementation outcomes such as uptake and engagement and changes in risk profiles. The quantitative component includes pre and post-intervention surveys.

The research project will ultimately develop a sustainable community engagement-based strategy for the primary prevention of CVD, to support or enhance the performance of NHS health care.

Key words: Implementation research, Cardiovascular disorders prevention, community engagement.

Introduction:

Cardiovascular disorders (CVD) are the single greatest cause of mortality worldwide each year, estimated to contribute to 31% of all deaths globally (1). Tackling CVD is an international priority and there have been many global initiatives such as the “Global Hearts” programme, a package launched by the World Health Organisation (WHO) and partners, to enhance the prevention and control of CVD. Some risk factors for CVD are non-modifiable, such as age, ethnicity and family history (2). Some other risk factors for CVD are modifiable, such as smoking, a lack of physical activity, being overweight, lower consumption of fruit and vegetables, high blood pressure, diabetes and high cholesterol (2). These risk factors can be changed through lifestyle or behavioural modifications. There is evidence of a social gradient in the prevalence of CVD, which points to associations between social and financial deprivation, vulnerability and risk factors for CVD. (3).

In 2015, CVD was the leading cause of mortality in the context of all chronic diseases, accounting for 27% and 25% of deaths in men and women respectively, in the UK(2). Coronary heart disease (CHD) and stroke were the main CVDs responsible for this mortality of men and women across all ages. As per British Heart Foundation report in 2017 CVD has a huge financial burden with annual associated healthcare costs estimated to be £9 billion annually in the UK (2). The UK has a standardised CVD death rate of 265.1 per 100,000 (2).

In the UK, the National Health Service (NHS) has launched the Health Check initiative aimed to prevent CVD. It is a national risk assessment and management program, free to adults aged 40 to 74 living in England, who do not currently have any vascular disorders and are not being treated for certain risk factors such as diabetes (4). It aims to assess the 10-year risk of CV events and disorders. Risk is assessed using QRISK2 (5), a tool which involves collection of the following information: age, gender, ethnicity, smoking status, family history of CHD, body mass index (BMI), cholesterol test, systolic and diastolic blood pressure, levels of physical activity, and alcohol consumption. Attendees receive a low (<10 % chance of event in 10 years), medium (>10 % but <20 %), or high (>20 %) 10-year cardiovascular (QRISK2) score. Above the 10% cut-off, attendees are offered a discussion with a qualified person, such as a nurse, about lifestyle and motivation to change, which may include goal setting and plans for follow up. Patients may also be offered medication for cholesterol and blood pressure. The NHS Health Check is recommended to be undertaken every five years.

Modelling predicted that the NHS Health Check could prevent 1,600 heart attacks and strokes each year if implemented as intended (6). Whilst evidence suggests that the Health Check programme has the potential to reduce CVD events and has therefore been rolled out nationally across the UK, its implementation has been poor, especially in some of the most disadvantaged groups at highest risk of developing CVD. In 2014, Public Health England (PHE) issued a call for action to increase the uptake rate of NHS Health Checks to 75% (7) and to increase awareness of risk and engagement with existing resources. Yet, as of 2017, current uptake remains far from this target with current predictions suggesting only 40% of the eligible population will receive one (8), due to the fact that uptake is low (48%) even when Health Checks are offered. (8) (9)

Data from some regions with very large ethnic minority community and socio-economically challenged populations showed that only 45% of patients who were invited for the check attended and subsequently received some form of counselling when they needed it. Authors have discussed how higher uptake in deprived communities would reduce the possibility of exacerbation of inequalities (10). Difficulty with accessing general practices, especially among socially vulnerable groups, has been highlighted as a common barrier to

attendance at Health Checks (11). A community-based engagement approach, which takes the CVD risking profiling and affiliated advice processes outside of the formal healthcare facility setting, has the potential to improve access to Health Checks and could be an effective and scalable way for improving the implementation and uptake of Health Checks. Community engagement (CE) has been conceptualised as "the process of working collaboratively with and through groups of people affiliated by geographic proximity, special interest, or similar situations, to address issues affecting the well-being of those people" (12). A review of community engagement interventions found them to be effective in improving health behaviours (such as physical activity), health consequences and psychological outcomes (i.e. self-efficacy and perceived social support) (13). Community-based intervention programmes have been implemented to increase the uptake of cancer screening programmes. The programmes have been found to be effective in increasing outcomes such as recognition, receipt and maintenance of screening behaviours (14). The CE approach offers the opportunity for task-shifting and owning the programme, whereby trained non-healthcare-professionals can perform CVD risk profiling assessments to individuals who might not otherwise be captured by the formal care pathway.

There is evidence that CVD risk assessments can be successfully delivered by Community Health Workers (CHWs), outside or inside the healthcare system. An observational study conducted in Bangladesh, Guatemala, Mexico and South Africa has demonstrated that CHWs who are inhabitants of their local communities and were fluent in the community's predominant language, can perform community-based screenings to predict CVD risk as effectively as physicians and nurses when using the non-laboratory-based Gaziano CVD risk scoring tool (15). CHWs were trained for 1-2 weeks, and results showed a 96.8% agreement between risk scores assigned by CHWs and healthcare professionals. However, a question remains whether the model taken in the global South could be transferrable to the global North, but it is at least plausible that a community-based engagement approach will be effective for increasing the uptake of CVD risk assessment, particularly in disadvantaged communities of the global North. There are examples in the global North on community engagement in health (16), and indeed the voluntary or 'third sector' have been considered key partners in the delivery of health promotion initiatives in the community (17).

Authors have argued that because of the current economic constraints with the formal healthcare system, the focus should be upon supplementing a service delivery model with an alternative community development model (18). The key aspect is supplementing formal service delivery by utilizing communities' 'social capital'. The term 'social capital' describes the various resources that people may have through their relationships in families, communities and other social networks. Social capital bonds people together and helps them make links beyond their immediate friends and neighbours (19).

For this compassionate community approach to work, contextual appropriateness and cultural sensitivity of an intervention is crucial (20). Following this argument, the SPICES project in two areas of England, East Sussex and Nottingham, will co-produce a multi-component community-engagement intervention focussed on delivering a Health Check-style CVD risk screening, with appropriate health coaching and follow-up, in a community setting (21) and delivered by community volunteers. The intervention will be trialled and evaluated using a mixed methods approach using both qualitative and quantitative methods. The specific objectives of the project are:

To evaluate with stakeholders the potential for a community engagement-based CVD primary prevention programme to support or enhance the NHS Health Check Programme.

To co-produce with the communities an evidence-informed community-engagement intervention on CVD risk, based on the NHS Health Check model, tailored to the context in disadvantaged communities in East Sussex and Nottingham.

To implement the intervention in the local communities where it was co-produced, and:
-assess its effectiveness versus routine care.
-assess the fidelity, feasibility, acceptability, uptake and scalability of the implementation.
-carry out a process evaluation of the intervention and its implementation

This project is part of the SPICES (Scaling-up Packages of Interventions for Cardiovascular disease prevention in selected sites in Europe and Sub-Saharan Africa) project (22). This is a Horizon 2020 project financed by the European Commission that aims to address the CVD burden. The overall objective is to implement and evaluate a comprehensive cardiovascular disease (CVD) prevention and care program at the community level in five countries (Belgium, France, Uganda, UK, South Africa), to identify and compare barriers and facilitators for implementation across study contexts and to develop a learning community.

Methods:

Theoretical Model
SPICES is underpinned by the Consolidated Framework for Advancing Implementation Research (23), and Reach, Effectiveness, Adoption, Implementation, and Maintenance (sustainability) framework /RE-AIM models (24). We also recognize as a global health project the need for the use of the socio-ecological framework (25). As mentioned above, this model allows an understanding of the multifaceted and interactive effects of personal, social and environmental factors that determine behaviour; and for identifying behavioural and organisational leverage points and intermediaries for health promotion within organisations and communities.

Study Design
A mixed-methods research methodology will be applied strategically combining qualitative and quantitative methods at both sites. This approach will allow us to model the iterative nature of coproduction and implementation research without compromising the rigour of the study (26; 27). The study will take place in three phases:
- Pre-intervention; when stakeholder mapping and local adaptation will be carried out
- Intervention roll out, recruitment and evaluation
- Post-intervention evaluations and feedback (28)- Process evaluation will be conducted in all three phases.

Stage 1: To explore the implementation context and co-produce the intervention.
To explore the context where the implementation will take place we will carry out several mappings. These will give us the context for recruitment and implementation co-design.
They are as follows:
(a) Mapping the potential stakeholders: Mapping of the stakeholders will be done to find out who are the key stakeholders, where they come from, and what they are looking for in relationship to the study objectives(29). To engage the community, it is essential to map the community stakeholders (civil society organisations) as they are the gatekeepers of the community. Three levels of stakeholder mapping will be carried out, namely at macro, meso and micro levels.
Macro-level: stakeholders will be identified via the existing link of PI of the project in the community through meetings with local public health or other relevant departments and CSOs and using online information. Interviews with this category of stakeholders will provide insights into implementation sustainability.
Meso-level: a strategic community volunteer organisation mapping will be carried out to find out the relevant organisations, through which individual volunteers will be selected. This will

be done in three ways; using online searches, personal contacts and snowballing. In-depth interviews will be conducted to co-design a sustainable intervention implementation.
Micro-level: an exploration will be done with volunteers and end-user groups to co-design an acceptable and feasible intervention implementation.
(b) Mapping the context: social mapping will be carried out to explore the lifestyle context of the community via observations.
(c) Training of volunteers by professional health trainers and researchers following current NICE Public health guideline [PH6] 'Behaviour change: general approaches' (30)
(d) CVD risk profiling by trained community health volunteers (CHV).
CHVs will be the persons who have been involved in health-related volunteering for example volunteers who worked in cancer prevention, health check, healthy lifestyle etc programme. They will be involved in the screening of the CVD risk population and implement the designed intervention.

Expected Intervention

The final elements of the intervention will be co-produced within each community setting, following the mapping exercises outlined above. As outlined in the CFAIR (23), interventions are usually composed of a core component which is essential and indispensable, and an adaptable periphery, which can and should be tailored to the specific setting and users.
Core Components: Following identification of moderate to high risk for CVD, the intervention will consist of non-clinical (non-NHS) individual or group support sessions within the community, focus on motivating behaviour change. Each participant will be supported by trained SPICES researchers or community health workers to identify behaviour change goals, produce action plans to achieve them, and problem solve in cases of unexpected outcomes. All SPICES Interventions are theoretically grounded in the theory of behaviour change and deploy the strongest evidenced Behaviour Change Techniques (BCTs) from the literature.

1. Goal Setting
2. Action Planning
3. Problem Solving
4. Motivational Interviewing
5. Feedback on progress towards goals
6. Feedback on the health impact

The use of these six BCTs are focussed in SPICES on five Target Behaviours:

1. Reduce/cease smoking
2. Increase moderate physical activity
3. Reduce fat, salt, the sugar content of the diet
4. Increase fibre, oily fish, fruit and vegetable content of the diet
5. Reduce sedentary hours

Community Adaptation: The exact elements of the support sessions will be tailored to individuals and their community context, will be determined during iterative co-design with community representatives, and will be drawn from the following (31; 32):

Step-I - Goal setting

Every participant should receive specific healthy lifestyle counselling/feedback based on their individual item InterHE ART assessment scores (the moderate group). The feedback will be based on a review of international guidelines conducted as formative work for the SPICES project intervention (33). SPICES behaviour change support sessions will be based on the best-evidenced approaches to healthy lifestyle modification and community context and preferences.

Two further screening questionnaires may be used with individuals to assess the benefit of possibly behaviour change;

- International Physical Activity Questionnaire (IPAQ, see appendix) is an internationally validated instrument to capture information about weekly physical activity habits, behaviours and routines.
- The Dietary Approaches to Stop Hypertension Questionnaire DASH-Q is a self-reporting lifestyle questionnaire (see appendix) to capture information about weekly dietary habits, routines and behaviours, based around 'Dietary Approach to Stopping Hypertension' (34).
- Current behaviours audit: Using food and physical activity diaries prepared by and provided to participants by the SPICES research team, participants will be encouraged to complete an audit of one week of current dietary and physical activity behaviours, habits and routines to establish a baseline from which goals for change and improvement can be set in negotiation with SPICES CHVs
- The ABCD self-reporting questionnaire (see appendix) to assess participant perception of personal heart health risk.
- The EQ-5D-5L internationally validated Quality of Life self-reporting questionnaire (see appendix).

Step-II - Action Planning by the participants

Participants will be asked to create an action plan with appropriate goal setting for two behaviours (diet and exercise habits) in relation to when, where and how they will undertake, for example, physical activity (based on the item stems used by Luszczynska & Schwarzer (35); when the physical activity will be performed, where it will be performed, how often it will be performed. The way goals are reached and plans recorded will be co-designed with key stakeholders.

Step III - Problem-solving

CHVs will help participants to analyse any factors which may influence their ability to achieve the goals and to generate strategies which could help them overcome these barriers.

CHVs will use Motivational Interviewing techniques about health, social and environmental, and emotional barriers and consequences. Culturally and context-sensitive information will be provided (both verbally and in the form of leaflets) about the importance of eating healthily, being physically active, and not smoking for positive outcomes on physical and mental health.

Trial of Intervention

This will be an open-label, non-controlled trial, examining fidelity, feasibility, acceptability, uptake and scalability of the intervention.

Eligible Population

Economically disadvantaged, lower socio-economic status (SES) postcodes, will be identified using the overall Index of Multiple Deprivation (36a); Participants' SES will be determined by their postcode of residence. Any resident aged 18 or above living in the study postcode areas will be eligible to take part in the baseline assessment for the study.

Study Sample Size

The sample size calculation for the quantitative study used statistical modelling for a stepped wedge design, randomising community centres over time with the InterRHEART score as the outcome (90% power for 5% significance, effect size (Cohen's D)=0.25, intracluster correlation coefficient of 0.05, control clusters crossing to intervention in 4 steps, participant autocorrelation=0.7 and cluster autocorrelation=0.9), which requires a total of at least 144 persons. This needs approximately 200-300 people across the two sites as we expect a high level of attrition (as much as 50%). At least 1500 community members will need to be screened to achieve this recruitment (37).

Recruitment of Community Health Volunteers and Trial Participants

Community Health Volunteers (CHVs) will be recruited to perform CVD risk profiling assessments through a combination of 'doorstep outreach' and 'intermediary organisation recruitment' approaches in East Sussex and through existing community and neighbourhood groups with the assistance of partners such as Self-Help UK, the Renewal Trust, Nottingham CVS and others in Nottingham.

For recruitment of trial participants, we will use similar community networks, and endeavour to use quota sampling, in that we will seek to ensure the inclusion of high, low and median income neighbourhood residents, citizens from the South Asian and African diasporas; and will encourage participants to refer others to the researchers who may be able to potentially contribute or participate in the study.

Baseline Screening of CVD Risk

Participants will fill in the validated InterHEART score to determine suitability for the trial. The non-laboratory-based InterHEART scoring tool requires minimal resources which is practical for use within the community. There is also evidence to suggest that the InterHEART can reliably predict the incidence of CVD and death in low, middle, and high-income countries for a mean follow-up of 4.1 years (38). Risk is expressed as a score from the InterHEART: 0-9 (Low risk), 10-15 (moderate risk), and 16-48 (high risk). The InterHEART scoring tool will be translated onto a mHealth platform so that the trained CHVs can easily administer them during community engagement and contact, and online data will directly reach the University repository in real time from the respondents' device.

Participants who score moderate or high risk in the baseline assessment will be invited to participate in the intervention. The moderate risk (amber) score population will be selected for participation in the intervention (=score of 10 or higher), and will fill out the self-completion survey InterHEART scoring every three months. The InterHEART scoring tool will be translated onto a mHealth platform so that the trained CHVs can easily administer them during community engagement and contact, and online data will directly reach the University repository in real time from the respondents' device (39).

Clinical Outcome and Follow-Up

The primary outcome will be the change in the risk score among people who complete the community delivered CVD risk assessment and coaching. Secondary outcomes will be gathered from participants identified as 'high risk'. Numbers of participants who a) self-referred (defined as having contacted their GP surgery requesting for a formal check-up) and b) completed the NHS Health Checks

Data collected during the trial of intervention will comprise:

- Self-reported lifestyle (modifiable and non-modifiable) risk factors gathered through survey instruments and interviews.
- Observed/measured data on all participants' age, gender, ethnicity, postcode, hip to waist ratio, gathered by trained volunteers.
- Quantitative analysis of changes in behavioural intention, target behaviours, and measurable CVD risk.

Outcomes will be assessed at three months post-intervention.

Post-intervention Qualitative Evaluation and Feedback

In the post-intervention phase, a qualitative evaluation will be carried out during which

The following implementation parameters will be assessed:

1. The impact on awareness of CVD risks and mitigating measures, amongst disadvantaged populations of a community-based, non-clinical, CVD risk scoring tool and education.
2. The impact of the community based non-clinical CVD risk scoring tool and education on motivational healthy lifestyle among disadvantaged populations.
3. The facilitators and barriers to the adoption of a community-based CVD prevention implementation programme, by target populations.
4. The perspectives of participants regarding their experience and meaning of the intervention.

These will be explored with a subset of intervention participants using focus groups or/and in-depth interview and community mapping. Participants for the qualitative component will include adult volunteers, public health stakeholders and people within the community. The community volunteers will be selected via community organisations and public health stakeholders will be selected from the same area of the research site. Community participants for the qualitative component will be selected via the community volunteers. This post-intervention qualitative study will include randomly selected trial participants.

We will be flexible in terms of the number of participants for the qualitative component. The number will be determined through the principle of saturation and diversity. However, from each site, we will aim to include at least 12 respondents and a maximum of 30 respondents from different categories (40; 41).

Process evaluation of the intervention

To assess the fidelity of the conclusions concerning the project's effectiveness, ongoing assessment, monitoring, and enhancement is important. If significant results are found, but fidelity was not assessed, it cannot be determined if the effectiveness is attributable to unintentionally added or omitted components. Bellg and colleagues (42) propose that considerations of fidelity should permeate all stages of the study: design of the study, provision of training, delivery of the intervention, receipt of the intervention, and re-enactment of skills. As a result, we will carry out a process evaluation of the project. This will be done through Process Documentation of all the stages of this project including community volunteers mapping, Healthy lifestyle counselling, action planning and problem-solving.

Thirsk and Clark (43) argue how health-care interventions need to be understood in ways that are responsive to the complexities and intricacies of programs, people and places. They emphasise the understanding of the comprehensive experience of the persons who are delivering and receiving the intervention. Process Evaluation is a tool that can capture the intervention experience. We will be following the model designed by Moore et al (44):

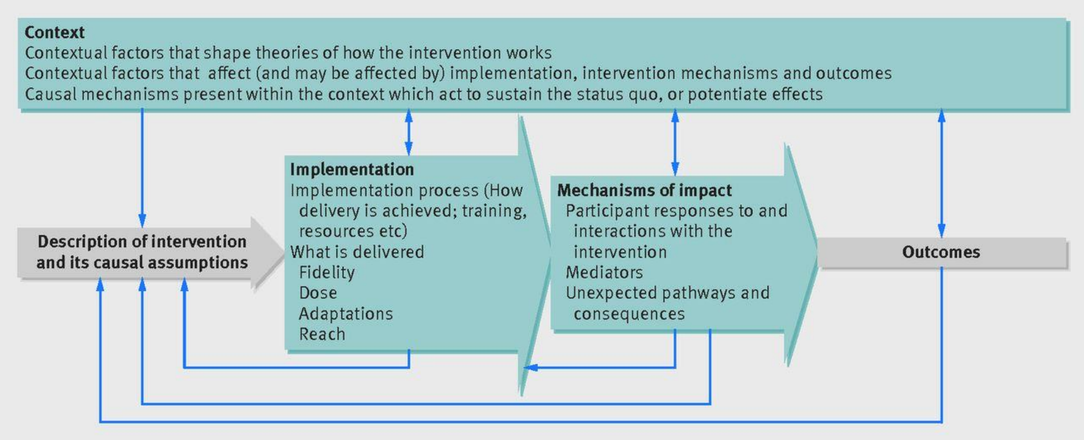

Data Analysis:
Quantitative data will be analysed using Stata version 15 or later. Descriptive statistics will summarise outcomes before and after clusters cross over to the intervention (45. Normally distributed variables will be summarised by means and standard deviations, skewed continuous variables by medians and interquartile ranges, categorical variables by frequencies and percentages. We will estimate the treatment effect using a cross-classified linear mixed effects model. A statistical analysis plan will be agreed and signed off prior to final analysis commencing. Thematic analysis of qualitative data will be carried out using a constant comparison method of analysis, which will gather and generate ideas and categories through inductive processes. The computer package NVivo will be used for primary analysis (46). Memo writing will be carried out to describe details of the interview setting and interaction of respondent and interviewer that may not be captured in audio transcriptions. This thematic analysis has deductive and inductive elements, lending itself to multidisciplinary health research (47). The analysis framework will incorporate the key theoretical constructs and respond to the context of policy and practice to include a range of deductive themes. Further themes will be induced from the interview data.

An appropriate balance of integration between empirical data and interpretation will be ensured. The investigators will extract the meaning of the empirical data and interpret them whilst acknowledging the complexity of the phenomena of CVD risk reduction in the context of community engagement (48). This method holds links to the original data and the output allows comprehensive and transparent data analysis.

Conclusion:
Given that despite the rolling out of the NHS Health Checks programme over and above current care across the UK has not been implemented as well as it could have been, especially in some of the most disadvantaged groups prone to developing CVD, the project aims to scale-up packages of interventions for cardiovascular prevention particularly to these vulnerable populations. This interdisciplinary project includes public health, social and behavioural science approaches. The main focus aspect of this project is the deinstitutionalization of health care by operating outside of formal healthcare settings. The project will emphasise on the power of citizens, combining their efforts to generate cultures of care which complement or even compensate for the inadequacies of formal systems thus sustainable. The research project will ultimately develop a community engagement-based CVD primary prevention programme to support or enhance the performance of the NHS health care.

Funding statement:
This protocol is a contextual plan for the SPICES project in the UK. The SPICES project received funding from the European Commission through the Horizon 2020 Research and Innovation Action Grant Agreement No 733356 to implement and evaluate a comprehensive CVD prevention programme in five settings: a rural & semi-urban community in a low-income country (Uganda), middle income (South Africa) and vulnerable groups in three high-income countries (Belgium, France and United Kingdom). The funder had no role in the design, decision to publish, or preparation of the manuscript.

Availability of data and materials:
A protocol should not contain any data; it sets out the research questions and how they will be addressed.

Ethics approval and consent to participate:
This protocol has received two ethics approval from the University of Sussex, The **BSMS** Research Governance and Ethics Committee (RGEC (ER/BSMS9E3G/1)), and from Nottingham Trent University (no. TBA). All participants will be requested to consent before enrolment into the study. All participant information will be kept confidential and accessible only to the key investigative team. All published data will be anonymised and can be accessed based on a written request to the Principal Investigator.

Competing interests:
Authors declare that they have no competing interests.

Authors' contributions:
PN has written the first draft and received feedback from HvM and SA on it. PN prepared the second draft and it received feedback from LG. The third draft received feedback from all the authors. All authors read and approved the final contextual protocol (4th version).

References:

1. World Health Organisation; WHO. *Global Health Observatory (GHO) data*. 2017. Retrieved on 18th May 2019, retrieved from https://www.who.int/gho/mortality_burden_disease/causes_death/top_10/en

2. British Heart Foundation report BHF. https://www.bhf.org.uk/informationsupport/publications/policy-documents/annual-report-2017.

3. Amuzu A, Carson C, Watt HC, Lawlor DA, Ebrahim S. Influence of area and individual life course deprivation on health behaviours: findings from the British Women's Heart and Health Study. Eur J Cardiovasc Prev Rehabil. 2009;16(2): 169-73. doi: 10.1097/HJR.0b013e328325d64d.

4.Public Health England (2013). NHS Health Check implementation review and action plan Summary, https://assets.publishing.service.gov.uk/government/uploads/system/uploads/attachment_data/file/224536/NHS_Health_Check_implementation_review_and_action_plan_summary_web.pdf 5.

5. QRISK2. 2017. https://qrisk.org/2017/

6. Public Health, England. NHS Health Checks 'prevented thousands of heart attacks'. 2016. https://www.nhs.uk/news/heart-and-lungs/nhs-health-checks-prevented-thousands-of-heart-attacks/

7. Public Health England. Factsheet: Implementation of the NHS Health Check programme. 2014. https://www.england.nhs.uk/wp-content/uploads/2014/02/pm-fs-3-1.pdf

8. Waterall Jamie. PHE. Getting Serious About CVD Prevention. Reducing Variation & Optimising Care. 2018. https://www.healthcheck.nhs.uk/seecmsfile/?id=195

9. Dalton AR, Bottle A, Okoro C, Majeed A, Millett C. Uptake of the NHS Health Checks programme in a deprived, culturally diverse setting: cross-sectional study. J Public Health (Oxf). 2011; 33(3):422-9. doi: 10.1093/pubmed/fdr034.

10. Robson J, Dostal I, Sheikh A, et al. The NHS Health Check in England: an evaluation of the first 4 years. 2015. BMJ Open. 2015;6: e008840. doi:10.1136/ bmjopen-2015

11. Harte Emma, Calum MacLure, Adam Martin, Catherine L Saunders, Catherine Meads, Fiona M Walter, Simon J Griffin, Jonathan Mant and Juliet A Usher-Smith (2018), Reasons why people do not attend NHS Health Checks: a systematic review and qualitative synthesis, British Journal of General Practice. 2018;68 (666): e28-e35. DOI: https://doi.org/10.3399/bjgp17X693929

12. CTSA. Principles of Community Engagement, Second Edition, NIH Publication No. 11-7782. 2011. https://.atsdr.cdc.gov/communityengagement/pdf/PCE_Report_508_FINAL.pdf

13. O'Mara-Eves A., G Brunton, D McDaid, S Oliver, J Kavanagh, F Jamal, T Matosevic, A Harden, and J Thomas. Community engagement to reduce inequalities in health: a systematic review, meta-analysis and economic analysis, Public Health Research. 2013;1:4

14. Bird JA; McPhee SJ, Ha NT, Le B, Davis T, Jenkins CN. Opening pathways to cancer screening for Vietnamese-American women: lay health workers hold a key. Prev Med. 1998;27(6):821-9.

15. Gaziano Thomas A, Shafi ka Abrahams-Gessel, Catalina A Denman, Carlos Mendoza Montano, Masuma Khanam, Thandi Puoane, Naomi S Levitt (2015). An assessment of community health workers' ability to screen for cardiovascular disease risk with a simple, non invasive risk assessment instrument in Bangladesh, Guatemala, Mexico, and South Africa: an observational study. Lancet Glob Health. 2015; 3:e556–63. http://dx.doi.org/10.1016/ S2214-109X(15)00142-4

16. Wood, J. The University as a Public Good: Active citizenship and university community engagement. International Journal of Progressive Education. 2012; 8 (3), 15-31. Retrieved from http://dergipark.org.tr/ijpe/issue/26312/277299

17. Kings Fund. 2011. Retrieved from, https://www.kingsfund.org.uk/sites/default/files/Voluntary-and-community-sector-in-health-implications-NHS-reforms-The-Kings-Fund-june-2011_0.pdf

18. Abel, J., Bowra, J., Walter, T. et al. Compassionate Community networks: Supporting home dying. BMJ Supportive and Palliative Care. 2011;1,129-133. http://dx.doi.org/10.1136/bmjspcare-2011-000068

19. Baum FE and Ziersch AM. Social capital. Journal of Epidemiology & Community Health. 2003; 57: 320-323.

20. Sallnow L. Libby, Heather Richardson, Scott Murray, Allan Kellehear. Understanding the impact of a new public health approach to end of life care: a qualitative study of a community-

led intervention. The Lancet. 2017; 389: Special issue 788. DOI:https://doi.org/10.1016/S0140-6736(17)30484-1

21. Boyd, Stephen McKernon, Bernie Mullin, Andrew Old. Improving healthcare through the use of co-design. The New Zeeland Medical Journal. 2012; 125:(1357).

22. Musinguzi Geofrey, Rhoda K. Wanyenze1, Rawlance Ndejjo, Isaac Ssinabulya, Harm van Marwijk, Isaac Ddumba, Hilde Bastiaens and Fred Nuwaha. An implementation science study to enhance cardiovascular disease prevention in Mukono and Buikwe districts in Uganda: a stepped-wedge design. Health Service research. 2019; 19:253 doi.org/10.1186/s12913-019-4095-0

23. Damschroder Laura J, David C Aron, Rosalind E Keith, Susan R Kirsh, Jeffery A Alexander and Julie C Lowery. Fostering implementation of health services research findings into practice: a Consolidated Framework for Advancing Implementation Science. Implementation Science. 2009; 4:50 doi:10.1186/1748-5908-4-50

24. Glasgow RE, McKay HG, Piette JD, Reynolds KD: The RE-AIM framework for evaluating interventions: what can it tell us about approaches to chronic illness management? Patient Educ Couns. 2001;44:119-127.

25. Theobald, S., Brandes, N., Gyapong, M., et al. Implementation research: new imperatives and opportunities in global health, Lancet. 2018;392: 2214–28

26. Peters DH, Adam T, Alonge O, et al. Implementation research: what it is and how to do it. BMJ. 2013; 347: f6753.

27. Brown CH, Curran G, Palinkas LA, et al. (2017). An overview of research and evaluation designs for dissemination and implementation. Annual Review of Public Health. 2017; 38:1–22

28. Greene, J. C. Mixed Methods in Social Inquiry. 2007. Jossey-Bass. San Francisco.

29. BSR. Stakeholder Mapping (2011). www.bsr.org/reports/BSR_Stakeholder_Engagement_Stakeholder_Mapping.final.pdf

30. NICE Public health guideline [PH6] 'Behaviour change: general approaches' (https://www.nice.org.uk/guidance/ph6/resources/behaviour-change-general-approaches-pdf-55457515717).

31. Michie Susan, Michelle Richardson, Marie Johnston, et al. (2013). The Behaviour Change Technique Taxonomy (v1) of 93 Hierarchically Clustered Techniques: Building an International Consensus for the Reporting of Behavior Change Interventions. Annals of Behavioural Medicine. 2013. 46;(1):81–95, https://doi.org/10.1007/s12160-013 9486-6

32. Michie, Susan, Maartje M. Van Stralen, and Robert West. "The Behaviour Change Wheel: A New Method for Characterising and Designing Behaviour Change Interventions. Implementation Science. 2011;6,1:42.

33. Yusuf Salim, Steven Hawken, Stephanie Ôunpuu, et al., on behalf of the INTERHEART Study Investigators. Effect of potentially modifiable risk factors associated with myocardial infarction in 52 countries (the INTERHEART study): a case-control study. Lancet. 2004; 364: 937–52

34. Sacks Frank M., Laura P. Svetkey, M.D., William M. Vollmer et al. Effects on Blood Pressure of Reduced Dietary Sodium and the Dietary Approaches to Stop Hypertension (DASH) Diet.
N Engl J Med. 2001; 344:3-10, DOI:10.1056/NEJM200101043440101

35. Luszczynska Aleksandra, Urte Scholz & Ralf Schwarzer. The General Self-Efficacy Scale: Multicultural Validation Studies, The Journal of Psychology. 2005; 139:5, 439-457, doi:10.3200/JRLP.139.5.439-457

36.. Kontopantelis E, Mamas MA, Van Marwijk H, Ryan AM, Buchan IE, Ashcroft DM, et al. Geographical epidemiology of health and overall deprivation in England, its changes and persistence from 2004 to 2015: A longitudinal spatial population study. 2018; 72 (2):140–7.

37. Hooper R, Teerenstra S, de Hoop E & Eldridge S. Sample size calculation for the stepped wedge and other longitudinal cluster randomised trials. Statist. Med. 2016; 35 4718–4728

38. Yusuf Salim, Sumathy Rangarajan, Koon Teo, Shofiqul Islam, et al. Cardiovascular. Risk and Events in 17 Low-, Middle-, and High-Income Countries. N Engl J Med. 2014; 371:818-827, DOI: 10.1056/NEJMoa1311890

39. Williams Natalie Joseph, Amy Lloyd, Adrian Edwards, Lynne Stobbart, David Tomson, Sheila Macphail, Carole Dodd, Kate Brain, Glyn Elwyn, Richard Thomson. Implementing shared decision making in the NHS: lessons from the MAGIC programme. BMJ. 2017a;357:j1744 doi: https://doi.org/10.1136/bmj.j1744

40. Guest, G., Bunce, A., & Johnson, L. How Many Interviews Are Enough?: An Experiment with Data Saturation and Variability. Field Methods. 2006; 18 (1): 59–82. https://doi.org/10.1177/1525822X05279903

41. Malterud K, Siersma VD, Guassora AD. Sample Size in Qualitative Interview Studies: Guided by Information Power. Qual Health Res. 2015; 1–8.

42. Bellg, A. J., Borrelli, B., Resnick, B., Hecht, J., Minicucci, D. S., Ory, M., et al. Treatment Fidelity Workgroup of the NIH Behavior Change Consortium. (2004). Enhancing Treatment Fidelity in Health Behavior Change Studies: Best Practices and Recommendations From the NIH Behavior Change Consortium. Health Psychology. 2004; 23(5):443-451. http://dx.doi.org/10.1037/0278 6133.23.5.443

43. Thirsk Lorraine M. and Alexander M. Clark. Using Qualitative Research for Complex Interventions: The Contributions of Hermeneutics. International journal of qualitative methods. 2017; 16:1-10. doi.org/10.1177/1609406917721068

44. Moore Laurence, Alicia O'Cathain, Tannaze Tinati, Daniel Wight, Janis Baird (2015). Process evaluation of complex interventions: Medical Research Council guidance. The BMJ. 2015;350:h1258. doi: 10.1136/bmj.h1258

45. StataCorp. 2017. *Stata Statistical Software: Release 15*. College Station, TX: StataCorp LLC

46. Ritchie Jane, Jane Lewis, Carol McNaughton Nicholls, Rachel Ormston. Qualitative Research Practice: A Guide for Social Science Students and Researcher; 2013. Sage Publications.

47. Gale NK, Heath G, Cameron E, Rashid S, Redwood S. Using the framework method for the analysis of qualitative data in multi-disciplinary health research. BMC Med Res Methodol. 2013;(13):117. doi.org/10.1186/1471-2288-13-117

48. Green J, Thorogood N. Qualitative methods for health research. 2005. London: Sage Publications Ltd.

Authors Information:

1. Papreen Nahar. Department of Primary Care and Public Health, Brighton and Sussex Medical School. University of Sussex, UK.
1. Harm van Marwijk. Department of Primary Care and Public Health, Brighton and Sussex Medical School. The University of Sussex. UK
2. Linda Gibson: School of Social Sciences. Nottingham Trent University, UK
3. Musinguzi Geofrey. Department of Disease Control and Environmental Health, School of Public Health, College of Health Sciences. Makerere University, Uganda
4. Sibyl Anthierens. Department of Primary and Interdisciplinary Care, University of Antwerp, Belgium
1. Elizabeth Ford. Department of Primary Care and Public Health Brighton and Sussex Medical School. University of Sussex, UK
1. Stephen A Bremner. Department of Primary Care and Public Health Brighton and Sussex Medical School. University of Sussex, UK
2. Mark Bower. School of Social Sciences, Nottingham Trent University, UK
5. JY Reste. Faculté de médecine et des sciences de la santé, Université de Bretagne Occidentale, Brest, France
6. Sodi Tholene. Department of Psychology. University of Limpopo, South Africa
4. Hilde Bastiaens. Department of Primary and Interdisciplinary care. University of Antwerp, Belgium
