# Supplementary material for: Psychometric evaluation of the ‘Attitudes and Beliefs about Cardiovascular Disease (ABCD) Risk Questionnaire’ with validation of a previously untested ‘Intentions and Beliefs around Smoking’ subscale
Source: BMJ Open. 2023 Jan 13;13(1):e054532. doi: 10.1136/bmjopen-2021-054532 (PMC9843199; doi:10.1136/bmjopen-2021-054532)
Supplement: Author's manuscript [file bmjopen-2021-054532.draft_revisions.pdf]

BMJ Open is committed to open peer review. As part of this commitment we make the peer review history of every article we publish publicly available.

When an article is published we post the peer reviewers' comments and the authors' responses online. We also post the versions of the paper that were used during peer review. These are the versions that the peer review comments apply to.

The versions of the paper that follow are the versions that were submitted during the peer review process. They are not the versions of record or the final published versions. They should not be cited or distributed as the published version of this manuscript.

BMJ Open is an open access journal and the full, final, typeset and author-corrected version of record of the manuscript is available on our site with no access controls, subscription charges or pay-per-view fees (http://bmjopen.bmj.com).

If you have any questions on BMJ Open's open peer review process please email info.bmjopen@bmj.com

BMJ Open

Validating a previously untested 'Intentions and Beliefs around Smoking' sub-scale for inclusion in the published 'Attitudes and Beliefs about Cardiovascular Disease (ABCD) Risk Questionnaire' using a cross-sectional sample

Journal:	*BMJ Open*
Manuscript ID	bmjopen-2021-054532
Article Type:	Original research
Date Submitted by the Author:	24-Jun-2021
Complete List of Authors:	Bowyer, Mark; Nottingham Trent University<br>Hassen, Hamid; University of Antwerp,<br>Bastiaens, Hilde; University of Antwerp Faculty of Medicine and Health Sciences, Department of Primary and Interdisciplinary Care<br>Gibson, Linda ; Nottingham Trent University, ; Nottingham trent university
Keywords:	PUBLIC HEALTH, STATISTICS & RESEARCH METHODS, PREVENTIVE MEDICINE

SCHOLARONE™
Manuscripts

I, the Submitting Author has the right to grant and does grant on behalf of all authors of the Work (as defined in the below author licence), an exclusive licence and/or a non-exclusive licence for contributions from authors who are: i) UK Crown employees; ii) where BMJ has agreed a CC-BY licence shall apply, and/or iii) in accordance with the terms applicable for US Federal Government officers or employees acting as part of their official duties; on a worldwide, perpetual, irrevocable, royalty-free basis to BMJ Publishing Group Ltd ("BMJ") its licensees and where the relevant Journal is co-owned by BMJ to the co-owners of the Journal, to publish the Work in this journal and any other BMJ products and to exploit all rights, as set out in our licence.

The Submitting Author accepts and understands that any supply made under these terms is made by BMJ to the Submitting Author unless you are acting as an employee on behalf of your employer or a postgraduate student of an affiliated institution which is paying any applicable article publishing charge ("APC") for Open Access articles. Where the Submitting Author wishes to make the Work available on an Open Access basis (and intends to pay the relevant APC), the terms of reuse of such Open Access shall be governed by a Creative Commons licence – details of these licences and which Creative Commons licence will apply to this Work are set out in our licence referred to above.

Other than as permitted in any relevant BMJ Author's Self Archiving Policies, I confirm this Work has not been accepted for publication elsewhere, is not being considered for publication elsewhere and does not duplicate material already published. I confirm all authors consent to publication of this Work and authorise the granting of this licence.

For peer review only

TITLE PAGE

Validating a previously untested 'Intentions and Beliefs around Smoking' sub-scale for inclusion in the published 'Attitudes and Beliefs about Cardiovascular Disease (ABCD) Risk Questionnaire' using a cross-sectional sample

Mark Bowyer, Nottingham Trent University (Corresponding Author)

mark.bowyer@ntu.ac.uk **(ORCID** 0000-0002-1474-5711)

Nottingham Trent University School of Social Sciences

Chaucer Building, Burton Street

Nottingham NG1 4BT

Tel: (+1) 7786 993405 Fax: (+1)115 8485574

Hamid Yimam Hassen, University of Antwerp, Belgium

hamid.hassen@uantwerpen.be **(ORCID** 0000-0001-6485-4193)

Dr Hilde Bastiaens
Associate professor
Dept Family Medicine and Population Health
Faculty of Medicine and Health Sciences
University of Antwerp

Tel: 0032 (0)3 265.29.10 Fax: 0032 (0)3 265.25.26 Hilde.bastiaens@uantwerpen.be

Dr Linda Gibson

Associate Professor in Public Health, Institute of Health & Allied Professions

Nottingham Trent University Linda.gibson@ntu.ac.uk

Key words

Cardiovascular Diseases

- Cardiovascular risk factors
- Instrumentation

Psychometrics

- Surveys and questionnaires
- Instrumentation

Primary prevention

- Instrumentation

Word count 3957

For peer review only - http://bmjopen.bmj.com/site/about/guidelines.xhtml

Validating a previously untested 'Intentions and Beliefs around Smoking' sub-scale for inclusion in the published 'Attitudes and Beliefs about Cardiovascular Disease (ABCD) Risk Questionnaire' using a cross-sectional sample

ABSTRACT

Objectives:

To provide evidence of validity, reliability and generalisability of results obtained using the Attitudes and Beliefs about Cardiovascular Disease (ABCD) Risk Questionnaire with a sample of the English population surveyed within the 'SPICES' Horizon 2020 project (Nottingham study site), and to specifically evaluate the psychometric and factor properties of an as-yet untested 5 item sub-scale relating to smoking behaviours.

Design and setting:

Community based cross-sectional study in Nottingham, UK.

Participants:

466 English adults fitting inclusion criteria (aged 18+, without known history of CVD, not pregnant, able to provide informed consent) were included in the study.

Methods:

We re-validated the published ABCD questionnaire on a sample of the general population in Nottingham to confirm the psychometric properties. Furthermore, we introduced 5 items related to smoking which were dropped in the original study due to inadequate valid samples.

Primary and secondary outcome measures:

- Psychometric and factor performance of untested 5 item 'smoking behaviours' sub-scale
- Psychometric and factorial properties in combination with the remaining 18 items across 3 sub-scales

Results:

Analyses of the data largely confirmed the validity, reliability, and factor structure of the original ABCD Risk Questionnaire. Sufficient participants in our study provided data against an additional five smoking related items to confirm their validity as a sub-scale and to advocate for their inclusion in future applications of the scale. EFA and CFA calculations support some minor changes to the remaining sub-scales which may further improve psychometric performance and therefore generalisability of the instrument.

Conclusions:

An amended version of the ABCD Risk Questionnaire would provide public health researchers and practitioners with a brief, easy to use, reliable and valid survey tool. The amended tool may now assist public health practitioners and researchers to quickly survey patient or public intentions and beliefs around three key areas of individually modifiable risk (Physical Activity, Diet, and Smoking).

For peer review only - http://bmjopen.bmj.com/site/about/guidelines.xhtml

Trial registration:

ISRCTN68334579 https://doi.org/10.1186/ISRCTN68334579
Heart health without a doctor: an implementation study of CVD prevention and behaviour change interventions in community settings

Ethical approval

Ethical approval for the 'SPICES' Nottingham study protocol (incorporating the ABCD Risk Questionnaire) was secured from the Nottingham Trent University College of Business, Law and Social Sciences on the 20th February 2019. Participants were required to provide informed consent (Appendix 4).

Article summary

Strengths and Limitations of this study

- Large sample (n=466) of English adults from the Nottingham UK population
- Sufficient case data to validate additional sub-scale related to attitudes and intentions of smokers
- Criterion validity not explored
- Full assessment of the utility of ABCD Risk Questionnaire in health promotion and CVD prevention not explored, further studies may be required to position the tool in clinical and public health practice.

Original protocol (Appendix 3)

Funding statement

This work was supported by the European Commission Horizon 2020 Non-communicable diseases and the challenge of healthy ageing Grant agreement 733356 'SPICES'.

Competing interests statement

None declared

Patient and public involvement

Patients and/or the public were not involved in the design, or conduct, or reporting, or dissemination plans of this research.

Patient consent for publication (data sharing agreement)

Not required (participant information and informed consent attached Appendix 4)

Provenance and peer review

Not commissioned.

Data availability statement

Data are available on reasonable request

Keywords

For peer review only - http://bmjopen.bmj.com/site/about/guidelines.xhtml

Cardiovascular diseases- Cardiovascular risk factors

Cardiovascular diseases- Instrumentation

Psychometrics- Instrumentation

Surveys and questionnaires- Instrumentation

Primary prevention- Instrumentation

Author contributions

Mark Bowyer: Design of work, acquisition of data, analysis and interpretation of data, drafting and revising the paper, final approval, accountability for accuracy and integrity.

Hamid Hassen: Analysis and interpretation of data, drafting and interpretation of results, accountability for accuracy and integrity.

Acknowledgements

The authors would like to acknowledge the cooperation of Rolls-Royce plc Hucknall Site; Nottingham City Council Adult Care in providing access to employees. Crabtree Farm Community Centre, Middle Street Resource Centre, Self-Help UK, in facilitating access to members, users and premises.

INTRODUCTION

Scientific Background and Rationale

In the UK, Cardiovascular Disease (CVD) is responsible for over 130,000 deaths per annum.[1] CVD morbidity is also the biggest contributor to the inequalities in Healthy Life Expectancy between members of the wealthiest neighbourhoods and the most deprived.[2] In 2009 the NHS Health Check [3]was established and more recently (2019) the CVD Prevent initiative to implement 'upstream' interventions for the prevention of CVD morbidity.[4] Both of these initiatives seek to improve early case-finding to prevent avoidable strokes and heart attacks. Both recognise the importance of supported lifestyle change in conjunction with drug therapies.

Lifestyle or behavioural change requires a degree of individual agency and commitment which drug therapies do not. Unhealthy lifestyle behaviours are linked to culture and habit, environment, emotions, and confidence which can all moderate an individual's readiness to change and the commitment required to sustain those changes over time.[5] Understanding the attitudes and beliefs that people hold towards diet, exercise and smoking, as well as their perception of their own risk could assist primary care and public health professionals in providing relevant and effective behavioural advice and social prescribing options. To support evaluations of the NHS Health Check programme, in 2017 a questionnaire was developed to evaluate patients' awareness of

For peer review only - http://bmjopen.bmj.com/site/about/guidelines.xhtml

cardiovascular disease risk at University College London.[6] This ABCD Risk Questionnaire attempts to provide a short survey drawing from the dominant theoretical models of behaviour change (Trans-Theoretical Model, Health Beliefs Model),[7] covering diet, smoking, exercise and alcohol behaviours, and incorporating a conceptual spread of perceived risk from immediate to lifetime.

Specific Objectives

In this study we re-validated the tool on a sample of the general population in Nottingham to confirm the psychometric and factorial properties. Furthermore, we introduced 5 items related to smoking which were dropped in the original study due to inadequate case numbers.

To the best of our knowledge, this is the first study which has incorporated items relating to attitudes and intentions towards stopping smoking into the published version of the ABCD Risk Questionnaire and collected sufficient data to submit them to analysis of validity, reliability and factor structure.

In the original ABCD study, over the course of three stages of validity testing (content, face, reliability) items relating to alcohol use and smoking were rejected, leaving four final sub-scales: Knowledge of CVD Risks; Perceived Risk of Heart Attack/ Stroke; Perceived Benefits and Intentions to Change; and Healthy Eating Intentions. During Exploratory Factor Analysis (EFA) none of the items relating to alcohol use achieved strong enough loadings to be included in the final scale, and items related to smoking could not be included due to the high proportion of missing data in the experimental sample. The authors of the study note this limitation '*the questionnaire does not encompass all aspects of CVD risk observed in the general population*' and that '*future studies examining populations at increased CVD risk can look into incorporating smoking and alcohol into the ABCD Risk Questionnaire to learn about these individuals' preconceptions and attendance of follow-up care*'.[8]

The present study

Nottingham is one of five global sites of the EU Horizon 2020 'SPICES' [9] CVD prevention implementation study which began in 2017. SPICES investigates contextual and health system barriers to the scaling up of successful behaviour change interventions for improved cardiovascular health in low, middle and high income European countries.

The SPICES Nottingham population survey carried out in 2019-20 utilised the ABCD Risk Questionnaire alongside the non-clinical INTERHEART CVD risk prediction instrument.[10] The SPICES study team chose to re-introduce 5 pre-written items relating to 'Intentions and Readiness to Stop Smoking' from the 65 item University College London (UCL) item pool into the questionnaire due to the high prevalence of smoking in the Nottingham population compared to England averages,[11] and its importance as a CVD risk.[12] This created a 31 item questionnaire.

In so doing, NTU researchers attempted to '*replicate the factor analytic process on an independent, larger sample to confirm the generalisability of (the original) findings*' as requested by the authors of the original study.[13] At the same time, we anticipated securing sufficient responses against the reintroduced 5 item 'smoking' sub-scale to analyse its reliability and validity as an integral part of future versions of the Questionnaire.

METHODS

For peer review only - http://bmjopen.bmj.com/site/about/guidelines.xhtml

Incorporating the ABCD Risk Questionnaire into the SPICES Nottingham baseline survey provided cross-sectional study data across a broad sample of adult participants. The data-set generated was therefore suitable for psychometric validation of the original and modified versions of the ABCD questionnaire.

Participants

Participants were recruited from across the Nottingham conurbation between April 2019 and March 2020 as part of the SPICES Nottingham baseline survey.[14] A purposive sampling method was employed based on community engagement. This strategy had two components:

1. engagement of citizens in neighbourhoods through existing community groups, organisations and venues, and
2. engagement of employees in the workplace through large city-based employers.

Community groups were targeted on the basis of the demographic of their membership to ensure that neighbourhoods of differing mean household income, those who are not in employment or of working age, and those from different ethnicities were included. In this way 327 participants were recruited.

Employers were targeted on the basis of workforce size, and policies relating to workforce well-being. Nottingham City Council Adult Care teams and the Rolls-Royce plc Hucknall site both responded positively and between them provided 156 participants. NTU researchers administered the SPICES Nottingham baseline survey individually within the community or workplace setting and personalised feedback about CVD risks was provided confidentially once the survey had been completed.

Materials

The SPICES baseline survey incorporated the ABCD risk questionnaire into a digitised survey instrument created in the Research Electronic Data Capture (REDCap) database system,[15] a secure web application for building and managing online surveys and databases, and the online survey responses were uploaded automatically. No participant data was stored on local devices. Both the ABCD Risk Questionnaire (Table 1) and the non-laboratory INTERHEART questionnaire were included unchanged from their published versions apart from an additional 5 items pertaining to smoking behaviour (Table 2).[16]

Table 1. Published ABCD Risk Questionnaire

Scale	Items
**Knowledge**<br><br>True/False/Don't Know<br><br>Correct score =1<br>Incorrect/ Don't know score = 0<br><br>Higher sum score= more knowledgeable/ more correct	1. One of the main causes of heart attack and stroke is stress
	2. Walking and gardening are considered types of exercise that can lower the risk of having a heart attack or stroke
	3. Moderately intense activity of 2.5 hours a week will reduce your chances of having a heart attack or stroke
	4. People who have diabetes are at higher risk of heart attack or stroke
	5. Managing your stress levels will help you to manage your blood pressure

For peer review only - http://bmjopen.bmj.com/site/about/guidelines.xhtml

about having a heart attack or stroke	6. Drinking high levels of alcohol can increase your cholesterol and triglyceride levels
	7. HDL refers to 'good' cholesterol, and LDL refers to 'bad' cholesterol
	8. A family history of heart disease is not a risk factor for high blood pressure
**Perceived Risk of Heart Attack or Stroke**<br><br>4= Strongly disagree, 3= Disagree, 2= Agree, 1= Strongly Agree; N/A= 0<br><br>Higher sum score = higher perception of risk of having a heart attack or stroke	9. I feel I will suffer from a heart attack or stroke sometime during my life
	10. It is likely that I will suffer from a heart attack or stroke in the future
	11. It is likely that I will have a heart attack or stroke some time during my life
	12. There is a good chance I will experience a heart attack or stroke in the next 10 years
	13. My chances of suffering from a heart attack or stroke in the next 10 years are great
	14. It is likely I will have a heart attack or stroke because of my past and/or present behaviours
	15. I am not worried that I might have a heart attack or stroke (**Reverse coded**)
	16. I am concerned about the likelihood of having a heart attack or stroke in the near future
**Perceived Benefits and Intentions to Change**<br><br>4= Strongly disagree, 3= Disagree, 2= Agree, 1= Strongly Agree; N/A= 0<br><br>Higher average score = Higher perceived benefits of diet and exercise and higher perceived readiness for change in regards to exercise and behaviour	17. I am thinking about exercising at least 2.5 hours a week
	18. I intend or want to exercise at least 2.5 hours a week
	19. When I exercise for at least 2.5 hours a week I am doing something good for the health of my heart
	20. I am confident that I can maintain a healthy weight by exercising at least 2.5 hours a week
	21. I am not thinking about exercising for 2.5 hours a week **(Reverse coded)**
	22. When I eat five portions of fruit and vegetables a day I am doing something good for the health of my heart
	23. Increasing my exercise to at least 2.5 hours a week will decrease my chances of having a heart attack or stroke
**Healthy Eating Intentions**<br><br>4= Strongly disagree, 3= Disagree, 2= Agree, 1= Strongly Agree; N/A= 0<br><br>Higher average score = Higher perceived readiness for change with regard to healthy dietary behaviour	24. I am confident that I can eat at least five portions of fruit and vegetables a day within the next two months
	25. I am thinking about eating at least five portions of fruit and vegetables a day
	26. I am not thinking about eating at least five portions of fruit and vegetables a day **(Reverse coded)**

The surveys were administered in the field by a team of trained researchers recruited from the NTU student body and directly supervised by the SPICES Nottingham coordinator. The surveys were accessed using dedicated tablet computers. Items were reproduced word for word and in the same

For peer review only - http://bmjopen.bmj.com/site/about/guidelines.xhtml

sequence as the original ABCD Risk Questionnaire with the additional 5 smoking items inserted after all 26 original items.

Table 2. Additional 'smoking' sub-scale

**Benefits and Intentions to Stop Smoking**<br><br>4= Strongly disagree, 3= Disagree, 2= Agree, 1= Strongly Agree; N/A= 0<br><br>Higher average score = Higher perceived readiness for change with regard to healthy dietary behaviour	27. I am thinking of stopping smoking within two months<br>28. I have reduced or stopped smoking<br>29. I intend or want to stop smoking<br>30. If I stop smoking it will reduce my chances of having a heart attack or stroke<br>31. I am not thinking about stopping smoking

Validating the sample

The baseline survey dataset was extracted from REDCap for analysis. Sample was checked for representativeness of the Nottingham population across parameters of age, gender, household income and known rates of physical activity and smoking.

Data analysis

We took the published 26-item ABCD Risk Questionnaire, introduced 5 further items relating to smoking behaviours, and administered it alongside a validated CVD risk assessment instrument (INTERHEART) to 486 individuals in Nottingham over a period of 12 months. Item, scale, and factor reliabilities were remeasured. Correlation was tested between and amongst ABCD sub-scale scores and selected INTERHEART variables, closely matching the methods applied in the original study (Appendix 6) and results were compared accordingly. After data cleansing, 466 valid cases were entered for analysis, four times the sample size of the original study.

Item and sub-scale reliabilities were tested using inter-item correlations, corrected item-total correlations and Cronbach's Alpha. [17] We performed an exploratory factor analysis (EFA) to evaluate the dimensionality of items of the original and modified risk scale with and without the smoking items.[18] The EFA was performed using the maximum likelihood extraction and varimax rotation method. [19] Sample and data adequacy was assessed using Kaiser-Meyer-Olkin (KMO) test and Bartlett's test of sphericity was performed to compare an observed correlation matrix to the identity matrix.[20] The adequate number of factors was determined using a scree plot. To further test the consistency of factors, we tested using Confirmatory Factor Analysis (CFA). We evaluated the model fit of the CFA using; the X2 test, the Tucker-Lewis and Comparative Fit Indexes and the root mean square error of approximation (RMSEA).[21] The analysis was performed using a free statistical software R version 4.0.2. UK postcodes were collected for all participants which allowed them to be sorted into income deciles using Office for National Statistics Index of Multiple Deprivation (IMD) public datasets, allowing correlations to be analysed. Case data from the

For peer review only - http://bmjopen.bmj.com/site/about/guidelines.xhtml

‘Knowledge’ sub-scale (8 items) were omitted from the analysis since they utilise a separate response format.

We used the STROBE cross sectional checklist when writing our report.[22]

RESULTS

Participants

Participation was voluntary, and self-selection may have been influenced by sensitivities around disclosure of health status and lifestyle habits forming a barrier to those with co-morbidities and socially ‘questionable’ behaviours (heavy smoking, high alcohol intake).

The sample cohort is strongly parametric, with a 49:51 percent gender split, normal distribution of age ranges (18-92), and a distribution of Socio-Economic Status (SES) which reflects known data about neighbourhood income in Nottingham. Nottingham is the 11th most deprived district in England with higher unemployment, lower education and skills, and shorter life expectancy than the national averages.[23]Using the Index of Multiple Deprivation a relative measure of deprivation across seven domains, Health and Disability s the domain on which the city does worst. Nevertheless, the mean INTERHEART predicted risk score for all 466 participants was 10.32 which closely matches the global reported mean for the instrument.[24]

Smoking sub-scale

The percentage of smokers in our sample was 15.5%. The five items in the smoking subscale are measured on the same four-point response scale as the 18 items submitted for Factor Analysis in the original published ABCD Risk Questionnaire (Strongly agree, agree, disagree, strongly disagree, and not applicable).

With the original 18 items this ‘Not Applicable’ response option was not used by any of the SPICES Nottingham study participants. By contrast, within their responses to the items in the ‘smoking’ subscale, ‘Not Applicable’ was the modal answer. Participants chose the ‘N/A’ response option whenever they reported being a non-smoker. This mirrors the behaviour of the original 110 NHS Health Check attendees who formed the pilot sample cohort for the original study, leaving an insufficient number of cases to assess validity and reliability of smoking sub-scale items. In the present study, 88 cases were found where participants reported smoking behaviours and this was sufficient to enter them into analysis.

Sub-scale Alpha values, Cronbach’s Alpha if item deleted calculated for all items, inter-item correlations and corrected item-total correlations were all calculated, mirroring the analysis reported in the original study.

Interitem correlations calculated for these five items produced a range between 0.654 and 0.834. All of these five ‘smoking’ items therefore correlate with one another more strongly than recommended (<.6) and were considered for rejection. However, we found each item to be qualitatively different, and that the differences were conceptually clear and well expressed in the item wording so that no participant could be expected to confuse one with any other, and they were retained.

For peer review only - http://bmjopen.bmj.com/site/about/guidelines.xhtml

Discrimination was confirmed using item-total correlations. These fell between the range 0.751 and 0.906 meaning that all five 'smoking' sub-scale items are comfortably above the standard cut-off for acceptability of 0.3.

EFA was carried out twice, firstly with the 88 confirmed smoking cases, and then again with all cases. The first operation ensured that factor loadings were not skewed by the lower number of cases reporting smoking behaviours, the second ensured that factor loadings for the remaining sub-scales where more case data was available were not skewed by outliers.

Exploratory Factor Analysis:

We conducted EFA on the original 18-item risk perception questionnaire and the modified 23-item (with smoking items). For the original 18-item, a total of 420 samples were included in the analysis, which was sufficient for factor analysis as indicated with KMO of 0.82, which is within the recommended range (0.8 to 1). The Bartlett's Test of Sphericity was significant (X2 = 4235.007, p-value < 0.001) indicating the data is adequate for factor analysis. As a result, a three-factor solution emerged based on the Scree plot (figure 1), accounting 57.4% of the total variance. Factor loading patterns in the present analysis slightly varied from the original subscales. The domains in the original subscales were risk perception, benefit finding and healthy eating intentions. In our analysis, Item 14 ('*When I eat at least 5 portions of fruit and vegetables a day I am doing something good for the health of my heart*') showed a better loading to healthy eating intention, which was loaded to benefit finding in the original study (Appendix 1).

For the modified 23-item (including the smoking sub-scale), 88 samples were valid and included in the analysis. The KMO was 0.78, which was slightly below the recommended range, but Bartlett's Test of Sphericity was significant (X2 = 1223.459, p-value < 0.001), indicating adequacy for factor analysis. The analysis showed that the smoking items loaded to another latent construct resulting in four factors in total (figure 2).

Confirmatory Factor Analysis of the published ABCD Risk Questionnaire

A Confirmatory Factor Analysis was undertaken using the SPICES Nottingham dataset to investigate further. Conducting CFA allowed us to construct the sub-scales of the published ABCD Risk Questionnaire in a three-factor measurement model and test its fit against relevant indices. Original 18 item survey comprising three sub-scales (Perceived Risk of Heart Attack/Stroke 8 items; Perceived Benefits and Intentions to Change 7 items; Healthy Eating Intentions 3 items) were used to create measurement model in SPSS Amos 25. The model was then updated to include an additional 5 item sub-scale relating to smoking behaviours.

For peer review only - http://bmjopen.bmj.com/site/about/guidelines.xhtml

Editing the measurement model

The CFA measurement model was then reconstructed removing items which had confused participants and generated high inter-item correlations, and additionally re-assigning an item relating to dietary behaviour into the dietary behaviour sub-scale. This resulted in a four-factor model (Perceived Risk of Heart Attack/ Stroke' 6 items; 'Perceived Benefits and Intentions to Exercise' 6 items; 'Healthy Eating Intentions' 4 items, Perceived Benefits and Intentions to Reduce Smoking' 5 items).

Analysis properties were set to Estimation: Maximum Likelihood, Fit the saturated and independence models; Outputs: Minimisation history, Standardised estimates, Squared multiple correlations, Residual moments, Modification indices, Factor score weights, Covariances of estimates, Correlations of estimates, Threshold for modification indices =4. Calculated model fit estimates considered: CMIN (Chi square), p, CMIN/DF, RMR, TLI, CFI, RMSEA. Modification Indices considered: Covariances between error terms within sub-scales.

Table 3. CFA fit indices for the original and modified ABCD Questionnaire measurement models

**Original 18 item ABCD**						
CMIN	P	CMIN/DF	TLI	CFI	RMSEA	RMR
714.941	.000	5.416	.826	.850	.097	.049
**Updated 23 item ABCD with Smoking sub-scale**						
CMIN	P	CMIN/DF	TLI	CFI	RMSEA	RMR
994.931	.000	4.442	.865	.881	.086	.049
**Edited 20 item ABCD with Smoking sub-scale**						
CMIN	P	CMIN/DF	TLI	CFI	RMSEA	RMR
638.973	.000	3.896	.881	.897	.079	.052
**Modified 20 item ABCD with Smoking sub-scale**						
CMIN	P	CMIN/DF	TLI	CFI	RMSEA	RMR
385.312	.000	2.439	.941	.951	.056	.046

Similarly, in the 23-item factor analysis, item 14 was loaded to the healthy eating intention. The model fit indices showed a slight improvement as indicated in table 3.

Based on factor loading and face validity, we also tested a slightly shorter version of the questionnaire, 20-items including five smoking items and the result shows that the model fit improved (CFI=0.941; TLI=0.951; RMSEA=0.056, SRMR=0.046).

The three published factors achieved a poor fit in CFA (Table 3). Including the five smoking related items which had performed strongly in EFA as their own latent factor improved overall model fit slightly, but not to an acceptable level.

Modification of the measurement model

Reviewing modification indices and expected parameter changes for factor loadings and measurement intercepts we observed an extreme covariance value (116.812) and parameter change (.209) between two of the risk perception items ('there is a good chance that I will experience a

For peer review only - http://bmjopen.bmj.com/site/about/guidelines.xhtml

heart attack or stroke in the next 10 years' and 'my chances of suffering a heart attack or stroke in the next 10 years are great') which had caused confusion for participants in our study.

Removing one of these two items (item #13), and the two other duplicative items (items #9 & #10) from the 'perceived risk of heart attack or stroke' sub-scale retains the conceptual spread of risk embodied by the items (lifetime, 10 year, near future, behaviour related). Moving the diet related item (#22) which appears in the 'perceived benefits and intentions to change' over to the 'healthy eating intentions' sub-scale might allow greater clarity for researchers analysing results from the questionnaire. Co-varying items within sub-scales that generated values above 20 (a high cut-off due to large sample used) resulted in acceptable or good fit across all sub-scales. Each of the three behaviour related sub-scales now contain items drawn from HBM, TTM and SE models providing a sound conceptual basis for comparison. Using EFA to check these results shows the modified sub-scale structure performs better than the published version (all EFA results Appendix 1).

DISCUSSION

Inadequate knowledge and/or a gap between perceived and actual CVD risk in the population could be an obstacle to better health outcomes. Improving an individual's CVD knowledge and risk perception may be important in improving a healthy lifestyle. Measuring CVD knowledge and risk perception may be a method to initiate a healthy lifestyle intervention as well as to monitor and evaluate the impact of interventions. Following this rationale, Woringer and colleagues developed the ABCD Risk questionnaire in order to measure CVD knowledge and risk perception. In this study, we re-validated the tool on a sample of the general population in Nottingham to confirm the psychometric properties.

In this Nottingham sample the proportion of current smokers was 15.5% which is lower than the England average (18%), and lower than the Nottingham city sample average (20.6%) based on the ONS Annual Population Survey.[25] ONS notes that smoking prevalence estimates by local authority can fluctuate due to smaller sample sizes. Our SPICES Nottingham sample cohort also includes some participants from neighbouring Local Authorities with different recorded rates of smoking.

The 88 participants in this study who reported smoking is a low number for pilot testing of psychometric scales but it does exceed a 10:1 ratio of cases to variables making it reasonable to proceed to analysis.

Based on EFA and CFA, we confirmed a three-factor structure, which is somewhat similar to the original sub-scales. However, in our analysis item 14 ('*When I eat at least 5 portions of fruit and vegetables a day I am doing something good for the health of my heart*") showed a better loading to the 'healthy eating intentions' sub-scale, in contrast to the factor loading in the original study, which placed this item in the 'perceived benefits and intentions to change' sub-scale. This is the only item which loaded onto a different sub-scale when using the Nottingham dataset, all others continued to load onto their original factors although many of these loaded weakly and failed to meet usual thresholds for validity (Appendix 1). The larger numbers of participants in our dataset (466 compared to 110) provides greater statistical confidence in the reported results, and we therefore adopted this change in the Confirmatory Factor Analysis which also indicated a better fit when item 14 loaded to Healthy Eating Intentions.

For peer review only - http://bmjopen.bmj.com/site/about/guidelines.xhtml

These results suggest that the additional five smoking items perform acceptably and should be incorporated into future applications of the ABCD Risk Questionnaire.

Other observations

Researchers in the Nottingham SPICES team administering the questionnaire during fieldwork reported that three items within the ‘Perception of Risk of Heart Attack/Stroke’ sub-scale caused consistent difficulties for respondents due to apparent duplication and confusion over fine semantic differences. It was difficult for participants to see a semantic difference between statements 9, 10, 11, and 12, 13 respectively. For items 9, 10, and 11, if we agree that *suffer from* and *have* are synonymous, it is hard to differentiate between *in the future* and *some time during my life* because you would imagine that respondents will be thinking about the future in both cases.

For the questionnaire to be reliable across all sections of the population, including those with limited ability in English (whether native or non-native, first, second or additional language, etc.) who may find it particularly hard to differentiate with any confidence between different pairs/sets of statements with largely synonymous meanings, this confusion is a problem. Items 12 and 13 seem to differ mainly only in the possible interpretation of a difference of degree between *good* and *great.*

These face validity issues and their impact can be observed in the inter-item correlation results generated during item reliability analysis. In the original study, two items in the perception of risk sub-scale had been rejected due to correlations in excess of 0.6 leaving 8 items. Of these remaining 8 items half had inter-item correlations which exceeded 0.6 when tested against the Nottingham dataset. These were items 9, 10, 11, and 12 which generated inter-item correlation values of .832, .869, .616, and .729 respectively. Removing items 9, 10, and 13 does not reduce the conceptual range of the ‘perception of risk’ subscale which is framed temporally from immediate threat to lifetime risk, it simply removes the duplicate or confusing items. Testing this shortened scale with factor analysis strengthens both item and scale reliability and improves factor loadings (Appendix 1). We recommend that future versions of the English language ABCD Risk Questionnaire adopt these edits (Appendix 2).

CONCLUSIONS

The published English language version of the ABCD Risk Questionnaire, with the removal of three problematic ‘perception’ items, the shift of one item from the ‘perceived benefits and intentions to change’ sub-scale into the ‘healthy eating intentions’ sub-scale, and the addition of a 5 item ‘smoking’ sub-scale performs sufficiently well in validity, reliability and factor analysis with an independent, larger sample to confirm the generalisability of its original published findings. This result supports continued use of the ABCD Risk Questionnaire in the field of CVD prevention research and practice. The inclusion of a smoking behaviours sub-scale is likely to increase its relevance where smoking behaviours still account for a large proportion of individually modifiable CVD risk in a target population. Although criterion validity has now been established for the ‘Perception of risk of heart attack/stroke sub-scale’ by two published studies, the utility of the remaining sub-scales individually or in combination has been under-examined. Future studies should investigate the criterion validity of these sub-scales and the conceptual strength of the items and variables from which they have been composed in order to unambiguously position the resulting survey instrument and evaluate its utility in CVD prevention and treatment practices.

For peer review only - http://bmjopen.bmj.com/site/about/guidelines.xhtml

REFERENCES

1 https://www.bhf.org.uk/bhf-cvd-statistics-uk-factsheet.pdf (Accessed 10/05/2021)
2 https://www.england.nhs.uk/ourwork/clinical-policy/cvd/ (Accessed 10/05/2021)
3 Robson J, Dostal I, Sheikh A, *et al*
The NHS Health Check in England: an evaluation of the first 4 years
BMJ Open 2016;**6:**e008840. doi: 10.1136/bmjopen-2015-008840
4 Kearney M
Prevention and treatment of CVD: a new priority for the NHS
Heart 2019;**105:**1924.
5 Michie, S., van Stralen, M.M. & West, R. The behaviour change wheel: A new method for characterising and designing behaviour change interventions. *Implementation Sci* **6,** 42 (2011). https://doi.org/10.1186/1748-5908-6-42
6 Woringer M, Nielsen JJ, Zibarras L*, et al*
Development of a questionnaire to evaluate patients' awareness of cardiovascular disease risk in England's National Health Service Health Check preventive cardiovascular programme
BMJ Open 2017;**7:**e014413. doi: 10.1136/bmjopen-2016-014413

7 Health Behavior: Theory, Research, and Practice
edited by Karen Glanz, Barbara K. Rimer, K. Viswanath
8 Woringer M, Nielsen JJ, Zibarras L*, et al* IBID
9 Nahar, P., van Marwijk, H., Gibson, L. *et al.* A protocol paper: community engagement interventions for cardiovascular disease prevention in socially disadvantaged populations in the UK: an implementation research study. *glob health res policy* **5,** 12 (2020). https://doi.org/10.1186/s41256-020-0131-1
10 Yusuf S , Hawken S , Ounpuu S , *et al* . Effect of potentially modifiable risk factors associated with myocardial infarction in 52 countries (the INTERHEART study): case-control study. Lancet 2004;**364**:937–52.doi:10.1016/S0140-6736(04)17018-9
11 https://www.nottinghaminsight.org.uk/themes/health-and-wellbeing/joint-strategic-needs-assessment/adults/smoking-and-tobacco-control-2019/
12 John A Ambrose, Rajat S Barua,
The pathophysiology of cigarette smoking and cardiovascular disease: An update,
Journal of the American College of Cardiology, Volume 43, Issue 10, 2004, Pages 1731-1737, ISSN 0735-1097, https://doi.org/10.1016/j.jacc.2003.12.047.
(https://www.sciencedirect.com/science/article/pii/S0735109704004346)
13 Woringer M, Nielsen JJ, Zibarras L*, et al* IBID
14 Nahar, P., van Marwijk, H., Gibson, L. *et al.* IBID
15 Harris, P.A., et al., Research electronic data capture (REDCap)—A metadata-driven methodology and workflow process for providing translational research informatics support. Journal of Biomedical Informatics, 2009. **42**(2): p. 377-381 DOI: https://doi.org/10.1016/j.jbi.2008.08.010.
16 McGorrian C, Yusuf S, Islam S, Jung H, Rangarajan S, Avezum A, et al. Estimating modifiable coronary heart disease risk in multiple regions of the world: the INTERHEART Modifiable Risk Score. European Heart Journal. 2011;32(5):581-9. doi: 10.1093/eurheartj/ehq448.
17 Cronbach, L.J. Coefficient alpha and the internal structure of tests. *Psychometrika* **16,** 297–334 (1951). https://doi.org/10.1007/BF02310555

18 Ferguson, E. and Cox, T. (1993), Exploratory Factor Analysis: A Users'Guide. International Journal of Selection and Assessment, 1: 84-94. https://doi.org/10.1111/j.1468-2389.1993.tb00092.x

19 Kaiser, H.F. The varimax criterion for analytic rotation in factor analysis. Psychometrika 23, 187–200 (1958). https://doi.org/10.1007/BF02289233

20 Bartlett, M. S. (1951). The effect of standardization on a Chi-square approximation in factor analysis. Biometrika, 38(3/4), 337-344

21 Hu Lt, Bentler PM. Cutoff criteria for fit indexes in covariance structure analysis: Conventional criteria versus new alternatives. Structural Equation Modeling: A Multidisciplinary Journal. 1999;6(1):1-55. doi: 10.1080/10705519909540118.

For peer review only - http://bmjopen.bmj.com/site/about/guidelines.xhtml

22 von Elm E, Altman DG, Egger M, Pocock SJ, Gotzsche PC, Vandenbroucke JP. The Strengthening the Reporting of Observational Studies in Epidemiology (STROBE) Statement: guidelines for reporting observational studies.
23 Ministry of Housing, Communities & Local Government. The English indices of deprivation 2019 statistical release https://www.gov.uk/government/statistics/english-indices-of-deprivation-2019 (accessed 10/05/2021)
24 Yusuf S , Hawken S , Ounpuu S , *et al* **IBID**
25 UK Government Office for National Statistics 2019 https://www.ons.gov.uk/peoplepopulationandcommunity/healthandsocialcare/healthandlifeexpectancies/bulletins/adultsomkinghabitsgreatbritain/2018 (Accessed 10/05/2021)

Figure legends

Figure 1. 18 item ABCD Questionnaire scree plot results from Nottingham dataset

Figure 2. Modified ABCD Questionnaire 20 items with smoking scree plot results Nottingham dataset

For peer review only

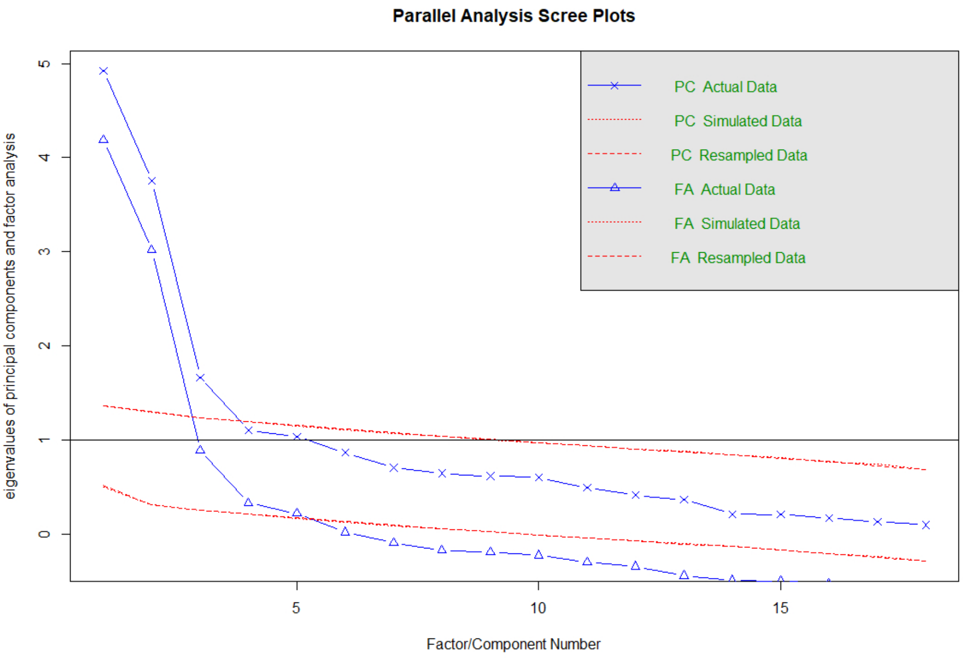

Figure 1. 18 item ABCD Questionnaire scree plot results from Nottingham dataset

286x198mm (96 x 96 DPI)

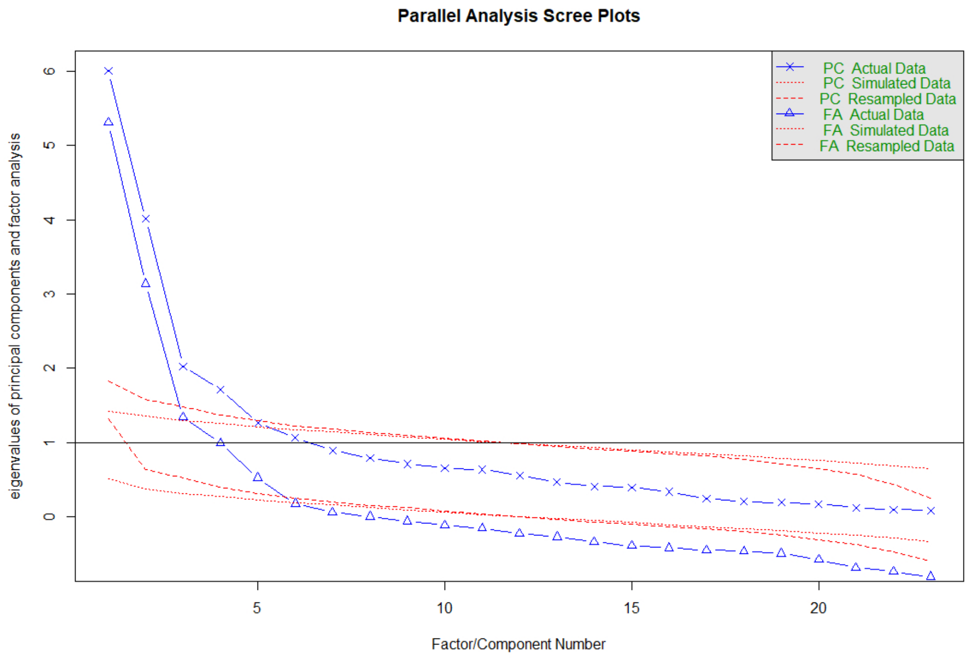

Figure 2. Modified ABCD Questionnaire 20 items with smoking scree plot results Nottingham dataset

286x198mm (96 x 96 DPI)

Without smoking items –

Non-missing samples: 420

Bartlett's Test of Sphericity (X2 = 4235.007, p-value < 0.001)

The overall KMO is 0.82, which is within the recommended range (0.8 to 1).

EFA results

- The root mean square of the residuals (RMSR) is 0.05
- Tucker Lewis Index of factoring reliability = 0.77
- RMSEA index = 0.121 and the 90 % confidence intervals are 0.113 0.129
- BIC = 165.35

Scree plot

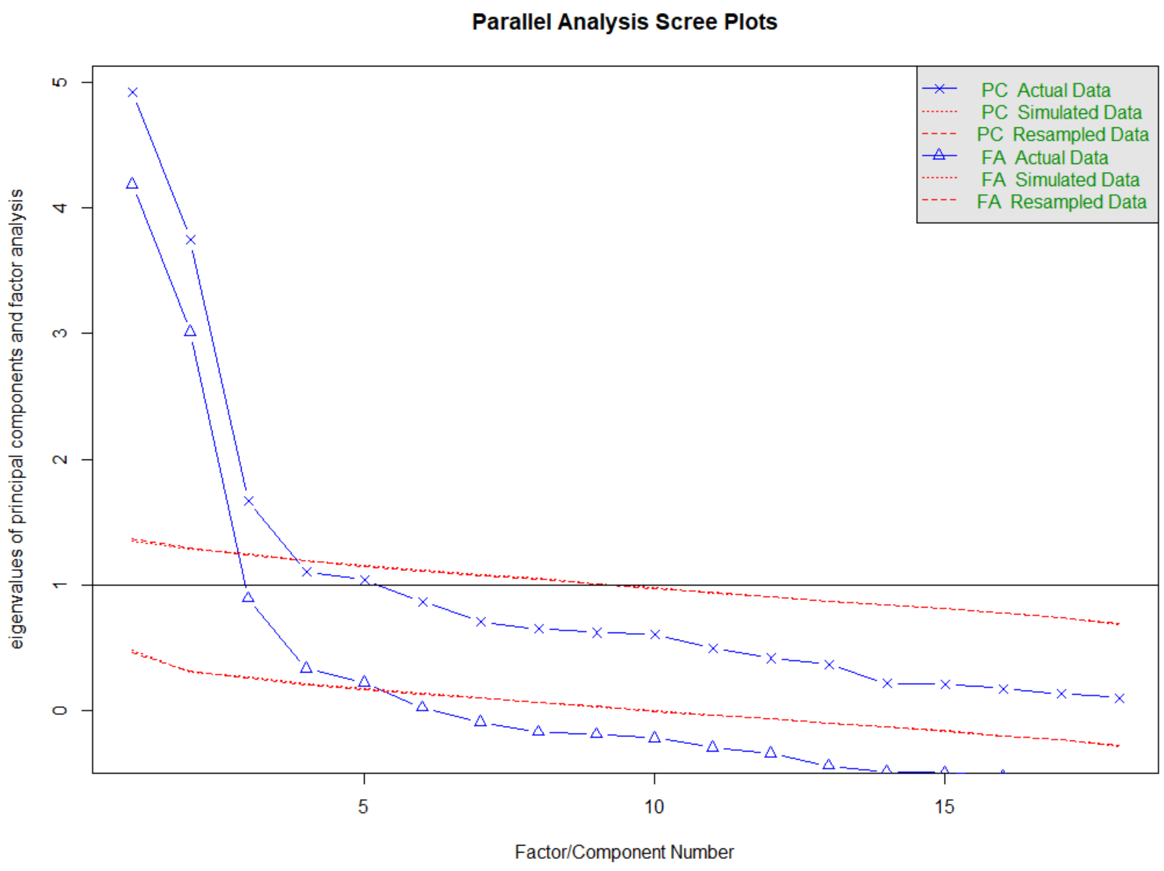

Factor loadings

Table ___. Factor loadings of the exploratory factor analysis of the risk scale without the smoking items

Item	Factor2	Factor1	Factor3	communalit	uniqueness
suffer_heartattack	0.86	0.02	-0.03	0.74	0.26
hrtattack_stroke_future	0.91	0.05	0.00	0.82	0.18
attck_stoke_during_life	0.88	0.01	0.01	0.77	0.23
hrtattack_next_10yrs	0.73	-0.07	0.01	0.55	0.45
highchance_hrtattck_10yrs	0.65	-0.10	0.01	0.44	0.56
hrtattack_past_fut_behav	0.56	-0.03	-0.01	0.32	0.68
reversenoworry	0.28	-0.11	0.10	0.10	0.90
concern_hrtattack	0.40	-0.02	0.11	0.16	0.84
think_exercise	-0.02	0.87	-0.06	0.73	0.27
want_exercise	-0.01	0.91	-0.04	0.80	0.20
exercise_gud_hrt_hlth	0.02	0.69	0.10	0.53	0.47
confident_hlth_wgt	-0.05	0.45	0.19	0.31	0.69
revnotthinkPA	0.04	0.56	0.05	0.34	0.66
fruit_veg_gud_hrthlth	0.02	0.37	0.35	0.36	0.64
high_exerc_low_hrtattack	0.02	0.39	0.27	0.30	0.70
diet_1	-0.04	0.07	0.64	0.46	0.54
diet_2	0.01	-0.01	0.93	0.85	0.15
revdiet3	-0.01	-0.03	0.78	0.60	0.40

	Factor 2	Factor 1	Factor 3
**SS loadings**	3.86	3.04	2.28
**Proportion Var**	0.21	0.17	0.13
**Cumulative Var**	0.21	0.38	0.51
**Proportion Explained**	0.42	0.33	0.25
**Cumulative Proportion**	0.42	0.75	1.00

With smoking item

For peer review only

Non-missing samples: 88

The overall KMO is 0.78, which is slightly below the recommended range (0.8 to 1).

The Bartlett's test of Sphericity is significant (X2 = 1223.459, p-value < 0.001), indicating the sample adequacy for factor analysis.

EFA results

- The root mean square of the residuals (RMSR) is 0.06
- Tucker Lewis Index of factoring reliability = 0.69
- RMSEA index = 0.129 and the 90 % confidence intervals are 0.124 and 0.136
- BIC = 440.9

Scree plot

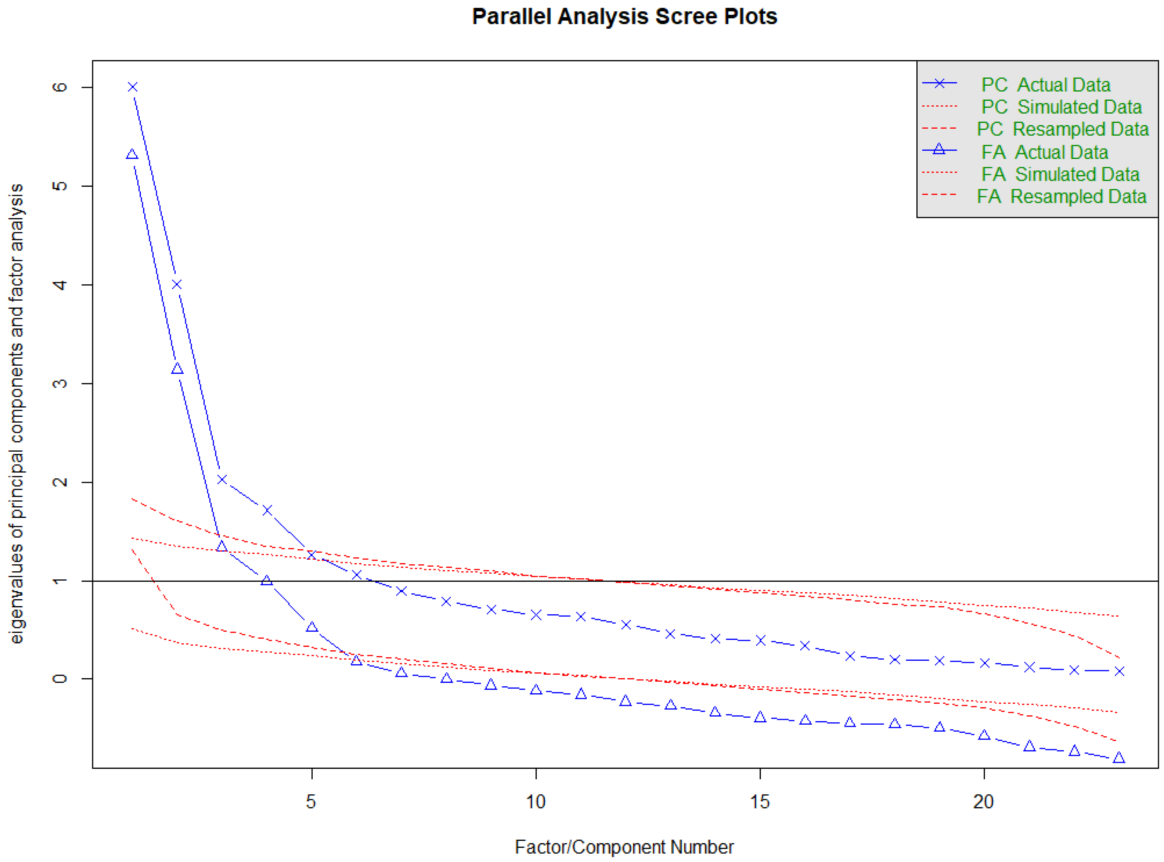

Factor loadings

Table ___. Factor loadings of the exploratory factor analysis of the ABCD Questionnaire with the smoking items

Item	Factor2	Factor3	Factor1	Factor4	Communality	Uniqueness
suffer_heartattack	0.86	-0.1	0.05	-0.02	0.76	0.24
hrtattack_stroke_future	0.91	0.06	0.02	-0.01	0.82	0.18
attck_stoke_during_life	0.88	0.02	0	0	0.77	0.23
hrtattack_next_10yrs	0.72	0	-0.09	0.01	0.54	0.46
highchance_hrtattck_10yrs	0.64	-0.03	-0.1	0.01	0.45	0.55
hrtattack_past_fut_behav	0.57	-0.07	0	0	0.33	0.67
reversenoworry	0.28	0.02	-0.14	0.1	0.1	0.9
concern_hrtattack	0.41	0.19	-0.12	0.08	0.19	0.81
think_exercise	-0.03	-0.05	0.88	-0.02	0.73	0.27
want_exercise	-0.02	0.05	0.87	-0.02	0.79	0.21
exercise_gud_hrt_hlth	0.03	0.17	0.62	0.09	0.55	0.45
confident_hlth_wgt	-0.05	0.09	0.42	0.18	0.32	0.68
revnotthinkPA	0.02	0	0.53	0.09	0.33	0.67
fruit_veg_gud_hrthlth	0.04	0.07	0.35	0.35	0.36	0.64
high_exerc_low_hrtattack	0.04	0.12	0.37	0.24	0.32	0.68
diet_1	-0.04	-0.05	0.12	0.64	0.45	0.55
diet_2	0.01	0	0.02	0.89	0.8	0.2
revdiet3	-0.01	0	-0.06	0.83	0.66	0.34
smoking_1	0.06	0.78	0.12	-0.06	0.67	0.33
smoking_2	-0.03	0.83	0.02	-0.01	0.71	0.29
smoking_3	-0.05	0.9	-0.02	-0.01	0.8	0.2
smoking_4	0.16	0.58	0.09	0.08	0.43	0.57
revsmoke5	-0.12	0.56	-0.2	0.17	0.35	0.65

	**Factor 2**	**Factor 3**	**Factor 1**	**Factor 4**
**SS loadings**	3.90	3.00	2.97	2.33
**Proportion Var**	0.17	0.13	0.13	0.10
**Cumulative Var**	0.17	0.30	0.43	0.53
**Proportion Explained**	0.32	0.25	0.24	0.19
**Cumulative Proportion**	0.32	0.57	0.81	1.00

For peer review only

Modified scale (20-items including the smoking items)

Non-missing samples: 89

The overall KMO is 0.79, which is slightly below the recommended range (0.8 to 1).

The Bartlett's test of Sphericity is significant (X2 = 915.41, p-value < 0.001), indicating the sample adequacy for factor analysis.

EFA results

- The root mean square of the residuals (RMSR) is 0.06
- Tucker Lewis Index of factoring reliability = 0.72
- RMSEA index = 0.118 and the 90 % confidence intervals are 0.111 and 0.126
- BIC = 153.72

Scree plot

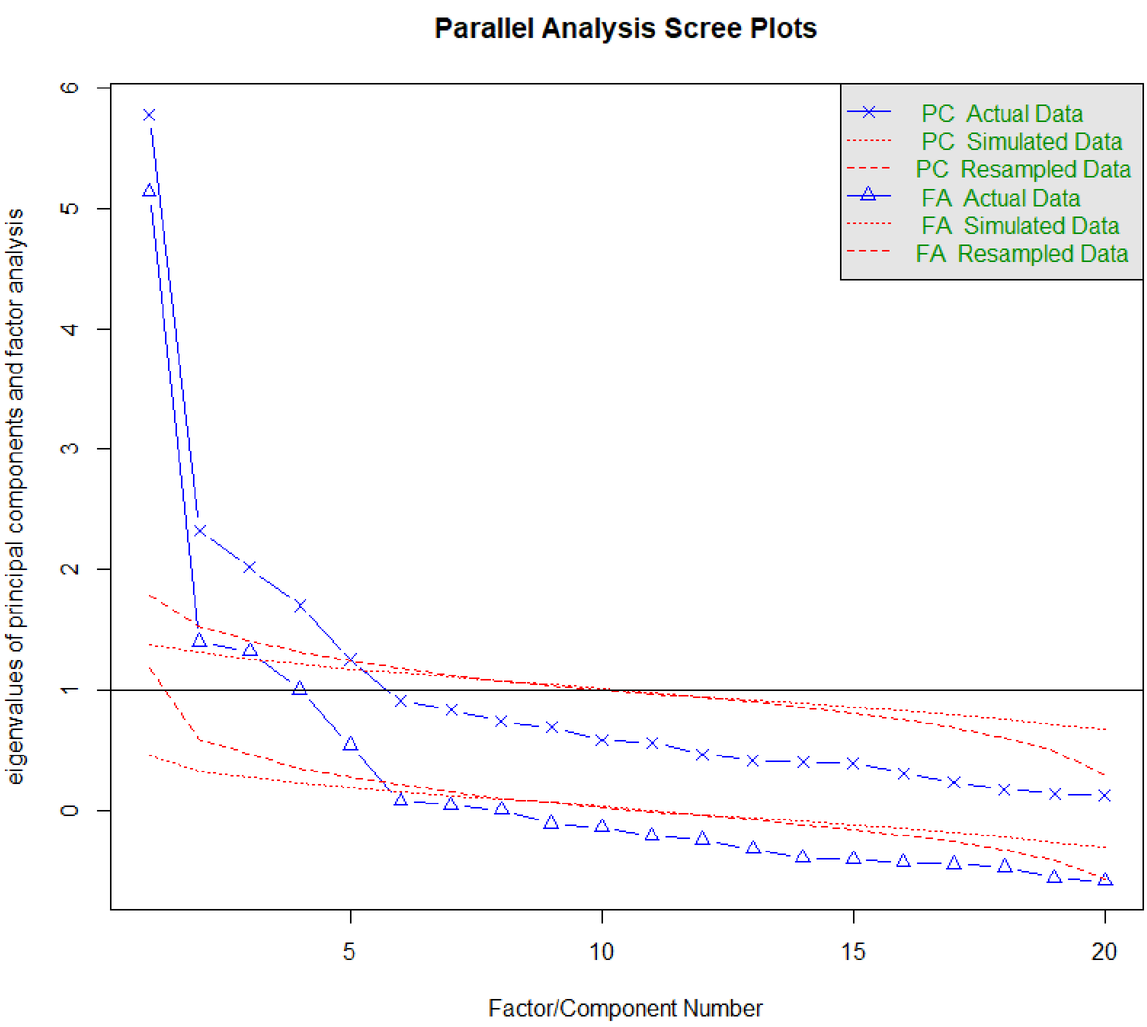

Table ___. Factor loadings of the exploratory factor analysis of the modified ABCD questionnaire (20 items including the smoking items)

Item	Factor3	Factor1	Factor4	Factor2	Communality	Uniqueness
suffer_heartattack	-0.08	0.04	-0.03	0.76	0.60	0.40
hrtattack_next_10yrs	0.02	-0.08	-0.01	0.68	0.48	0.52
hrtattack_past_fut_behav	-0.04	0.01	-0.01	0.61	0.38	0.62
reversenoworry	0.04	-0.13	0.10	0.35	0.14	0.86
concern_hrtattack	0.22	-0.11	0.07	0.45	0.23	0.77
think_exercise	-0.06	0.88	-0.02	-0.04	0.74	0.26
want_exercise	0.05	0.87	-0.02	-0.02	0.79	0.21
exercise_gud_hrt_hlth	0.17	0.62	0.09	0.04	0.55	0.45
confident_hlth_wgt	0.09	0.42	0.18	-0.06	0.32	0.68
revnotthinkPA	0.01	0.53	0.09	0.03	0.32	0.68
fruit_veg_gud_hrthlth	0.08	0.35	0.35	0.07	0.37	0.63
high_exerc_low_hrtattack	0.13	0.37	0.24	0.06	0.32	0.68
diet_1	-0.06	0.12	0.64	-0.05	0.46	0.54
diet_2	0.00	0.02	0.89	0.01	0.80	0.20
revdiet3	0.00	-0.06	0.83	-0.01	0.67	0.33
smoking_1	0.78	0.12	-0.06	0.04	0.66	0.34
smoking_2	0.83	0.02	-0.01	-0.03	0.70	0.30
smoking_3	0.89	-0.02	-0.01	-0.07	0.80	0.20
smoking_4	0.59	0.10	0.07	0.18	0.43	0.57
revsmoke5	0.56	-0.20	0.17	-0.10	0.34	0.66

	**Factor3**	**Factor1**	**Factor4**	**Factor2**
**SS loadings**	3.00	2.96	2.33	1.80
**Proportion Var**	0.15	0.15	0.12	0.09
**Cumulative Var**	0.15	0.30	0.41	0.50
**Proportion Explained**	0.30	0.29	0.23	0.18
**Cumulative Proportion**	0.30	0.59	0.82	1.00

For peer review only

Modified ABCD Risk Questionnaire

Mark Bowyer, Hamid Hassen

Scale	Items	Coding
**Perceived Risk of Heart Attack or Stroke**	1. It is likely that I will have a heart attack or stroke sometime in my life	4= Strongly disagree, 3= Disagree, 2= Agree, 1= Strongly Agree; N/A= 0
	2. There is a good chance I will experience a heart attack or stroke in the next 10 years	4= Strongly disagree, 3= Disagree, 2= Agree, 1= Strongly Agree; N/A= 0
	3. It is (more) likely I will have a heart attack or stroke because of my past and/or present behaviours	4= Strongly disagree, 3= Disagree, 2= Agree, 1= Strongly Agree; N/A= 0
	4. I am not worried that I might have a heart attack or stroke	**REVERSE CODED** 4= Strongly disagree, 3= Disagree, 2= Agree, 1= Strongly Agree; N/A= 0
	5. I am concerned about the likelihood of having a heart attack or stroke in the near future	4= Strongly disagree, 3= Disagree, 2= Agree, 1= Strongly Agree; N/A= 0
**Perceived Benefits and Intentions to Exercise**	6. I am thinking about exercising at least 2.5 hours a week	4= Strongly disagree, 3= Disagree, 2= Agree, 1= Strongly Agree; N/A= 0
	7. I intend or want to exercise at least 2.5 hours a week	4= Strongly disagree, 3= Disagree, 2= Agree, 1= Strongly Agree; N/A= 0
	8. When I exercise for at least 2.5 hours a week I am doing something good for the health of my heart	4= Strongly disagree, 3= Disagree, 2= Agree, 1= Strongly Agree; N/A= 0
	9. I am confident that I can maintain a healthy weight by exercising at least 2.5 hours a week	4= Strongly disagree, 3= Disagree, 2= Agree, 1= Strongly Agree; N/A= 0
	10. I am not thinking about exercising for 2.5 hours a week	**REVERSE CODED** 4= Strongly disagree, 3= Disagree, 2= Agree, 1= Strongly Agree; N/A= 0
	11. Increasing my exercise to at least 2.5 hours a week will decrease my chances of having a heart attack or stroke	4= Strongly disagree, 3= Disagree, 2= Agree, 1= Strongly Agree; N/A= 0

For peer review only

**Perceived Benefit and Healthy Eating Intentions**	12. I am confident that I can eat at least five portions of fruit and vegetables a day within the next two months	4= Strongly disagree, 3= Disagree, 2= Agree, 1= Strongly Agree; N/A= 0
	13. I am thinking about eating at least five portions of fruit and vegetables a day	4= Strongly disagree, 3= Disagree, 2= Agree, 1= Strongly Agree; N/A= 0
	14. I am not thinking about eating at least five portions of fruit and vegetables a day	**REVERSE CODED** 4= Strongly disagree, 3= Disagree, 2= Agree, 1= Strongly Agree; N/A= 0
	15. When I eat five portions of fruit and vegetables a day I am doing something good for the health of my heart	4= Strongly disagree, 3= Disagree, 2= Agree, 1= Strongly Agree; N/A= 0
**Benefits and Intentions to Stop Smoking**	16. I am thinking of stopping smoking within two months	4= Strongly disagree, 3= Disagree, 2= Agree, 1= Strongly Agree; N/A= 0
	17. I have reduced or stopped smoking	4= Strongly disagree, 3= Disagree, 2= Agree, 1= Strongly Agree; N/A= 0
	18. I intend or want to stop smoking	4= Strongly disagree, 3= Disagree, 2= Agree, 1= Strongly Agree; N/A= 0
	19. If I stop smoking it will reduce my chances of having a heart attack or stroke	4= Strongly disagree, 3= Disagree, 2= Agree, 1= Strongly Agree; N/A= 0
	20. I am not thinking about stopping smoking	**REVERSE CODED** 4= Strongly disagree, 3= Disagree, 2= Agree, 1= Strongly Agree; N/A= 0

For peer review only

A Protocol Paper: Community engagement interventions for Cardiovascular Disorders prevention in socially disadvantaged populations in the UK: An implementation research study
Final 15072019

Target Journal: Journal of Global Health Research and Policy
https://ghrp.biomedcentral.com/?gclid=Cj0KCQiA68bhBRCKARIsABYUGifuKd-xktjcmV7tn3r7G-IEqS5rAb6QmiEl6P9dXGBdNRDhsIPVzA0aAiJWEALw_wcB

Papreen Nahar[1], Harm van Marwijk[1], Linda Gibson[2], Geofrey Musinguzi[3], Sibyl Anthierens[4], Elizabeth Ford [1], Stephen A Bremner [1], Mark Bower [2], Jean Yves Le Reste[5], Tholene Sodi [6], Hilde Bastiaens[4]

Corresponding author: Dr Papreen Nahar, Department of Primary Care and Public Health, Brighton and Sussex Medical School, UK. The University of Sussex. E-mail: P.Nahar@bsms.ac.uk

Abstract:

Cardiovascular disorders (CVD) are the single greatest cause of mortality worldwide. In the UK, the National Health Service (NHS) has launched an initiative of health checks over and above current care to tackle CVD. However, the uptake of Health Checks is poor in disadvantaged communities. This protocol paper sets out a UK-based study aiming to co-produce a community delivered CVD risk assessment and coaching intervention to support community members to reduce their risk of CVD.

The overall aim of the project is to implement a tailored-to-context community engagement (CE) intervention on awareness of CVD risks in vulnerable populations in high, middle and low-income countries. This paper describes the protocol for the UK sites in Sussex and Nottingham. The specific objectives of the study are to enhance stakeholder' engagement; to implement lifestyle interventions for cardiovascular primary prevention, in disadvantaged populations and motivate uptake of NHS health checks.

This study takes a mixed methods approach, combining qualitative and quantitative methods in three phases of evaluation, including pre-, during- and post-implementation. To ensure contextual appropriateness the SPICES project will organize a multi-component community-engagement intervention implementation. For the qualitative component, the pre-implementation phase will involve a contextual assessment and stakeholder mapping, exploring potentials for CVD risk profiling strategies and led by trained Community Health Volunteers (CHV) to identify accessibility and acceptability. The during-implementation phase will involve healthy lifestyle counselling provided by CHVs and evaluation of the outcome to identify fidelity and scalability. The post-implementation phase will involve developing sustainable community-based strategies for CVD risk reduction. All three components will include a process evaluation. The theory of the socio-ecological framework will be applied to analyse the community engagement approach.

A stepped wedge quantitative evaluation of the roll out will focus on implementation outcomes such as uptake and engagement and changes in risk profiles. The quantitative component includes pre and post-intervention surveys.

The research project will ultimately develop a sustainable community engagement-based strategy for the primary prevention of CVD, to support or enhance the performance of NHS health care.

For peer review only - http://bmjopen.bmj.com/site/about/guidelines.xhtml

Key words: Implementation research, Cardiovascular disorders prevention, community engagement.

Introduction:

Cardiovascular disorders (CVD) are the single greatest cause of mortality worldwide each year, estimated to contribute to 31% of all deaths globally (1). Tackling CVD is an international priority and there have been many global initiatives such as the "Global Hearts" programme, a package launched by the World Health Organisation (WHO) and partners, to enhance the prevention and control of CVD. Some risk factors for CVD are non-modifiable, such as age, ethnicity and family history (2). Some other risk factors for CVD are modifiable, such as smoking, a lack of physical activity, being overweight, lower consumption of fruit and vegetables, high blood pressure, diabetes and high cholesterol (2). These risk factors can be changed through lifestyle or behavioural modifications. There is evidence of a social gradient in the prevalence of CVD, which points to associations between social and financial deprivation, vulnerability and risk factors for CVD. (3).

In 2015, CVD was the leading cause of mortality in the context of all chronic diseases, accounting for 27% and 25% of deaths in men and women respectively, in the UK(2). Coronary heart disease (CHD) and stroke were the main CVDs responsible for this mortality of men and women across all ages. As per British Heart Foundation report in 2017 CVD has a huge financial burden with annual associated healthcare costs estimated to be £9 billion annually in the UK (2). The UK has a standardised CVD death rate of 265.1 per 100,000 (2).

In the UK, the National Health Service (NHS) has launched the Health Check initiative aimed to prevent CVD. It is a national risk assessment and management program, free to adults aged 40 to 74 living in England, who do not currently have any vascular disorders and are not being treated for certain risk factors such as diabetes (4). It aims to assess the 10-year risk of CV events and disorders. Risk is assessed using QRISK2 (5), a tool which involves collection of the following information: age, gender, ethnicity, smoking status, family history of CHD, body mass index (BMI), cholesterol test, systolic and diastolic blood pressure, levels of physical activity, and alcohol consumption. Attendees receive a low (<10 % chance of event in 10 years), medium (>10 % but <20 %), or high (>20 %) 10-year cardiovascular (QRISK2) score. Above the 10% cut-off, attendees are offered a discussion with a qualified person, such as a nurse, about lifestyle and motivation to change, which may include goal setting and plans for follow up. Patients may also be offered medication for cholesterol and blood pressure. The NHS Health Check is recommended to be undertaken every five years.

Modelling predicted that the NHS Health Check could prevent 1,600 heart attacks and strokes each year if implemented as intended (6). Whilst evidence suggests that the Health Check programme has the potential to reduce CVD events and has therefore been rolled out nationally across the UK, its implementation has been poor, especially in some of the most disadvantaged groups at highest risk of developing CVD. In 2014, Public Health England (PHE) issued a call for action to increase the uptake rate of NHS Health Checks to 75% (7) and to increase awareness of risk and engagement with existing resources. Yet, as of 2017, current uptake remains far from this target with current predictions suggesting only 40% of the eligible population will receive one (8), due to the fact that uptake is low (48%) even when Health Checks are offered. (8) (9)

Data from some regions with very large ethnic minority community and socio-economically challenged populations showed that only 45% of patients who were invited for the check attended and subsequently received some form of counselling when they needed it. Authors have discussed how higher uptake in deprived communities would reduce the possibility of exacerbation of inequalities (10). Difficulty with accessing general practices, especially among socially vulnerable groups, has been highlighted as a common barrier to

For peer review only - http://bmjopen.bmj.com/site/about/guidelines.xhtml

attendance at Health Checks (11). A community-based engagement approach, which takes the CVD risking profiling and affiliated advice processes outside of the formal healthcare facility setting, has the potential to improve access to Health Checks and could be an effective and scalable way for improving the implementation and uptake of Health Checks. Community engagement (CE) has been conceptualised as "the process of working collaboratively with and through groups of people affiliated by geographic proximity, special interest, or similar situations, to address issues affecting the well-being of those people" (12). A review of community engagement interventions found them to be effective in improving health behaviours (such as physical activity), health consequences and psychological outcomes (i.e. self-efficacy and perceived social support) (13). Community-based intervention programmes have been implemented to increase the uptake of cancer screening programmes. The programmes have been found to be effective in increasing outcomes such as recognition, receipt and maintenance of screening behaviours (14). The CE approach offers the opportunity for task-shifting and owning the programme, whereby trained non-healthcare-professionals can perform CVD risk profiling assessments to individuals who might not otherwise be captured by the formal care pathway.

There is evidence that CVD risk assessments can be successfully delivered by Community Health Workers (CHWs), outside or inside the healthcare system. An observational study conducted in Bangladesh, Guatemala, Mexico and South Africa has demonstrated that CHWs who are inhabitants of their local communities and were fluent in the community's predominant language, can perform community-based screenings to predict CVD risk as effectively as physicians and nurses when using the non-laboratory-based Gaziano CVD risk scoring tool (15). CHWs were trained for 1-2 weeks, and results showed a 96.8% agreement between risk scores assigned by CHWs and healthcare professionals. However, a question remains whether the model taken in the global South could be transferrable to the global North, but it is at least plausible that a community-based engagement approach will be effective for increasing the uptake of CVD risk assessment, particularly in disadvantaged communities of the global North. There are examples in the global North on community engagement in health (16), and indeed the voluntary or 'third sector' have been considered key partners in the delivery of health promotion initiatives in the community (17).

Authors have argued that because of the current economic constraints with the formal healthcare system, the focus should be upon supplementing a service delivery model with an alternative community development model (18). The key aspect is supplementing formal service delivery by utilizing communities' 'social capital'. The term 'social capital' describes the various resources that people may have through their relationships in families, communities and other social networks. Social capital bonds people together and helps them make links beyond their immediate friends and neighbours (19).

For this compassionate community approach to work, contextual appropriateness and cultural sensitivity of an intervention is crucial (20). Following this argument, the SPICES project in two areas of England, East Sussex and Nottingham, will co-produce a multi-component community-engagement intervention focussed on delivering a Health Check-style CVD risk screening, with appropriate health coaching and follow-up, in a community setting (21) and delivered by community volunteers. The intervention will be trialled and evaluated using a mixed methods approach using both qualitative and quantitative methods. The specific objectives of the project are:

To evaluate with stakeholders the potential for a community engagement-based CVD primary prevention programme to support or enhance the NHS Health Check Programme.

To co-produce with the communities an evidence-informed community-engagement intervention on CVD risk, based on the NHS Health Check model, tailored to the context in disadvantaged communities in East Sussex and Nottingham.

For peer review only - http://bmjopen.bmj.com/site/about/guidelines.xhtml

To implement the intervention in the local communities where it was co-produced, and:
-assess its effectiveness versus routine care.
-assess the fidelity, feasibility, acceptability, uptake and scalability of the implementation.
-carry out a process evaluation of the intervention and its implementation

This project is part of the SPICES (Scaling-up Packages of Interventions for Cardiovascular disease prevention in selected sites in Europe and Sub-Saharan Africa) project (22). This is a Horizon 2020 project financed by the European Commission that aims to address the CVD burden. The overall objective is to implement and evaluate a comprehensive cardiovascular disease (CVD) prevention and care program at the community level in five countries (Belgium, France, Uganda, UK, South Africa), to identify and compare barriers and facilitators for implementation across study contexts and to develop a learning community.

Methods:

Theoretical Model

SPICES is underpinned by the Consolidated Framework for Advancing Implementation Research (23), and Reach, Effectiveness, Adoption, Implementation, and Maintenance (sustainability) framework /RE-AIM models (24). We also recognize as a global health project the need for the use of the socio-ecological framework (25). As mentioned above, this model allows an understanding of the multifaceted and interactive effects of personal, social and environmental factors that determine behaviour; and for identifying behavioural and organisational leverage points and intermediaries for health promotion within organisations and communities.

Study Design

A mixed-methods research methodology will be applied strategically combining qualitative and quantitative methods at both sites. This approach will allow us to model the iterative nature of coproduction and implementation research without compromising the rigour of the study (26; 27). The study will take place in three phases:
- Pre-intervention; when stakeholder mapping and local adaptation will be carried out
- Intervention roll out, recruitment and evaluation
- Post-intervention evaluations and feedback (28)- Process evaluation will be conducted in all three phases.

Stage 1: To explore the implementation context and co-produce the intervention.

To explore the context where the implementation will take place we will carry out several mappings. These will give us the context for recruitment and implementation co-design.
They are as follows:
(a) Mapping the potential stakeholders: Mapping of the stakeholders will be done to find out who are the key stakeholders, where they come from, and what they are looking for in relationship to the study objectives(29). To engage the community, it is essential to map the community stakeholders (civil society organisations) as they are the gatekeepers of the community. Three levels of stakeholder mapping will be carried out, namely at macro, meso and micro levels.
Macro-level: stakeholders will be identified via the existing link of PI of the project in the community through meetings with local public health or other relevant departments and CSOs and using online information. Interviews with this category of stakeholders will provide insights into implementation sustainability.
Meso-level: a strategic community volunteer organisation mapping will be carried out to find out the relevant organisations, through which individual volunteers will be selected. This will

For peer review only - http://bmjopen.bmj.com/site/about/guidelines.xhtml

be done in three ways; using online searches, personal contacts and snowballing. In-depth interviews will be conducted to co-design a sustainable intervention implementation.
Micro-level: an exploration will be done with volunteers and end-user groups to co-design an acceptable and feasible intervention implementation.
(b) Mapping the context: social mapping will be carried out to explore the lifestyle context of the community via observations.
(c) Training of volunteers by professional health trainers and researchers following current NICE Public health guideline [PH6] ‘Behaviour change: general approaches’ (30)
(d) CVD risk profiling by trained community health volunteers (CHV).
CHVs will be the persons who have been involved in health-related volunteering for example volunteers who worked in cancer prevention, health check, healthy lifestyle etc programme. They will be involved in the screening of the CVD risk population and implement the designed intervention.

Expected Intervention

The final elements of the intervention will be co-produced within each community setting, following the mapping exercises outlined above. As outlined in the CFAIR (23), interventions are usually composed of a core component which is essential and indispensable, and an adaptable periphery, which can and should be tailored to the specific setting and users.
Core Components: Following identification of moderate to high risk for CVD, the intervention will consist of non-clinical (non-NHS) individual or group support sessions within the community, focus on motivating behaviour change. Each participant will be supported by trained SPICES researchers or community health workers to identify behaviour change goals, produce action plans to achieve them, and problem solve in cases of unexpected outcomes. All SPICES Interventions are theoretically grounded in the theory of behaviour change and deploy the strongest evidenced Behaviour Change Techniques (BCTs) from the literature.

1. Goal Setting
2. Action Planning
3. Problem Solving
4. Motivational Interviewing
5. Feedback on progress towards goals
6. Feedback on the health impact

The use of these six BCTs are focussed in SPICES on five Target Behaviours:

1. Reduce/cease smoking
2. Increase moderate physical activity
3. Reduce fat, salt, the sugar content of the diet
4. Increase fibre, oily fish, fruit and vegetable content of the diet
5. Reduce sedentary hours

Community Adaptation: The exact elements of the support sessions will be tailored to individuals and their community context, will be determined during iterative co-design with community representatives, and will be drawn from the following (31; 32):

Step-I - Goal setting

Every participant should receive specific healthy lifestyle counselling/feedback based on their individual item InterHE ART assessment scores (the moderate group). The feedback will be based on a review of international guidelines conducted as formative work for the SPICES project intervention (33). SPICES behaviour change support sessions will be based on the best-evidenced approaches to healthy lifestyle modification and community context and preferences.

For peer review only - http://bmjopen.bmj.com/site/about/guidelines.xhtml

Two further screening questionnaires may be used with individuals to assess the benefit of possibly behaviour change;

- International Physical Activity Questionnaire (IPAQ, see appendix) is an internationally validated instrument to capture information about weekly physical activity habits, behaviours and routines.
- The Dietary Approaches to Stop Hypertension Questionnaire DASH-Q is a self-reporting lifestyle questionnaire (see appendix) to capture information about weekly dietary habits, routines and behaviours, based around 'Dietary Approach to Stopping Hypertension' (34).
- Current behaviours audit: Using food and physical activity diaries prepared by and provided to participants by the SPICES research team, participants will be encouraged to complete an audit of one week of current dietary and physical activity behaviours, habits and routines to establish a baseline from which goals for change and improvement can be set in negotiation with SPICES CHVs
- The ABCD self-reporting questionnaire (see appendix) to assess participant perception of personal heart health risk.
- The EQ-5D-5L internationally validated Quality of Life self-reporting questionnaire (see appendix).

Step-II - Action Planning by the participants

Participants will be asked to create an action plan with appropriate goal setting for two behaviours (diet and exercise habits) in relation to when, where and how they will undertake, for example, physical activity (based on the item stems used by Luszczynska & Schwarzer (35); when the physical activity will be performed, where it will be performed, how often it will be performed. The way goals are reached and plans recorded will be co-designed with key stakeholders.

Step III - Problem-solving

CHVs will help participants to analyse any factors which may influence their ability to achieve the goals and to generate strategies which could help them overcome these barriers.

CHVs will use Motivational Interviewing techniques about health, social and environmental, and emotional barriers and consequences. Culturally and context-sensitive information will be provided (both verbally and in the form of leaflets) about the importance of eating healthily, being physically active, and not smoking for positive outcomes on physical and mental health.

Trial of Intervention

This will be an open-label, non-controlled trial, examining fidelity, feasibility, acceptability, uptake and scalability of the intervention.

Eligible Population

Economically disadvantaged, lower socio-economic status (SES) postcodes, will be identified using the overall Index of Multiple Deprivation (36a); Participants' SES will be determined by their postcode of residence. Any resident aged 18 or above living in the study postcode areas will be eligible to take part in the baseline assessment for the study.

Study Sample Size

The sample size calculation for the quantitative study used statistical modelling for a stepped wedge design, randomising community centres over time with the InterRHEART score as the outcome (90% power for 5% significance, effect size (Cohen's D)=0.25, intracluster correlation coefficient of 0.05, control clusters crossing to intervention in 4 steps, participant autocorrelation=0.7 and cluster autocorrelation=0.9), which requires a total of at least 144 persons. This needs approximately 200-300 people across the two sites as we expect a high level of attrition (as much as 50%). At least 1500 community members will need to be screened to achieve this recruitment (37).

For peer review only - http://bmjopen.bmj.com/site/about/guidelines.xhtml

Recruitment of Community Health Volunteers and Trial Participants

Community Health Volunteers (CHVs) will be recruited to perform CVD risk profiling assessments through a combination of 'doorstep outreach' and 'intermediary organisation recruitment' approaches in East Sussex and through existing community and neighbourhood groups with the assistance of partners such as Self-Help UK, the Renewal Trust, Nottingham CVS and others in Nottingham.

For recruitment of trial participants, we will use similar community networks, and endeavour to use quota sampling, in that we will seek to ensure the inclusion of high, low and median income neighbourhood residents, citizens from the South Asian and African diasporas; and will encourage participants to refer others to the researchers who may be able to potentially contribute or participate in the study.

Baseline Screening of CVD Risk

Participants will fill in the validated InterHEART score to determine suitability for the trial. The non-laboratory-based InterHEART scoring tool requires minimal resources which is practical for use within the community. There is also evidence to suggest that the InterHEART can reliably predict the incidence of CVD and death in low, middle, and high-income countries for a mean follow-up of 4.1 years (38). Risk is expressed as a score from the InterHEART: 0-9 (Low risk), 10-15 (moderate risk), and 16-48 (high risk). The InterHEART scoring tool will be translated onto a mHealth platform so that the trained CHVs can easily administer them during community engagement and contact, and online data will directly reach the University repository in real time from the respondents' device.

Participants who score moderate or high risk in the baseline assessment will be invited to participate in the intervention. The moderate risk (amber) score population will be selected for participation in the intervention (=score of 10 or higher), and will fill out the self-completion survey InterHEART scoring every three months. The InterHEART scoring tool will be translated onto a mHealth platform so that the trained CHVs can easily administer them during community engagement and contact, and online data will directly reach the University repository in real time from the respondents' device (39).

Clinical Outcome and Follow-Up

The primary outcome will be the change in the risk score among people who complete the community delivered CVD risk assessment and coaching. Secondary outcomes will be gathered from participants identified as 'high risk'. Numbers of participants who a) self-referred (defined as having contacted their GP surgery requesting for a formal check-up) and b) completed the NHS Health Checks

Data collected during the trial of intervention will comprise:

- Self-reported lifestyle (modifiable and non-modifiable) risk factors gathered through survey instruments and interviews.
- Observed/measured data on all participants' age, gender, ethnicity, postcode, hip to waist ratio, gathered by trained volunteers.
- Quantitative analysis of changes in behavioural intention, target behaviours, and measurable CVD risk.

Outcomes will be assessed at three months post-intervention.

Post-intervention Qualitative Evaluation and Feedback

In the post-intervention phase, a qualitative evaluation will be carried out during which

The following implementation parameters will be assessed:

For peer review only - http://bmjopen.bmj.com/site/about/guidelines.xhtml

1. The impact on awareness of CVD risks and mitigating measures, amongst disadvantaged populations of a community-based, non-clinical, CVD risk scoring tool and education.
2. The impact of the community based non-clinical CVD risk scoring tool and education on motivational healthy lifestyle among disadvantaged populations.
3. The facilitators and barriers to the adoption of a community-based CVD prevention implementation programme, by target populations.
4. The perspectives of participants regarding their experience and meaning of the intervention.

These will be explored with a subset of intervention participants using focus groups or/and in-depth interview and community mapping. Participants for the qualitative component will include adult volunteers, public health stakeholders and people within the community. The community volunteers will be selected via community organisations and public health stakeholders will be selected from the same area of the research site. Community participants for the qualitative component will be selected via the community volunteers. This post-intervention qualitative study will include randomly selected trial participants.

We will be flexible in terms of the number of participants for the qualitative component. The number will be determined through the principle of saturation and diversity. However, from each site, we will aim to include at least 12 respondents and a maximum of 30 respondents from different categories (40; 41).

Process evaluation of the intervention

To assess the fidelity of the conclusions concerning the project's effectiveness, ongoing assessment, monitoring, and enhancement is important. If significant results are found, but fidelity was not assessed, it cannot be determined if the effectiveness is attributable to unintentionally added or omitted components. Bellg and colleagues (42) propose that considerations of fidelity should permeate all stages of the study: design of the study, provision of training, delivery of the intervention, receipt of the intervention, and re-enactment of skills. As a result, we will carry out a process evaluation of the project. This will be done through Process Documentation of all the stages of this project including community volunteers mapping, Healthy lifestyle counselling, action planning and problem-solving.

Thirsk and Clark (43) argue how health-care interventions need to be understood in ways that are responsive to the complexities and intricacies of programs, people and places. They emphasise the understanding of the comprehensive experience of the persons who are delivering and receiving the intervention. Process Evaluation is a tool that can capture the intervention experience. We will be following the model designed by Moore et al (44):

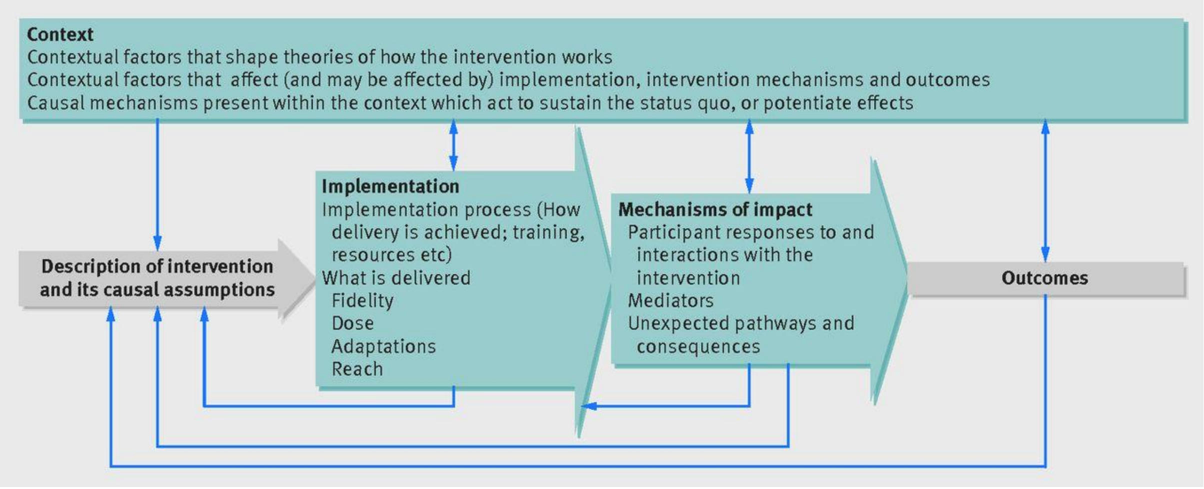

For peer review only - http://bmjopen.bmj.com/site/about/guidelines.xhtml

Data Analysis:

Quantitative data will be analysed using Stata version 15 or later. Descriptive statistics will summarise outcomes before and after clusters cross over to the intervention (45. Normally distributed variables will be summarised by means and standard deviations, skewed continuous variables by medians and interquartile ranges, categorical variables by frequencies and percentages. We will estimate the treatment effect using a cross-classified linear mixed effects model. A statistical analysis plan will be agreed and signed off prior to final analysis commencing. Thematic analysis of qualitative data will be carried out using a constant comparison method of analysis, which will gather and generate ideas and categories through inductive processes. The computer package NVivo will be used for primary analysis (46). Memo writing will be carried out to describe details of the interview setting and interaction of respondent and interviewer that may not be captured in audio transcriptions. This thematic analysis has deductive and inductive elements, lending itself to multidisciplinary health research (47). The analysis framework will incorporate the key theoretical constructs and respond to the context of policy and practice to include a range of deductive themes. Further themes will be induced from the interview data.

An appropriate balance of integration between empirical data and interpretation will be ensured. The investigators will extract the meaning of the empirical data and interpret them whilst acknowledging the complexity of the phenomena of CVD risk reduction in the context of community engagement (48). This method holds links to the original data and the output allows comprehensive and transparent data analysis.

Conclusion:

Given that despite the rolling out of the NHS Health Checks programme over and above current care across the UK has not been implemented as well as it could have been, especially in some of the most disadvantaged groups prone to developing CVD, the project aims to scale-up packages of interventions for cardiovascular prevention particularly to these vulnerable populations. This interdisciplinary project includes public health, social and behavioural science approaches. The main focus aspect of this project is the deinstitutionalization of health care by operating outside of formal healthcare settings. The project will emphasise on the power of citizens, combining their efforts to generate cultures of care which complement or even compensate for the inadequacies of formal systems thus sustainable. The research project will ultimately develop a community engagement-based CVD primary prevention programme to support or enhance the performance of the NHS health care.

Funding statement:

This protocol is a contextual plan for the SPICES project in the UK. The SPICES project received funding from the European Commission through the Horizon 2020 Research and Innovation Action Grant Agreement No 733356 to implement and evaluate a comprehensive CVD prevention programme in five settings: a rural & semi-urban community in a low-income country (Uganda), middle income (South Africa) and vulnerable groups in three high-income countries (Belgium, France and United Kingdom). The funder had no role in the design, decision to publish, or preparation of the manuscript.

Availability of data and materials:

A protocol should not contain any data; it sets out the research questions and how they will be addressed.

For peer review only - http://bmjopen.bmj.com/site/about/guidelines.xhtml

For peer review only

Ethics approval and consent to participate:
This protocol has received two ethics approval from the University of Sussex, The **BSMS** Research Governance and Ethics Committee (RGEC (ER/BSMS9E3G/1)), and from Nottingham Trent University (no. TBA). All participants will be requested to consent before enrolment into the study. All participant information will be kept confidential and accessible only to the key investigative team. All published data will be anonymised and can be accessed based on a written request to the Principal Investigator.

Competing interests:
Authors declare that they have no competing interests.

Authors' contributions:
PN has written the first draft and received feedback from HvM and SA on it. PN prepared the second draft and it received feedback from LG. The third draft received feedback from all the authors. All authors read and approved the final contextual protocol (4th version).

References:

1. World Health Organisation; WHO. *Global Health Observatory (GHO) data*. 2017. Retrieved on 18th May 2019, retrieved from https://www.who.int/gho/mortality_burden_disease/causes_death/top_10/en

2. British Heart Foundation report BHF. https://www.bhf.org.uk/informationsupport/publications/policy-documents/annual-report-2017.

3. Amuzu A, Carson C, Watt HC, Lawlor DA, Ebrahim S. Influence of area and individual life course deprivation on health behaviours: findings from the British Women's Heart and Health Study. Eur J Cardiovasc Prev Rehabil. 2009;16(2): 169-73. doi: 10.1097/HJR.0b013e328325d64d.

4.Public Health England (2013). NHS Health Check implementation review and action plan Summary, https://assets.publishing.service.gov.uk/government/uploads/system/uploads/attachment_data/file/224536/NHS_Health_Check_implementation_review_and_action_plan_summary_web.pdf 5.

5. QRISK2. 2017. https://qrisk.org/2017/

6. Public Health, England. NHS Health Checks 'prevented thousands of heart attacks'. 2016. https://www.nhs.uk/news/heart-and-lungs/nhs-health-checks-prevented-thousands-of-heart-attacks/

7. Public Health England. Factsheet: Implementation of the NHS Health Check programme. 2014. https://www.england.nhs.uk/wp-content/uploads/2014/02/pm-fs-3-1.pdf

8. Waterall Jamie. PHE. Getting Serious About CVD Prevention. Reducing Variation & Optimising Care. 2018. https://www.healthcheck.nhs.uk/seecmsfile/?id=195

For peer review only - http://bmjopen.bmj.com/site/about/guidelines.xhtml

9. Dalton AR, Bottle A, Okoro C, Majeed A, Millett C. Uptake of the NHS Health Checks programme in a deprived, culturally diverse setting: cross-sectional study. J Public Health (Oxf). 2011; 33(3):422-9. doi: 10.1093/pubmed/fdr034.

10. Robson J, Dostal I, Sheikh A, et al. The NHS Health Check in England: an evaluation of the first 4 years. 2015. BMJ Open. 2015;6: e008840. doi:10.1136/ bmjopen-2015

11. Harte Emma, Calum MacLure, Adam Martin, Catherine L Saunders, Catherine Meads, Fiona M Walter, Simon J Griffin, Jonathan Mant and Juliet A Usher-Smith (2018), Reasons why people do not attend NHS Health Checks: a systematic review and qualitative synthesis, British Journal of General Practice. 2018;68 (666): e28-e35. DOI: https://doi.org/10.3399/bjgp17X693929

12. CTSA. Principles of Community Engagement, Second Edition, NIH Publication No. 11-7782. 2011. https://.atsdr.cdc.gov/communityengagement/pdf/PCE_Report_508_FINAL.pdf

13. O'Mara-Eves A., G Brunton, D McDaid, S Oliver, J Kavanagh, F Jamal, T Matosevic, A Harden, and J Thomas. Community engagement to reduce inequalities in health: a systematic review, meta-analysis and economic analysis, Public Health Research. 2013;1:4

14. Bird JA; McPhee SJ, Ha NT, Le B, Davis T, Jenkins CN. Opening pathways to cancer screening for Vietnamese-American women: lay health workers hold a key. Prev Med. 1998;27(6):821-9.

15. Gaziano Thomas A, Shafi ka Abrahams-Gessel, Catalina A Denman, Carlos Mendoza Montano, Masuma Khanam, Thandi Puoane, Naomi S Levitt (2015). An assessment of community health workers' ability to screen for cardiovascular disease risk with a simple, non invasive risk assessment instrument in Bangladesh, Guatemala, Mexico, and South Africa: an observational study. Lancet Glob Health. 2015; 3:e556–63. http://dx.doi.org/10.1016/ S2214-109X(15)00142-4

16. Wood, J. The University as a Public Good: Active citizenship and university community engagement. International Journal of Progressive Education. 2012; 8 (3), 15-31. Retrieved from http://dergipark.org.tr/ijpe/issue/26312/277299

17. Kings Fund. 2011. Retrieved from, https://www.kingsfund.org.uk/sites/default/files/Voluntary-and-community-sector-in-health-implications-NHS-reforms-The-Kings-Fund-june-2011_0.pdf

18. Abel, J., Bowra, J., Walter, T. et al. Compassionate Community networks: Supporting home dying. BMJ Supportive and Palliative Care. 2011;1,129-133. http://dx.doi.org/10.1136/bmjspcare-2011-000068

19. Baum FE and Ziersch AM. Social capital. Journal of Epidemiology & Community Health. 2003; 57: 320-323.

20. Sallnow L. Libby, Heather Richardson, Scott Murray, Allan Kellehear. Understanding the impact of a new public health approach to end of life care: a qualitative study of a community-

For peer review only - http://bmjopen.bmj.com/site/about/guidelines.xhtml

led intervention. The Lancet. 2017; 389: Special issue 788. DOI:https://doi.org/10.1016/S0140-6736(17)30484-1

21. Boyd, Stephen McKernon, Bernie Mullin, Andrew Old. Improving healthcare through the use of co-design. The New Zeeland Medical Journal. 2012; 125:(1357).

22. Musinguzi Geofrey, Rhoda K. Wanyenze1, Rawlance Ndejjo, Isaac Ssinabulya, Harm van Marwijk, Isaac Ddumba, Hilde Bastiaens and Fred Nuwaha. An implementation science study to enhance cardiovascular disease prevention in Mukono and Buikwe districts in Uganda: a stepped-wedge design. Health Service research. 2019; 19:253 doi.org/10.1186/s12913-019-4095-0

23. Damschroder Laura J, David C Aron, Rosalind E Keith, Susan R Kirsh, Jeffery A Alexander and Julie C Lowery. Fostering implementation of health services research findings into practice: a Consolidated Framework for Advancing Implementation Science. Implementation Science. 2009; 4:50 doi:10.1186/1748-5908-4-50

24. Glasgow RE, McKay HG, Piette JD, Reynolds KD: The RE-AIM framework for evaluating interventions: what can it tell us about approaches to chronic illness management? Patient Educ Couns. 2001;44:119-127.

25. Theobald, S., Brandes, N., Gyapong, M., et al. Implementation research: new imperatives and opportunities in global health, Lancet. 2018;392: 2214–28

26. Peters DH, Adam T, Alonge O, et al. Implementation research: what it is and how to do it. BMJ. 2013; 347: f6753.

27. Brown CH, Curran G, Palinkas LA, et al. (2017). An overview of research and evaluation designs for dissemination and implementation. Annual Review of Public Health. 2017; 38:1–22

28. Greene, J. C. Mixed Methods in Social Inquiry. 2007. Jossey-Bass. San Francisco.

29. BSR. Stakeholder Mapping (2011). www.bsr.org/reports/BSR_Stakeholder_Engagement_Stakeholder_Mapping.final.pdf

30. NICE Public health guideline [PH6] 'Behaviour change: general approaches' (https://www.nice.org.uk/guidance/ph6/resources/behaviour-change-general-approaches-pdf-55457515717).

31. Michie Susan, Michelle Richardson, Marie Johnston, et al. (2013). The Behaviour Change Technique Taxonomy (v1) of 93 Hierarchically Clustered Techniques: Building an International Consensus for the Reporting of Behavior Change Interventions. Annals of Behavioural Medicine. 2013. 46;(1):81–95, https://doi.org/10.1007/s12160-013 9486-6

32. Michie, Susan, Maartje M. Van Stralen, and Robert West. "The Behaviour Change Wheel: A New Method for Characterising and Designing Behaviour Change Interventions. Implementation Science. 2011;6,1:42.

For peer review only - http://bmjopen.bmj.com/site/about/guidelines.xhtml

33. Yusuf Salim, Steven Hawken, Stephanie Ôunpuu, et al., on behalf of the INTERHEART Study Investigators. Effect of potentially modifiable risk factors associated with myocardial infarction in 52 countries (the INTERHEART study): a case-control study. Lancet. 2004; 364: 937–52

34. Sacks Frank M., Laura P. Svetkey, M.D., William M. Vollmer et al. Effects on Blood Pressure of Reduced Dietary Sodium and the Dietary Approaches to Stop Hypertension (DASH) Diet.
N Engl J Med. 2001; 344:3-10, DOI:10.1056/NEJM200101043440101

35. Luszczynska Aleksandra, Urte Scholz & Ralf Schwarzer. The General Self-Efficacy Scale: Multicultural Validation Studies, The Journal of Psychology. 2005; 139:5, 439-457, doi:10.3200/JRLP.139.5.439-457

36.. Kontopantelis E, Mamas MA, Van Marwijk H, Ryan AM, Buchan IE, Ashcroft DM, et al. Geographical epidemiology of health and overall deprivation in England, its changes and persistence from 2004 to 2015: A longitudinal spatial population study. 2018; 72 (2):140–7.

37. Hooper R, Teerenstra S, de Hoop E & Eldridge S. Sample size calculation for the stepped wedge and other longitudinal cluster randomised trials. Statist. Med. 2016; 35 4718–4728

38. Yusuf Salim, Sumathy Rangarajan, Koon Teo, Shofiqul Islam, et al. Cardiovascular. Risk and Events in 17 Low-, Middle-, and High-Income Countries. N Engl J Med. 2014; 371:818-827, DOI: 10.1056/NEJMoa1311890

39. Williams Natalie Joseph, Amy Lloyd, Adrian Edwards, Lynne Stobbart, David Tomson, Sheila Macphail, Carole Dodd, Kate Brain, Glyn Elwyn, Richard Thomson. Implementing shared decision making in the NHS: lessons from the MAGIC programme. BMJ. 2017a;357:j1744 doi: https://doi.org/10.1136/bmj.j1744

40. Guest, G., Bunce, A., & Johnson, L. How Many Interviews Are Enough?: An Experiment with Data Saturation and Variability. Field Methods. 2006; 18 (1): 59–82. https://doi.org/10.1177/1525822X05279903

41. Malterud K, Siersma VD, Guassora AD. Sample Size in Qualitative Interview Studies: Guided by Information Power. Qual Health Res. 2015; 1–8.

42. Bellg, A. J., Borrelli, B., Resnick, B., Hecht, J., Minicucci, D. S., Ory, M., et al. Treatment Fidelity Workgroup of the NIH Behavior Change Consortium. (2004). Enhancing Treatment Fidelity in Health Behavior Change Studies: Best Practices and Recommendations From the NIH Behavior Change Consortium. Health Psychology. 2004; 23(5):443-451. http://dx.doi.org/10.1037/0278 6133.23.5.443

43. Thirsk Lorraine M. and Alexander M. Clark. Using Qualitative Research for Complex Interventions: The Contributions of Hermeneutics. International journal of qualitative methods. 2017; 16:1-10. doi.org/10.1177/1609406917721068

For peer review only

44. Moore Laurence, Alicia O'Cathain, Tannaze Tinati, Daniel Wight, Janis Baird (2015). Process evaluation of complex interventions: Medical Research Council guidance. The BMJ. 2015;350:h1258. doi: 10.1136/bmj.h1258

45. StataCorp. 2017. *Stata Statistical Software: Release 15*. College Station, TX: StataCorp LLC

46. Ritchie Jane, Jane Lewis, Carol McNaughton Nicholls, Rachel Ormston. Qualitative Research Practice: A Guide for Social Science Students and Researcher; 2013. Sage Publications.

47. Gale NK, Heath G, Cameron E, Rashid S, Redwood S. Using the framework method for the analysis of qualitative data in multi-disciplinary health research. BMC Med Res Methodol. 2013;(13):117. doi.org/10.1186/1471-2288-13-117

48. Green J, Thorogood N. Qualitative methods for health research. 2005. London: Sage Publications Ltd.

Authors Information:

1. Papreen Nahar. Department of Primary Care and Public Health, Brighton and Sussex Medical School. University of Sussex, UK.
1. Harm van Marwijk. Department of Primary Care and Public Health, Brighton and Sussex Medical School. The University of Sussex. UK
2. Linda Gibson: School of Social Sciences. Nottingham Trent University, UK
3. Musinguzi Geofrey. Department of Disease Control and Environmental Health, School of Public Health, College of Health Sciences. Makerere University, Uganda
4. Sibyl Anthierens. Department of Primary and Interdisciplinary Care, University of Antwerp, Belgium
1. Elizabeth Ford. Department of Primary Care and Public Health Brighton and Sussex Medical School. University of Sussex, UK
1. Stephen A Bremner. Department of Primary Care and Public Health Brighton and Sussex Medical School. University of Sussex, UK
2. Mark Bower. School of Social Sciences, Nottingham Trent University, UK
5. JY Reste. Faculté de médecine et des sciences de la santé, Université de Bretagne Occidentale, Brest, France
6. Sodi Tholene. Department of Psychology. University of Limpopo, South Africa
4. Hilde Bastiaens. Department of Primary and Interdisciplinary care. University of Antwerp, Belgium

For peer review only - http://bmjopen.bmj.com/site/about/guidelines.xhtml

For peer review only

Horizon 2020
European Union funding
for Research & Innovation

NOTTINGHAM
TRENT UNIVERSITY

'SPICES' Heart Diseases Prevention Research

Introduction to SPICES research

Nottingham Trent University is part of an international research team investigating ways to build good practice in the prevention of Heart Diseases. Researchers and doctors have a lot of evidence about what causes heart diseases and what prevents them. Heart Diseases are now the biggest cause of death globally, and one of the leading causes of disability, so the more people know what the doctors know, the better they can protect themselves and maintain a good quality of life.

The research project is called 'SPICES' and here in Nottingham we are going to see if working with people in the community instead of at the doctor's surgery, we can spread the message quicker and further.

If you choose to take part we will ask you to complete a simple survey. From the we will be able see how well you are looking after your heart in terms of your lifestyle. Then there will be three possible options:

If the data you provide suggests you may need to make some lifestyle changes we will recommend that you make an appointment to see your doctor. As researchers we cannot give any medical advice, but it would be inappropriate for us to ignore any signs of an unhealthy lifestyle that could give rise to heart problems.

If the data you provide suggests you have a healthy lifestyle, then this is positive news and we'll talk to you about how you might be able to help the project in other ways.

If you are somewhere in the middle we will show you some simple ways to reduce your risk and stay healthier for longer.

N.B. In all cases, the data you provided is for research purposes only and a decision about your health cannot be made on the basis of questionnaires only.   Whilst we advise you to see a doctor if figures are high, lower figures should not be taken to indicate a healthy heart, and the results should not be used to replace medical assessments and the taking of medical advice about other health monitoring strategies. The dividing of participants into three groups is for research purposes only and is not a medical intervention.

If you're interested please complete our survey (It might take about 10 minutes, and you will need a tape measure for one of the questions).

Our researchers will then get in touch with you about ways that we can support you to make your heart healthier.   Any information we collect will be kept securely and not shared outside of the research team. Your name and personal details will not be used in any reports, and all our records will be destroyed at the end of the project in line with the relevant GDPR legislation. Additionally you may withdraw your data at any time up to but no later than December 31st 2020 by contacting Mark Bowyer, SPICES Coordinator, Nottingham Trent University 0115 8485574 mark.bowyer@ntu.ac.uk

OK? Let's start with your agreement to take part.

For peer review only

Horizon 2020
European Union funding
for Research & Innovation

NOTTINGHAM
TRENT UNIVERSITY

CONSENT FORM

'SPICES' Heart Diseases Prevention Research

You are making a decision to take part. By ticking ALL statements and signing your name below you will indicate that you have read the information provided above and decided to participate.

If you choose to discontinue participation in this study, you may withdraw at any time without judgement, or effect on your status.

**CONSENT STATEMENT**		**Please tick if you agree**
**1.**	I have received, read and understood the SPICES participant information sheet	
**2.**	I am aware that I can withdraw my participation at any time without prejudice, judgement or effect on my status in relation to Nottingham Trent University or its research partners	
**3.**	I understand that information I provide during my participation can be deleted at my request up to but no later than December 31st 2020	
**4.**	I agree to be contacted by SPICES researchers using the details that I have supplied below	
**5.**	I understand that the collection of data is not part of medical assessment or diagnosis and cannot be relied upon to reach conclusions as to the state of my health	
**5.**	I understand that any information I provide as part of the SPICES research will be managed in accordance with the EU General Data Protection Regulation (GDPR) framework (see SPICES participant information sheet)	
**6.**	I agree to take part in this research project	

Name:

Preferred contact details:

D.O.B.

Gender:

Postcode:

Signature:

Date:

……………………………………………………………………….

Staff signature:

Date:

For peer review only

Appendix 5. Item Analysis of published ABCD Risk Questionnaire sub-scales plus 5 unpublished items relating to smoking.

**Perceived Risk of Heart Attack/ Stroke 8 Items Cronbach's Alpha .861**	**Inter-item correlation**	**Corrected Item-total correlation**	**Cronbach's alpha if item deleted**
It is likely that I will suffer from a heart attack or stroke in the future	**.832**	.756	.826
It is likely that I will have a heart attack or stroke some time during my life	**.869**	.777	.824
I feel I will suffer a heart attack or stroke some time during my life	**.616**	.784	.824
There is a good chance I will experience a heart attack or stroke in the next 10 years	**.729**	.722	.832
I am not worried that I might have a heart attack or stroke	.403	.624	.843
My chances of suffering a heart attack or stroke in the next 10 years are great	.245	.544	.852
It is likely that I will have a heart attack or stroke because of my past/present behaviours	.266	.319	**.876**
I am concerned about the likelihood of having a heart attack or stroke in the near future	.259	.387	**.870**
**Perceived Benefits and Intentions to Change 7 items Cronbach's Alpha .801**	**Inter-item correlation**	**Corrected Item-total correlation**	**Cronbach's alpha if item deleted**
I am thinking about exercising at least 2.5 hours a week	**.727**	**.605**	.760
I intend or want to exercise at least 2.5 hours a week	.442	**.651**	.752
When I exercise for at least 2.5 hours a week I am doing something good for the health of my heart	.426	.593	.769
I am confident that I can maintain a healthy weight by exercising at least 2.5 hours a week within the next 2 months	.294	.452	.790
I am not thinking about exercising at least 2.5 hours a week	.264	.508	.781
When I eat at least 5 portions of fruit and vegetables a day I am	.483	.483	.783

For peer review only - http://bmjopen.bmj.com/site/about/guidelines.xhtml

doing something good for the health of my heart			
Increasing my exercise to at least 2.5 hours a week will decrease my chances of having a heart attack or stroke	.326	.474	.786
**Healthy Eating Intentions**<br>**3 items**<br>**Cronbach's Alpha .787**	**Inter-item correlation**	**Corrected Item-total correlation**	**Cronbach's alpha if item deleted**
I am confident that I can eat at least 5 portions of fruit and vegetables a day within the next 2 months	.555	.533	**.812**
I am thinking about eating at least 5 portions of fruit and vegetables a day	**.683**	**.732**	.596
I am not thinking about eating at least 5 portions of fruit and vegetables a day	.424	**.624**	.713
**Perceived Benefits and Intentions to Stop Smoking**<br>**5 Items**<br>**Cronbach's Alpha .943**	**Inter-item correlation**	**Corrected item-total correlation**	**Cronbach's alpha if item deleted**
I am thinking of stopping smoking within the next 2 months	**.654**	.848	.932
I have reduced or stopped smoking	**.694**	.751	**.949**
I intend or want to stop smoking	**.829**	.906	.919
If I stop smoking it will reduce my chances of having a heart attack or stroke	**.834**	.886	.922
I am not thinking about stopping smoking	**.789**	.872	.925

For peer review only

Appendix 6

ABCD subscale and selected INTERHEART variable correlation values from Nottingham study compared with values reported in the original Woringer study.

		Knowledge	Perceived Risk	Perceived Benefit	Healthy Intentions	IMD2010 Quintile	BMI/W2 Hr	Qrisk2/ INTERHEART
Knowledge	Correlation Coefficient		-.124**/ .013**	-.148**/ -.021**	-.106**/ -.039**	-.002/ .085	-.225/ -.084	-.007/ -.018
	Sig 2 tailed		.236/ .722	.175/ .645	.319/ .400	.986/ .066	.021/ .082	.941/ .714
	N		93/462	86/462	91/462	99/466	105/433	104/436
Perceived Risk	Correlation Coefficient			-.195/ -.112	-.188/ -0.36	.239/ .039	.389/ .182	.220/ .356
	Sig 2 tailed			.080/ .016	.088/ .441	.025/ .397	.000/ .000	.036/ .000
	N			82/462	84/462	87/466	92/433	91/436
Perceived Benefits	Correlation Coefficient				.533/ .383	-.287/ .071	-.068/ .000	-.118/ -.164
	Sig 2 tailed				.000/ .000	.009/ .127	.538/ .997	.284/ .001
	N				83/462	81/466	85/433	84/436
Healthy Intentions	Correlation Coefficient					-.261/ .098	.084/ .044	-.072/ -.079
	Sig 2 tailed					.016/ .034	.430/ .365	.504/ .100
	N					85/466	90/462	89/436

Reporting checklist for cross sectional study.

Based on the STROBE cross sectional guidelines.

Instructions to authors

Complete this checklist by entering the page numbers from your manuscript where readers will find each of the items listed below.

Your article may not currently address all the items on the checklist. Please modify your text to include the missing information. If you are certain that an item does not apply, please write "n/a" and provide a short explanation.

Upload your completed checklist as an extra file when you submit to a journal.

In your methods section, say that you used the STROBE cross sectionalreporting guidelines, and cite them as:

von Elm E, Altman DG, Egger M, Pocock SJ, Gotzsche PC, Vandenbroucke JP. The Strengthening the Reporting of Observational Studies in Epidemiology (STROBE) Statement: guidelines for reporting observational studies.

		Reporting Item	Page Number
**Title and abstract**			
Title	#1a	Indicate the study's design with a commonly used term in the title or the abstract	1
Abstract	#1b	Provide in the abstract an informative and balanced summary of what was done and what was found	1
**Introduction**			
Background / rationale	#2	Explain the scientific background and rationale for the investigation being reported	3
Objectives	#3	State specific objectives, including any prespecified hypotheses	3
**Methods**			
Study design	#4	Present key elements of study design early in the	4

For peer review only

		paper	
Setting	#5	Describe the setting, locations, and relevant dates, including periods of recruitment, exposure, follow-up, and data collection	4
Eligibility criteria	#6a	Give the eligibility criteria, and the sources and methods of selection of participants.	4
	#7	Clearly define all outcomes, exposures, predictors, potential confounders, and effect modifiers. Give diagnostic criteria, if applicable	6
Data sources / measurement	#8	For each variable of interest give sources of data and details of methods of assessment (measurement). Describe comparability of assessment methods if there is more than one group. Give information separately for for exposed and unexposed groups if applicable.	6
Bias	#9	Describe any efforts to address potential sources of bias	7
Study size	#10	Explain how the study size was arrived at	7
Quantitative variables	#11	Explain how quantitative variables were handled in the analyses. If applicable, describe which groupings were chosen, and why	7
Statistical methods	#12a	Describe all statistical methods, including those used to control for confounding	7
Statistical methods	#12b	Describe any methods used to examine subgroups and interactions	7
Statistical methods	#12c	Explain how missing data were addressed	7
Statistical methods	#12d	If applicable, describe analytical methods taking account of sampling strategy	7
Statistical methods	#12e	Describe any sensitivity analyses	7
**Results**			
Participants	#13a	Report numbers of individuals at each stage of study—	7

For peer review only

		eg numbers potentially eligible, examined for eligibility, confirmed eligible, included in the study, completing follow-up, and analysed. Give information separately for for exposed and unexposed groups if applicable.	
Participants	#13b	Give reasons for non-participation at each stage	7
Participants	#13c	Consider use of a flow diagram	n/a No drop-out
Descriptive data	#14a	Give characteristics of study participants (eg demographic, clinical, social) and information on exposures and potential confounders. Give information separately for exposed and unexposed groups if applicable.	7
Descriptive data	#14b	Indicate number of participants with missing data for each variable of interest	7
Outcome data	#15	Report numbers of outcome events or summary measures. Give information separately for exposed and unexposed groups if applicable.	7
Main results	#16a	Give unadjusted estimates and, if applicable, confounder-adjusted estimates and their precision (eg, 95% confidence interval). Make clear which confounders were adjusted for and why they were included	8
Main results	#16b	Report category boundaries when continuous variables were categorized	n/a Continuous variables not measured
Main results	#16c	If relevant, consider translating estimates of relative risk into absolute risk for a meaningful time period	n/a No measurement of risk
Other analyses	#17	Report other analyses done—e.g., analyses of subgroups and interactions, and sensitivity analyses	10
**Discussion**			
Key results	#18	Summarise key results with reference to study objectives	12
Limitations	#19	Discuss limitations of the study, taking into account	12

For peer review only

		sources of potential bias or imprecision. Discuss both direction and magnitude of any potential bias.	
Interpretation	#20	Give a cautious overall interpretation considering objectives, limitations, multiplicity of analyses, results from similar studies, and other relevant evidence.	12
Generalisability	#21	Discuss the generalisability (external validity) of the study results	13
**Other Information**			
Funding	#22	Give the source of funding and the role of the funders for the present study and, if applicable, for the original study on which the present article is based	1

Notes:

- 13c: n/a No drop-out
- 16b: n/a Continuous variables not measured
- 16c: n/a No measurement of risk The STROBE checklist is distributed under the terms of the Creative Commons Attribution License CC-BY. This checklist was completed on 08. June 2021 using https://www.goodreports.org/, a tool made by the EQUATOR Network in collaboration with Penelope.ai

For peer review only

BMJ Open

Validating a previously untested 'Intentions and Beliefs around Smoking' sub-scale for inclusion in the published 'Attitudes and Beliefs about Cardiovascular Disease (ABCD) Risk Questionnaire' using a cross-sectional sample

Journal:	*BMJ Open*
Manuscript ID	bmjopen-2021-054532.R1
Article Type:	Original research
Date Submitted by the Author:	04-Jan-2022
Complete List of Authors:	Bowyer, Mark; Nottingham Trent University, Institute of Health and Allied Professions, School of Social Sciences<br>Hassen, Hamid; University of Antwerp, Family Medicine and Population Health, Faculty of Medicine and Health Services<br>Bastiaens, Hilde; University of Antwerp Faculty of Medicine and Health Sciences, Family Medicine and Population Health<br>Gibson, Linda ; Nottingham Trent University, Institute of Health and Allied Professions, School of Social Sciences
**Primary Subject Heading**:	Public health
Secondary Subject Heading:	Cardiovascular medicine, Smoking and tobacco
Keywords:	PUBLIC HEALTH, STATISTICS & RESEARCH METHODS, PREVENTIVE MEDICINE

SCHOLARONE™
Manuscripts

I, the Submitting Author has the right to grant and does grant on behalf of all authors of the Work (as defined in the below author licence), an exclusive licence and/or a non-exclusive licence for contributions from authors who are: i) UK Crown employees; ii) where BMJ has agreed a CC-BY licence shall apply, and/or iii) in accordance with the terms applicable for US Federal Government officers or employees acting as part of their official duties; on a worldwide, perpetual, irrevocable, royalty-free basis to BMJ Publishing Group Ltd ("BMJ") its licensees and where the relevant Journal is co-owned by BMJ to the co-owners of the Journal, to publish the Work in this journal and any other BMJ products and to exploit all rights, as set out in our licence.

The Submitting Author accepts and understands that any supply made under these terms is made by BMJ to the Submitting Author unless you are acting as an employee on behalf of your employer or a postgraduate student of an affiliated institution which is paying any applicable article publishing charge ("APC") for Open Access articles. Where the Submitting Author wishes to make the Work available on an Open Access basis (and intends to pay the relevant APC), the terms of reuse of such Open Access shall be governed by a Creative Commons licence – details of these licences and which Creative Commons licence will apply to this Work are set out in our licence referred to above.

Other than as permitted in any relevant BMJ Author's Self Archiving Policies, I confirm this Work has not been accepted for publication elsewhere, is not being considered for publication elsewhere and does not duplicate material already published. I confirm all authors consent to publication of this Work and authorise the granting of this licence.

For peer review only

TITLE PAGE

Validating a previously untested 'Intentions and Beliefs around Smoking' sub-scale for inclusion in the published 'Attitudes and Beliefs about Cardiovascular Disease (ABCD) Risk Questionnaire' using a cross-sectional sample

Mark Bowyer, Nottingham Trent University (Corresponding Author)

mark.bowyer@ntu.ac.uk **(ORCID** 0000-0002-1474-5711)

Nottingham Trent University School of Social Sciences

Chaucer Building, Burton Street

Nottingham NG1 4BT

Tel: (+1) 7786 993405 Fax: (+1)115 8485574

Hamid Yimam Hassen, University of Antwerp, Belgium

hamid.hassen@uantwerpen.be **(ORCID** 0000-0001-6485-4193)

Dr Hilde Bastiaens (Participating Investigator)
Associate professor
Dept Family Medicine and Population Health
Faculty of Medicine and Health Sciences
University of Antwerp

Tel: 0032 (0)3 265.29.10 Fax: 0032 (0)3 265.25.26 Hilde.bastiaens@uantwerpen.be

Dr Linda Gibson (Participating Investigator)

Professor in Public Health, Institute of Health & Allied Professions

Nottingham Trent University Linda.gibson@ntu.ac.uk

Key words

Cardiovascular Diseases

- Cardiovascular risk factors
- Instrumentation

Psychometrics

- Surveys and questionnaires
- Instrumentation

Primary prevention

- Instrumentation

Word count 4002

For peer review only

Validating a previously untested 'Intentions and Beliefs around Smoking' sub-scale for inclusion in the published 'Attitudes and Beliefs about Cardiovascular Disease (ABCD) Risk Questionnaire' using a cross-sectional sample

ABSTRACT

Objectives:

To provide evidence of validity, reliability and generalisability of results obtained using the Attitudes and Beliefs about Cardiovascular Disease (ABCD) Risk Questionnaire with a sample of the English population surveyed within the 'SPICES' Horizon 2020 project (Nottingham study site), and to specifically evaluate the psychometric and factor properties of an as-yet untested 5 item sub-scale relating to smoking behaviours.

Design and setting:

Community based cross-sectional study in Nottingham, UK.

Participants:

466 English adults fitting inclusion criteria (aged 18+, without known history of CVD, not pregnant, able to provide informed consent) participated in the study.

Methods:

We re-validated the ABCD questionnaire on a sample of the general population in Nottingham to confirm the psychometric properties. Furthermore, we introduced 5 items related to smoking which were dropped in the original study due to inadequate valid samples.

Primary and secondary outcome measures:

1. Psychometric and factor performance of untested 5 item 'smoking behaviours' sub-scale
2. Psychometric and factorial properties in combination with the remaining 18 items across 3 sub-scales

Results:

Analyses of the data largely confirmed the validity, reliability, and factor structure of the original ABCD Risk Questionnaire. Sufficient participants in our study provided data against an additional five smoking related items to confirm their validity as a sub-scale and to advocate for their inclusion in future applications of the scale. EFA and CFA calculations support some minor changes to the remaining sub-scales which may further improve psychometric performance and therefore generalisability of the instrument.

Conclusions:

An amended version of the ABCD Risk Questionnaire would provide public health researchers and practitioners with a brief, easy to use, reliable and valid survey tool. The amended tool may assist public health practitioners and researchers to survey patient or public intentions and beliefs around three key areas of individually modifiable risk (Physical Activity, Diet, Smoking).

For peer review only - http://bmjopen.bmj.com/site/about/guidelines.xhtml

Trial registration:

ISRCTN68334579 https://doi.org/10.1186/ISRCTN68334579
Heart health without a doctor: an implementation study of CVD prevention and behaviour change interventions in community settings

Ethical approval

Ethical approval for the 'SPICES' Nottingham study protocol (incorporating the ABCD Risk Questionnaire) was secured from the Nottingham Trent University College of Business, Law and Social Sciences on the 20th February 2019. Participants were required to provide informed consent (Appendix 1).

Article summary

Strengths and Limitations of this study

- Large sample (n=466) of English adults from the Nottingham UK population
- Sufficient case data to validate additional sub-scale related to attitudes and intentions of smokers
- Criterion validity not explored
- Full assessment of the utility of ABCD Risk Questionnaire in health promotion and CVD prevention not explored; further studies may be required to position the tool in clinical and public health practice.
- The planned pre-post intervention measurement and analysis was not possible due to COVID-19 interruption of fieldwork.

Original protocol (Appendix 2)

Funding statement

This work was supported by the European Commission Horizon 2020 Non-communicable diseases and the challenge of healthy ageing Grant agreement 733356 'SPICES'.

Competing interests statement

None declared

Patient and public involvement

Patients and/or the public were not involved in the design, or conduct, or reporting, or dissemination plans of this research.

Patient consent for publication (data sharing agreement)

Not required (participant information and informed consent attached Appendix 1)

Provenance and peer review

Not commissioned.

Data availability statement

Data are available on reasonable request

For peer review only - http://bmjopen.bmj.com/site/about/guidelines.xhtml

Keywords

Cardiovascular diseases- Cardiovascular risk factors

Cardiovascular diseases- Instrumentation

Psychometrics- Instrumentation

Surveys and questionnaires- Instrumentation

Primary prevention- Instrumentation

Author contributions

Following ICMJE recommendations, Mark Bowyer and Hamid Hassen assert authorship based on the following 4 criteria:

Substantial contributions to the conception or design of the work; or the acquisition, analysis, or interpretation of data for the work; AND

Drafting the work or revising it critically for important intellectual content; AND

Final approval of the version to be published; AND

Agreement to be accountable for all aspects of the work in ensuring that questions related to the accuracy or integrity of any part of the work are appropriately investigated and resolved.

Professor Linda Gibson and Professor Hilde Bastiaens assert Participating Investigator status having served as scientific advisors, critically reviewed the study proposal, and participated in writing or technical editing of the manuscript.

Acknowledgements

The authors would like to acknowledge the cooperation of Rolls-Royce plc Hucknall Site; Nottingham City Council Adult Care in providing access to employees. Crabtree Farm Community Centre, Middle Street Resource Centre, Self-Help UK, in facilitating access to members, users and premises.

INTRODUCTION

Scientific Background and Rationale

In the UK, Cardiovascular Disease (CVD) is responsible for over 130,000 deaths per annum.[1] CVD morbidity is also the biggest contributor to the inequalities in Healthy Life Expectancy between members of the wealthiest neighbourhoods and the most deprived.[2] In 2009 the NHS Health Check [3]was established and more recently (2019) the CVD Prevent initiative to implement ‘upstream’ interventions for the prevention of CVD morbidity.[4] Both of these initiatives seek to

For peer review only - http://bmjopen.bmj.com/site/about/guidelines.xhtml

For peer review only

improve early case-finding to prevent avoidable strokes and heart attacks. Both recognise the importance of supported lifestyle change in conjunction with drug therapies.

Lifestyle or behavioural change requires a degree of individual agency and commitment which drug therapies do not. Unhealthy lifestyle behaviours are linked to culture and habit, environment, emotions, and confidence which can all moderate an individual's readiness to change and the commitment required to sustain those changes over time.[5] Understanding the attitudes and beliefs that people hold towards diet, exercise and smoking, as well as their perception of their own risk could assist primary care and public health professionals in providing relevant and effective behavioural advice and social prescribing options. To support evaluations of the NHS Health Check programme, in 2017 a questionnaire was developed to evaluate patients' awareness of cardiovascular disease risk at University College London.[6] This ABCD Risk Questionnaire attempts to provide a short survey drawing from the dominant theoretical models of behaviour change (Trans-Theoretical Model, Health Beliefs Model),[7] covering diet, smoking, exercise and alcohol behaviours, and incorporating a conceptual spread of perceived risk from immediate to lifetime.

Specific Objectives

In this study we re-validated the tool on a sample of the general population in Nottingham to confirm the psychometric properties. Furthermore, we introduced 5 items related to smoking which were dropped in the original study due to inadequate case numbers.

To the best of our knowledge, this is the first study which has incorporated items relating to attitudes and intentions towards stopping smoking into the published version of the ABCD Risk Questionnaire and collected sufficient data to submit them to analysis of validity, reliability and factor structure.

In the original ABCD study, over the course of three stages of validity testing (content, face, reliability) items relating to alcohol use and smoking were rejected, leaving four final sub-scales: Knowledge of CVD Risks; Perceived Risk of Heart Attack/ Stroke; Perceived Benefits and Intentions to Change; and Healthy Eating Intentions. During Exploratory Factor Analysis (EFA) none of the items relating to alcohol use achieved strong enough loadings to be included in the final scale, and items related to smoking could not be included due to the high proportion of missing data in the experimental sample. The authors of the study note this limitation '*the questionnaire does not encompass all aspects of CVD risk observed in the general population*' and that '*future studies examining populations at increased CVD risk can look into incorporating smoking and alcohol into the ABCD Risk Questionnaire to learn about these individuals' preconceptions and attendance of follow-up care*'.[8]

The present study

Nottingham is one of five global sites of the EU Horizon 2020 'SPICES' [9] CVD prevention implementation study which began in 2017. SPICES investigates contextual and health system barriers to the scaling up of successful behaviour change interventions for improved cardiovascular health in low, middle and high income European countries. The most recent data (2016) indicate that "The prevalence of CVD recorded in Nottingham City GP Practices is significantly less that the national (England) average and in comparable areas, despite the CVD mortality rate being significantly higher than average; this partly reflects the differing age structures of the populations, but also indicates significant under-detection/diagnosis"[9]

For peer review only - http://bmjopen.bmj.com/site/about/guidelines.xhtml

The SPICES Nottingham population survey carried out in 2019-20 utilised the ABCD Risk Questionnaire alongside the non-clinical INTERHEART CVD risk prediction instrument.[10] The SPICES study team chose to re-introduce 5 pre-written items relating to 'Intentions and Readiness to Stop Smoking' from the 65 item University College London (UCL) item pool into the questionnaire due to the high prevalence of smoking in the Nottingham population compared to England averages,[11] and its importance as a CVD risk.[12] This created a 31 item questionnaire. 4 items relating to Alcohol intake from the same item pool were also considered for inclusion but ommitted on two grounds: alcohol related CVD risk was not a specific focus of the 'SPICES' study; concerns about the time-burden on participants of including the additional items which can be a barrier to participation.

In so doing, NTU researchers attempted to '*replicate the factor analytic process on an independent, larger sample to confirm the generalisability of (the original) findings*' as requested by the authors of the original study.[13] At the same time, we anticipated securing sufficient responses against the reintroduced 5 item 'smoking' sub-scale to analyse its reliability and validity as an integral part of future versions of the Questionnaire.

METHODS

Incorporating the ABCD Risk Questionnaire into the SPICES Nottingham baseline survey provided cross-sectional study data across a broad sample of adult participants. The data-set generated was therefore suitable for psychometric validation of the original and modified versions of the ABCD questionnaire. Surveys were administered in-person by researchers in the field during attendance at community venues and workplaces. Administration of the survey took approximately ten minutes including provision of consent, and confidential communication of results another ten minutes on average. Participation was entirely voluntary.

Participants

Participants were recruited from across the Nottingham conurbation between April 2019 and March 2020 as part of the SPICES Nottingham baseline survey.[14] A purposive sampling method was employed based on community engagement. This strategy had two components:

1. engagement of citizens in neighbourhoods through existing community groups, organisations and venues, and
2. engagement of employees in the workplace through large city-based employers.

Community groups were targeted on the basis of the demographic of their membership to ensure that neighbourhoods of differing mean household income, those who are not in employment or of working age, and those from different ethnicities were included. In this way 327 participants were recruited.

Employers were targeted on the basis of workforce size, and policies relating to workforce well-being. Nottingham City Council Adult Care teams and the Rolls-Royce plc Hucknall site both responded positively and between them provided 156 participants. NTU researchers administered the SPICES Nottingham baseline survey individually within the community or workplace setting and personalised feedback about CVD risks was provided confidentially once the survey had been completed.

For peer review only - http://bmjopen.bmj.com/site/about/guidelines.xhtml

Criteria for inclusion included being aged 18+, resident in Nottinghamshire, not previously diagnosed with a heart condition, not pregnant, and able to provide informed consent.

Materials

The SPICES baseline survey incorporated the ABCD risk questionnaire into a digitised survey instrument created in the Research Electronic Data Capture (REDCap) database system,[15] a secure web application for building and managing online surveys and databases, and the online survey responses were uploaded automatically. No participant data was stored on local devices. Both the ABCD Risk Questionnaire (Table 1) and the non-laboratory INTERHEART questionnaire were included unchanged from their published versions apart from an additional 5 items pertaining to smoking behaviour (Table 2).[16]

Table 1. Published ABCD Risk Questionnaire

Scale	Items
**Knowledge**<br><br>True/False/Don't Know<br><br>Correct score =1<br>Incorrect/ Don't know score = 0<br><br>Higher sum score= more knowledgeable/ more correct about having a heart attack or stroke	1. One of the main causes of heart attack and stroke is stress
	2. Walking and gardening are considered types of exercise that can lower the risk of having a heart attack or stroke
	3. Moderately intense activity of 2.5 hours a week will reduce your chances of having a heart attack or stroke
	4. People who have diabetes are at higher risk of heart attack or stroke
	5. Managing your stress levels will help you to manage your blood pressure
	6. Drinking high levels of alcohol can increase your cholesterol and triglyceride levels
	7. HDL refers to 'good' cholesterol, and LDL refers to 'bad' cholesterol
	8. A family history of heart disease is not a risk factor for high blood pressure
**Perceived Risk of Heart Attack or Stroke**<br><br>4= Strongly disagree, 3= Disagree, 2= Agree, 1= Strongly Agree; N/A= 0<br><br>Higher sum score = higher perception of risk of having a heart attack or stroke	9. I feel I will suffer from a heart attack or stroke sometime during my life
	10. It is likely that I will suffer from a heart attack or stroke in the future
	11. It is likely that I will have a heart attack or stroke some time during my life
	12. There is a good chance I will experience a heart attack or stroke in the next 10 years
	13. My chances of suffering from a heart attack or stroke in the next 10 years are great
	14. It is likely I will have a heart attack or stroke because of my past and/or present behaviours
	15. I am not worried that I might have a heart attack or stroke (**Reverse coded**)
	16. I am concerned about the likelihood of having a heart attack or stroke in the near future
**Perceived Benefits and**	17. I am thinking about exercising at least 2.5 hours a week
	18. I intend or want to exercise at least 2.5 hours a week

For peer review only

**Intentions to Change**<br><br>4= Strongly disagree, 3= Disagree, 2= Agree, 1= Strongly Agree; N/A= 0<br><br>Higher average score = Higher perceived benefits of diet and exercise and higher perceived readiness for change in regards to exercise and behaviour	19. When I exercise for at least 2.5 hours a week I am doing something good for the health of my heart
	20. I am confident that I can maintain a healthy weight by exercising at least 2.5 hours a week
	21. I am not thinking about exercising for 2.5 hours a week **(Reverse coded)**
	22. When I eat five portions of fruit and vegetables a day I am doing something good for the health of my heart
	23. Increasing my exercise to at least 2.5 hours a week will decrease my chances of having a heart attack or stroke
**Healthy Eating Intentions**<br><br>4= Strongly disagree, 3= Disagree, 2= Agree, 1= Strongly Agree; N/A= 0<br><br>Higher average score = Higher perceived readiness for change with regard to healthy dietary behaviour	24. I am confident that I can eat at least five portions of fruit and vegetables a day within the next two months
	25. I am thinking about eating at least five portions of fruit and vegetables a day
	26. I am not thinking about eating at least five portions of fruit and vegetables a day **(Reverse coded)**

The surveys were administered in the field by a team of trained researchers recruited from the NTU student body and directly supervised by the SPICES Nottingham coordinator. The surveys were accessed using dedicated tablet computers. Items were reproduced word for word and in the same sequence as the original ABCD Risk Questionnaire with the additional 5 smoking items inserted after all 26 original items. These five smoking sub-scale items were drawn from the 65 item pool developed in the original study but omitted from analysis due to a high proportion of missing responses.

Table 2. Additional 'smoking' sub-scale

**Benefits and Intentions to Stop Smoking**<br><br>4= Strongly disagree, 3= Disagree, 2= Agree, 1= Strongly Agree; N/A= 0<br><br>Higher average score = Higher perceived readiness for change with regard to healthy dietary behaviour	27. I am thinking of stopping smoking within two months
	28. I have reduced or stopped smoking
	29. I intend or want to stop smoking
	30. If I stop smoking it will reduce my chances of having a heart attack or stroke
	31. I am not thinking about stopping smoking

Validating the sample

For peer review only - http://bmjopen.bmj.com/site/about/guidelines.xhtml

For peer review only

The baseline survey dataset was extracted from REDCap for analysis. Sample was checked for representativeness of the Nottingham population across parameters of age, gender, household income and known rates of physical activity and smoking.

Data analysis

We took the published 26-item ABCD Risk Questionnaire, introduced 5 further items relating to smoking behaviours, and administered it alongside a validated CVD risk assessment instrument (INTERHEART) to 486 individuals in Nottingham over a period of 12 months. Item, scale, and factor reliabilities were remeasured. Correlation was tested between and amongst ABCD sub-scale scores and selected INTERHEART variables, closely matching the methods applied in the original study (Appendix 3) and results were compared accordingly. After removing incomplete responses, 466 valid cases were entered for analysis, four times the sample size of the original study.

Item and sub-scale reliabilities were tested using inter-item correlations, corrected item-total correlations and Cronbach's Alpha. [17] We performed an exploratory factor analysis (EFA) to evaluate the dimensionality of items of the original and modified risk scale with and without the smoking items.[18] The EFA was performed using the maximum likelihood extraction and varimax rotation method. [19] Sample and data adequacy was assessed using Kaiser-Meyer-Olkin (KMO) test and Bartlett's test of sphericity was performed to compare an observed correlation matrix to the identity matrix.[20] The adequate number of factors was determined using a scree plot. To further test the consistency of factors, we tested using Confirmatory Factor Analysis (CFA). We evaluated the model fit of the CFA using; the X2 test, the Tucker-Lewis and Comparative Fit Indexes and the root mean square error of approximation (RMSEA).[21] The analysis was performed using a free statistical software R version 4.0.2. UK postcodes were collected for all participants which allowed them to be sorted into income deciles using Office for National Statistics Index of Multiple Deprivation (IMD) public datasets, allowing correlations to be analysed. Case data from the 'Knowledge' sub-scale (8 items) were omitted from the analysis since they utilise a separate response format.

We used the STROBE cross sectional checklist when writing our report.[22]

RESULTS

Participants

Participation was voluntary, and self-selection may have been influenced by sensitivities around disclosure of health status and lifestyle habits forming a barrier to those with co-morbidities and socially 'questionable' behaviours (heavy smoking, high alcohol intake).

The sample cohort is strongly parametric, with a 49:51 percent gender split, normal distribution of age ranges (18-92), and a distribution of Socio-Economic Status (SES) which reflects known data about neighbourhood income in Nottingham. Nottingham is the 11th most deprived district in England with higher unemployment, lower education and skills, and shorter life expectancy than the national averages.[23]Using the Index of Multiple Deprivation a relative measure of deprivation across seven domains, Health and Disability is the domain on which the city does worst. Nevertheless, the mean INTERHEART predicted risk score for all 466 participants was 10.32 which closely matches the global reported mean for the instrument.[24]

Smoking sub-scale

For peer review only - http://bmjopen.bmj.com/site/about/guidelines.xhtml

The percentage of smokers in our sample was 15.5%. The number of smokers in our sample was therefore lower than the England average (18%), and lower than the Nottingham city population average (20.6%) based on the ONS Annual Population Survey.[25] ONS notes that smoking prevalence estimates by local authority can fluctuate due to smaller sample sizes. Our SPICES Nottingham sample cohort also includes some participants from neighbouring Local Authorities with different recorded rates of smoking.

The five items in the smoking subscale are measured on the same four-point response scale as the 18 items submitted for Factor Analysis in the original published ABCD Risk Questionnaire (Strongly agree, agree, disagree, strongly disagree, and not applicable).

With the original 18 items this 'Not Applicable' response option was not used by any of the SPICES Nottingham study participants. By contrast, within their responses to the items in the 'smoking' subscale, 'Not Applicable' was the modal answer. Participants chose the 'N/A' response option whenever they reported being a non-smoker. This mirrors the behaviour of the original 110 NHS Health Check attendees who formed the pilot sample cohort for the original study, leaving an insufficient number of cases to assess validity and reliability of smoking sub-scale items. In the present study, 88 cases were found where participants reported smoking behaviours and this was sufficient to enter them into analysis.

Sub-scale Alpha values, Cronbach's Alpha if item deleted calculated for all items, inter-item correlations and corrected item-total correlations were all calculated, mirroring the analysis reported in the original study (Appendix 4).

Interitem correlations calculated for these five items produced a range between 0.654 and 0.834. All of these five 'smoking' items therefore correlate with one another more strongly than recommended (<.6) and were considered for rejection (Appendix 4). However, we found each item to be qualitatively different, and that the differences were conceptually clear and well expressed in the item wording so that no participant could be expected to confuse one with any other, and they were retained.

Discrimination was confirmed using item-total correlations. These fell between the range 0.751 and 0.906 meaning that all five 'smoking' sub-scale items are comfortably above the standard cut-off for acceptability of 0.3.

EFA was carried out twice, firstly with all cases, and then again with 88 confirmed smoking cases. The first operation ensured that factor loadings were not skewed by the lower number of cases reporting smoking behaviours, the second ensured that factor loadings for the remaining sub-scales where more case data was available were not skewed by outliers.

Exploratory Factor Analysis:

We conducted EFA on the original 18-item risk perception questionnaire and the modified 23-item (with smoking items). For the original 18-item, a total of 420 observations were included in the analysis, which was sufficient for factor analysis as indicated with KMO of 0.82, which is within the recommended range (0.8 to 1). The Bartlett's Test of Sphericity was significant (X2 = 4235.007, p-value < 0.001) indicating the data is adequate for factor analysis. As a result, a three-factor solution emerged based on the Scree plot (figure 1), accounting 57.4% of the total variance. Factor loading patterns in the present analysis slightly varied from the original subscales. The domains in the original subscales were risk perception, benefit finding and healthy eating intentions. In our analysis, Item 14 (*'When I eat at least 5 portions of fruit and vegetables a day I am doing something good for the health of my*

For peer review only - http://bmjopen.bmj.com/site/about/guidelines.xhtml

heart') showed a better loading to healthy eating intention, which was loaded to benefit finding in the original study (Appendix 5).

For the modified 23-item (including the smoking sub-scale), 88 samples were valid and included in the analysis. The KMO was 0.78, which was slightly below the recommended range, but Bartlett's Test of Sphericity was significant (X2 = 1223.459, p-value < 0.001), indicating adequacy for factor analysis. The analysis showed that the smoking items loaded to another latent construct resulting in four factors in total (figure 2).

Confirmatory Factor Analysis of the published ABCD Risk Questionnaire

A Confirmatory Factor Analysis was undertaken using the SPICES Nottingham dataset to investigate further. Conducting CFA allowed us to construct the sub-scales of the published ABCD Risk Questionnaire in a three-factor measurement model and test its fit against relevant indices. Original 18 item survey comprising three sub-scales (Perceived Risk of Heart Attack/Stroke 8 items; Perceived Benefits and Intentions to Change 7 items; Healthy Eating Intentions 3 items) were used to create measurement model in SPSS Amos 25. The model was then updated to include an additional 5 item sub-scale relating to smoking behaviours.

Editing the measurement model

The CFA measurement model was then reconstructed removing items which had confused participants and generated high inter-item correlations, and additionally re-assigning an item relating to dietary behaviour into the dietary behaviour sub-scale. This resulted in a four-factor model (Perceived Risk of Heart Attack/ Stroke' 6 items; 'Perceived Benefits and Intentions to Exercise' 6 items; 'Healthy Eating Intentions' 4 items, Perceived Benefits and Intentions to Reduce Smoking' 5 items).

Analysis properties were set to Estimation: Maximum Likelihood.

Table 3. CFA fit indices for the original and modified ABCD Questionnaire measurement models

**Original 18 item ABCD[1]**						
CMIN	P	CMIN/DF	TLI	CFI	RMSEA	RMR
714.941	.000	5.416	.826	.850	.097	.049
**Original 18 item ABCD with 5 Smoking items added[2]**						
CMIN	P	CMIN/DF	TLI	CFI	RMSEA	RMR

[1] In the original study of 2017, 18 items were entered into factor analysis. This Confirmatory Factor Analysis tests the fit of these original items to their structure using the larger Nottingham SPICES dataset.

[2] In th[illegible]uded in the [illegible]e made to test these smoking items.

For peer review only - http://bmjopen.bmj.com/site/about/guidelines.xhtml

994.931	.000	4.442	.865	.881	.086	.049
**Edited 20 item ABCD with Smoking sub-scale[3]**						
CMIN	P	CMIN/DF	TLI	CFI	RMSEA	RMR
638.973	.000	3.896	.881	.897	.079	.052
**Modified 20 item ABCD with Smoking sub-scale[4]**						
CMIN	P	CMIN/DF	TLI	CFI	RMSEA	RMR
385.312	.000	2.439	.941	.951	.056	.046

Similarly, in the 23-item factor analysis, item 14 was loaded to the healthy eating intention. The model fit indices showed a slight improvement as indicated in table 3.

Based on factor loading, inter-item correlations, and face validity results, we also tested a slightly shorter version of the questionnaire, 20-items including five smoking items and the result shows that the model fit improved (CFI=0.941; TLI=0.951; RMSEA=0.056, SRMR=0.046).

The three published factors achieved a poor fit in CFA (Table 3). Including the five smoking related items which had performed strongly in EFA as their own latent factor improved overall model fit slightly, but not to an acceptable level.

Modification of the measurement model

Reviewing modification indices and expected parameter changes for factor loadings and measurement intercepts we observed an extreme covariance value (116.812) and parameter change (.209) between two of the risk perception items ('there is a good chance that I will experience a heart attack or stroke in the next 10 years' and 'my chances of suffering a heart attack or stroke in the next 10 years are great') which had caused confusion for participants in our study.

Removing one of these two items (item #13), and the two other duplicative items (items #9 & #10) from the 'perceived risk of heart attack or stroke' sub-scale retains the conceptual spread of risk embodied by the items (lifetime, 10 year, near future, behaviour related). Moving the diet related item (#22) which appears in the 'perceived benefits and intentions to change' over to the 'healthy eating intentions' sub-scale might allow greater clarity for researchers analysing results from the questionnaire. Co-varying items within sub-scales that generated values above 20 (a high cut-off due to large sample used) resulted in acceptable or good fit across all sub-scales. Each of the three behaviour related sub-scales now contain items drawn from HBM, TTM and SE models providing a sound conceptual basis for comparison. Using EFA to check these results shows the modified sub-scale structure performs better than the published version (all EFA results Appendix 5).

For peer review only

[3] As discussed above, independent item analysis and Exploratory factor Analysis using the independent SPICES Nottingham dataset revealed issues with the continued inclusion of some of the original 'perception of risk' sub-scale items, and the allocation of an item relating to dietary behaviours in the physical activity behaviours sub-scale. The published ABCD questionnaire was edited to remove or re-assign the problematic items and retested using Confirmatory Factor Analysis.

[4] The measurement model created for the Confirmatory Factor Analysis was modified so that items within each ABCD sub-scale were set to co-vary with one another.

DISCUSSION

Inadequate knowledge and/or a gap between perceived and actual CVD risk in the population could be an obstacle to better health outcomes. Improving an individual's CVD knowledge and risk perception may be important in improving a healthy lifestyle. Measuring CVD knowledge and risk perception may be a method to initiate a healthy lifestyle intervention as well as to monitor and evaluate the impact of interventions. Following this rationale, Woringer and colleagues developed the ABCD Risk questionnaire in order to measure CVD knowledge and risk perception. In this study, we re-validated the tool on a sample of the general population in Nottingham to confirm the psychometric properties.

The 88 participants in this study who reported smoking is a low number for pilot testing of psychometric scales but it does exceed a 10:1 ratio of cases to variables making it reasonable to proceed to analysis.

Based on EFA and CFA, we confirmed a three-factor structure, which closely matched the results reported in the original study, but differed in certain important respects. Item 14 ('*When I eat at least 5 portions of fruit and vegetables a day I am doing something good for the health of my heart*") showed a better loading to the 'healthy eating intentions' sub-scale, in contrast to the factor loading in the original study, which placed this item in 'perceived benefits and intentions to change'. This is the only item which loaded onto a different sub-scale when using the Nottingham dataset, all others continued to load onto their original factors although many of these loaded weakly and failed to meet usual thresholds for validity (Appendix 5). The larger numbers of participants in our dataset (466 compared to 110) provides statistical confidence in the new results, and we therefore modelled this revised allocation of items and factors alongside the original factor allocations in the subsequent Confirmatory Factor Analysis. The revised measurement model with item 14 allocated to 'Healthy Eating Intentions' indicated a better fit in CFA results.

These results suggest that the additional five smoking items perform acceptably and should be incorporated into future applications of the ABCD Risk Questionnaire.

We believe that psychometric performance based on reliability calculations and factorial analysis is not an end in itself. The resulting scale has to have some utility in the world and generate results which can add value to existing understanding of beliefs and attitudes to cardiovascular disease. This is only very lightly touched on in the original paper which states that 'the questionnaire can be used to assess patients' understanding of CVD risk'. We believe that because there is a recognised gap between 'knowing' and 'doing' in relation to CVD risk factors which means that much health education may be failing to stimulate the healthy changes in the population, it is important to consider the attitudes and beliefs about elective change in relation to risky lifestyle behaviours which may be mediating this relationship. If it is not enough simply to educate vulnerable people to the nature of the risks in order to stimulate the necessary changes to reduce CVD risk, then although socio-economic factors will also play a part here, and there may be additional psychological factors (such as 'present-bias') which also mediate this space, the ABCD Risk Questionnaire goes a long way to investigating and measuring the personal beliefs and attitudes which operate in this space.

Other observations

Researchers in the Nottingham SPICES team administering the questionnaire during fieldwork reported that three items within the 'Perception of Risk of Heart Attack/Stroke' sub-scale caused consistent difficulties for respondents due to apparent duplication and confusion over fine semantic differences. It was difficult for participants to see a semantic difference between statements 9, 10,

For peer review only - http://bmjopen.bmj.com/site/about/guidelines.xhtml

11, and 12, 13 respectively. For items 9, 10, and 11, if we agree that *suffer from* and *have* are synonymous, it is hard to differentiate between *in the future* and *some time during my life* because you would imagine that respondents will be thinking about the future in both cases.

For the questionnaire to be reliable across all sections of the population, including those with limited ability in English (whether native or non-native, first, second or additional language, etc.) who may find it particularly hard to differentiate with any confidence between different pairs/sets of statements with largely synonymous meanings, this confusion is a problem. Items 12 and 13 seem to differ mainly only in the possible interpretation of a difference of degree between *good* and *great.*

These face validity issues and their impact can be observed in the inter-item correlation results generated during item reliability analysis. In the original study, two items in the perception of risk sub-scale had been rejected due to correlations in excess of 0.6 leaving 8 items. Of these remaining 8 items half had inter-item correlations which exceeded 0.6 when tested against the Nottingham dataset. These were items 9, 10, 11, and 12 which generated inter-item correlation values of .832, .869, .616, and .729 respectively. Removing items 9, 10, and 13 does not reduce the conceptual range of the 'perception of risk' subscale which is framed temporally from immediate threat to lifetime risk, it simply removes the duplicate or confusing items. Testing this shortened scale with factor analysis strengthens both item and scale reliability and improves factor loadings (Appendix 5). We recommend that future versions of the English language ABCD Risk Questionnaire adopt these edits (Appendix 6).

CONCLUSIONS

The published English language version of the ABCD Risk Questionnaire, with the removal of three problematic 'perception' items, the shift of one item from the 'perceived benefits and intentions to change' sub-scale into the 'healthy eating intentions' sub-scale, and the addition of a 5 item 'smoking' sub-scale performs sufficiently well in validity, reliability and factor analysis with an independent, larger sample to confirm the generalisability of its original published findings. This result supports continued use of the ABCD Risk Questionnaire in the field of CVD prevention research and practice. The inclusion of a smoking behaviours sub-scale is likely to increase its relevance where smoking behaviours still account for a large proportion of individually modifiable CVD risk in a target population. Although criterion validity has now been established for the 'Perception of risk of heart attack/stroke sub-scale' by two published studies, the utility of the remaining sub-scales individually or in combination has been under-examined. Future studies should investigate the criterion validity of these sub-scales and the conceptual strength of the items and variables from which they have been composed in order to unambiguously position the resulting survey instrument and evaluate its utility in CVD prevention and treatment practices. Neither this study or the original published study of 2017 were able to conduct pre-post measurements in their study design. Measuring using the ABCD survey before an intervention (such as the NHS Health Check) and then again at some time afterwards- in tandem with a validated CVD risk prediction scale (such as INTERHEART or Q Risk 2) would help to establish the ABCD Risk Questionnaire's sensitivity to change, and perhaps also its ability to discern between types of respondent.

REFERENCES

1 https://www.bhf.org.uk/bhf-cvd-statistics-uk-factsheet.pdf (Accessed 10/05/2021)

For peer review only - http://bmjopen.bmj.com/site/about/guidelines.xhtml

2 https://www.england.nhs.uk/ourwork/clinical-policy/cvd/ (Accessed 10/05/2021)

3 Robson J, Dostal I, Sheikh A*, et al*
The NHS Health Check in England: an evaluation of the first 4 years
BMJ Open 2016;**6:**e008840. doi: 10.1136/bmjopen-2015-008840

4 Kearney M
Prevention and treatment of CVD: a new priority for the NHS
Heart 2019;**105:**1924.

5 Michie, S., van Stralen, M.M. & West, R. The behaviour change wheel: A new method for characterising and designing behaviour change interventions. *Implementation Sci* **6,** 42 (2011). https://doi.org/10.1186/1748-5908-6-42

6 Woringer M, Nielsen JJ, Zibarras L*, et al*
Development of a questionnaire to evaluate patients' awareness of cardiovascular disease risk in England's National Health Service Health Check preventive cardiovascular programme
BMJ Open 2017;**7:**e014413. doi: 10.1136/bmjopen-2016-014413

7 Health Behavior: Theory, Research, and Practice
edited by Karen Glanz, Barbara K. Rimer, K. Viswanath

8 Woringer M, Nielsen JJ, Zibarras L*, et al* IBID
https://www.nottinghaminsight.org.uk/themes/health-and-wellbeing/joint-strategic-needs-assessment/adults/cardiovascular-disease-2016/ (accessed 29/11/21)

9 Nahar, P., van Marwijk, H., Gibson, L. *et al.* A protocol paper: community engagement interventions for cardiovascular disease prevention in socially disadvantaged populations in the UK: an implementation research study. *glob health res policy* **5,** 12 (2020). https://doi.org/10.1186/s41256-020-0131-1

10 Yusuf S , Hawken S , Ounpuu S , *et al* . Effect of potentially modifiable risk factors associated with myocardial infarction in 52 countries (the INTERHEART study): case-control study. Lancet 2004;**364**:937–52.doi:10.1016/S0140-6736(04)17018-9

11 https://www.nottinghaminsight.org.uk/themes/health-and-wellbeing/joint-strategic-needs-assessment/adults/smoking-and-tobacco-control-2019/

12 John A Ambrose, Rajat S Barua,
The pathophysiology of cigarette smoking and cardiovascular disease: An update,
Journal of the American College of Cardiology, Volume 43, Issue 10, 2004, Pages 1731-1737, ISSN 0735-1097,
https://doi.org/10.1016/j.jacc.2003.12.047.
(https://www.sciencedirect.com/science/article/pii/S0735109704004346)

13 Woringer M, Nielsen JJ, Zibarras L*, et al* IBID

14 Nahar, P., van Marwijk, H., Gibson, L. *et al.* IBID

15 Harris, P.A., et al., Research electronic data capture (REDCap)—A metadata-driven methodology and workflow process for providing translational research informatics support. Journal of Biomedical Informatics, 2009. **42**(2): p. 377-381 DOI: https://doi.org/10.1016/j.jbi.2008.08.010.

16 McGorrian C, Yusuf S, Islam S, Jung H, Rangarajan S, Avezum A, et al. Estimating modifiable coronary heart disease risk in multiple regions of the world: the INTERHEART Modifiable Risk Score. European Heart Journal. 2011;32(5):581-9. doi: 10.1093/eurheartj/ehq448.

17 Cronbach, L.J. Coefficient alpha and the internal structure of tests. *Psychometrika* **16,** 297–334 (1951). https://doi.org/10.1007/BF02310555

18 Ferguson, E. and Cox, T. (1993), Exploratory Factor Analysis: A Users'Guide. International Journal of Selection and Assessment, 1: 84-94. https://doi.org/10.1111/j.1468-2389.1993.tb00092.x

19 Kaiser, H.F. The varimax criterion for analytic rotation in factor analysis. Psychometrika 23, 187–200 (1958). https://doi.org/10.1007/BF02289233

20 Bartlett, M. S. (1951). The effect of standardization on a Chi-square approximation in factor analysis. Biometrika, 38(3/4), 337-344

21 Hu Lt, Bentler PM. Cutoff criteria for fit indexes in covariance structure analysis: Conventional criteria versus new alternatives. Structural Equation Modeling: A Multidisciplinary Journal. 1999;6(1):1-55. doi: 10.1080/10705519909540118.

For peer review only

For peer review only - http://bmjopen.bmj.com/site/about/guidelines.xhtml

22 von Elm E, Altman DG, Egger M, Pocock SJ, Gotzsche PC, Vandenbroucke JP. The Strengthening the Reporting of Observational Studies in Epidemiology (STROBE) Statement: guidelines for reporting observational studies.
23 Ministry of Housing, Communities & Local Government. The English indices of deprivation 2019 statistical release https://www.gov.uk/government/statistics/english-indices-of-deprivation-2019 (accessed 10/05/2021)
24 Yusuf S , Hawken S , Ounpuu S , *et al* **IBID**
25 UK Government Office for National Statistics 2019 https://www.ons.gov.uk/peoplepopulationandcommunity/healthandsocialcare/healthandlifeexpectancies/bulletins/adultsomkinghabitsgreatbritain/2018 (Accessed 10/05/2021)

Figure legends

Figure 1. 18 item ABCD Questionnaire scree plot results from Nottingham dataset

Figure 2. Modified ABCD Questionnaire 20 items with smoking scree plot results Nottingham dataset

For peer review only

For peer review only - http://bmjopen.bmj.com/site/about/guidelines.xhtml

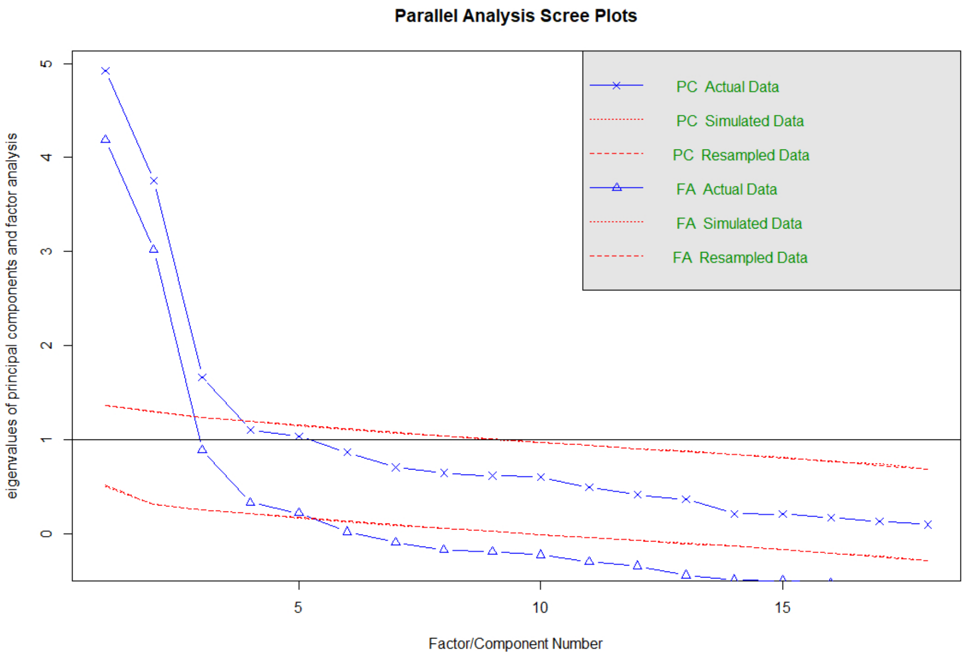

Figure 1. 18 item ABCD Questionnaire scree plot results from Nottingham dataset

286x198mm (96 x 96 DPI)

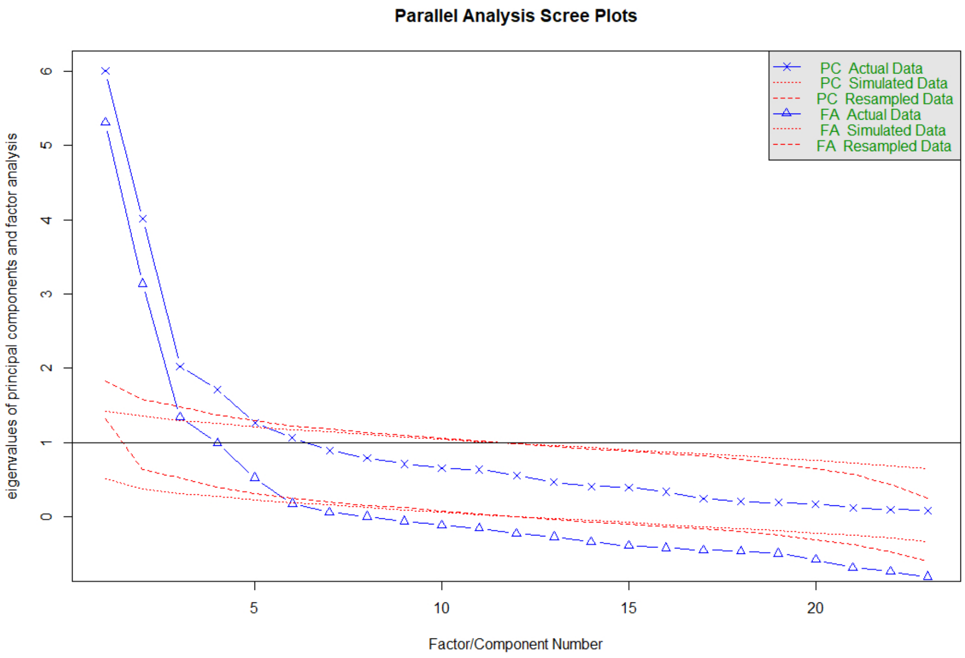

Figure 2. Modified ABCD Questionnaire 20 items with smoking scree plot results Nottingham dataset

286x198mm (96 x 96 DPI)

Horizon 2020
European Union funding
for Research & Innovation

NOTTINGHAM
TRENT UNIVERSITY

‘SPICES’ Heart Diseases Prevention Research

Introduction to SPICES research

Nottingham Trent University is part of an international research team investigating ways to build good practice in the prevention of Heart Diseases. Researchers and doctors have a lot of evidence about what causes heart diseases and what prevents them. Heart Diseases are now the biggest cause of death globally, and one of the leading causes of disability, so the more people know what the doctors know, the better they can protect themselves and maintain a good quality of life.

The research project is called 'SPICES' and here in Nottingham we are going to see if working with people in the community instead of at the doctor's surgery, we can spread the message quicker and further.

If you choose to take part we will ask you to complete a simple survey. From the we will be able see how well you are looking after your heart in terms of your lifestyle. Then there will be three possible options:

If the data you provide suggests you may need to make some lifestyle changes we will recommend that you make an appointment to see your doctor. As researchers we cannot give any medical advice, but it would be inappropriate for us to ignore any signs of an unhealthy lifestyle that could give rise to heart problems.

If the data you provide suggests you have a healthy lifestyle, then this is positive news and we'll talk to you about how you might be able to help the project in other ways.

If you are somewhere in the middle we will show you some simple ways to reduce your risk and stay healthier for longer.

N.B. In all cases, the data you provided is for research purposes only and a decision about your health cannot be made on the basis of questionnaires only.   Whilst we advise you to see a doctor if figures are high, lower figures should not be taken to indicate a healthy heart, and the results should not be used to replace medical assessments and the taking of medical advice about other health monitoring strategies. The dividing of participants into three groups is for research purposes only and is not a medical intervention.

If you're interested please complete our survey (It might take about 10 minutes, and you will need a tape measure for one of the questions).

Our researchers will then get in touch with you about ways that we can support you to make your heart healthier.   Any information we collect will be kept securely and not shared outside of the research team. Your name and personal details will not be used in any reports, and all our records will be destroyed at the end of the project in line with the relevant GDPR legislation. Additionally you may withdraw your data at any time up to but no later than December 31st 2020 by contacting Mark Bowyer, SPICES Coordinator, Nottingham Trent University 0115 8485574 mark.bowyer@ntu.ac.uk

OK? Let's start with your agreement to take part.

For peer review only

Horizon 2020
European Union funding
for Research & Innovation

NOTTINGHAM TRENT UNIVERSITY

CONSENT FORM

'SPICES' Heart Diseases Prevention Research

You are making a decision to take part. By ticking ALL statements and signing your name below you will indicate that you have read the information provided above and decided to participate.

If you choose to discontinue participation in this study, you may withdraw at any time without judgement, or effect on your status.

CONSENT STATEMENT		Please tick if you agree
**1.**	I have received, read and understood the SPICES participant information sheet	
**2.**	I am aware that I can withdraw my participation at any time without prejudice, judgement or effect on my status in relation to Nottingham Trent University or its research partners	
**3.**	I understand that information I provide during my participation can be deleted at my request up to but no later than December 31st 2020	
**4.**	I agree to be contacted by SPICES researchers using the details that I have supplied below	
**5.**	I understand that the collection of data is not part of medical assessment or diagnosis and cannot be relied upon to reach conclusions as to the state of my health	
**5.**	I understand that any information I provide as part of the SPICES research will be managed in accordance with the EU General Data Protection Regulation (GDPR) framework (see SPICES participant information sheet)	
**6.**	I agree to take part in this research project	

Name:

Preferred contact details:

D.O.B.

Gender:

Postcode:

Signature:

Date:

……………………………………………………………………………

Staff signature:

Date:

For peer review only

A Protocol Paper: Community engagement interventions for Cardiovascular Disorders prevention in socially disadvantaged populations in the UK: An implementation research study
Final 15072019

Target Journal: Journal of Global Health Research and Policy
https://ghrp.biomedcentral.com/?gclid=Cj0KCQiA68bhBRCKARIsABYUGifuKd-xktjcmV7tn3r7G-IEqS5rAb6QmiEl6P9dXGBdNRDhsIPVzA0aAiJWEALw_wcB

Papreen Nahar[1], Harm van Marwijk[1], Linda Gibson[2], Geofrey Musinguzi[3], Sibyl Anthierens[4], Elizabeth Ford [1], Stephen A Bremner [1], Mark Bower [2], Jean Yves Le Reste[5], Tholene Sodi [6], Hilde Bastiaens[4]

Corresponding author: Dr Papreen Nahar, Department of Primary Care and Public Health, Brighton and Sussex Medical School, UK. The University of Sussex. E-mail: P.Nahar@bsms.ac.uk

For peer review only

Abstract:

Cardiovascular disorders (CVD) are the single greatest cause of mortality worldwide. In the UK, the National Health Service (NHS) has launched an initiative of health checks over and above current care to tackle CVD. However, the uptake of Health Checks is poor in disadvantaged communities. This protocol paper sets out a UK-based study aiming to co-produce a community delivered CVD risk assessment and coaching intervention to support community members to reduce their risk of CVD.

The overall aim of the project is to implement a tailored-to-context community engagement (CE) intervention on awareness of CVD risks in vulnerable populations in high, middle and low-income countries. This paper describes the protocol for the UK sites in Sussex and Nottingham. The specific objectives of the study are to enhance stakeholder' engagement; to implement lifestyle interventions for cardiovascular primary prevention, in disadvantaged populations and motivate uptake of NHS health checks.

This study takes a mixed methods approach, combining qualitative and quantitative methods in three phases of evaluation, including pre-, during- and post-implementation. To ensure contextual appropriateness the SPICES project will organize a multi-component community-engagement intervention implementation. For the qualitative component, the pre-implementation phase will involve a contextual assessment and stakeholder mapping, exploring potentials for CVD risk profiling strategies and led by trained Community Health Volunteers (CHV) to identify accessibility and acceptability. The during-implementation phase will involve healthy lifestyle counselling provided by CHVs and evaluation of the outcome to identify fidelity and scalability. The post-implementation phase will involve developing sustainable community-based strategies for CVD risk reduction. All three components will include a process evaluation. The theory of the socio-ecological framework will be applied to analyse the community engagement approach.

A stepped wedge quantitative evaluation of the roll out will focus on implementation outcomes such as uptake and engagement and changes in risk profiles. The quantitative component includes pre and post-intervention surveys.

The research project will ultimately develop a sustainable community engagement-based strategy for the primary prevention of CVD, to support or enhance the performance of NHS health care.

Key words: Implementation research, Cardiovascular disorders prevention, community engagement.

Introduction:

Cardiovascular disorders (CVD) are the single greatest cause of mortality worldwide each year, estimated to contribute to 31% of all deaths globally (1). Tackling CVD is an international priority and there have been many global initiatives such as the "Global Hearts" programme, a package launched by the World Health Organisation (WHO) and partners, to enhance the prevention and control of CVD. Some risk factors for CVD are non-modifiable, such as age, ethnicity and family history (2). Some other risk factors for CVD are modifiable, such as smoking, a lack of physical activity, being overweight, lower consumption of fruit and vegetables, high blood pressure, diabetes and high cholesterol (2). These risk factors can be changed through lifestyle or behavioural modifications. There is evidence of a social gradient in the prevalence of CVD, which points to associations between social and financial deprivation, vulnerability and risk factors for CVD. (3).

In 2015, CVD was the leading cause of mortality in the context of all chronic diseases, accounting for 27% and 25% of deaths in men and women respectively, in the UK(2). Coronary heart disease (CHD) and stroke were the main CVDs responsible for this mortality of men and women across all ages. As per British Heart Foundation report in 2017 CVD has a huge financial burden with annual associated healthcare costs estimated to be £9 billion annually in the UK (2). The UK has a standardised CVD death rate of 265.1 per 100,000 (2).

In the UK, the National Health Service (NHS) has launched the Health Check initiative aimed to prevent CVD. It is a national risk assessment and management program, free to adults aged 40 to 74 living in England, who do not currently have any vascular disorders and are not being treated for certain risk factors such as diabetes (4). It aims to assess the 10-year risk of CV events and disorders. Risk is assessed using QRISK2 (5), a tool which involves collection of the following information: age, gender, ethnicity, smoking status, family history of CHD, body mass index (BMI), cholesterol test, systolic and diastolic blood pressure, levels of physical activity, and alcohol consumption. Attendees receive a low (<10 % chance of event in 10 years), medium (>10 % but <20 %), or high (>20 %) 10-year cardiovascular (QRISK2) score. Above the 10% cut-off, attendees are offered a discussion with a qualified person, such as a nurse, about lifestyle and motivation to change, which may include goal setting and plans for follow up. Patients may also be offered medication for cholesterol and blood pressure. The NHS Health Check is recommended to be undertaken every five years.

Modelling predicted that the NHS Health Check could prevent 1,600 heart attacks and strokes each year if implemented as intended (6). Whilst evidence suggests that the Health Check programme has the potential to reduce CVD events and has therefore been rolled out nationally across the UK, its implementation has been poor, especially in some of the most disadvantaged groups at highest risk of developing CVD. In 2014, Public Health England (PHE) issued a call for action to increase the uptake rate of NHS Health Checks to 75% (7) and to increase awareness of risk and engagement with existing resources. Yet, as of 2017, current uptake remains far from this target with current predictions suggesting only 40% of the eligible population will receive one (8), due to the fact that uptake is low (48%) even when Health Checks are offered. (8) (9)

Data from some regions with very large ethnic minority community and socio-economically challenged populations showed that only 45% of patients who were invited for the check attended and subsequently received some form of counselling when they needed it. Authors have discussed how higher uptake in deprived communities would reduce the possibility of exacerbation of inequalities (10). Difficulty with accessing general practices, especially among socially vulnerable groups, has been highlighted as a common barrier to

For peer review only - http://bmjopen.bmj.com/site/about/guidelines.xhtml

attendance at Health Checks (11). A community-based engagement approach, which takes the CVD risking profiling and affiliated advice processes outside of the formal healthcare facility setting, has the potential to improve access to Health Checks and could be an effective and scalable way for improving the implementation and uptake of Health Checks. Community engagement (CE) has been conceptualised as "the process of working collaboratively with and through groups of people affiliated by geographic proximity, special interest, or similar situations, to address issues affecting the well-being of those people" (12). A review of community engagement interventions found them to be effective in improving health behaviours (such as physical activity), health consequences and psychological outcomes (i.e. self-efficacy and perceived social support) (13). Community-based intervention programmes have been implemented to increase the uptake of cancer screening programmes. The programmes have been found to be effective in increasing outcomes such as recognition, receipt and maintenance of screening behaviours (14). The CE approach offers the opportunity for task-shifting and owning the programme, whereby trained non-healthcare-professionals can perform CVD risk profiling assessments to individuals who might not otherwise be captured by the formal care pathway.

There is evidence that CVD risk assessments can be successfully delivered by Community Health Workers (CHWs), outside or inside the healthcare system. An observational study conducted in Bangladesh, Guatemala, Mexico and South Africa has demonstrated that CHWs who are inhabitants of their local communities and were fluent in the community's predominant language, can perform community-based screenings to predict CVD risk as effectively as physicians and nurses when using the non-laboratory-based Gaziano CVD risk scoring tool (15). CHWs were trained for 1-2 weeks, and results showed a 96.8% agreement between risk scores assigned by CHWs and healthcare professionals. However, a question remains whether the model taken in the global South could be transferrable to the global North, but it is at least plausible that a community-based engagement approach will be effective for increasing the uptake of CVD risk assessment, particularly in disadvantaged communities of the global North. There are examples in the global North on community engagement in health (16), and indeed the voluntary or 'third sector' have been considered key partners in the delivery of health promotion initiatives in the community (17).

Authors have argued that because of the current economic constraints with the formal healthcare system, the focus should be upon supplementing a service delivery model with an alternative community development model (18). The key aspect is supplementing formal service delivery by utilizing communities' 'social capital'. The term 'social capital' describes the various resources that people may have through their relationships in families, communities and other social networks. Social capital bonds people together and helps them make links beyond their immediate friends and neighbours (19).

For this compassionate community approach to work, contextual appropriateness and cultural sensitivity of an intervention is crucial (20). Following this argument, the SPICES project in two areas of England, East Sussex and Nottingham, will co-produce a multi-component community-engagement intervention focussed on delivering a Health Check-style CVD risk screening, with appropriate health coaching and follow-up, in a community setting (21) and delivered by community volunteers. The intervention will be trialled and evaluated using a mixed methods approach using both qualitative and quantitative methods. The specific objectives of the project are:

To evaluate with stakeholders the potential for a community engagement-based CVD primary prevention programme to support or enhance the NHS Health Check Programme.

To co-produce with the communities an evidence-informed community-engagement intervention on CVD risk, based on the NHS Health Check model, tailored to the context in disadvantaged communities in East Sussex and Nottingham.

For peer review only - http://bmjopen.bmj.com/site/about/guidelines.xhtml

To implement the intervention in the local communities where it was co-produced, and:
-assess its effectiveness versus routine care.
-assess the fidelity, feasibility, acceptability, uptake and scalability of the implementation.
-carry out a process evaluation of the intervention and its implementation

This project is part of the SPICES (Scaling-up Packages of Interventions for Cardiovascular disease prevention in selected sites in Europe and Sub-Saharan Africa) project (22). This is a Horizon 2020 project financed by the European Commission that aims to address the CVD burden. The overall objective is to implement and evaluate a comprehensive cardiovascular disease (CVD) prevention and care program at the community level in five countries (Belgium, France, Uganda, UK, South Africa), to identify and compare barriers and facilitators for implementation across study contexts and to develop a learning community.

Methods:

Theoretical Model

SPICES is underpinned by the Consolidated Framework for Advancing Implementation Research (23), and Reach, Effectiveness, Adoption, Implementation, and Maintenance (sustainability) framework /RE-AIM models (24). We also recognize as a global health project the need for the use of the socio-ecological framework (25). As mentioned above, this model allows an understanding of the multifaceted and interactive effects of personal, social and environmental factors that determine behaviour; and for identifying behavioural and organisational leverage points and intermediaries for health promotion within organisations and communities.

Study Design

A mixed-methods research methodology will be applied strategically combining qualitative and quantitative methods at both sites. This approach will allow us to model the iterative nature of coproduction and implementation research without compromising the rigour of the study (26; 27). The study will take place in three phases:
- Pre-intervention; when stakeholder mapping and local adaptation will be carried out
- Intervention roll out, recruitment and evaluation
- Post-intervention evaluations and feedback (28)- Process evaluation will be conducted in all three phases.

Stage 1: To explore the implementation context and co-produce the intervention.

To explore the context where the implementation will take place we will carry out several mappings. These will give us the context for recruitment and implementation co-design.
They are as follows:
(a) Mapping the potential stakeholders: Mapping of the stakeholders will be done to find out who are the key stakeholders, where they come from, and what they are looking for in relationship to the study objectives(29). To engage the community, it is essential to map the community stakeholders (civil society organisations) as they are the gatekeepers of the community. Three levels of stakeholder mapping will be carried out, namely at macro, meso and micro levels.
Macro-level: stakeholders will be identified via the existing link of PI of the project in the community through meetings with local public health or other relevant departments and CSOs and using online information. Interviews with this category of stakeholders will provide insights into implementation sustainability.
Meso-level: a strategic community volunteer organisation mapping will be carried out to find out the relevant organisations, through which individual volunteers will be selected. This will

For peer review only - http://bmjopen.bmj.com/site/about/guidelines.xhtml

be done in three ways; using online searches, personal contacts and snowballing. In-depth interviews will be conducted to co-design a sustainable intervention implementation.
Micro-level: an exploration will be done with volunteers and end-user groups to co-design an acceptable and feasible intervention implementation.
(b) Mapping the context: social mapping will be carried out to explore the lifestyle context of the community via observations.
(c) Training of volunteers by professional health trainers and researchers following current NICE Public health guideline [PH6] 'Behaviour change: general approaches' (30)
(d) CVD risk profiling by trained community health volunteers (CHV).
CHVs will be the persons who have been involved in health-related volunteering for example volunteers who worked in cancer prevention, health check, healthy lifestyle etc programme. They will be involved in the screening of the CVD risk population and implement the designed intervention.

Expected Intervention

The final elements of the intervention will be co-produced within each community setting, following the mapping exercises outlined above. As outlined in the CFAIR (23), interventions are usually composed of a core component which is essential and indispensable, and an adaptable periphery, which can and should be tailored to the specific setting and users.
Core Components: Following identification of moderate to high risk for CVD, the intervention will consist of non-clinical (non-NHS) individual or group support sessions within the community, focus on motivating behaviour change. Each participant will be supported by trained SPICES researchers or community health workers to identify behaviour change goals, produce action plans to achieve them, and problem solve in cases of unexpected outcomes. All SPICES Interventions are theoretically grounded in the theory of behaviour change and deploy the strongest evidenced Behaviour Change Techniques (BCTs) from the literature.

1. Goal Setting
2. Action Planning
3. Problem Solving
4. Motivational Interviewing
5. Feedback on progress towards goals
6. Feedback on the health impact

The use of these six BCTs are focussed in SPICES on five Target Behaviours:

1. Reduce/cease smoking
2. Increase moderate physical activity
3. Reduce fat, salt, the sugar content of the diet
4. Increase fibre, oily fish, fruit and vegetable content of the diet
5. Reduce sedentary hours

Community Adaptation: The exact elements of the support sessions will be tailored to individuals and their community context, will be determined during iterative co-design with community representatives, and will be drawn from the following (31; 32):

Step-I - Goal setting

Every participant should receive specific healthy lifestyle counselling/feedback based on their individual item InterHE ART assessment scores (the moderate group). The feedback will be based on a review of international guidelines conducted as formative work for the SPICES project intervention (33). SPICES behaviour change support sessions will be based on the best-evidenced approaches to healthy lifestyle modification and community context and preferences.

For peer review only - http://bmjopen.bmj.com/site/about/guidelines.xhtml

Two further screening questionnaires may be used with individuals to assess the benefit of possibly behaviour change;

- International Physical Activity Questionnaire (IPAQ, see appendix) is an internationally validated instrument to capture information about weekly physical activity habits, behaviours and routines.
- The Dietary Approaches to Stop Hypertension Questionnaire DASH-Q is a self-reporting lifestyle questionnaire (see appendix) to capture information about weekly dietary habits, routines and behaviours, based around ‘Dietary Approach to Stopping Hypertension’ (34).
- Current behaviours audit: Using food and physical activity diaries prepared by and provided to participants by the SPICES research team, participants will be encouraged to complete an audit of one week of current dietary and physical activity behaviours, habits and routines to establish a baseline from which goals for change and improvement can be set in negotiation with SPICES CHVs
- The ABCD self-reporting questionnaire (see appendix) to assess participant perception of personal heart health risk.
- The EQ-5D-5L internationally validated Quality of Life self-reporting questionnaire (see appendix).

Step-II - Action Planning by the participants

Participants will be asked to create an action plan with appropriate goal setting for two behaviours (diet and exercise habits) in relation to when, where and how they will undertake, for example, physical activity (based on the item stems used by Luszczynska & Schwarzer (35); when the physical activity will be performed, where it will be performed, how often it will be performed. The way goals are reached and plans recorded will be co-designed with key stakeholders.

Step III - Problem-solving

CHVs will help participants to analyse any factors which may influence their ability to achieve the goals and to generate strategies which could help them overcome these barriers.

CHVs will use Motivational Interviewing techniques about health, social and environmental, and emotional barriers and consequences. Culturally and context-sensitive information will be provided (both verbally and in the form of leaflets) about the importance of eating healthily, being physically active, and not smoking for positive outcomes on physical and mental health.

Trial of Intervention

This will be an open-label, non-controlled trial, examining fidelity, feasibility, acceptability, uptake and scalability of the intervention.

Eligible Population

Economically disadvantaged, lower socio-economic status (SES) postcodes, will be identified using the overall Index of Multiple Deprivation (36a); Participants’ SES will be determined by their postcode of residence. Any resident aged 18 or above living in the study postcode areas will be eligible to take part in the baseline assessment for the study.

Study Sample Size

The sample size calculation for the quantitative study used statistical modelling for a stepped wedge design, randomising community centres over time with the InterRHEART score as the outcome (90% power for 5% significance, effect size (Cohen’s D)=0.25, intracluster correlation coefficient of 0.05, control clusters crossing to intervention in 4 steps, participant autocorrelation=0.7 and cluster autocorrelation=0.9), which requires a total of at least 144 persons. This needs approximately 200-300 people across the two sites as we expect a high level of attrition (as much as 50%). At least 1500 community members will need to be screened to achieve this recruitment (37).

For peer review only - http://bmjopen.bmj.com/site/about/guidelines.xhtml

Recruitment of Community Health Volunteers and Trial Participants

Community Health Volunteers (CHVs) will be recruited to perform CVD risk profiling assessments through a combination of 'doorstep outreach' and 'intermediary organisation recruitment' approaches in East Sussex and through existing community and neighbourhood groups with the assistance of partners such as Self-Help UK, the Renewal Trust, Nottingham CVS and others in Nottingham.

For recruitment of trial participants, we will use similar community networks, and endeavour to use quota sampling, in that we will seek to ensure the inclusion of high, low and median income neighbourhood residents, citizens from the South Asian and African diasporas; and will encourage participants to refer others to the researchers who may be able to potentially contribute or participate in the study.

Baseline Screening of CVD Risk

Participants will fill in the validated InterHEART score to determine suitability for the trial. The non-laboratory-based InterHEART scoring tool requires minimal resources which is practical for use within the community. There is also evidence to suggest that the InterHEART can reliably predict the incidence of CVD and death in low, middle, and high-income countries for a mean follow-up of 4.1 years (38). Risk is expressed as a score from the InterHEART: 0-9 (Low risk), 10-15 (moderate risk), and 16-48 (high risk). The InterHEART scoring tool will be translated onto a mHealth platform so that the trained CHVs can easily administer them during community engagement and contact, and online data will directly reach the University repository in real time from the respondents' device.

Participants who score moderate or high risk in the baseline assessment will be invited to participate in the intervention. The moderate risk (amber) score population will be selected for participation in the intervention (=score of 10 or higher), and will fill out the self-completion survey InterHEART scoring every three months. The InterHEART scoring tool will be translated onto a mHealth platform so that the trained CHVs can easily administer them during community engagement and contact, and online data will directly reach the University repository in real time from the respondents' device (39).

Clinical Outcome and Follow-Up

The primary outcome will be the change in the risk score among people who complete the community delivered CVD risk assessment and coaching. Secondary outcomes will be gathered from participants identified as 'high risk'. Numbers of participants who a) self-referred (defined as having contacted their GP surgery requesting for a formal check-up) and b) completed the NHS Health Checks

Data collected during the trial of intervention will comprise:

- Self-reported lifestyle (modifiable and non-modifiable) risk factors gathered through survey instruments and interviews.
- Observed/measured data on all participants' age, gender, ethnicity, postcode, hip to waist ratio, gathered by trained volunteers.
- Quantitative analysis of changes in behavioural intention, target behaviours, and measurable CVD risk.

Outcomes will be assessed at three months post-intervention.

Post-intervention Qualitative Evaluation and Feedback

In the post-intervention phase, a qualitative evaluation will be carried out during which

The following implementation parameters will be assessed:

For peer review only - http://bmjopen.bmj.com/site/about/guidelines.xhtml

1. The impact on awareness of CVD risks and mitigating measures, amongst disadvantaged populations of a community-based, non-clinical, CVD risk scoring tool and education.
2. The impact of the community based non-clinical CVD risk scoring tool and education on motivational healthy lifestyle among disadvantaged populations.
3. The facilitators and barriers to the adoption of a community-based CVD prevention implementation programme, by target populations.
4. The perspectives of participants regarding their experience and meaning of the intervention.

These will be explored with a subset of intervention participants using focus groups or/and in-depth interview and community mapping. Participants for the qualitative component will include adult volunteers, public health stakeholders and people within the community. The community volunteers will be selected via community organisations and public health stakeholders will be selected from the same area of the research site. Community participants for the qualitative component will be selected via the community volunteers. This post-intervention qualitative study will include randomly selected trial participants.

We will be flexible in terms of the number of participants for the qualitative component. The number will be determined through the principle of saturation and diversity. However, from each site, we will aim to include at least 12 respondents and a maximum of 30 respondents from different categories (40; 41).

Process evaluation of the intervention

To assess the fidelity of the conclusions concerning the project's effectiveness, ongoing assessment, monitoring, and enhancement is important. If significant results are found, but fidelity was not assessed, it cannot be determined if the effectiveness is attributable to unintentionally added or omitted components. Bellg and colleagues (42) propose that considerations of fidelity should permeate all stages of the study: design of the study, provision of training, delivery of the intervention, receipt of the intervention, and re-enactment of skills. As a result, we will carry out a process evaluation of the project. This will be done through Process Documentation of all the stages of this project including community volunteers mapping, Healthy lifestyle counselling, action planning and problem-solving.

Thirsk and Clark (43) argue how health-care interventions need to be understood in ways that are responsive to the complexities and intricacies of programs, people and places. They emphasise the understanding of the comprehensive experience of the persons who are delivering and receiving the intervention. Process Evaluation is a tool that can capture the intervention experience. We will be following the model designed by Moore et al (44):

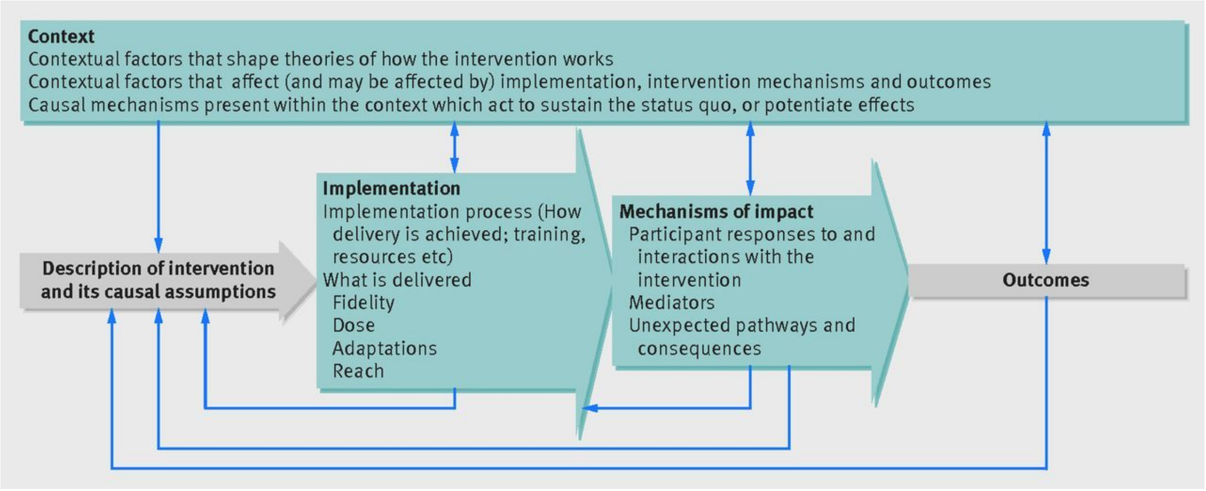

Data Analysis:
Quantitative data will be analysed using Stata version 15 or later. Descriptive statistics will summarise outcomes before and after clusters cross over to the intervention (45. Normally distributed variables will be summarised by means and standard deviations, skewed continuous variables by medians and interquartile ranges, categorical variables by frequencies and percentages. We will estimate the treatment effect using a cross-classified linear mixed effects model. A statistical analysis plan will be agreed and signed off prior to final analysis commencing. Thematic analysis of qualitative data will be carried out using a constant comparison method of analysis, which will gather and generate ideas and categories through inductive processes. The computer package NVivo will be used for primary analysis (46). Memo writing will be carried out to describe details of the interview setting and interaction of respondent and interviewer that may not be captured in audio transcriptions. This thematic analysis has deductive and inductive elements, lending itself to multidisciplinary health research (47). The analysis framework will incorporate the key theoretical constructs and respond to the context of policy and practice to include a range of deductive themes. Further themes will be induced from the interview data.

An appropriate balance of integration between empirical data and interpretation will be ensured. The investigators will extract the meaning of the empirical data and interpret them whilst acknowledging the complexity of the phenomena of CVD risk reduction in the context of community engagement (48). This method holds links to the original data and the output allows comprehensive and transparent data analysis.

Conclusion:
Given that despite the rolling out of the NHS Health Checks programme over and above current care across the UK has not been implemented as well as it could have been, especially in some of the most disadvantaged groups prone to developing CVD, the project aims to scale-up packages of interventions for cardiovascular prevention particularly to these vulnerable populations. This interdisciplinary project includes public health, social and behavioural science approaches. The main focus aspect of this project is the deinstitutionalization of health care by operating outside of formal healthcare settings. The project will emphasise on the power of citizens, combining their efforts to generate cultures of care which complement or even compensate for the inadequacies of formal systems thus sustainable. The research project will ultimately develop a community engagement-based CVD primary prevention programme to support or enhance the performance of the NHS health care.

Funding statement:
This protocol is a contextual plan for the SPICES project in the UK. The SPICES project received funding from the European Commission through the Horizon 2020 Research and Innovation Action Grant Agreement No 733356 to implement and evaluate a comprehensive CVD prevention programme in five settings: a rural & semi-urban community in a low-income country (Uganda), middle income (South Africa) and vulnerable groups in three high-income countries (Belgium, France and United Kingdom). The funder had no role in the design, decision to publish, or preparation of the manuscript.

Availability of data and materials:
A protocol should not contain any data; it sets out the research questions and how they will be addressed.

For peer review only - http://bmjopen.bmj.com/site/about/guidelines.xhtml

Ethics approval and consent to participate:
This protocol has received two ethics approval from the University of Sussex, The **BSMS** Research Governance and Ethics Committee (RGEC (ER/BSMS9E3G/1)), and from Nottingham Trent University (no. TBA). All participants will be requested to consent before enrolment into the study. All participant information will be kept confidential and accessible only to the key investigative team. All published data will be anonymised and can be accessed based on a written request to the Principal Investigator.

Competing interests:
Authors declare that they have no competing interests.

Authors' contributions:
PN has written the first draft and received feedback from HvM and SA on it. PN prepared the second draft and it received feedback from LG. The third draft received feedback from all the authors. All authors read and approved the final contextual protocol (4th version).

References:

1. World Health Organisation; WHO. *Global Health Observatory (GHO) data*. 2017. Retrieved on 18th May 2019, retrieved from https://www.who.int/gho/mortality_burden_disease/causes_death/top_10/en

2. British Heart Foundation report BHF. https://www.bhf.org.uk/informationsupport/publications/policy-documents/annual-report-2017.

3. Amuzu A, Carson C, Watt HC, Lawlor DA, Ebrahim S. Influence of area and individual life course deprivation on health behaviours: findings from the British Women's Heart and Health Study. Eur J Cardiovasc Prev Rehabil. 2009;16(2): 169-73. doi: 10.1097/HJR.0b013e328325d64d.

4.Public Health England (2013). NHS Health Check implementation review and action plan Summary, https://assets.publishing.service.gov.uk/government/uploads/system/uploads/attachment_data/file/224536/NHS_Health_Check_implementation_review_and_action_plan_summary_web.pdf 5.

5. QRISK2. 2017. https://qrisk.org/2017/

6. Public Health, England. NHS Health Checks 'prevented thousands of heart attacks'. 2016. https://www.nhs.uk/news/heart-and-lungs/nhs-health-checks-prevented-thousands-of-heart-attacks/

7. Public Health England. Factsheet: Implementation of the NHS Health Check programme. 2014. https://www.england.nhs.uk/wp-content/uploads/2014/02/pm-fs-3-1.pdf

8. Waterall Jamie. PHE. Getting Serious About CVD Prevention. Reducing Variation & Optimising Care. 2018. https://www.healthcheck.nhs.uk/seecmsfile/?id=195

For peer review only - http://bmjopen.bmj.com/site/about/guidelines.xhtml

9. Dalton AR, Bottle A, Okoro C, Majeed A, Millett C. Uptake of the NHS Health Checks programme in a deprived, culturally diverse setting: cross-sectional study. J Public Health (Oxf). 2011; 33(3):422-9. doi: 10.1093/pubmed/fdr034.

10. Robson J, Dostal I, Sheikh A, et al. The NHS Health Check in England: an evaluation of the first 4 years. 2015. BMJ Open. 2015;6: e008840. doi:10.1136/ bmjopen-2015

11. Harte Emma, Calum MacLure, Adam Martin, Catherine L Saunders, Catherine Meads, Fiona M Walter, Simon J Griffin, Jonathan Mant and Juliet A Usher-Smith (2018), Reasons why people do not attend NHS Health Checks: a systematic review and qualitative synthesis, British Journal of General Practice. 2018;68 (666): e28-e35. DOI: https://doi.org/10.3399/bjgp17X693929

12. CTSA. Principles of Community Engagement, Second Edition, NIH Publication No. 11-7782. 2011. https://.atsdr.cdc.gov/communityengagement/pdf/PCE_Report_508_FINAL.pdf

13. O'Mara-Eves A., G Brunton, D McDaid, S Oliver, J Kavanagh, F Jamal, T Matosevic, A Harden, and J Thomas. Community engagement to reduce inequalities in health: a systematic review, meta-analysis and economic analysis, Public Health Research. 2013;1:4

14. Bird JA; McPhee SJ, Ha NT, Le B, Davis T, Jenkins CN. Opening pathways to cancer screening for Vietnamese-American women: lay health workers hold a key. Prev Med. 1998;27(6):821-9.

15. Gaziano Thomas A, Shafi ka Abrahams-Gessel, Catalina A Denman, Carlos Mendoza Montano, Masuma Khanam, Thandi Puoane, Naomi S Levitt (2015). An assessment of community health workers' ability to screen for cardiovascular disease risk with a simple, non invasive risk assessment instrument in Bangladesh, Guatemala, Mexico, and South Africa: an observational study. Lancet Glob Health. 2015; 3:e556–63. http://dx.doi.org/10.1016/ S2214-109X(15)00142-4

16. Wood, J. The University as a Public Good: Active citizenship and university community engagement. International Journal of Progressive Education. 2012; 8 (3), 15-31. Retrieved from http://dergipark.org.tr/ijpe/issue/26312/277299

17. Kings Fund. 2011. Retrieved from, https://www.kingsfund.org.uk/sites/default/files/Voluntary-and-community-sector-in-health-implications-NHS-reforms-The-Kings-Fund-june-2011_0.pdf

18. Abel, J., Bowra, J., Walter, T. et al. Compassionate Community networks: Supporting home dying. BMJ Supportive and Palliative Care. 2011;1,129-133. http://dx.doi.org/10.1136/bmjspcare-2011-000068

19. Baum FE and Ziersch AM. Social capital. Journal of Epidemiology & Community Health. 2003; 57: 320-323.

20. Sallnow L. Libby, Heather Richardson, Scott Murray, Allan Kellehear. Understanding the impact of a new public health approach to end of life care: a qualitative study of a community-

For peer review only - http://bmjopen.bmj.com/site/about/guidelines.xhtml

led intervention. The Lancet. 2017; 389: Special issue 788. DOI:https://doi.org/10.1016/S0140-6736(17)30484-1

21. Boyd, Stephen McKernon, Bernie Mullin, Andrew Old. Improving healthcare through the use of co-design. The New Zeeland Medical Journal. 2012; 125:(1357).

22. Musinguzi Geofrey, Rhoda K. Wanyenze1, Rawlance Ndejjo, Isaac Ssinabulya, Harm van Marwijk, Isaac Ddumba, Hilde Bastiaens and Fred Nuwaha. An implementation science study to enhance cardiovascular disease prevention in Mukono and Buikwe districts in Uganda: a stepped-wedge design. Health Service research. 2019; 19:253 doi.org/10.1186/s12913-019-4095-0

23. Damschroder Laura J, David C Aron, Rosalind E Keith, Susan R Kirsh, Jeffery A Alexander and Julie C Lowery. Fostering implementation of health services research findings into practice: a Consolidated Framework for Advancing Implementation Science. Implementation Science. 2009; 4:50 doi:10.1186/1748-5908-4-50

24. Glasgow RE, McKay HG, Piette JD, Reynolds KD: The RE-AIM framework for evaluating interventions: what can it tell us about approaches to chronic illness management? Patient Educ Couns. 2001;44:119-127.

25. Theobald, S., Brandes, N., Gyapong, M., et al. Implementation research: new imperatives and opportunities in global health, Lancet. 2018;392: 2214–28

26. Peters DH, Adam T, Alonge O, et al. Implementation research: what it is and how to do it. BMJ. 2013; 347: f6753.

27. Brown CH, Curran G, Palinkas LA, et al. (2017). An overview of research and evaluation designs for dissemination and implementation. Annual Review of Public Health. 2017; 38:1–22

28. Greene, J. C. Mixed Methods in Social Inquiry. 2007. Jossey-Bass. San Francisco.

29. BSR. Stakeholder Mapping (2011). www.bsr.org/reports/BSR_Stakeholder_Engagement_Stakeholder_Mapping.final.pdf

30. NICE Public health guideline [PH6] 'Behaviour change: general approaches' (https://www.nice.org.uk/guidance/ph6/resources/behaviour-change-general-approaches-pdf-55457515717).

31. Michie Susan, Michelle Richardson, Marie Johnston, et al. (2013). The Behaviour Change Technique Taxonomy (v1) of 93 Hierarchically Clustered Techniques: Building an International Consensus for the Reporting of Behavior Change Interventions. Annals of Behavioural Medicine. 2013. 46;(1):81–95, https://doi.org/10.1007/s12160-013 9486-6

32. Michie, Susan, Maartje M. Van Stralen, and Robert West. "The Behaviour Change Wheel: A New Method for Characterising and Designing Behaviour Change Interventions. Implementation Science. 2011;6,1:42.

For peer review only - http://bmjopen.bmj.com/site/about/guidelines.xhtml

33. Yusuf Salim, Steven Hawken, Stephanie Ôunpuu, et al., on behalf of the INTERHEART Study Investigators. Effect of potentially modifiable risk factors associated with myocardial infarction in 52 countries (the INTERHEART study): a case-control study. Lancet. 2004; 364: 937–52

34. Sacks Frank M., Laura P. Svetkey, M.D., William M. Vollmer et al. Effects on Blood Pressure of Reduced Dietary Sodium and the Dietary Approaches to Stop Hypertension (DASH) Diet.
N Engl J Med. 2001; 344:3-10, DOI:10.1056/NEJM200101043440101

35. Luszczynska Aleksandra, Urte Scholz & Ralf Schwarzer. The General Self-Efficacy Scale: Multicultural Validation Studies, The Journal of Psychology. 2005; 139:5, 439-457, doi:10.3200/JRLP.139.5.439-457

36.. Kontopantelis E, Mamas MA, Van Marwijk H, Ryan AM, Buchan IE, Ashcroft DM, et al. Geographical epidemiology of health and overall deprivation in England, its changes and persistence from 2004 to 2015: A longitudinal spatial population study. 2018; 72 (2):140–7.

37. Hooper R, Teerenstra S, de Hoop E & Eldridge S. Sample size calculation for the stepped wedge and other longitudinal cluster randomised trials. Statist. Med. 2016; 35 4718–4728

38. Yusuf Salim, Sumathy Rangarajan, Koon Teo, Shofiqul Islam, et al. Cardiovascular. Risk and Events in 17 Low-, Middle-, and High-Income Countries. N Engl J Med. 2014; 371:818-827, DOI: 10.1056/NEJMoa1311890

39. Williams Natalie Joseph, Amy Lloyd, Adrian Edwards, Lynne Stobbart, David Tomson, Sheila Macphail, Carole Dodd, Kate Brain, Glyn Elwyn, Richard Thomson. Implementing shared decision making in the NHS: lessons from the MAGIC programme. BMJ. 2017a;357:j1744 doi: https://doi.org/10.1136/bmj.j1744

40. Guest, G., Bunce, A., & Johnson, L. How Many Interviews Are Enough?: An Experiment with Data Saturation and Variability. Field Methods. 2006; 18 (1): 59–82. https://doi.org/10.1177/1525822X05279903

41. Malterud K, Siersma VD, Guassora AD. Sample Size in Qualitative Interview Studies: Guided by Information Power. Qual Health Res. 2015; 1–8.

42. Bellg, A. J., Borrelli, B., Resnick, B., Hecht, J., Minicucci, D. S., Ory, M., et al. Treatment Fidelity Workgroup of the NIH Behavior Change Consortium. (2004). Enhancing Treatment Fidelity in Health Behavior Change Studies: Best Practices and Recommendations From the NIH Behavior Change Consortium. Health Psychology. 2004; 23(5):443-451. http://dx.doi.org/10.1037/0278 6133.23.5.443

43. Thirsk Lorraine M. and Alexander M. Clark. Using Qualitative Research for Complex Interventions: The Contributions of Hermeneutics. International journal of qualitative methods. 2017; 16:1-10. doi.org/10.1177/1609406917721068

For peer review only - http://bmjopen.bmj.com/site/about/guidelines.xhtml

44. Moore Laurence, Alicia O'Cathain, Tannaze Tinati, Daniel Wight, Janis Baird (2015). Process evaluation of complex interventions: Medical Research Council guidance. The BMJ. 2015;350:h1258. doi: 10.1136/bmj.h1258

45. StataCorp. 2017. *Stata Statistical Software: Release 15*. College Station, TX: StataCorp LLC

46. Ritchie Jane, Jane Lewis, Carol McNaughton Nicholls, Rachel Ormston. Qualitative Research Practice: A Guide for Social Science Students and Researcher; 2013. Sage Publications.

47. Gale NK, Heath G, Cameron E, Rashid S, Redwood S. Using the framework method for the analysis of qualitative data in multi-disciplinary health research. BMC Med Res Methodol. 2013;(13):117. doi.org/10.1186/1471-2288-13-117

48. Green J, Thorogood N. Qualitative methods for health research. 2005. London: Sage Publications Ltd.

Authors Information:

1. Papreen Nahar. Department of Primary Care and Public Health, Brighton and Sussex Medical School. University of Sussex, UK.
1. Harm van Marwijk. Department of Primary Care and Public Health, Brighton and Sussex Medical School. The University of Sussex. UK
2. Linda Gibson: School of Social Sciences. Nottingham Trent University, UK
3. Musinguzi Geofrey. Department of Disease Control and Environmental Health, School of Public Health, College of Health Sciences. Makerere University, Uganda
4. Sibyl Anthierens. Department of Primary and Interdisciplinary Care, University of Antwerp, Belgium
1. Elizabeth Ford. Department of Primary Care and Public Health Brighton and Sussex Medical School. University of Sussex, UK
1. Stephen A Bremner. Department of Primary Care and Public Health Brighton and Sussex Medical School. University of Sussex, UK
2. Mark Bower. School of Social Sciences, Nottingham Trent University, UK
5. JY Reste. Faculté de médecine et des sciences de la santé, Université de Bretagne Occidentale, Brest, France
6. Sodi Tholene. Department of Psychology. University of Limpopo, South Africa
4. Hilde Bastiaens. Department of Primary and Interdisciplinary care. University of Antwerp, Belgium

For peer review only - http://bmjopen.bmj.com/site/about/guidelines.xhtml

Appendix 6

ABCD subscale and selected INTERHEART variable correlation values from Nottingham study compared with values reported in the original Woringer study.

		Knowledge	Perceived Risk	Perceived Benefit	Healthy Intentions	IMD2010 Quintile	BMI/W2 Hr	Qrisk2/ INTERHEART
Knowledge	Correlation Coefficient		-.124/ **.013**	-.148/ **-.021**	-.106/ **-.039**	-.002/ .085	-.225/ -.084	-.007/ -.018
	Sig 2 tailed		.236/ .722	.175/ .645	.319/ .400	.986/ .066	.021/ .082	.941/ .714
	N		93/462	86/462	91/462	99/466	105/433	104/436
Perceived Risk	Correlation Coefficient			-.195/ -.112	-.188/ -0.36	.239/ .039	.389/ .182	.220/ .356
	Sig 2 tailed			.080/ .016	.088/ .441	.025/ .397	.000/ .000	.036/ .000
	N			82/462	84/462	87/466	92/433	91/436
Perceived Benefits	Correlation Coefficient				.533/ .383	-.287/ .071	-.068/ .000	-.118/ -.164
	Sig 2 tailed				.000/ .000	.009/ .127	.538/ .997	.284/ .001
	N				83/462	81/466	85/433	84/436
Healthy Intentions	Correlation Coefficient					-.261/ .098	.084/ .044	-.072/ -.079
	Sig 2 tailed					.016/ .034	.430/ .365	.504/ .100
	N					85/466	90/462	89/436

Correlations

Correlations

Correlations

			knowledge score	Risk score	Benefit score	Diet score	Smoke score	total_score
Spearman's rho	knowledge score	Correlation Coefficient	1.000	.118**	.103*	.078	-.079	.006
		Sig. (2-tailed)	.	.009	.023	.086	.082	.896
		N	483	483	483	483	483	440
	Risk score	Correlation Coefficient	.118**	1.000	-.003	.057	.107*	.371**
		Sig. (2-tailed)	.009	.	.950	.212	.019	.000
		N	483	483	483	483	483	440
	Benefit score	Correlation Coefficient	.103*	-.003	1.000	.538**	.009	-.236**
		Sig. (2-tailed)	.023	.950	.	.000	.851	.000
		N	483	483	483	483	483	440
	Diet score	Correlation Coefficient	.078	.057	.538**	1.000	-.022	-.143**
		Sig. (2-tailed)	.086	.212	.000	.	.635	.003
		N	483	483	483	483	483	440
	Smoke score	Correlation Coefficient	-.079	.107*	.009	-.022	1.000	.240**
		Sig. (2-tailed)	.082	.019	.851	.635	.	.000
		N	483	483	483	483	483	440
	total_score	Correlation Coefficient	.006	.371**	-.236**	-.143**	.240**	1.000
		Sig. (2-tailed)	.896	.000	.000	.003	.000	.
		N	440	440	440	440	440	440

** Correlation is significant at the 0.01 level (2-tailed).

* Correlation is significant at the 0.05 level (2-tailed).

For peer review only

Appendix 5.

Item Analysis of published ABCD Risk Questionnaire sub-scales plus 5 unpublished items relating to smoking compared to Item Analysis of recommended edited ABCD Risk Questionnaire sub-scales plus 5 unpublished items relating to smoking.

Table 1. Item Analysis of published ABCD Risk Questionnaire sub-scales plus 5 unpublished items relating to smoking

**Perceived Risk of Heart Attack/ Stroke<br>8 Items<br>Cronbach's Alpha .861<br>(0.84,0.88) 95% CI**	**Inter-item correlation**	**Corrected Item-total correlation**	**Cronbach's alpha if item deleted**
It is likely that I will suffer from a heart attack or stroke in the future	**.832**	.756	.826
It is likely that I will have a heart attack or stroke some time during my life	**.869**	.777	.824
I feel I will suffer a heart attack or stroke some time during my life	**.616**	.784	.824
There is a good chance I will experience a heart attack or stroke in the next 10 years	**.729**	.722	.832
I am not worried that I might have a heart attack or stroke	.403	.624	.843
My chances of suffering a heart attack or stroke in the next 10 years are great	.245	.544	.852
It is likely that I will have a heart attack or stroke because of my past/present behaviours	.266	.319	**.876**
I am concerned about the likelihood of having a heart attack or stroke in the near future	.259	.387	**.870**
**Perceived Benefits and Intentions to Change<br>7 items<br>Cronbach's Alpha .801**	**Inter-item correlation**	**Corrected Item-total correlation**	**Cronbach's alpha if item deleted**
I am thinking about exercising at least 2.5 hours a week	**.727**	**.605**	.760
I intend or want to exercise at least 2.5 hours a week	.442	**.651**	.752
When I exercise for at least 2.5 hours a week I am doing something good for the health of my heart	.426	.593	.769
I am confident that I can maintain a healthy weight by exercising at	.294	.452	.790

For peer review only

least 2.5 hours a week within the next 2 months			
I am not thinking about exercising at least 2.5 hours a week	.264	.508	.781
When I eat at least 5 portions of fruit and vegetables a day I am doing something good for the health of my heart	.483	.483	.783
Increasing my exercise to at least 2.5 hours a week will decrease my chances of having a heart attack or stroke	.326	.474	.786
**Healthy Eating Intentions 3 items Cronbach's Alpha .787 (95% CI**	**Inter-item correlation**	**Corrected Item-total correlation**	**Cronbach's alpha if item deleted**
I am confident that I can eat at least 5 portions of fruit and vegetables a day within the next 2 months	.555	.533	**.812**
I am thinking about eating at least 5 portions of fruit and vegetables a day	**.683**	**.732**	.596
I am not thinking about eating at least 5 portions of fruit and vegetables a day	.424	**.624**	.713
**Perceived Benefits and Intentions to Stop Smoking 5 Items Cronbach's Alpha .943 95% CI**	**Inter-item correlation**	**Corrected item-total correlation**	**Cronbach's alpha if item deleted**
I am thinking of stopping smoking within the next 2 months	**.654**	.848	.932
I have reduced or stopped smoking	**.694**	.751	**.949**
I intend or want to stop smoking	**.829**	.906	.919
If I stop smoking it will reduce my chances of having a heart attack or stroke	**.834**	.886	.922
I am not thinking about stopping smoking	**.789**	.872	.925

For peer review only

Table 2. Item Analysis of edited ABCD Risk Questionnaire sub-scales plus 5 unpublished items relating to smoking.

**Perceived Risk of Heart Attack/ Stroke<br>5 Items<br>Cronbach's Alpha .86 (0.84,0.88) 95% CI<br>Omega 0.85 (0.83, 0.88) 95% CI**	**Inter-item correlation**	**Corrected Item-total correlation**	**Cronbach's alpha if item deleted**
It is likely that I will have a heart attack or stroke some time during my life	**.869**	.777	.824
There is a good chance I will experience a heart attack or stroke in the next 10 years	**.729**	.722	.832
I am not worried that I might have a heart attack or stroke	.403	.624	.843
It is likely that I will have a heart attack or stroke because of my past/present behaviours	.266	.319	**.876**
I am concerned about the likelihood of having a heart attack or stroke in the near future	.259	.387	**.870**
**Perceived Benefits and Intentions to Change<br>6 items<br>Cronbach's Alpha .84 (.81-.86) 95% CI<br>Omega 0.82 (0.78, 0.85) 95% CI**	**Inter-item correlation**	**Corrected Item-total correlation**	**Cronbach's alpha if item deleted**
I am thinking about exercising at least 2.5 hours a week	**.727**	**.605**	.760
I intend or want to exercise at least 2.5 hours a week	.442	**.651**	.752
When I exercise for at least 2.5 hours a week I am doing something good for the health of my heart	.426	.593	.769
I am confident that I can maintain a healthy weight by exercising at least 2.5 hours a week within the next 2 months	.294	.452	.790
I am not thinking about exercising at least 2.5 hours a week	.264	.508	.781
Increasing my exercise to at least 2.5 hours a week will decrease my chances of having a heart attack or stroke	.326	.474	.786
**Healthy Eating Intentions<br>4 items**	**Inter-item correlation**	**Corrected Item-total correlation**	**Cronbach's alpha if item deleted**

**Cronbach's Alpha .84 (.81-.86) 95% CI Omega 0.84 (0.81, 0.88) 95% CI**			
I am confident that I can eat at least 5 portions of fruit and vegetables a day within the next 2 months	.555	.533	**.812**
I am thinking about eating at least 5 portions of fruit and vegetables a day	**.683**	**.732**	.596
I am not thinking about eating at least 5 portions of fruit and vegetables a day	.424	**.624**	.713
When I eat at least 5 portions of fruit and vegetables a day I am doing something good for the health of my heart	.483	.483	.783
**Smoking Intentions 5 items Cronbach's Alpha .85 (.83-.87) 95% CI Omega 0.84 (0.81, 0.91) 95% CI**	**Inter-item correlation**	**Corrected Item-total correlation**	**Cronbach's alpha if item deleted**
I am thinking of stopping smoking within the next 2 months	**.654**	.848	.932
I have reduced or stopped smoking	**.694**	.751	**.949**
I intend or want to stop smoking	**.829**	.906	.919
If I stop smoking it will reduce my chances of having a heart attack or stroke	**.834**	.886	.922
I am not thinking about stopping smoking	**.789**	.872	.925

For peer review only

Without smoking items –

Non-missing samples: 420

Bartlett's Test of Sphericity (X2 = 4235.007, p-value < 0.001)

The overall KMO is 0.82, which is within the recommended range (0.8 to 1).

EFA results

- The root mean square of the residuals (RMSR) is 0.05
- Tucker Lewis Index of factoring reliability = 0.77
- RMSEA index = 0.121 and the 90 % confidence intervals are 0.113 0.129
- BIC = 165.35

Scree plot

For pe

Figure 1. 18 item ABCD Questionnaire results from Nottingham dataset

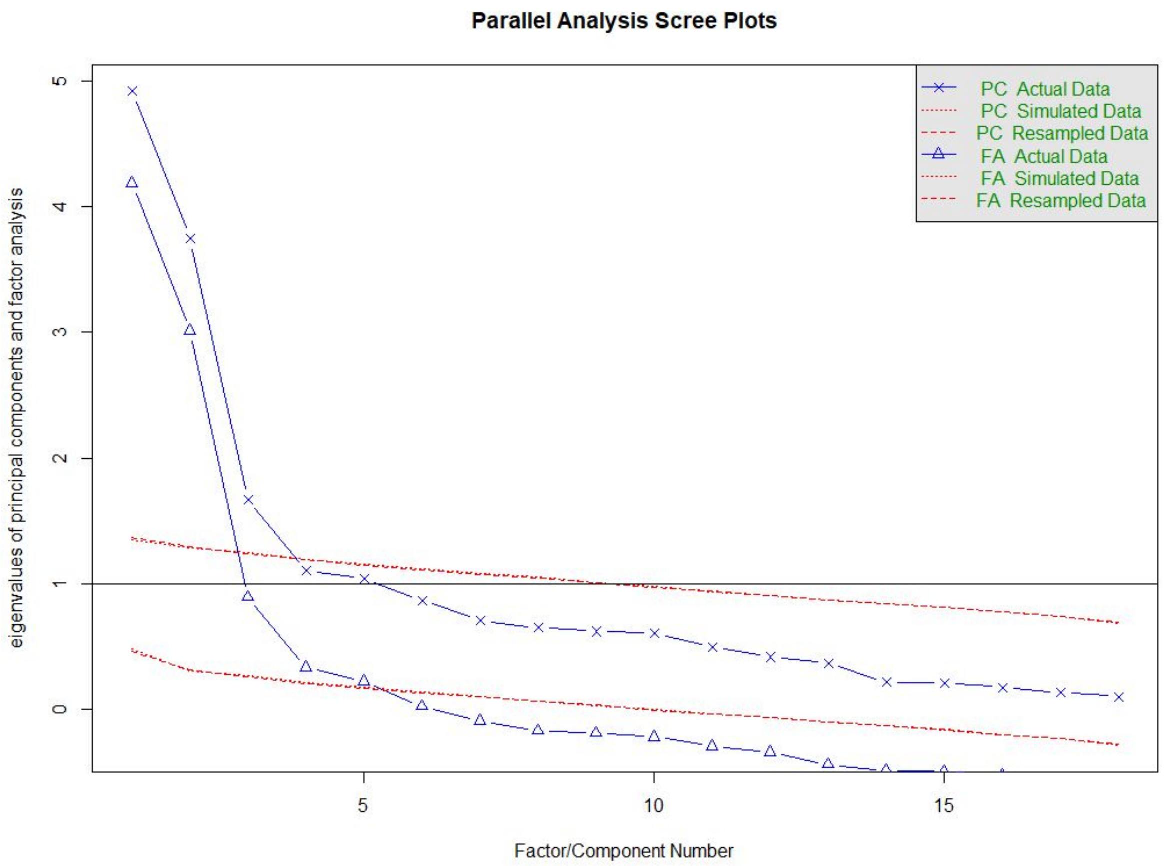

Factor loadings

Table ___. Factor loadings of the exploratory factor analysis of the risk scale without the smoking items

Item	Factor2	Factor1	Factor3	communalit	uniqueness
suffer_heartattack	0.86	0.02	-0.03	0.74	0.26
hrtattack_stroke_future	0.91	0.05	0.00	0.82	0.18
attck_stoke_during_life	0.88	0.01	0.01	0.77	0.23
hrtattack_next_10yrs	0.73	-0.07	0.01	0.55	0.45
highchance_hrtattck_10yrs	0.65	-0.10	0.01	0.44	0.56
hrtattack_past_fut_behav	0.56	-0.03	-0.01	0.32	0.68
reversenoworry	0.28	-0.11	0.10	0.10	0.90
concern_hrtattack	0.40	-0.02	0.11	0.16	0.84
think_exercise	-0.02	0.87	-0.06	0.73	0.27
want_exercise	-0.01	0.91	-0.04	0.80	0.20
exercise_gud_hrt_hlth	0.02	0.69	0.10	0.53	0.47
confident_hlth_wgt	-0.05	0.45	0.19	0.31	0.69
revnotthinkPA	0.04	0.56	0.05	0.34	0.66
fruit_veg_gud_hrthlth	0.02	0.37	0.35	0.36	0.64
high_exerc_low_hrtattack	0.02	0.39	0.27	0.30	0.70
diet_1	-0.04	0.07	0.64	0.46	0.54
diet_2	0.01	-0.01	0.93	0.85	0.15
revdiet3	-0.01	-0.03	0.78	0.60	0.40

With (might not be included in the manuscript)

	Factor 2	Factor 1	Factor 3
**SS loadings**	3.86	3.04	2.28
**Proportion Var**	0.21	0.17	0.13
**Cumulative Var**	0.21	0.38	0.51
**Proportion Explained**	0.42	0.33	0.25
**Cumulative Proportion**	0.42	0.75	1.00

With smoking items

Non-missing samples: 88

The overall KMO is 0.78, which is slightly below the recommended range (0.8 to 1).

The Bartlet's test of Sphericity is significant (X2 = 1223.459, p-value < 0.001), indicating the sample adequacy for factor analysis.

EFA results

- The root mean square of the residuals (RMSR) is 0.06
- Tucker Lewis Index of factoring reliability = 0.69
- RMSEA index = 0.129 and the 90 % confidence intervals are 0.124 and 0.136
- BIC = 440.9

Scree plot

Figure 2. Modified ABCD Questionnaire 20 items with smoking. Nottingham dataset.

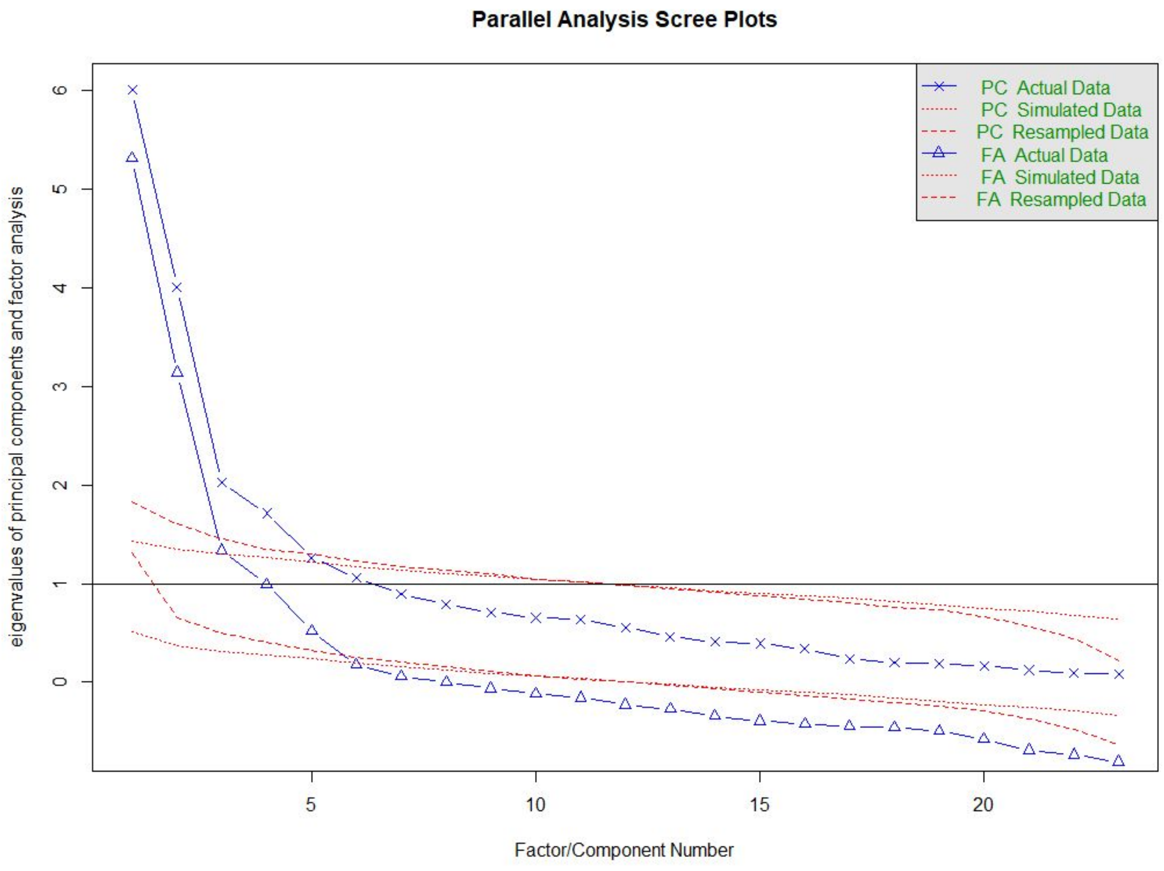

For peer review only - http://bmjopen.bmj.com/site/about/guidelines.xhtml

Factor loadings

Table ___. Factor loadings of the exploratory factor analysis of the risk scale with the smoking items

Item	Factor2	Factor3	Factor1	Factor4	Communality	Uniqueness
suffer_heartattack	0.86	-0.1	0.05	-0.02	0.76	0.24
hrtattack_stroke_future	0.91	0.06	0.02	-0.01	0.82	0.18
attck_stoke_during_life	0.88	0.02	0	0	0.77	0.23
hrtattack_next_10yrs	0.72	0	-0.09	0.01	0.54	0.46
highchance_hrtattck_10yrs	0.64	-0.03	-0.1	0.01	0.45	0.55
hrtattack_past_fut_behav	0.57	-0.07	0	0	0.33	0.67
reversenoworry	0.28	0.02	-0.14	0.1	0.1	0.9
concern_hrtattack	0.41	0.19	-0.12	0.08	0.19	0.81
think_exercise	-0.03	-0.05	0.88	-0.02	0.73	0.27
want_exercise	-0.02	0.05	0.87	-0.02	0.79	0.21
exercise_gud_hrt_hlth	0.03	0.17	0.62	0.09	0.55	0.45
confident_hlth_wgt	-0.05	0.09	0.42	0.18	0.32	0.68
revnotthinkPA	0.02	0	0.53	0.09	0.33	0.67
fruit_veg_gud_hrthlth	0.04	0.07	0.35	0.35	0.36	0.64
high_exerc_low_hrtattack	0.04	0.12	0.37	0.24	0.32	0.68
diet_1	-0.04	-0.05	0.12	0.64	0.45	0.55
diet_2	0.01	0	0.02	0.89	0.8	0.2
revdiet3	-0.01	0	-0.06	0.83	0.66	0.34
smoking_1	0.06	0.78	0.12	-0.06	0.67	0.33
smoking_2	-0.03	0.83	0.02	-0.01	0.71	0.29
smoking_3	-0.05	0.9	-0.02	-0.01	0.8	0.2
smoking_4	0.16	0.58	0.09	0.08	0.43	0.57
revsmoke5	-0.12	0.56	-0.2	0.17	0.35	0.65

With (might not be included in the manuscript)

	**Factor 2**	**Factor 3**	**Factor 1**	**Factor 4**
**SS loadings**	3.90	3.00	2.97	2.33
**Proportion Var**	0.17	0.13	0.13	0.10
**Cumulative Var**	0.17	0.30	0.43	0.53
**Proportion Explained**	0.32	0.25	0.24	0.19
**Cumulative Proportion**	0.32	0.57	0.81	1.00

For peer review only

Modified scale (20-items including the smoking items)

Non-missing samples: 89

The overall KMO is 0.79, which is slightly below the recommended range (0.8 to 1).

The Bartlet's test of Sphericity is significant (X2 = 915.41, p-value < 0.001), indicating the sample adequacy for factor analysis.

EFA results

- The root mean square of the residuals (RMSR) is 0.06
- Tucker Lewis Index of factoring reliability = 0.72
- RMSEA index = 0.118 and the 90 % confidence intervals are 0.111 and 0.126
- BIC = 153.72

Scree plot

For peer review only

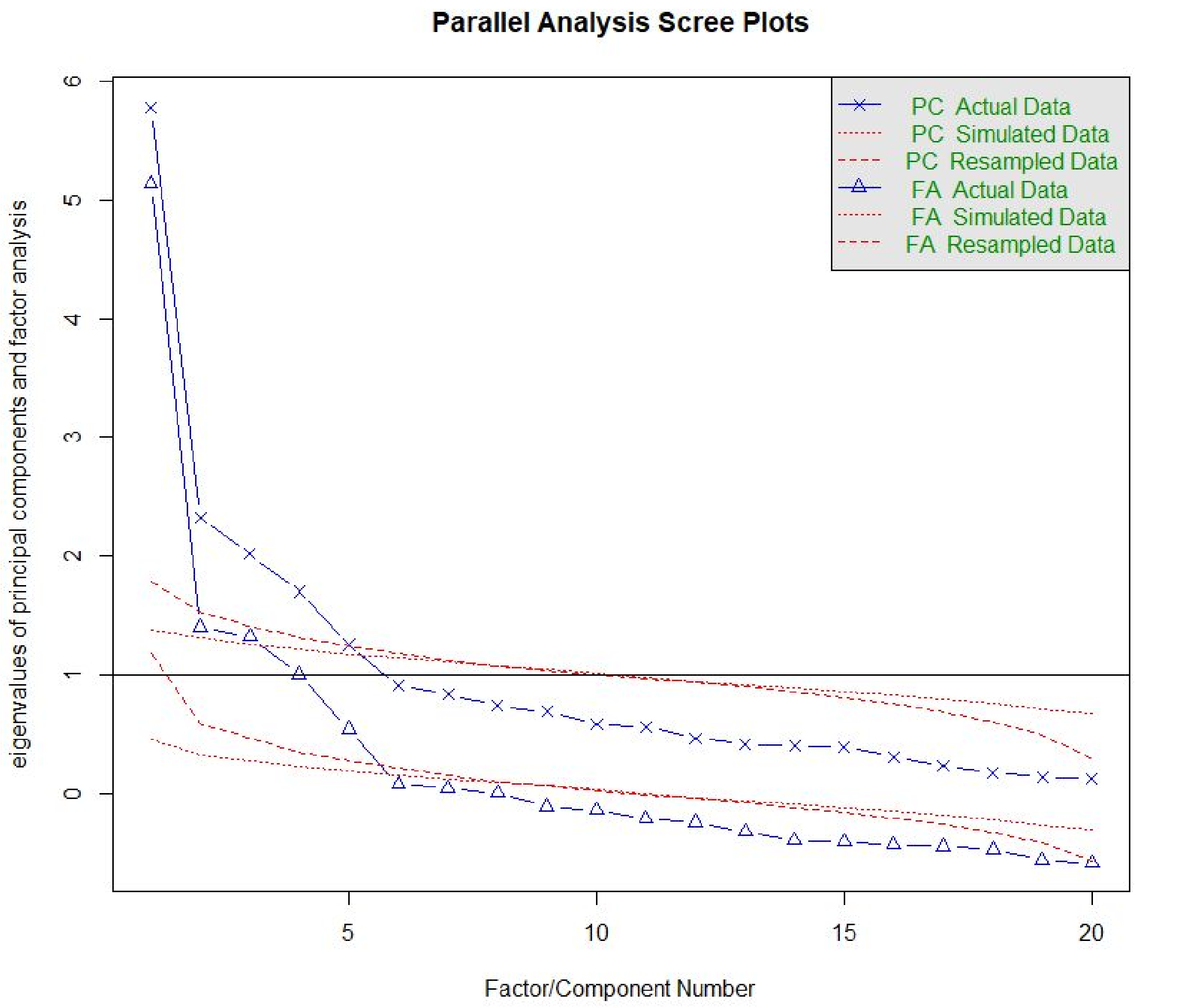

Table ___. Factor loadings of the exploratory factor analysis of the modified risk scale (20 items including the smoking items)

Item	Factor3	Factor1	Factor4	Factor2	Communality	Uniqueness
suffer_heartattack	-0.08	0.04	-0.03	0.76	0.60	0.40
hrtattack_next_10yrs	0.02	-0.08	-0.01	0.68	0.48	0.52
hrtattack_past_fut_behav	-0.04	0.01	-0.01	0.61	0.38	0.62
reversenoworry	0.04	-0.13	0.10	0.35	0.14	0.86
concern_hrtattack	0.22	-0.11	0.07	0.45	0.23	0.77
think_exercise	-0.06	0.88	-0.02	-0.04	0.74	0.26
want_exercise	0.05	0.87	-0.02	-0.02	0.79	0.21
exercise_gud_hrt_hlth	0.17	0.62	0.09	0.04	0.55	0.45
confident_hlth_wgt	0.09	0.42	0.18	-0.06	0.32	0.68
revnotthinkPA	0.01	0.53	0.09	0.03	0.32	0.68
fruit_veg_gud_hrthlth	0.08	0.35	0.35	0.07	0.37	0.63

high_exerc_low_hrtattack	0.13	0.37	0.24	0.06	0.32	0.68
diet_1	-0.06	0.12	0.64	-0.05	0.46	0.54
diet_2	0.00	0.02	0.89	0.01	0.80	0.20
revdiet3	0.00	-0.06	0.83	-0.01	0.67	0.33
smoking_1	0.78	0.12	-0.06	0.04	0.66	0.34
smoking_2	0.83	0.02	-0.01	-0.03	0.70	0.30
smoking_3	0.89	-0.02	-0.01	-0.07	0.80	0.20
smoking_4	0.59	0.10	0.07	0.18	0.43	0.57
revsmoke5	0.56	-0.20	0.17	-0.10	0.34	0.66

With (might not be included in the manuscript)

	**Factor3**	**Factor1**	**Factor4**	**Factor2**
**SS loadings**	3.00	2.96	2.33	1.80
**Proportion Var**	0.15	0.15	0.12	0.09
**Cumulative Var**	0.15	0.30	0.41	0.50
**Proportion Explained**	0.30	0.29	0.23	0.18
**Cumulative Proportion**	0.30	0.59	0.82	1.00

For peer review only

Modified ABCD Risk Questionnaire

Mark Bowyer, Hamid Hassen

Scale	Items	Coding
**Perceived Risk of Heart Attack or Stroke**	1. It is likely that I will have a heart attack or stroke sometime in my life	4= Strongly disagree, 3= Disagree, 2= Agree, 1= Strongly Agree; N/A= 0
	2. There is a good chance I will experience a heart attack or stroke in the next 10 years	4= Strongly disagree, 3= Disagree, 2= Agree, 1= Strongly Agree; N/A= 0
	3. It is (more) likely I will have a heart attack or stroke because of my past and/or present behaviours	4= Strongly disagree, 3= Disagree, 2= Agree, 1= Strongly Agree; N/A= 0
	4. I am not worried that I might have a heart attack or stroke	**REVERSE CODED** 4= Strongly disagree, 3= Disagree, 2= Agree, 1= Strongly Agree; N/A= 0
	5. I am concerned about the likelihood of having a heart attack or stroke in the near future	4= Strongly disagree, 3= Disagree, 2= Agree, 1= Strongly Agree; N/A= 0
**Perceived Benefits and Intentions to Exercise**	6. I am thinking about exercising at least 2.5 hours a week	4= Strongly disagree, 3= Disagree, 2= Agree, 1= Strongly Agree; N/A= 0
	7. I intend or want to exercise at least 2.5 hours a week	4= Strongly disagree, 3= Disagree, 2= Agree, 1= Strongly Agree; N/A= 0
	8. When I exercise for at least 2.5 hours a week I am doing something good for the health of my heart	4= Strongly disagree, 3= Disagree, 2= Agree, 1= Strongly Agree; N/A= 0
	9. I am confident that I can maintain a healthy weight by exercising at least 2.5 hours a week	4= Strongly disagree, 3= Disagree, 2= Agree, 1= Strongly Agree; N/A= 0
	10. I am not thinking about exercising for 2.5 hours a week	**REVERSE CODED** 4= Strongly disagree, 3= Disagree, 2= Agree, 1= Strongly Agree; N/A= 0
	11. Increasing my exercise to at least 2.5 hours a week will decrease my chances of having a heart attack or stroke	4= Strongly disagree, 3= Disagree, 2= Agree, 1= Strongly Agree; N/A= 0

For peer review only

**Perceived Benefit and Healthy Eating Intentions**	12. I am confident that I can eat at least five portions of fruit and vegetables a day within the next two months	4= Strongly disagree, 3= Disagree, 2= Agree, 1= Strongly Agree; N/A= 0
	13. I am thinking about eating at least five portions of fruit and vegetables a day	4= Strongly disagree, 3= Disagree, 2= Agree, 1= Strongly Agree; N/A= 0
	14. I am not thinking about eating at least five portions of fruit and vegetables a day	**REVERSE CODED** 4= Strongly disagree, 3= Disagree, 2= Agree, 1= Strongly Agree; N/A= 0
	15. When I eat five portions of fruit and vegetables a day I am doing something good for the health of my heart	4= Strongly disagree, 3= Disagree, 2= Agree, 1= Strongly Agree; N/A= 0
**Benefits and Intentions to Stop Smoking**	16. I am thinking of stopping smoking within two months	4= Strongly disagree, 3= Disagree, 2= Agree, 1= Strongly Agree; N/A= 0
	17. I have reduced or stopped smoking	4= Strongly disagree, 3= Disagree, 2= Agree, 1= Strongly Agree; N/A= 0
	18. I intend or want to stop smoking	4= Strongly disagree, 3= Disagree, 2= Agree, 1= Strongly Agree; N/A= 0
	19. If I stop smoking it will reduce my chances of having a heart attack or stroke	4= Strongly disagree, 3= Disagree, 2= Agree, 1= Strongly Agree; N/A= 0
	20. I am not thinking about stopping smoking	**REVERSE CODED** 4= Strongly disagree, 3= Disagree, 2= Agree, 1= Strongly Agree; N/A= 0

For peer review only

Reporting checklist for cross sectional study.

Based on the STROBE cross sectional guidelines.

Instructions to authors

Complete this checklist by entering the page numbers from your manuscript where readers will find each of the items listed below.

Your article may not currently address all the items on the checklist. Please modify your text to include the missing information. If you are certain that an item does not apply, please write "n/a" and provide a short explanation.

Upload your completed checklist as an extra file when you submit to a journal.

In your methods section, say that you used the STROBE cross sectionalreporting guidelines, and cite them as:

von Elm E, Altman DG, Egger M, Pocock SJ, Gotzsche PC, Vandenbroucke JP. The Strengthening the Reporting of Observational Studies in Epidemiology (STROBE) Statement: guidelines for reporting observational studies.

For peer review only

		Reporting Item	Page Number
**Title and abstract**			
Title	#1a	Indicate the study's design with a commonly used term in the title or the abstract	1
Abstract	#1b	Provide in the abstract an informative and balanced summary of what was done and what was found	1

**Introduction**			
Background / rationale	#2	Explain the scientific background and rationale for the investigation being reported	3
Objectives	#3	State specific objectives, including any prespecified hypotheses	3
**Methods**			
Study design	#4	Present key elements of study design early in the paper	4
Setting	#5	Describe the setting, locations, and relevant dates, including periods of recruitment, exposure, follow-up, and data collection	4
Eligibility criteria	#6a	Give the eligibility criteria, and the sources and methods of selection of participants.	4
	#7	Clearly define all outcomes, exposures, predictors, potential confounders, and effect modifiers. Give diagnostic criteria, if applicable	6
Data sources / measurement	#8	For each variable of interest give sources of data and details of methods of assessment (measurement). Describe comparability of assessment methods if there is more than one group. Give information separately for for exposed and unexposed groups if applicable.	6
Bias	#9	Describe any efforts to address potential sources of	7

For peer review only

		bias	
Study size	#10	Explain how the study size was arrived at	7
Quantitative variables	#11	Explain how quantitative variables were handled in the analyses. If applicable, describe which groupings were chosen, and why	7
Statistical methods	#12a	Describe all statistical methods, including those used to control for confounding	7
Statistical methods	#12b	Describe any methods used to examine subgroups and interactions	7
Statistical methods	#12c	Explain how missing data were addressed	7
Statistical methods	#12d	If applicable, describe analytical methods taking account of sampling strategy	7
Statistical methods	#12e	Describe any sensitivity analyses	7
**Results**			
Participants	#13a	Report numbers of individuals at each stage of study—eg numbers potentially eligible, examined for eligibility, confirmed eligible, included in the study, completing follow-up, and analysed. Give information separately for for exposed and unexposed groups if applicable.	7
Participants	#13b	Give reasons for non-participation at each stage	7

For peer review only

Participants	#13c	Consider use of a flow diagram	n/a No drop-out
Descriptive data	#14a	Give characteristics of study participants (eg demographic, clinical, social) and information on exposures and potential confounders. Give information separately for exposed and unexposed groups if applicable.	7
Descriptive data	#14b	Indicate number of participants with missing data for each variable of interest	7
Outcome data	#15	Report numbers of outcome events or summary measures. Give information separately for exposed and unexposed groups if applicable.	7
Main results	#16a	Give unadjusted estimates and, if applicable, confounder-adjusted estimates and their precision (eg, 95% confidence interval). Make clear which confounders were adjusted for and why they were included	8
Main results	#16b	Report category boundaries when continuous variables were categorized	n/a Continuous variables not measured
Main results	#16c	If relevant, consider translating estimates of relative risk into absolute risk for a meaningful time period	n/a No measurement of risk
Other analyses	#17	Report other analyses done—e.g., analyses of	10

For peer review only

		subgroups and interactions, and sensitivity analyses	
**Discussion**			
Key results	#18	Summarise key results with reference to study objectives	12
Limitations	#19	Discuss limitations of the study, taking into account sources of potential bias or imprecision. Discuss both direction and magnitude of any potential bias.	12
Interpretation	#20	Give a cautious overall interpretation considering objectives, limitations, multiplicity of analyses, results from similar studies, and other relevant evidence.	12
Generalisability	#21	Discuss the generalisability (external validity) of the study results	13
**Other Information**			
Funding	#22	Give the source of funding and the role of the funders for the present study and, if applicable, for the original study on which the present article is based	1

Notes:

- 13c: n/a No drop-out
- 16b: n/a Continuous variables not measured
- 16c: n/a No measurement of risk The STROBE checklist is distributed under the terms of the Creative Commons Attribution License CC-BY. This checklist was completed on 08. June 2021

For peer review only

using https://www.goodreports.org/, a tool made by the EQUATOR Network in collaboration with Penelope.ai

For peer review only

BMJ Open

Psychometric evaluation of the 'Attitudes and Beliefs about Cardiovascular Disease (ABCD) Risk Questionnaire' with validation of a previously untested 'Intentions and Beliefs around Smoking' sub-scale.

Journal:	*BMJ Open*
Manuscript ID	bmjopen-2021-054532.R2
Article Type:	Original research
Date Submitted by the Author:	11-Aug-2022
Complete List of Authors:	Bowyer, Mark; Nottingham Trent University, Institute of Health and Allied Professions, School of Social Sciences<br>Hassen, Hamid; University of Antwerp, Family Medicine and Population Health, Faculty of Medicine and Health Services<br>Bastiaens, Hilde; University of Antwerp Faculty of Medicine and Health Sciences, Family Medicine and Population Health<br>Gibson, Linda ; Nottingham Trent University, Institute of Health and Allied Professions, School of Social Sciences
**Primary Subject Heading**:	Public health
Secondary Subject Heading:	Cardiovascular medicine, Smoking and tobacco
Keywords:	PUBLIC HEALTH, STATISTICS & RESEARCH METHODS, PREVENTIVE MEDICINE

SCHOLARONE™
Manuscripts

I, the Submitting Author has the right to grant and does grant on behalf of all authors of the Work (as defined in the below author licence), an exclusive licence and/or a non-exclusive licence for contributions from authors who are: i) UK Crown employees; ii) where BMJ has agreed a CC-BY licence shall apply, and/or iii) in accordance with the terms applicable for US Federal Government officers or employees acting as part of their official duties; on a worldwide, perpetual, irrevocable, royalty-free basis to BMJ Publishing Group Ltd ("BMJ") its licensees and where the relevant Journal is co-owned by BMJ to the co-owners of the Journal, to publish the Work in this journal and any other BMJ products and to exploit all rights, as set out in our licence.

The Submitting Author accepts and understands that any supply made under these terms is made by BMJ to the Submitting Author unless you are acting as an employee on behalf of your employer or a postgraduate student of an affiliated institution which is paying any applicable article publishing charge ("APC") for Open Access articles. Where the Submitting Author wishes to make the Work available on an Open Access basis (and intends to pay the relevant APC), the terms of reuse of such Open Access shall be governed by a Creative Commons licence – details of these licences and which Creative Commons licence will apply to this Work are set out in our licence referred to above.

Other than as permitted in any relevant BMJ Author's Self Archiving Policies, I confirm this Work has not been accepted for publication elsewhere, is not being considered for publication elsewhere and does not duplicate material already published. I confirm all authors consent to publication of this Work and authorise the granting of this licence.

For peer review only

TITLE PAGE

Psychometric evaluation of the 'Attitudes and Beliefs about Cardiovascular Disease (ABCD) Risk Questionnaire' with validation of a previously untested 'Intentions and Beliefs around Smoking' sub-scale.

Mark Bowyer, Nottingham Trent University (Corresponding Author)

mark.bowyer@ntu.ac.uk **(ORCID** 0000-0002-1474-5711)

Nottingham Trent University School of Social Sciences

Chaucer Building, Burton Street

Nottingham NG1 4BT

Tel: (+1) 7786 993405 Fax: (+1)115 8485574

Hamid Yimam Hassen, University of Antwerp, Belgium

hamid.hassen@uantwerpen.be **(ORCID** 0000-0001-6485-4193)

Dr Hilde Bastiaens (Participating Investigator)
Associate professor
Dept Family Medicine and Population Health
Faculty of Medicine and Health Sciences
University of Antwerp

Tel: 0032 (0)3 265.29.10 Fax: 0032 (0)3 265.25.26 Hilde.bastiaens@uantwerpen.be

Dr Linda Gibson (Participating Investigator)

Professor in Public Health, Institute of Health & Allied Professions

Nottingham Trent University Linda.gibson@ntu.ac.uk

Key words

Cardiovascular Diseases

- Cardiovascular risk factors
- Instrumentation

Psychometrics

- Surveys and questionnaires
- Instrumentation

Primary prevention

- Instrumentation

Word count 4440

For peer review only

Psychometric evaluation of the 'Attitudes and Beliefs about Cardiovascular Disease (ABCD) Risk Questionnaire' with validation of a previously untested 'Intentions and Beliefs around Smoking' sub-scale.

ABSTRACT

Objectives:

To provide evidence of validity, reliability and generalisability of results obtained using the Attitudes and Beliefs about Cardiovascular Disease (ABCD) Risk Questionnaire with a sample of the English population surveyed within the 'SPICES' Horizon 2020 project (Nottingham study site), and to specifically evaluate the psychometric and factor properties of an as-yet untested 5 item sub-scale relating to smoking behaviours.

Design and setting:

Community and workplace-based cross-sectional study in Nottingham, UK.

Participants:

466 English adults fitting inclusion criteria (aged 18+, without known history of CVD, not pregnant, able to provide informed consent) participated in the study.

Methods:

We re-validated the ABCD questionnaire on a sample of the general population in Nottingham to confirm the psychometric properties. Furthermore, we introduced 5 items related to smoking which were dropped in the original study due to inadequate valid samples.

Primary and secondary outcome measures:

1. Psychometric and factor performance of untested 5 item 'smoking behaviours' sub-scale
2. Psychometric and factorial properties in combination with the remaining 18 items across 3 sub-scales

Results:

Analyses of the data largely confirmed the validity, reliability, and factor structure of the original ABCD Risk Questionnaire. Sufficient participants in our study provided data against an additional five smoking related items to confirm their validity as a sub-scale and to advocate for their inclusion in future applications of the scale. EFA and CFA calculations support some minor changes to the remaining sub-scales which may further improve psychometric performance and therefore generalisability of the instrument.

Conclusions:

An amended version of the ABCD Risk Questionnaire would provide public health researchers and practitioners with a brief, easy to use, reliable and valid survey tool. The amended tool may assist public health practitioners and researchers to survey patient or public intentions and beliefs around three key areas of individually modifiable risk (Physical Activity, Diet, Smoking).

For peer review only - http://bmjopen.bmj.com/site/about/guidelines.xhtml

Trial registration:

ISRCTN68334579 https://doi.org/10.1186/ISRCTN68334579
Heart health without a doctor: an implementation study of CVD prevention and behaviour change interventions in community settings

Ethical approval

Ethical approval for the 'SPICES' Nottingham study protocol (incorporating the ABCD Risk Questionnaire) was secured from the Nottingham Trent University College of Business, Law and Social Sciences on the 20th February 2019. Participants were required to provide informed consent (Appendix 1).

Article summary

Strengths and Limitations of this study

- Large sample (n=466) of English adults from the Nottingham UK population
- Sufficient case data to validate additional sub-scale related to attitudes and intentions of smokers
- Criterion validity not explored
- Full assessment of the utility of ABCD Risk Questionnaire in health promotion and CVD prevention not explored; further studies may be required to position the tool in clinical and public health practice.
- The planned pre-post intervention measurement and analysis was not possible due to COVID-19 interruption of fieldwork.

Original protocol (Appendix 2)

Funding statement

This work was supported by the European Commission Horizon 2020 Non-communicable diseases and the challenge of healthy ageing Grant agreement 733356 'SPICES'.

Competing interests statement

None declared

Patient and public involvement

Patients and/or the public were not involved in the design, or conduct, or reporting, or dissemination plans of this research.

Patient consent for publication (data sharing agreement)

Not required (participant information and informed consent attached Appendix 1)

Provenance and peer review

Not commissioned.

Data availability statement

Data are available on reasonable request

For peer review only
For peer review only - http://bmjopen.bmj.com/site/about/guidelines.xhtml

Keywords

Cardiovascular diseases- Cardiovascular risk factors

Cardiovascular diseases- Instrumentation

Psychometrics- Instrumentation

Surveys and questionnaires- Instrumentation

Primary prevention- Instrumentation

Author contributions

Following ICMJE recommendations, Mark Bowyer and Hamid Hassen assert authorship based on the following 4 criteria:

Substantial contributions to the conception or design of the work; or the acquisition, analysis, or interpretation of data for the work; AND

Drafting the work or revising it critically for important intellectual content; AND

Final approval of the version to be published; AND

Agreement to be accountable for all aspects of the work in ensuring that questions related to the accuracy or integrity of any part of the work are appropriately investigated and resolved.

Professor Linda Gibson and Professor Hilde Bastiaens assert Participating Investigator status having served as scientific advisors, critically reviewed the study proposal, and participated in writing or technical editing of the manuscript.

Acknowledgements

The authors would like to acknowledge the cooperation of Rolls-Royce plc Hucknall Site; Nottingham City Council Adult Care in providing access to employees. Crabtree Farm Community Centre, Middle Street Resource Centre, Self-Help UK, in facilitating access to members, users and premises.

INTRODUCTION

Scientific Background and Rationale

In the UK, Cardiovascular Disease (CVD) is responsible for over 130,000 deaths per annum.[1] CVD morbidity is also the biggest contributor to the inequalities in healthy life expectancy between members of the wealthiest neighbourhoods and the most deprived.[2] In 2009 the NHS Health Check [3]was established and more recently (2019) the CVD Prevent initiative to implement ‘upstream’ interventions for the prevention of CVD morbidity.[4] Both of these initiatives seek to

For peer review only - http://bmjopen.bmj.com/site/about/guidelines.xhtml

For peer review only

improve early case-finding to prevent avoidable strokes and heart attacks. Both recognise the importance of supported lifestyle change in conjunction with drug therapies.

Lifestyle or behavioural change requires a degree of individual agency and commitment which drug therapies do not. Unhealthy lifestyle behaviours are linked to culture and habit, environment, emotions, and confidence which can all moderate an individual's readiness to change and the commitment required to sustain those changes over time.[5] Understanding the attitudes and beliefs that people hold towards diet, exercise and smoking, as well as their perception of their own risk could assist primary care and public health professionals in providing relevant and effective behavioural advice and social prescribing options. To support evaluations of the NHS Health Check programme, in 2017 a questionnaire was developed to evaluate patients' awareness of cardiovascular disease risk at University College London.[6] This ABCD Risk Questionnaire attempts to provide a short survey drawing from the dominant theoretical models of behaviour change (Trans-Theoretical Model, Health Beliefs Model),[7] covering diet, smoking, exercise and alcohol behaviours, and incorporating a conceptual spread of perceived risk from immediate to lifetime. Whilst a range of validated CVD risk questionnaires already exist,[8] and it is common to ask patients to self-report their physical activity, dietary and smoking behaviours through questionnaires and diaries, the ABCD Risk Questionnaire usefully investigates the knowledge, perceptions, beliefs and attitudes that govern these behaviours. To confirm the reliability and generalisability of the ABCD Risk Questionnaire, it was necessary to replicate the study with a new, larger independent dataset.

Specific Objectives

In this study we re-validated the tool on a sample of the general population in Nottingham to confirm the psychometric properties. Furthermore, we introduced 5 items related to smoking which were dropped in the original study due to inadequate case numbers.

To the best of our knowledge, this is the first study which has incorporated items relating to attitudes and intentions towards stopping smoking into the published version of the ABCD Risk Questionnaire and collected sufficient data to submit them to analysis of validity, reliability and factor structure.

In the original ABCD study, over the course of three stages of validity testing (content, face, reliability) items relating to alcohol use and smoking were rejected, leaving four final sub-scales: Knowledge of CVD Risks; Perceived Risk of Heart Attack/ Stroke; Perceived Benefits and Intentions to Change; and Healthy Eating Intentions. During Exploratory Factor Analysis (EFA) none of the items relating to alcohol use achieved strong enough loadings to be included in the final scale, and items related to smoking could not be included due to the high proportion of missing data in the experimental sample. The authors of the study note this limitation '*the questionnaire does not encompass all aspects of CVD risk observed in the general population*' and that '*future studies examining populations at increased CVD risk can look into incorporating smoking and alcohol into the ABCD Risk Questionnaire to learn about these individuals' preconceptions and attendance of follow-up care*'.[9]

The present study

Nottingham is one of five global sites of the EU Horizon 2020 'SPICES' [10] CVD prevention implementation study which began in 2017. SPICES investigates contextual and health system barriers to the scaling up of successful behaviour change interventions for improved cardiovascular health in low, middle and high income European countries. The most recent data (2016) indicate that "The prevalence of CVD recorded in Nottingham City GP Practices is significantly less that the

For peer review only - http://bmjopen.bmj.com/site/about/guidelines.xhtml

national (England) average and in comparable areas, despite the CVD mortality rate being significantly higher than average; this partly reflects the differing age structures of the populations, but also indicates significant under-detection/diagnosis"[11]

The SPICES Nottingham population survey carried out in 2019-20 utilised the ABCD Risk Questionnaire alongside the non-clinical INTERHEART CVD risk prediction instrument.[12] The SPICES study team chose to re-introduce 5 pre-written items relating to 'Intentions and Readiness to Stop Smoking' from the 65 item University College London (UCL) item pool into the questionnaire due to the high prevalence of smoking in the Nottingham population compared to England averages,[13] and its importance as a CVD risk.[14] This created a 31 item questionnaire. 4 items relating to Alcohol intake from the same item pool were also considered for inclusion but omitted on two grounds: alcohol related CVD risk was not a specific focus of the 'SPICES' study; concerns about the time-burden on participants of including the additional items which can be a barrier to participation.

In so doing, NTU researchers attempted to '*replicate the factor analytic process on an independent, larger sample to confirm the generalisability of (the original) findings*' as requested by the authors of the original study.[15] At the same time, we anticipated securing sufficient responses against the reintroduced 5 item 'smoking' sub-scale to analyse its reliability and validity as an integral part of future versions of the Questionnaire.

METHODS

Incorporating the ABCD Risk Questionnaire into the SPICES Nottingham baseline survey provided cross-sectional study data across a broad sample of adult participants. The data-set generated was therefore suitable for psychometric validation of the original and modified versions of the ABCD questionnaire. Surveys were administered in-person by researchers in the field during attendance at community venues and workplaces. Administration of the survey took approximately ten minutes including provision of consent, and confidential communication of results another ten minutes on average. Participation was entirely voluntary. The sample was checked for representativeness of the Nottingham population across parameters of age, gender, and household income (Appendix 3).

Participants

Participants were recruited from across the Nottingham conurbation between April 2019 and March 2020 as part of the SPICES Nottingham baseline survey.[16] A purposive sampling method was employed based on community engagement. This strategy had two components:

1. engagement of citizens in neighbourhoods through existing community groups, organisations and venues, and
2. engagement of employees in the workplace through large city-based employers.

Community groups were targeted on the basis of the demographic of their membership to ensure that neighbourhoods of differing mean household income, those who are not in employment or of working age, and those from different ethnicities were included. In this way 327 participants were recruited.

Employers were targeted on the basis of workforce size, and policies relating to workforce well-being. Nottingham City Council Adult Care teams and the Rolls-Royce plc Hucknall site both responded positively and between them provided 156 participants. NTU researchers administered the SPICES Nottingham baseline survey individually within the community or workplace setting and

For peer review only - http://bmjopen.bmj.com/site/about/guidelines.xhtml

personalised feedback about CVD risks was provided confidentially once the survey had been completed.

Criteria for inclusion included being aged 18+, resident in Nottinghamshire, not previously diagnosed with a heart condition, not pregnant, and able to provide informed consent.

Materials

The SPICES baseline survey incorporated the ABCD risk questionnaire into a digitised survey instrument created in the Research Electronic Data Capture (REDCap) database system,[17] a secure web application for building and managing online surveys and databases, and the online survey responses were uploaded automatically. No participant data was stored on local devices. Both the ABCD Risk Questionnaire (Table 1) and the non-laboratory INTERHEART questionnaire were included unchanged from their published versions apart from an additional 5 items pertaining to smoking behaviour (Table 2).[18]

Table 1. Published ABCD Risk Questionnaire

Scale	Items
**Knowledge**<br><br>True/False/Don't Know<br><br>Correct score =1<br>Incorrect/ Don't know score = 0<br><br>Higher sum score= more knowledgeable/ more correct about having a heart attack or stroke	1. One of the main causes of heart attack and stroke is stress
	2. Walking and gardening are considered types of exercise that can lower the risk of having a heart attack or stroke
	3. Moderately intense activity of 2.5 hours a week will reduce your chances of having a heart attack or stroke
	4. People who have diabetes are at higher risk of heart attack or stroke
	5. Managing your stress levels will help you to manage your blood pressure
	6. Drinking high levels of alcohol can increase your cholesterol and triglyceride levels
	7. HDL refers to 'good' cholesterol, and LDL refers to 'bad' cholesterol
	8. A family history of heart disease is not a risk factor for high blood pressure
**Perceived Risk of Heart Attack or Stroke**<br><br>4= Strongly disagree, 3= Disagree, 2= Agree, 1= Strongly Agree; N/A= 0<br><br>Higher sum score = higher perception of risk of having a heart attack or stroke	9. I feel I will suffer from a heart attack or stroke sometime during my life
	10. It is likely that I will suffer from a heart attack or stroke in the future
	11. It is likely that I will have a heart attack or stroke some time during my life
	12. There is a good chance I will experience a heart attack or stroke in the next 10 years
	13. My chances of suffering from a heart attack or stroke in the next 10 years are great
	14. It is likely I will have a heart attack or stroke because of my past and/or present behaviours
	15. I am not worried that I might have a heart attack or stroke (**Reverse coded**)
	16. I am concerned about the likelihood of having a heart attack or stroke in the near future

For peer review only - http://bmjopen.bmj.com/site/about/guidelines.xhtml

**Perceived Benefits and Intentions to Change**<br><br>4= Strongly disagree, 3= Disagree, 2= Agree, 1= Strongly Agree; N/A= 0<br><br>Higher average score = Higher perceived benefits of diet and exercise and higher perceived readiness for change in regards to exercise and behaviour	17. I am thinking about exercising at least 2.5 hours a week
	18. I intend or want to exercise at least 2.5 hours a week
	19. When I exercise for at least 2.5 hours a week I am doing something good for the health of my heart
	20. I am confident that I can maintain a healthy weight by exercising at least 2.5 hours a week
	21. I am not thinking about exercising for 2.5 hours a week **(Reverse coded)**
	22. When I eat five portions of fruit and vegetables a day I am doing something good for the health of my heart
	23. Increasing my exercise to at least 2.5 hours a week will decrease my chances of having a heart attack or stroke
**Healthy Eating Intentions**<br><br>4= Strongly disagree, 3= Disagree, 2= Agree, 1= Strongly Agree; N/A= 0<br><br>Higher average score = Higher perceived readiness for change with regard to healthy dietary behaviour	24. I am confident that I can eat at least five portions of fruit and vegetables a day within the next two months
	25. I am thinking about eating at least five portions of fruit and vegetables a day
	26. I am not thinking about eating at least five portions of fruit and vegetables a day **(Reverse coded)**

The surveys were administered in the field by a team of trained researchers recruited from the NTU student body and directly supervised by the SPICES Nottingham coordinator. The surveys were accessed using dedicated tablet computers. Items were reproduced word for word and in the same sequence as the original ABCD Risk Questionnaire with the additional 5 smoking items inserted after all 26 original items. The five smoking related items were developed by the authors of the original study through a process of literature review (construct validity), expert panel review (content validity), and modification by focus group (face validity). These five smoking sub-scale items were included in the 65 item pool developed in the original study but omitted from their analysis due to a high proportion of missing responses.

Table 2. Additional ‘smoking’ sub-scale

**Benefits and Intentions to Stop Smoking**<br><br>4= Strongly disagree, 3= Disagree, 2= Agree, 1= Strongly Agree; N/A= 0<br><br>Higher average score = Higher perceived readiness for change with regard to healthy dietary behaviour	27. I am thinking of stopping smoking within two months
	28. I have reduced or stopped smoking
	29. I intend or want to stop smoking
	30. If I stop smoking it will reduce my chances of having a heart attack or stroke
	31. I am not thinking about stopping smoking

For peer review only

For peer review only - http://bmjopen.bmj.com/site/about/guidelines.xhtml

Data analysis

We took the published 26-item ABCD Risk Questionnaire, introduced 5 further items relating to smoking behaviours, and administered it alongside a validated CVD risk assessment instrument (INTERHEART) to 486 individuals in Nottingham over a period of 12 months. Item, scale, and factor reliabilities were computed to generate a comparison to the results reported in the original study. Correlation was tested between and amongst ABCD sub-scale scores and selected INTERHEART variables, closely matching the methods applied in the original study (Appendix 4) and results were compared accordingly. After removing incomplete responses, 466 valid cases were entered for analysis, four times the sample size of the original study.

Item and sub-scale reliabilities were tested using inter-item correlations, corrected item-total correlations and Cronbach's Alpha. [19] We performed an exploratory factor analysis (EFA) to evaluate the dimensionality of items of the original and modified risk scale with and without the smoking items.[20] The EFA was performed using the maximum likelihood extraction and varimax rotation method. [21] Sample and data adequacy was assessed using Kaiser-Meyer-Olkin (KMO) test and Bartlett's test of sphericity was performed to compare an observed correlation matrix to the identity matrix.[22] The adequate number of factors was determined using a scree plot (Appendix 5). To further test the consistency of factors, we tested using Confirmatory Factor Analysis (CFA). We evaluated the model fit of the CFA using; the X2 test, the Tucker-Lewis and Comparative Fit Indexes and the root mean square error of approximation (RMSEA).[23] The analysis was performed using a free statistical software R version 4.0.2. UK postcodes were collected for all participants which allowed them to be sorted into income deciles using Office for National Statistics Index of Multiple Deprivation (IMD) public datasets,[24] allowing correlations to be analysed. Following the methods used in the original study, case data from the 'Knowledge' sub-scale (8 items) were omitted from the analysis since they utilise a separate response format.

We used the STROBE cross sectional checklist when writing our report.[25]

RESULTS

Participants

Participation was voluntary, and self-selection may have been influenced by sensitivities around disclosure of health status and lifestyle habits forming a barrier to those with co-morbidities and socially 'questionable' behaviours (heavy smoking, high alcohol intake).

The sample cohort has a 49:51 percent gender split, normal distribution of age ranges (18-92), and a distribution of Socio-Economic Status (SES) which reflects known data about neighbourhood income in Nottingham. Nottingham is the 11th most deprived district in England with higher unemployment, lower education and skills, and shorter life expectancy than the national averages. [26] Using the Index of Multiple Deprivation a relative measure of deprivation across seven domains, Health and Disability is the domain on which the city's scores are lowest. Nevertheless, the mean INTERHEART predicted risk score for all 466 participants was 10.32 which closely matches the global reported mean for the instrument.[27]

Smoking sub-scale

The percentage of smokers in our sample was 15.5%. The number of smokers in our sample was therefore higher than the 2019 England average (13.9%),[28] and lower than the Nottingham city

For peer review only - http://bmjopen.bmj.com/site/about/guidelines.xhtml

population average (20.6%) based on the ONS Annual Population Survey.[29] ONS notes that smoking prevalence estimates by local authority can fluctuate due to smaller sample sizes. Our SPICES Nottingham sample cohort also includes some participants from neighbouring Local Authorities with different recorded rates of smoking.

The five items in the smoking subscale are measured on the same four-point response scale as the 18 items submitted for Factor Analysis in the original published ABCD Risk Questionnaire (Strongly agree, agree, disagree, strongly disagree, and not applicable).

With the original 18 items this 'Not Applicable' response option was not used by any of the SPICES Nottingham study participants. By contrast, within their responses to the items in the 'smoking' subscale, 'Not Applicable' was the modal answer. Participants chose the 'N/A' response option whenever they reported being a non-smoker. This mirrors the behaviour of the original 110 NHS Health Check attendees who formed the pilot sample cohort for the original study, leaving an insufficient number of cases to assess validity and reliability of smoking sub-scale items. In the present study, 88 cases were found where participants reported smoking behaviours and this was sufficient to enter them into analysis.

Sub-scale Alpha values, Cronbach's Alpha if item deleted calculated for all items, inter-item correlations and corrected item-total correlations were all calculated, mirroring the analysis reported in the original study (Appendix 6).

Interitem correlations calculated for these five items produced a range between 0.654 and 0.834. All of these five 'smoking' items therefore correlate with one another more strongly than recommended (<.6) and were considered for rejection. However, we found each item to be qualitatively different, and that the differences were conceptually clear and well expressed in the item wording so that no participant could be expected to confuse one with any other, and they were retained.

Discrimination was confirmed using item-total correlations. These fell between the range 0.751 and 0.906 meaning that all five 'smoking' sub-scale items are comfortably above the standard cut-off for acceptability of 0.3.

EFA was carried out twice, firstly with all cases, and then again with 88 confirmed smoking cases. The first operation ensured that factor loadings were not skewed by the lower number of cases reporting smoking behaviours, the second ensured that factor loadings for the remaining sub-scales where more case data was available were not skewed by outliers.

Exploratory Factor Analysis:

We conducted EFA on the original 18-item risk perception questionnaire and the modified 23-item (with smoking items). For the original 18-item, a total of 420 observations were included in the analysis, which was sufficient for factor analysis as indicated with KMO of 0.82, which is within the recommended range (0.8 to 1). The Bartlett's Test of Sphericity was significant (X2 = 4235.007, p-value < 0.001) indicating the data is adequate for factor analysis. As a result, a three-factor solution emerged based on the Scree plot (figure 1), accounting 57.4% of the total variance. Factor loading patterns in the present analysis slightly varied from the original subscales. The domains in the original subscales were risk perception, benefit finding and healthy eating intentions. In our analysis, Item 14 ('*When I eat at least 5 portions of fruit and vegetables a day I am doing something good for the health of my heart*') showed a better loading to healthy eating intention, which was loaded to benefit finding in the original study (Appendix 5).

For peer review only - http://bmjopen.bmj.com/site/about/guidelines.xhtml

For the modified 23-item (including the smoking sub-scale), 88 samples were valid and included in the analysis. The KMO was 0.78, which was slightly below the recommended range, but Bartlett's Test of Sphericity was significant (X2 = 1223.459, p-value < 0.001), indicating adequacy for factor analysis. The analysis showed that the smoking items loaded to another latent construct resulting in four factors in total (figure 2).

For peer review only

Confirmatory Factor Analysis of the published ABCD Risk Questionnaire

In the original study of 2017, 18 items were entered into factor analysis. A Confirmatory Factor Analysis tests the fit of these original items to their structure using the larger Nottingham SPICES dataset. Conducting CFA allowed us to construct the sub-scales of the published ABCD Risk Questionnaire in a three-factor measurement model and test its fit against relevant indices. Original 18 item survey comprising three sub-scales (Perceived Risk of Heart Attack/Stroke 8 items; Perceived Benefits and Intentions to Change 7 items; Healthy Eating Intentions 3 items) were used to create measurement model in SPSS Amos 25. In the original study of 2017, items relating to smoking behaviours were developed but could not be included in the published scale due to insufficient data. In the Nottingham SPICES study sufficient observations were made to test these smoking items.

Editing the measurement model

As discussed above, independent item analysis and Exploratory factor Analysis using the independent SPICES Nottingham dataset revealed issues with the continued inclusion of some of the original 'perception of risk' sub-scale items, and the allocation of an item relating to dietary behaviours in the physical activity behaviours sub-scale. The published ABCD questionnaire was then reconstructed to remove items which had confused participants and generated high inter-item correlations, and additionally to re-assign an item relating to dietary behaviour into the dietary behaviour sub-scale. This resulted in a four-factor model (Perceived Risk of Heart Attack/ Stroke' 6 items; 'Perceived Benefits and Intentions to Exercise' 6 items; 'Healthy Eating Intentions' 4 items, Perceived Benefits and Intentions to Reduce Smoking' 5 items).

Finally, the CFA measurement model was modified so that items within each ABCD sub-scale were set to co-vary with one another. Analysis properties were set to Estimation: Maximum Likelihood.

Selection of fit indices

Commonly used model-data fitting indices were employed taking into account sample size and number of variables. Absolute fit was tested using Root Mean Square Error of Approximation

For peer review only - http://bmjopen.bmj.com/site/about/guidelines.xhtml

(RMSEA) where a value of .6 or less is indicative of good model fit; Root Mean Square Residual (RMR) where a value of .8 or less indicates good model fit.[30] Relative fit was tested using the Tucker-Lewis Index (TLI) and Comparative Fit Index (CFI) where good fit is indicated by a value of .95 or more.[31,32] We have also reported the Minimum Discrepancy Function by Degrees of Freedom (CMIN/DF) where good fit is indicated by values below .3.[33] Results are presented in Table 3.

Table 3. CFA fit indices for the original and modified ABCD Questionnaire measurement models

**Original 18 item ABCD**						
CMIN	P	CMIN/DF	TLI	CFI	RMSEA	RMR
714.941	.000	5.416	.826	.850	.097	.049
**Original 18 item ABCD with 5 Smoking items added**						
CMIN	P	CMIN/DF	TLI	CFI	RMSEA	RMR
994.931	.000	4.442	.865	.881	.086	.049
**Edited 20 item ABCD with Smoking sub-scale**						
CMIN	P	CMIN/DF	TLI	CFI	RMSEA	RMR
638.973	.000	3.896	.881	.897	.079	.052
**Modified 20 item ABCD with Smoking sub-scale**						
CMIN	P	CMIN/DF	TLI	CFI	RMSEA	RMR
385.312	.000	2.439	.941	.951	.056	.046

In the 23-item factor analysis, item 14 was loaded to the healthy eating intention. The model fit indices showed a slight improvement as indicated in table 3.

Based on factor loading, inter-item correlations, and face validity results, we also tested a slightly shorter version of the questionnaire, 20-items including five smoking items and the result shows that the model fit improved (CFI=0.941; TLI=0.951; RMSEA=0.056, SRMR=0.046).

The three published factors achieved a poor fit in CFA. Including the five smoking related items which had performed strongly in EFA as their own latent factor improved overall model fit slightly, but not to an acceptable level.

Modification of the measurement model

Reviewing modification indices and expected parameter changes for factor loadings and measurement intercepts we observed an extreme covariance value (116.812) and parameter change (.209) between two of the risk perception items ('there is a good chance that I will experience a heart attack or stroke in the next 10 years' and 'my chances of suffering a heart attack or stroke in the next 10 years are great') which had caused confusion for participants in our study.

Removing one of these two items (item #13), and the two other duplicative items (items #9 & #10) from the 'perceived risk of heart attack or stroke' sub-scale retains the conceptual spread of risk embodied by the items (lifetime, 10 year, near future, behaviour related). Moving the diet related item (#22) which appears in the 'perceived benefits and intentions to change' over to the 'healthy eating intentions' sub-scale might allow greater clarity for researchers analysing results from the

For peer review only - http://bmjopen.bmj.com/site/about/guidelines.xhtml

questionnaire. Co-varying items within sub-scales that generated values above 20 (a high cut-off due to large sample used) resulted in acceptable or good fit across all sub-scales. Each of the three behaviour related sub-scales now contain items drawn from HBM, TTM and SE models providing a sound conceptual basis for comparison. Using EFA to check these results shows the modified sub-scale structure performs better than the published version (all EFA results Appendix 5).

DISCUSSION

Inadequate knowledge and/or a gap between perceived and actual CVD risk in the population could be an obstacle to better health outcomes. Improving an individual's CVD knowledge and risk perception may be important in improving a healthy lifestyle. Measuring CVD knowledge and risk perception may be a method to initiate a healthy lifestyle intervention as well as to monitor and evaluate the impact of interventions. Following this rationale, Woringer and colleagues developed the ABCD Risk questionnaire in order to measure CVD knowledge and risk perception. In this study, we re-validated the tool on a sample of the general population in Nottingham to confirm the psychometric properties.

The 88 participants in this study who reported smoking is a low number for pilot testing of psychometric scales, but it does exceed a 10:1 ratio of cases to variables making it reasonable to proceed to analysis.

Based on EFA and CFA, we confirmed a three-factor structure, which closely matched the results reported in the original study but differed in certain important respects. Item 14 ('*When I eat at least 5 portions of fruit and vegetables a day I am doing something good for the health of my heart*") showed a better loading to the 'healthy eating intentions' sub-scale, in contrast to the factor loading in the original study, which placed this item in 'perceived benefits and intentions to change'. This is the only item which loaded onto a different sub-scale when using the Nottingham dataset, all others continued to load onto their original factors although many of these loaded weakly and failed to meet usual thresholds for validity (Appendix 5). The larger numbers of participants in our dataset (466 compared to 110) provides statistical confidence in the new results, and we therefore modelled this revised allocation of items and factors alongside the original factor allocations in the subsequent Confirmatory Factor Analysis. The revised measurement model with item 14 allocated to 'Healthy Eating Intentions' indicated a better fit in CFA results.

These results suggest that the additional five smoking items perform acceptably and should be incorporated into future applications of the ABCD Risk Questionnaire.

We believe that psychometric performance based on reliability calculations and factorial analysis is not an end in itself. The resulting scale has to have some utility in the world and generate results which can add value to existing understanding of beliefs and attitudes to cardiovascular disease. This is only very lightly touched on in the original paper which states that 'the questionnaire can be used to assess patients' understanding of CVD risk'. We believe that because there is a recognised gap between 'knowing' and 'doing' in relation to CVD risk factors which means that much health education may be failing to stimulate the healthy changes in the population, it is important to consider the attitudes and beliefs about elective change in relation to risky lifestyle behaviours which may be mediating this relationship. If it is not enough simply to educate vulnerable people to the nature of the risks in order to stimulate the necessary changes to reduce CVD risk, then although socio-economic factors will also

For peer review only - http://bmjopen.bmj.com/site/about/guidelines.xhtml

play a part here, and there may be additional psychological factors (such as 'present-bias') which also mediate this space, the ABCD Risk Questionnaire goes a long way to investigating and measuring the personal beliefs and attitudes which operate in this space.

Other observations

Researchers in the Nottingham SPICES team administering the questionnaire during fieldwork reported that three items within the 'Perception of Risk of Heart Attack/Stroke' sub-scale caused consistent difficulties for respondents due to apparent duplication and confusion over fine semantic differences. It was difficult for participants to see a semantic difference between statements 9, 10, 11, and 12, 13 respectively. For items 9, 10, and 11, if we agree that *suffer from* and *have* are synonymous, it is hard to differentiate between *in the future* and *some time during my life* because you would imagine that respondents will be thinking about the future in both cases.

For the questionnaire to be reliable across all sections of the population, including those with limited ability in English (whether native or non-native, first, second or additional language, etc.) who may find it particularly hard to differentiate with any confidence between different pairs/sets of statements with largely synonymous meanings, this confusion is a problem. Items 12 and 13 seem to differ mainly only in the possible interpretation of a difference of degree between *good* and *great.*

These face validity issues and their impact can be observed in the inter-item correlation results generated during item reliability analysis. In the original study, two items in the perception of risk sub-scale had been rejected due to correlations in excess of 0.6 leaving 8 items. Of these remaining 8 items half had inter-item correlations which exceeded 0.6 when tested against the Nottingham dataset. These were items 9, 10, 11, and 12 which generated inter-item correlation values of .832, .869, .616, and .729 respectively. Removing items 9, 10, and 13 does not reduce the conceptual range of the 'perception of risk' subscale which is framed temporally from immediate threat to lifetime risk, it simply removes the duplicate or confusing items. Testing this shortened scale with factor analysis strengthens both item and scale reliability and improves factor loadings (Appendix 5). We recommend that future versions of the English language ABCD Risk Questionnaire adopt these edits (Appendix 7).

CONCLUSIONS

The published English language version of the ABCD Risk Questionnaire, with the removal of three problematic 'perception' items, the shift of one item from the 'perceived benefits and intentions to change' sub-scale into the 'healthy eating intentions' sub-scale, and the addition of a 5 item 'smoking' sub-scale performs sufficiently well in validity, reliability and factor analysis with an independent, larger sample to confirm the generalisability of its original published findings. This result supports continued use of the ABCD Risk Questionnaire in the field of CVD prevention research and practice. The inclusion of a smoking behaviours sub-scale is likely to increase its relevance where smoking behaviours still account for a large proportion of individually modifiable CVD risk in a target population. Although criterion validity has now been established for the 'Perception of risk of heart attack/stroke sub-scale' by two published studies, the utility of the remaining sub-scales individually or in combination has been under-examined. Future studies should investigate the criterion validity of these sub-scales and the conceptual strength of the items and variables from which they have been composed in order to unambiguously position the resulting survey instrument and evaluate its utility in CVD prevention and treatment practices. Neither this study or the original published study of 2017 were able to conduct pre-post intervention

For peer review only - http://bmjopen.bmj.com/site/about/guidelines.xhtml

measurements in their study design. Measuring using the ABCD survey before an intervention (such as the NHS Health Check) and then again at some time afterwards- in tandem with a validated CVD risk prediction scale (such as INTERHEART or Q Risk 2) would help to establish the ABCD Risk Questionnaire's sensitivity to change, and perhaps also its ability to discern between types of respondent.

REFERENCES

1 https://www.bhf.org.uk/bhf-cvd-statistics-uk-factsheet.pdf (Accessed 10/05/2021)

2 https://www.england.nhs.uk/ourwork/clinical-policy/cvd/ (Accessed 10/05/2021)

3 Robson J, Dostal I, Sheikh A*, et al* The NHS Health Check in England: an evaluation of the first 4 years *4 BMJ Open* 2016;**6:**e008840. doi: 10.1136/bmjopen-2015-008840

Kearney M Prevention and treatment of CVD: a new priority for the NHS *Heart* 2019;**105:**1924.

5 Michie, S., van Stralen, M.M. & West, R. The behaviour change wheel: A new method for characterising and designing behaviour change interventions. *Implementation Sci* **6,** 42 (2011). https://doi.org/10.1186/1748-5908-6-42

6 Woringer M, Nielsen JJ, Zibarras L*, et al* Development of a questionnaire to evaluate patients' awareness of cardiovascular disease risk in England's National Health Service Health Check preventive cardiovascular programme *BMJ Open* 2017;**7:**e014413. doi: 10.1136/bmjopen-2016-014413

7 Health Behavior: Theory, Research, and Practice edited by Karen Glanz, Barbara K. Rimer, K. Viswanath

8 Gorenoi, V. and Hagen, A. Overview of risk-estimation tools for primary prevention of cardiovascular diseases in European populations. *Central European journal of public health 2015 23*(2), p.91.

9 Woringer M, Nielsen JJ, Zibarras L*, et al* IBID

10 Nahar, P., van Marwijk, H., Gibson, L. *et al.* A protocol paper: community engagement interventions for cardiovascular disease prevention in socially disadvantaged populations in the UK: an implementation research study. *glob health res policy* **5,** 12 (2020). https://doi.org/10.1186/s41256-020-0131-1

11 https://www.nottinghaminsight.org.uk/themes/health-and-wellbeing/joint-strategic-needs-assessment/adults/cardiovascular-disease-2016/ (accessed 29/11/21)

12 Yusuf S , Hawken S , Ounpuu S , *et al* . Effect of potentially modifiable risk factors associated with myocardial infarction in 52 countries (the INTERHEART study): case-control study. Lancet 2004;**364**:937–52.doi:10.1016/S0140-6736(04)17018-9

13 https://www.nottinghaminsight.org.uk/themes/health-and-wellbeing/joint-strategic-needs-assessment/adults/smoking-and-tobacco-control-2019/ (accessed 29/11/21)

14 John A Ambrose, Rajat S Barua The pathophysiology of cigarette smoking and cardiovascular disease: An update, Journal of the American College of Cardiology, Volume 43, Issue 10, 2004, Pages 1731-1737, ISSN 0735-1097,https://doi.org/10.1016/j.jacc.2003.12.047

15 Woringer M, Nielsen JJ, Zibarras L*, et al* IBID

16 Nahar, P., van Marwijk, H., Gibson, L. *et al.* IBID

17 Harris, P.A., et al., Research electronic data capture (REDCap)—A metadata-driven methodology and workflow process for providing translational research informatics support. Journal of Biomedical Informatics, 2009. **42**(2): p. 377-381 DOI: https://doi.org/10.1016/j.jbi.2008.08.010.

18 McGorrian C, Yusuf S, Islam S, Jung H, Rangarajan S, Avezum A, et al. Estimating modifiable coronary heart disease risk in multiple regions of the world: the INTERHEART Modifiable Risk Score. European Heart Journal. 2011;32(5):581-9. doi: 10.1093/eurheartj/ehq448.

19 Cronbach, L.J. Coefficient alpha and the internal structure of tests. *Psychometrika* **16,** 297–334 (1951). https://doi.org/10.1007/BF02310555

20 Ferguson, E. and Cox, T. (1993), Exploratory Factor Analysis: A Users'Guide. International Journal of Selection and Assessment, 1: 84-94. https://doi.org/10.1111/j.1468-2389.1993.tb00092.x

21 Kaiser, H.F. The varimax criterion for analytic rotation in factor analysis. Psychometrika 23, 187–200 (1958). https://doi.org/10.1007/BF02289233

22 Bartlett, M. S. (1951). The effect of standardization on a Chi-square approximation in factor analysis. Biometrika, 38(3/4), 337-344

For peer review only - http://bmjopen.bmj.com/site/about/guidelines.xhtml

23 Hu Lt, Bentler PM. Cutoff criteria for fit indexes in covariance structure analysis: Conventional criteria versus new alternatives. Structural Equation Modeling: A Multidisciplinary Journal. 1999;6(1):1-55. doi: 10.1080/10705519909540118.
24 Ministry of Housing, Communities & Local Government. The English indices of deprivation 2019 statistical release https://www.gov.uk/government/statistics/english-indices-of-deprivation-2019 (accessed 10/05/2021)
25 von Elm E, Altman DG, Egger M, Pocock SJ, Gotzsche PC, Vandenbroucke JP. The Strengthening the Reporting of Observational Studies in Epidemiology (STROBE) Statement: guidelines for reporting observational studies.
26 UK Government Office for National Statistics 2019 https://www.ons.gov.uk/peoplepopulationandcommunity/healthandsocialcare/healthandlifeexpectancies/bulletins/adultsomkinghabitsgreatbritain/2018 (Accessed 10/05/2021)
27 Yusuf S , Hawken S , Ounpuu S , *et al* **IBID**
28 UK Government Office for National Statistics 2019 https://www.ons.gov.uk/peoplepopulationandcommunity/healthandsocialcare/healthandlifeexpectancies/bulletins/adultsomkinghabitsgreatbritain/2018 (Accessed 10/05/2021)
29https://www.ons.gov.uk/peoplepopulationandcommunity/healthandsocialcare/healthandlifeexpectancies/bulletins/adultsmokinghabitsingreatbritain/2019 (accessed 01/08/2022)
30 Hu, L. T., & Bentler, P. M. 'Cutoff criteria for fit indexes in covariance structure analysis: Conventional criteria versus new alternatives'. *Structural equation modeling: a multidisciplinary journal*, 1999,*6*(1),1-55
31 Bentler, P. M. 'Comparative fit indexes in structural models' *Psychological bulletin*, 1990,*107*(2), 238
32 Bentler, P.M**. IBID**
33 Sen, S., & Bradshaw, L. 'Comparison of relative fit indices for diagnostic model selection' *Applied psychological measurement*, 2017,*41*(6), 422-438.

Figure legends

Figure 1. 18 item ABCD Questionnaire scree plot results from Nottingham dataset

Figure 2. Modified ABCD Questionnaire 20 items with smoking scree plot results Nottingham dataset

For peer review only - http://bmjopen.bmj.com/site/about/guidelines.xhtml

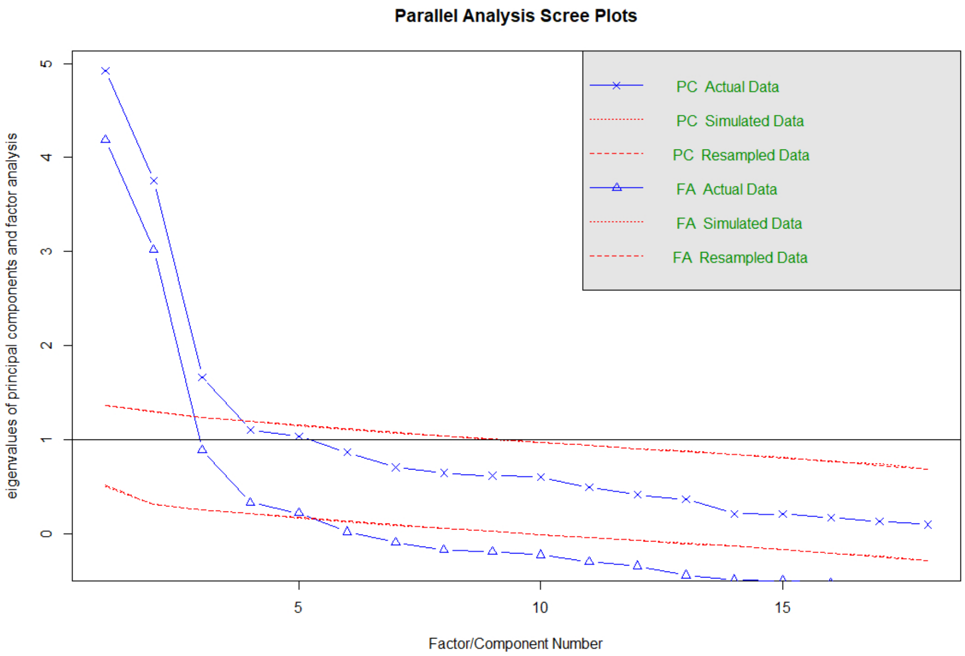

Figure 1. 18 item ABCD Questionnaire scree plot results from Nottingham dataset

286x198mm (96 x 96 DPI)

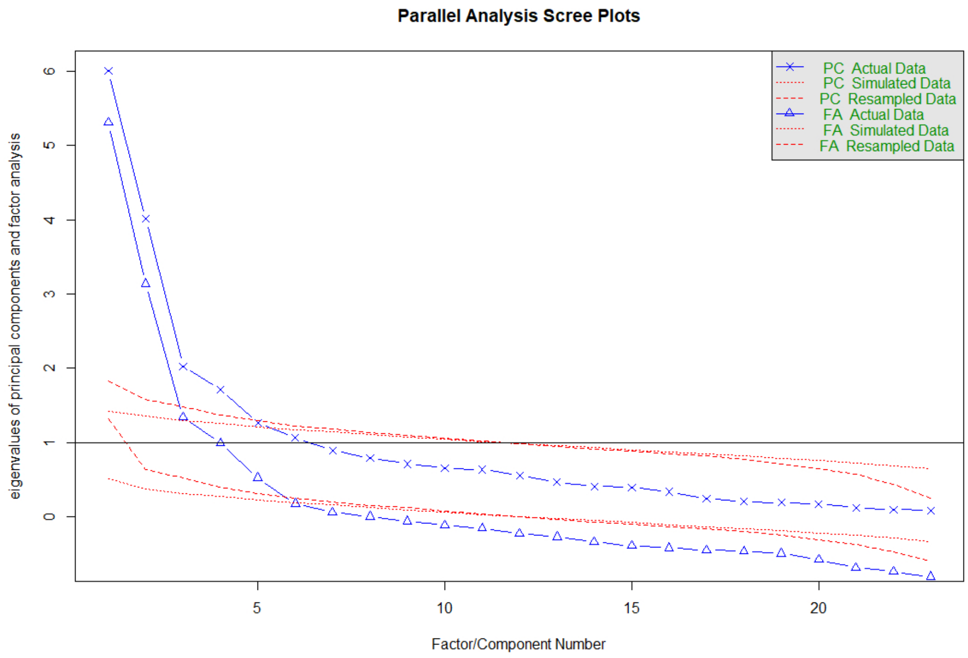

Figure 2. Modified ABCD Questionnaire 20 items with smoking scree plot results Nottingham dataset

286x198mm (96 x 96 DPI)

Horizon 2020
European Union funding
for Research & Innovation

NOTTINGHAM
TRENT UNIVERSITY

'SPICES' Heart Diseases Prevention Research

Introduction to SPICES research

Nottingham Trent University is part of an international research team investigating ways to build good practice in the prevention of Heart Diseases. Researchers and doctors have a lot of evidence about what causes heart diseases and what prevents them. Heart Diseases are now the biggest cause of death globally, and one of the leading causes of disability, so the more people know what the doctors know, the better they can protect themselves and maintain a good quality of life.

The research project is called 'SPICES' and here in Nottingham we are going to see if working with people in the community instead of at the doctor's surgery, we can spread the message quicker and further.

If you choose to take part we will ask you to complete a simple survey. From the we will be able see how well you are looking after your heart in terms of your lifestyle. Then there will be three possible options:

If the data you provide suggests you may need to make some lifestyle changes we will recommend that you make an appointment to see your doctor. As researchers we cannot give any medical advice, but it would be inappropriate for us to ignore any signs of an unhealthy lifestyle that could give rise to heart problems.

If the data you provide suggests you have a healthy lifestyle, then this is positive news and we'll talk to you about how you might be able to help the project in other ways.

If you are somewhere in the middle we will show you some simple ways to reduce your risk and stay healthier for longer.

N.B. In all cases, the data you provided is for research purposes only and a decision about your health cannot be made on the basis of questionnaires only.   Whilst we advise you to see a doctor if figures are high, lower figures should not be taken to indicate a healthy heart, and the results should not be used to replace medical assessments and the taking of medical advice about other health monitoring strategies. The dividing of participants into three groups is for research purposes only and is not a medical intervention.

If you're interested please complete our survey (It might take about 10 minutes, and you will need a tape measure for one of the questions).

Our researchers will then get in touch with you about ways that we can support you to make your heart healthier.   Any information we collect will be kept securely and not shared outside of the research team. Your name and personal details will not be used in any reports, and all our records will be destroyed at the end of the project in line with the relevant GDPR legislation. Additionally you may withdraw your data at any time up to but no later than December 31st 2020 by contacting Mark Bowyer, SPICES Coordinator, Nottingham Trent University 0115 8485574 mark.bowyer@ntu.ac.uk

OK? Let's start with your agreement to take part.

For peer review only

Horizon 2020
European Union funding
for Research & Innovation

NOTTINGHAM TRENT UNIVERSITY

CONSENT FORM

'SPICES' Heart Diseases Prevention Research

You are making a decision to take part. By ticking ALL statements and signing your name below you will indicate that you have read the information provided above and decided to participate.

If you choose to discontinue participation in this study, you may withdraw at any time without judgement, or effect on your status.

CONSENT STATEMENT		Please tick if you agree
**1.**	I have received, read and understood the SPICES participant information sheet	
**2.**	I am aware that I can withdraw my participation at any time without prejudice, judgement or effect on my status in relation to Nottingham Trent University or its research partners	
**3.**	I understand that information I provide during my participation can be deleted at my request up to but no later than December 31st 2020	
**4.**	I agree to be contacted by SPICES researchers using the details that I have supplied below	
**5.**	I understand that the collection of data is not part of medical assessment or diagnosis and cannot be relied upon to reach conclusions as to the state of my health	
**5.**	I understand that any information I provide as part of the SPICES research will be managed in accordance with the EU General Data Protection Regulation (GDPR) framework (see SPICES participant information sheet)	
**6.**	I agree to take part in this research project	

Name:

Preferred contact details:

D.O.B.

Gender:

Postcode:

Signature:

Date:

…………………………………………………………………………….

Staff signature:

Date:

For peer review only

A Protocol Paper: Community engagement interventions for Cardiovascular Disorders prevention in socially disadvantaged populations in the UK: An implementation research study
Final 15072019

Target Journal: Journal of Global Health Research and Policy
https://ghrp.biomedcentral.com/?gclid=Cj0KCQiA68bhBRCKARIsABYUGifuKd-xktjcmV7tn3r7G-IEqS5rAb6QmiEl6P9dXGBdNRDhsIPVzA0aAiJWEALw_wcB

Papreen Nahar[1], Harm van Marwijk[1], Linda Gibson[2], Geofrey Musinguzi[3], Sibyl Anthierens[4], Elizabeth Ford [1], Stephen A Bremner [1], Mark Bower [2], Jean Yves Le Reste[5], Tholene Sodi [6], Hilde Bastiaens[4]

Corresponding author: Dr Papreen Nahar, Department of Primary Care and Public Health, Brighton and Sussex Medical School, UK. The University of Sussex. E-mail: P.Nahar@bsms.ac.uk

Abstract:

Cardiovascular disorders (CVD) are the single greatest cause of mortality worldwide. In the UK, the National Health Service (NHS) has launched an initiative of health checks over and above current care to tackle CVD. However, the uptake of Health Checks is poor in disadvantaged communities. This protocol paper sets out a UK-based study aiming to co-produce a community delivered CVD risk assessment and coaching intervention to support community members to reduce their risk of CVD.

The overall aim of the project is to implement a tailored-to-context community engagement (CE) intervention on awareness of CVD risks in vulnerable populations in high, middle and low-income countries. This paper describes the protocol for the UK sites in Sussex and Nottingham. The specific objectives of the study are to enhance stakeholder' engagement; to implement lifestyle interventions for cardiovascular primary prevention, in disadvantaged populations and motivate uptake of NHS health checks.

This study takes a mixed methods approach, combining qualitative and quantitative methods in three phases of evaluation, including pre-, during- and post-implementation. To ensure contextual appropriateness the SPICES project will organize a multi-component community-engagement intervention implementation. For the qualitative component, the pre-implementation phase will involve a contextual assessment and stakeholder mapping, exploring potentials for CVD risk profiling strategies and led by trained Community Health Volunteers (CHV) to identify accessibility and acceptability. The during-implementation phase will involve healthy lifestyle counselling provided by CHVs and evaluation of the outcome to identify fidelity and scalability. The post-implementation phase will involve developing sustainable community-based strategies for CVD risk reduction. All three components will include a process evaluation. The theory of the socio-ecological framework will be applied to analyse the community engagement approach.

A stepped wedge quantitative evaluation of the roll out will focus on implementation outcomes such as uptake and engagement and changes in risk profiles. The quantitative component includes pre and post-intervention surveys.

The research project will ultimately develop a sustainable community engagement-based strategy for the primary prevention of CVD, to support or enhance the performance of NHS health care.

For peer review only

Key words: Implementation research, Cardiovascular disorders prevention, community engagement.

Introduction:

Cardiovascular disorders (CVD) are the single greatest cause of mortality worldwide each year, estimated to contribute to 31% of all deaths globally (1). Tackling CVD is an international priority and there have been many global initiatives such as the "Global Hearts" programme, a package launched by the World Health Organisation (WHO) and partners, to enhance the prevention and control of CVD. Some risk factors for CVD are non-modifiable, such as age, ethnicity and family history (2). Some other risk factors for CVD are modifiable, such as smoking, a lack of physical activity, being overweight, lower consumption of fruit and vegetables, high blood pressure, diabetes and high cholesterol (2). These risk factors can be changed through lifestyle or behavioural modifications. There is evidence of a social gradient in the prevalence of CVD, which points to associations between social and financial deprivation, vulnerability and risk factors for CVD. (3).

In 2015, CVD was the leading cause of mortality in the context of all chronic diseases, accounting for 27% and 25% of deaths in men and women respectively, in the UK(2). Coronary heart disease (CHD) and stroke were the main CVDs responsible for this mortality of men and women across all ages. As per British Heart Foundation report in 2017 CVD has a huge financial burden with annual associated healthcare costs estimated to be £9 billion annually in the UK (2). The UK has a standardised CVD death rate of 265.1 per 100,000 (2).

In the UK, the National Health Service (NHS) has launched the Health Check initiative aimed to prevent CVD. It is a national risk assessment and management program, free to adults aged 40 to 74 living in England, who do not currently have any vascular disorders and are not being treated for certain risk factors such as diabetes (4). It aims to assess the 10-year risk of CV events and disorders. Risk is assessed using QRISK2 (5), a tool which involves collection of the following information: age, gender, ethnicity, smoking status, family history of CHD, body mass index (BMI), cholesterol test, systolic and diastolic blood pressure, levels of physical activity, and alcohol consumption. Attendees receive a low (<10 % chance of event in 10 years), medium (>10 % but <20 %), or high (>20 %) 10-year cardiovascular (QRISK2) score. Above the 10% cut-off, attendees are offered a discussion with a qualified person, such as a nurse, about lifestyle and motivation to change, which may include goal setting and plans for follow up. Patients may also be offered medication for cholesterol and blood pressure. The NHS Health Check is recommended to be undertaken every five years.

Modelling predicted that the NHS Health Check could prevent 1,600 heart attacks and strokes each year if implemented as intended (6). Whilst evidence suggests that the Health Check programme has the potential to reduce CVD events and has therefore been rolled out nationally across the UK, its implementation has been poor, especially in some of the most disadvantaged groups at highest risk of developing CVD. In 2014, Public Health England (PHE) issued a call for action to increase the uptake rate of NHS Health Checks to 75% (7) and to increase awareness of risk and engagement with existing resources. Yet, as of 2017, current uptake remains far from this target with current predictions suggesting only 40% of the eligible population will receive one (8), due to the fact that uptake is low (48%) even when Health Checks are offered. (8) (9)

Data from some regions with very large ethnic minority community and socio-economically challenged populations showed that only 45% of patients who were invited for the check attended and subsequently received some form of counselling when they needed it. Authors have discussed how higher uptake in deprived communities would reduce the possibility of exacerbation of inequalities (10). Difficulty with accessing general practices, especially among socially vulnerable groups, has been highlighted as a common barrier to

For peer review only - http://bmjopen.bmj.com/site/about/guidelines.xhtml

attendance at Health Checks (11). A community-based engagement approach, which takes the CVD risking profiling and affiliated advice processes outside of the formal healthcare facility setting, has the potential to improve access to Health Checks and could be an effective and scalable way for improving the implementation and uptake of Health Checks. Community engagement (CE) has been conceptualised as "the process of working collaboratively with and through groups of people affiliated by geographic proximity, special interest, or similar situations, to address issues affecting the well-being of those people" (12). A review of community engagement interventions found them to be effective in improving health behaviours (such as physical activity), health consequences and psychological outcomes (i.e. self-efficacy and perceived social support) (13). Community-based intervention programmes have been implemented to increase the uptake of cancer screening programmes. The programmes have been found to be effective in increasing outcomes such as recognition, receipt and maintenance of screening behaviours (14). The CE approach offers the opportunity for task-shifting and owning the programme, whereby trained non-healthcare-professionals can perform CVD risk profiling assessments to individuals who might not otherwise be captured by the formal care pathway.

There is evidence that CVD risk assessments can be successfully delivered by Community Health Workers (CHWs), outside or inside the healthcare system. An observational study conducted in Bangladesh, Guatemala, Mexico and South Africa has demonstrated that CHWs who are inhabitants of their local communities and were fluent in the community's predominant language, can perform community-based screenings to predict CVD risk as effectively as physicians and nurses when using the non-laboratory-based Gaziano CVD risk scoring tool (15). CHWs were trained for 1-2 weeks, and results showed a 96.8% agreement between risk scores assigned by CHWs and healthcare professionals. However, a question remains whether the model taken in the global South could be transferrable to the global North, but it is at least plausible that a community-based engagement approach will be effective for increasing the uptake of CVD risk assessment, particularly in disadvantaged communities of the global North. There are examples in the global North on community engagement in health (16), and indeed the voluntary or 'third sector' have been considered key partners in the delivery of health promotion initiatives in the community (17).

Authors have argued that because of the current economic constraints with the formal healthcare system, the focus should be upon supplementing a service delivery model with an alternative community development model (18). The key aspect is supplementing formal service delivery by utilizing communities' 'social capital'. The term 'social capital' describes the various resources that people may have through their relationships in families, communities and other social networks. Social capital bonds people together and helps them make links beyond their immediate friends and neighbours (19).

For this compassionate community approach to work, contextual appropriateness and cultural sensitivity of an intervention is crucial (20). Following this argument, the SPICES project in two areas of England, East Sussex and Nottingham, will co-produce a multi-component community-engagement intervention focussed on delivering a Health Check-style CVD risk screening, with appropriate health coaching and follow-up, in a community setting (21) and delivered by community volunteers. The intervention will be trialled and evaluated using a mixed methods approach using both qualitative and quantitative methods. The specific objectives of the project are:

To evaluate with stakeholders the potential for a community engagement-based CVD primary prevention programme to support or enhance the NHS Health Check Programme.

To co-produce with the communities an evidence-informed community-engagement intervention on CVD risk, based on the NHS Health Check model, tailored to the context in disadvantaged communities in East Sussex and Nottingham.

For peer review only - http://bmjopen.bmj.com/site/about/guidelines.xhtml

To implement the intervention in the local communities where it was co-produced, and:
-assess its effectiveness versus routine care.
-assess the fidelity, feasibility, acceptability, uptake and scalability of the implementation.
-carry out a process evaluation of the intervention and its implementation

This project is part of the SPICES (Scaling-up Packages of Interventions for Cardiovascular disease prevention in selected sites in Europe and Sub-Saharan Africa) project (22). This is a Horizon 2020 project financed by the European Commission that aims to address the CVD burden. The overall objective is to implement and evaluate a comprehensive cardiovascular disease (CVD) prevention and care program at the community level in five countries (Belgium, France, Uganda, UK, South Africa), to identify and compare barriers and facilitators for implementation across study contexts and to develop a learning community.

Methods:

Theoretical Model

SPICES is underpinned by the Consolidated Framework for Advancing Implementation Research (23), and Reach, Effectiveness, Adoption, Implementation, and Maintenance (sustainability) framework /RE-AIM models (24). We also recognize as a global health project the need for the use of the socio-ecological framework (25). As mentioned above, this model allows an understanding of the multifaceted and interactive effects of personal, social and environmental factors that determine behaviour; and for identifying behavioural and organisational leverage points and intermediaries for health promotion within organisations and communities.

Study Design

A mixed-methods research methodology will be applied strategically combining qualitative and quantitative methods at both sites. This approach will allow us to model the iterative nature of coproduction and implementation research without compromising the rigour of the study (26; 27). The study will take place in three phases:
- Pre-intervention; when stakeholder mapping and local adaptation will be carried out
- Intervention roll out, recruitment and evaluation
- Post-intervention evaluations and feedback (28)- Process evaluation will be conducted in all three phases.

Stage 1: To explore the implementation context and co-produce the intervention.

To explore the context where the implementation will take place we will carry out several mappings. These will give us the context for recruitment and implementation co-design.
They are as follows:
(a) Mapping the potential stakeholders: Mapping of the stakeholders will be done to find out who are the key stakeholders, where they come from, and what they are looking for in relationship to the study objectives(29). To engage the community, it is essential to map the community stakeholders (civil society organisations) as they are the gatekeepers of the community. Three levels of stakeholder mapping will be carried out, namely at macro, meso and micro levels.
Macro-level: stakeholders will be identified via the existing link of PI of the project in the community through meetings with local public health or other relevant departments and CSOs and using online information. Interviews with this category of stakeholders will provide insights into implementation sustainability.
Meso-level: a strategic community volunteer organisation mapping will be carried out to find out the relevant organisations, through which individual volunteers will be selected. This will

For peer review only - http://bmjopen.bmj.com/site/about/guidelines.xhtml

be done in three ways; using online searches, personal contacts and snowballing. In-depth interviews will be conducted to co-design a sustainable intervention implementation.

Micro-level: an exploration will be done with volunteers and end-user groups to co-design an acceptable and feasible intervention implementation.

(b) Mapping the context: social mapping will be carried out to explore the lifestyle context of the community via observations.

(c) Training of volunteers by professional health trainers and researchers following current NICE Public health guideline [PH6] 'Behaviour change: general approaches' (30)

(d) CVD risk profiling by trained community health volunteers (CHV).

CHVs will be the persons who have been involved in health-related volunteering for example volunteers who worked in cancer prevention, health check, healthy lifestyle etc programme. They will be involved in the screening of the CVD risk population and implement the designed intervention.

Expected Intervention

The final elements of the intervention will be co-produced within each community setting, following the mapping exercises outlined above. As outlined in the CFAIR (23), interventions are usually composed of a core component which is essential and indispensable, and an adaptable periphery, which can and should be tailored to the specific setting and users.

Core Components: Following identification of moderate to high risk for CVD, the intervention will consist of non-clinical (non-NHS) individual or group support sessions within the community, focus on motivating behaviour change. Each participant will be supported by trained SPICES researchers or community health workers to identify behaviour change goals, produce action plans to achieve them, and problem solve in cases of unexpected outcomes. All SPICES Interventions are theoretically grounded in the theory of behaviour change and deploy the strongest evidenced Behaviour Change Techniques (BCTs) from the literature.

1. Goal Setting
2. Action Planning
3. Problem Solving
4. Motivational Interviewing
5. Feedback on progress towards goals
6. Feedback on the health impact

The use of these six BCTs are focussed in SPICES on five Target Behaviours:

1. Reduce/cease smoking
2. Increase moderate physical activity
3. Reduce fat, salt, the sugar content of the diet
4. Increase fibre, oily fish, fruit and vegetable content of the diet
5. Reduce sedentary hours

Community Adaptation: The exact elements of the support sessions will be tailored to individuals and their community context, will be determined during iterative co-design with community representatives, and will be drawn from the following (31; 32):

Step-I - Goal setting

Every participant should receive specific healthy lifestyle counselling/feedback based on their individual item InterHE ART assessment scores (the moderate group). The feedback will be based on a review of international guidelines conducted as formative work for the SPICES project intervention (33). SPICES behaviour change support sessions will be based on the best-evidenced approaches to healthy lifestyle modification and community context and preferences.

For peer review only - http://bmjopen.bmj.com/site/about/guidelines.xhtml

Two further screening questionnaires may be used with individuals to assess the benefit of possibly behaviour change;

- International Physical Activity Questionnaire (IPAQ, see appendix) is an internationally validated instrument to capture information about weekly physical activity habits, behaviours and routines.
- The Dietary Approaches to Stop Hypertension Questionnaire DASH-Q is a self-reporting lifestyle questionnaire (see appendix) to capture information about weekly dietary habits, routines and behaviours, based around 'Dietary Approach to Stopping Hypertension' (34).
- Current behaviours audit: Using food and physical activity diaries prepared by and provided to participants by the SPICES research team, participants will be encouraged to complete an audit of one week of current dietary and physical activity behaviours, habits and routines to establish a baseline from which goals for change and improvement can be set in negotiation with SPICES CHVs
- The ABCD self-reporting questionnaire (see appendix) to assess participant perception of personal heart health risk.
- The EQ-5D-5L internationally validated Quality of Life self-reporting questionnaire (see appendix).

Step-II - Action Planning by the participants

Participants will be asked to create an action plan with appropriate goal setting for two behaviours (diet and exercise habits) in relation to when, where and how they will undertake, for example, physical activity (based on the item stems used by Luszczynska & Schwarzer (35); when the physical activity will be performed, where it will be performed, how often it will be performed. The way goals are reached and plans recorded will be co-designed with key stakeholders.

Step III - Problem-solving

CHVs will help participants to analyse any factors which may influence their ability to achieve the goals and to generate strategies which could help them overcome these barriers.

CHVs will use Motivational Interviewing techniques about health, social and environmental, and emotional barriers and consequences. Culturally and context-sensitive information will be provided (both verbally and in the form of leaflets) about the importance of eating healthily, being physically active, and not smoking for positive outcomes on physical and mental health.

Trial of Intervention

This will be an open-label, non-controlled trial, examining fidelity, feasibility, acceptability, uptake and scalability of the intervention.

Eligible Population

Economically disadvantaged, lower socio-economic status (SES) postcodes, will be identified using the overall Index of Multiple Deprivation (36a); Participants' SES will be determined by their postcode of residence. Any resident aged 18 or above living in the study postcode areas will be eligible to take part in the baseline assessment for the study.

Study Sample Size

The sample size calculation for the quantitative study used statistical modelling for a stepped wedge design, randomising community centres over time with the InterRHEART score as the outcome (90% power for 5% significance, effect size (Cohen's D)=0.25, intracluster correlation coefficient of 0.05, control clusters crossing to intervention in 4 steps, participant autocorrelation=0.7 and cluster autocorrelation=0.9), which requires a total of at least 144 persons. This needs approximately 200-300 people across the two sites as we expect a high level of attrition (as much as 50%). At least 1500 community members will need to be screened to achieve this recruitment (37).

For peer review only - http://bmjopen.bmj.com/site/about/guidelines.xhtml

Recruitment of Community Health Volunteers and Trial Participants
Community Health Volunteers (CHVs) will be recruited to perform CVD risk profiling assessments through a combination of 'doorstep outreach' and 'intermediary organisation recruitment' approaches in East Sussex and through existing community and neighbourhood groups with the assistance of partners such as Self-Help UK, the Renewal Trust, Nottingham CVS and others in Nottingham.
For recruitment of trial participants, we will use similar community networks, and endeavour to use quota sampling, in that we will seek to ensure the inclusion of high, low and median income neighbourhood residents, citizens from the South Asian and African diasporas; and will encourage participants to refer others to the researchers who may be able to potentially contribute or participate in the study.

Baseline Screening of CVD Risk
Participants will fill in the validated InterHEART score to determine suitability for the trial. The non-laboratory-based InterHEART scoring tool requires minimal resources which is practical for use within the community. There is also evidence to suggest that the InterHEART can reliably predict the incidence of CVD and death in low, middle, and high-income countries for a mean follow-up of 4.1 years (38). Risk is expressed as a score from the InterHEART: 0-9 (Low risk), 10-15 (moderate risk), and 16-48 (high risk). The InterHEART scoring tool will be translated onto a mHealth platform so that the trained CHVs can easily administer them during community engagement and contact, and online data will directly reach the University repository in real time from the respondents' device.

Participants who score moderate or high risk in the baseline assessment will be invited to participate in the intervention. The moderate risk (amber) score population will be selected for participation in the intervention (=score of 10 or higher), and will fill out the self-completion survey InterHEART scoring every three months. The InterHEART scoring tool will be translated onto a mHealth platform so that the trained CHVs can easily administer them during community engagement and contact, and online data will directly reach the University repository in real time from the respondents' device (39).

Clinical Outcome and Follow-Up
The primary outcome will be the change in the risk score among people who complete the community delivered CVD risk assessment and coaching. Secondary outcomes will be gathered from participants identified as 'high risk'. Numbers of participants who a) self-referred (defined as having contacted their GP surgery requesting for a formal check-up) and b) completed the NHS Health Checks
Data collected during the trial of intervention will comprise:

- Self-reported lifestyle (modifiable and non-modifiable) risk factors gathered through survey instruments and interviews.
- Observed/measured data on all participants' age, gender, ethnicity, postcode, hip to waist ratio, gathered by trained volunteers.
- Quantitative analysis of changes in behavioural intention, target behaviours, and measurable CVD risk.

Outcomes will be assessed at three months post-intervention.

Post-intervention Qualitative Evaluation and Feedback
In the post-intervention phase, a qualitative evaluation will be carried out during which

The following implementation parameters will be assessed:

For peer review only - http://bmjopen.bmj.com/site/about/guidelines.xhtml

1. The impact on awareness of CVD risks and mitigating measures, amongst disadvantaged populations of a community-based, non-clinical, CVD risk scoring tool and education.
2. The impact of the community based non-clinical CVD risk scoring tool and education on motivational healthy lifestyle among disadvantaged populations.
3. The facilitators and barriers to the adoption of a community-based CVD prevention implementation programme, by target populations.
4. The perspectives of participants regarding their experience and meaning of the intervention.

These will be explored with a subset of intervention participants using focus groups or/and in-depth interview and community mapping. Participants for the qualitative component will include adult volunteers, public health stakeholders and people within the community. The community volunteers will be selected via community organisations and public health stakeholders will be selected from the same area of the research site. Community participants for the qualitative component will be selected via the community volunteers. This post-intervention qualitative study will include randomly selected trial participants.

We will be flexible in terms of the number of participants for the qualitative component. The number will be determined through the principle of saturation and diversity. However, from each site, we will aim to include at least 12 respondents and a maximum of 30 respondents from different categories (40; 41).

Process evaluation of the intervention

To assess the fidelity of the conclusions concerning the project's effectiveness, ongoing assessment, monitoring, and enhancement is important. If significant results are found, but fidelity was not assessed, it cannot be determined if the effectiveness is attributable to unintentionally added or omitted components. Bellg and colleagues (42) propose that considerations of fidelity should permeate all stages of the study: design of the study, provision of training, delivery of the intervention, receipt of the intervention, and re-enactment of skills. As a result, we will carry out a process evaluation of the project. This will be done through Process Documentation of all the stages of this project including community volunteers mapping, Healthy lifestyle counselling, action planning and problem-solving.

Thirsk and Clark (43) argue how health-care interventions need to be understood in ways that are responsive to the complexities and intricacies of programs, people and places. They emphasise the understanding of the comprehensive experience of the persons who are delivering and receiving the intervention. Process Evaluation is a tool that can capture the intervention experience. We will be following the model designed by Moore et al (44):

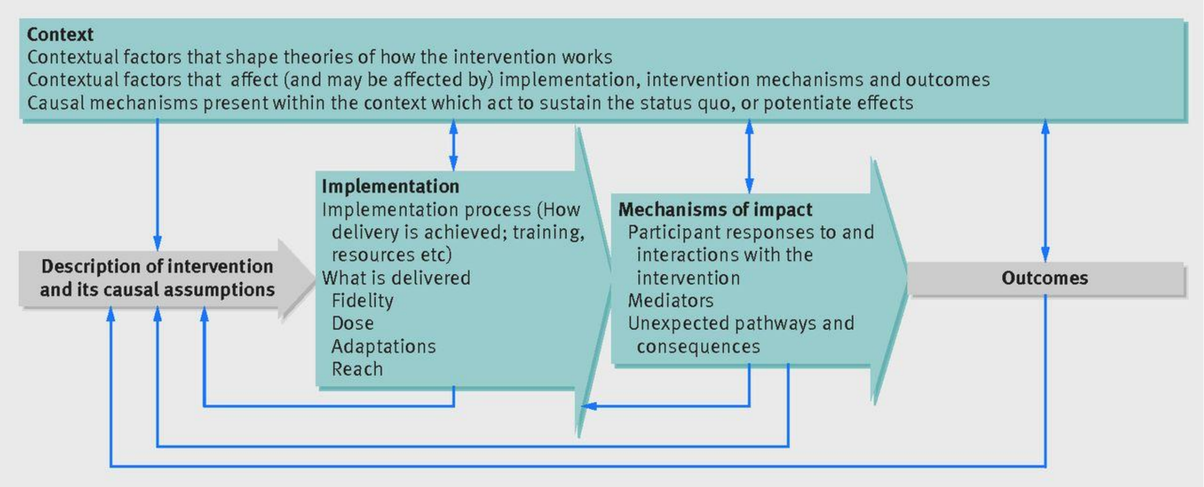

For peer review only - http://bmjopen.bmj.com/site/about/guidelines.xhtml

Data Analysis:
Quantitative data will be analysed using Stata version 15 or later. Descriptive statistics will summarise outcomes before and after clusters cross over to the intervention (45. Normally distributed variables will be summarised by means and standard deviations, skewed continuous variables by medians and interquartile ranges, categorical variables by frequencies and percentages. We will estimate the treatment effect using a cross-classified linear mixed effects model. A statistical analysis plan will be agreed and signed off prior to final analysis commencing. Thematic analysis of qualitative data will be carried out using a constant comparison method of analysis, which will gather and generate ideas and categories through inductive processes. The computer package NVivo will be used for primary analysis (46). Memo writing will be carried out to describe details of the interview setting and interaction of respondent and interviewer that may not be captured in audio transcriptions. This thematic analysis has deductive and inductive elements, lending itself to multidisciplinary health research (47). The analysis framework will incorporate the key theoretical constructs and respond to the context of policy and practice to include a range of deductive themes. Further themes will be induced from the interview data.

An appropriate balance of integration between empirical data and interpretation will be ensured. The investigators will extract the meaning of the empirical data and interpret them whilst acknowledging the complexity of the phenomena of CVD risk reduction in the context of community engagement (48). This method holds links to the original data and the output allows comprehensive and transparent data analysis.

Conclusion:
Given that despite the rolling out of the NHS Health Checks programme over and above current care across the UK has not been implemented as well as it could have been, especially in some of the most disadvantaged groups prone to developing CVD, the project aims to scale-up packages of interventions for cardiovascular prevention particularly to these vulnerable populations. This interdisciplinary project includes public health, social and behavioural science approaches. The main focus aspect of this project is the deinstitutionalization of health care by operating outside of formal healthcare settings. The project will emphasise on the power of citizens, combining their efforts to generate cultures of care which complement or even compensate for the inadequacies of formal systems thus sustainable. The research project will ultimately develop a community engagement-based CVD primary prevention programme to support or enhance the performance of the NHS health care.

Funding statement:
This protocol is a contextual plan for the SPICES project in the UK. The SPICES project received funding from the European Commission through the Horizon 2020 Research and Innovation Action Grant Agreement No 733356 to implement and evaluate a comprehensive CVD prevention programme in five settings: a rural & semi-urban community in a low-income country (Uganda), middle income (South Africa) and vulnerable groups in three high-income countries (Belgium, France and United Kingdom). The funder had no role in the design, decision to publish, or preparation of the manuscript.

Availability of data and materials:
A protocol should not contain any data; it sets out the research questions and how they will be addressed.

For peer review only - http://bmjopen.bmj.com/site/about/guidelines.xhtml

Ethics approval and consent to participate:
This protocol has received two ethics approval from the University of Sussex, The **BSMS** Research Governance and Ethics Committee (RGEC (ER/BSMS9E3G/1)), and from Nottingham Trent University (no. TBA). All participants will be requested to consent before enrolment into the study. All participant information will be kept confidential and accessible only to the key investigative team. All published data will be anonymised and can be accessed based on a written request to the Principal Investigator.

Competing interests:
Authors declare that they have no competing interests.

Authors' contributions:
PN has written the first draft and received feedback from HvM and SA on it. PN prepared the second draft and it received feedback from LG. The third draft received feedback from all the authors. All authors read and approved the final contextual protocol (4th version).

References:

1. World Health Organisation; WHO. *Global Health Observatory (GHO) data*. 2017. Retrieved on 18th May 2019, retrieved from https://www.who.int/gho/mortality_burden_disease/causes_death/top_10/en

2. British Heart Foundation report BHF. https://www.bhf.org.uk/informationsupport/publications/policy-documents/annual-report-2017.

3. Amuzu A, Carson C, Watt HC, Lawlor DA, Ebrahim S. Influence of area and individual life course deprivation on health behaviours: findings from the British Women's Heart and Health Study. Eur J Cardiovasc Prev Rehabil. 2009;16(2): 169-73. doi: 10.1097/HJR.0b013e328325d64d.

4.Public Health England (2013). NHS Health Check implementation review and action plan Summary, https://assets.publishing.service.gov.uk/government/uploads/system/uploads/attachment_data/file/224536/NHS_Health_Check_implementation_review_and_action_plan_summary_web.pdf 5.

5. QRISK2. 2017. https://qrisk.org/2017/

6. Public Health, England. NHS Health Checks 'prevented thousands of heart attacks'. 2016. https://www.nhs.uk/news/heart-and-lungs/nhs-health-checks-prevented-thousands-of-heart-attacks/

7. Public Health England. Factsheet: Implementation of the NHS Health Check programme. 2014. https://www.england.nhs.uk/wp-content/uploads/2014/02/pm-fs-3-1.pdf

8. Waterall Jamie. PHE. Getting Serious About CVD Prevention. Reducing Variation & Optimising Care. 2018. https://www.healthcheck.nhs.uk/seecmsfile/?id=195

For peer review only - http://bmjopen.bmj.com/site/about/guidelines.xhtml

9. Dalton AR, Bottle A, Okoro C, Majeed A, Millett C. Uptake of the NHS Health Checks programme in a deprived, culturally diverse setting: cross-sectional study. J Public Health (Oxf). 2011; 33(3):422-9. doi: 10.1093/pubmed/fdr034.

10. Robson J, Dostal I, Sheikh A, et al. The NHS Health Check in England: an evaluation of the first 4 years. 2015. BMJ Open. 2015;6: e008840. doi:10.1136/ bmjopen-2015

11. Harte Emma, Calum MacLure, Adam Martin, Catherine L Saunders, Catherine Meads, Fiona M Walter, Simon J Griffin, Jonathan Mant and Juliet A Usher-Smith (2018), Reasons why people do not attend NHS Health Checks: a systematic review and qualitative synthesis, British Journal of General Practice. 2018;68 (666): e28-e35. DOI: https://doi.org/10.3399/bjgp17X693929

12. CTSA. Principles of Community Engagement, Second Edition, NIH Publication No. 11-7782. 2011. https://.atsdr.cdc.gov/communityengagement/pdf/PCE_Report_508_FINAL.pdf

13. O'Mara-Eves A., G Brunton, D McDaid, S Oliver, J Kavanagh, F Jamal, T Matosevic, A Harden, and J Thomas. Community engagement to reduce inequalities in health: a systematic review, meta-analysis and economic analysis, Public Health Research. 2013;1:4

14. Bird JA; McPhee SJ, Ha NT, Le B, Davis T, Jenkins CN. Opening pathways to cancer screening for Vietnamese-American women: lay health workers hold a key. Prev Med. 1998;27(6):821-9.

15. Gaziano Thomas A, Shafi ka Abrahams-Gessel, Catalina A Denman, Carlos Mendoza Montano, Masuma Khanam, Thandi Puoane, Naomi S Levitt (2015). An assessment of community health workers' ability to screen for cardiovascular disease risk with a simple, non invasive risk assessment instrument in Bangladesh, Guatemala, Mexico, and South Africa: an observational study. Lancet Glob Health. 2015; 3:e556–63. http://dx.doi.org/10.1016/ S2214-109X(15)00142-4

16. Wood, J. The University as a Public Good: Active citizenship and university community engagement. International Journal of Progressive Education. 2012; 8 (3), 15-31. Retrieved from http://dergipark.org.tr/ijpe/issue/26312/277299

17. Kings Fund. 2011. Retrieved from, https://www.kingsfund.org.uk/sites/default/files/Voluntary-and-community-sector-in-health-implications-NHS-reforms-The-Kings-Fund-june-2011_0.pdf

18. Abel, J., Bowra, J., Walter, T. et al. Compassionate Community networks: Supporting home dying. BMJ Supportive and Palliative Care. 2011;1,129-133. http://dx.doi.org/10.1136/bmjspcare-2011-000068

19. Baum FE and Ziersch AM. Social capital. Journal of Epidemiology & Community Health. 2003; 57: 320-323.

20. Sallnow L. Libby, Heather Richardson, Scott Murray, Allan Kellehear. Understanding the impact of a new public health approach to end of life care: a qualitative study of a community-

For peer review only - http://bmjopen.bmj.com/site/about/guidelines.xhtml

led intervention. The Lancet. 2017; 389: Special issue 788. DOI:https://doi.org/10.1016/S0140-6736(17)30484-1

21. Boyd, Stephen McKernon, Bernie Mullin, Andrew Old. Improving healthcare through the use of co-design. The New Zeeland Medical Journal. 2012; 125:(1357).

22. Musinguzi Geofrey, Rhoda K. Wanyenze1, Rawlance Ndejjo, Isaac Ssinabulya, Harm van Marwijk, Isaac Ddumba, Hilde Bastiaens and Fred Nuwaha. An implementation science study to enhance cardiovascular disease prevention in Mukono and Buikwe districts in Uganda: a stepped-wedge design. Health Service research. 2019; 19:253 doi.org/10.1186/s12913-019-4095-0

23. Damschroder Laura J, David C Aron, Rosalind E Keith, Susan R Kirsh, Jeffery A Alexander and Julie C Lowery. Fostering implementation of health services research findings into practice: a Consolidated Framework for Advancing Implementation Science. Implementation Science. 2009; 4:50 doi:10.1186/1748-5908-4-50

24. Glasgow RE, McKay HG, Piette JD, Reynolds KD: The RE-AIM framework for evaluating interventions: what can it tell us about approaches to chronic illness management? Patient Educ Couns. 2001;44:119-127.

25. Theobald, S., Brandes, N., Gyapong, M., et al. Implementation research: new imperatives and opportunities in global health, Lancet. 2018;392: 2214–28

26. Peters DH, Adam T, Alonge O, et al. Implementation research: what it is and how to do it. BMJ. 2013; 347: f6753.

27. Brown CH, Curran G, Palinkas LA, et al. (2017). An overview of research and evaluation designs for dissemination and implementation. Annual Review of Public Health. 2017; 38:1–22

28. Greene, J. C. Mixed Methods in Social Inquiry. 2007. Jossey-Bass. San Francisco.

29. BSR. Stakeholder Mapping (2011). www.bsr.org/reports/BSR_Stakeholder_Engagement_Stakeholder_Mapping.final.pdf

30. NICE Public health guideline [PH6] 'Behaviour change: general approaches' (https://www.nice.org.uk/guidance/ph6/resources/behaviour-change-general-approaches-pdf-55457515717).

31. Michie Susan, Michelle Richardson, Marie Johnston, et al. (2013). The Behaviour Change Technique Taxonomy (v1) of 93 Hierarchically Clustered Techniques: Building an International Consensus for the Reporting of Behavior Change Interventions. Annals of Behavioural Medicine. 2013. 46;(1):81–95, https://doi.org/10.1007/s12160-013 9486-6

32. Michie, Susan, Maartje M. Van Stralen, and Robert West. "The Behaviour Change Wheel: A New Method for Characterising and Designing Behaviour Change Interventions. Implementation Science. 2011;6,1:42.

For peer review only - http://bmjopen.bmj.com/site/about/guidelines.xhtml

33. Yusuf Salim, Steven Hawken, Stephanie Ôunpuu, et al., on behalf of the INTERHEART Study Investigators. Effect of potentially modifiable risk factors associated with myocardial infarction in 52 countries (the INTERHEART study): a case-control study. Lancet. 2004; 364: 937–52

34. Sacks Frank M., Laura P. Svetkey, M.D., William M. Vollmer et al. Effects on Blood Pressure of Reduced Dietary Sodium and the Dietary Approaches to Stop Hypertension (DASH) Diet.
N Engl J Med. 2001; 344:3-10, DOI:10.1056/NEJM200101043440101

35. Luszczynska Aleksandra, Urte Scholz & Ralf Schwarzer. The General Self-Efficacy Scale: Multicultural Validation Studies, The Journal of Psychology. 2005; 139:5, 439-457, doi:10.3200/JRLP.139.5.439-457

36.. Kontopantelis E, Mamas MA, Van Marwijk H, Ryan AM, Buchan IE, Ashcroft DM, et al. Geographical epidemiology of health and overall deprivation in England, its changes and persistence from 2004 to 2015: A longitudinal spatial population study. 2018; 72 (2):140–7.

37. Hooper R, Teerenstra S, de Hoop E & Eldridge S. Sample size calculation for the stepped wedge and other longitudinal cluster randomised trials. Statist. Med. 2016; 35 4718–4728

38. Yusuf Salim, Sumathy Rangarajan, Koon Teo, Shofiqul Islam, et al. Cardiovascular. Risk and Events in 17 Low-, Middle-, and High-Income Countries. N Engl J Med. 2014; 371:818-827, DOI: 10.1056/NEJMoa1311890

39. Williams Natalie Joseph, Amy Lloyd, Adrian Edwards, Lynne Stobbart, David Tomson, Sheila Macphail, Carole Dodd, Kate Brain, Glyn Elwyn, Richard Thomson. Implementing shared decision making in the NHS: lessons from the MAGIC programme. BMJ. 2017a;357:j1744 doi: https://doi.org/10.1136/bmj.j1744

40. Guest, G., Bunce, A., & Johnson, L. How Many Interviews Are Enough?: An Experiment with Data Saturation and Variability. Field Methods. 2006; 18 (1): 59–82. https://doi.org/10.1177/1525822X05279903

41. Malterud K, Siersma VD, Guassora AD. Sample Size in Qualitative Interview Studies: Guided by Information Power. Qual Health Res. 2015; 1–8.

42. Bellg, A. J., Borrelli, B., Resnick, B., Hecht, J., Minicucci, D. S., Ory, M., et al. Treatment Fidelity Workgroup of the NIH Behavior Change Consortium. (2004). Enhancing Treatment Fidelity in Health Behavior Change Studies: Best Practices and Recommendations From the NIH Behavior Change Consortium. Health Psychology. 2004; 23(5):443-451. http://dx.doi.org/10.1037/0278 6133.23.5.443

43. Thirsk Lorraine M. and Alexander M. Clark. Using Qualitative Research for Complex Interventions: The Contributions of Hermeneutics. International journal of qualitative methods. 2017; 16:1-10. doi.org/10.1177/1609406917721068

For peer review only - http://bmjopen.bmj.com/site/about/guidelines.xhtml

44. Moore Laurence, Alicia O'Cathain, Tannaze Tinati, Daniel Wight, Janis Baird (2015). Process evaluation of complex interventions: Medical Research Council guidance. The BMJ. 2015;350:h1258. doi: 10.1136/bmj.h1258

45. StataCorp. 2017. *Stata Statistical Software: Release 15*. College Station, TX: StataCorp LLC

46. Ritchie Jane, Jane Lewis, Carol McNaughton Nicholls, Rachel Ormston. Qualitative Research Practice: A Guide for Social Science Students and Researcher; 2013. Sage Publications.

47. Gale NK, Heath G, Cameron E, Rashid S, Redwood S. Using the framework method for the analysis of qualitative data in multi-disciplinary health research. BMC Med Res Methodol. 2013;(13):117. doi.org/10.1186/1471-2288-13-117

48. Green J, Thorogood N. Qualitative methods for health research. 2005. London: Sage Publications Ltd.

Authors Information:

1. Papreen Nahar. Department of Primary Care and Public Health, Brighton and Sussex Medical School. University of Sussex, UK.
1. Harm van Marwijk. Department of Primary Care and Public Health, Brighton and Sussex Medical School. The University of Sussex. UK
2. Linda Gibson: School of Social Sciences. Nottingham Trent University, UK
3. Musinguzi Geofrey. Department of Disease Control and Environmental Health, School of Public Health, College of Health Sciences. Makerere University, Uganda
4. Sibyl Anthierens. Department of Primary and Interdisciplinary Care, University of Antwerp, Belgium
1. Elizabeth Ford. Department of Primary Care and Public Health Brighton and Sussex Medical School. University of Sussex, UK
1. Stephen A Bremner. Department of Primary Care and Public Health Brighton and Sussex Medical School. University of Sussex, UK
2. Mark Bower. School of Social Sciences, Nottingham Trent University, UK
5. JY Reste. Faculté de médecine et des sciences de la santé, Université de Bretagne Occidentale, Brest, France
6. Sodi Tholene. Department of Psychology. University of Limpopo, South Africa
4. Hilde Bastiaens. Department of Primary and Interdisciplinary care. University of Antwerp, Belgium

For peer review only

Appendix 3. Characteristics of the sample population

**Population Characteristics**		**N**	**% total**
**Gender**	Male	218	49.8
	Female	220	50.2
**Age Group**	18-30	78	17.8
	30-39	80	18.3
	40-49	82	18.7
	50-59	99	22.6
	60-74	78	17.8
	74+	53	12.1
**Deprivation**	IMD1- least deprived	84	17.98
	IMD2	55	11.77
	IMD3	83	17.77
	IMD4	89	19.05
	IMD5- most deprived	156	33.4

For peer review only

Appendix 4

ABCD subscale and selected INTERHEART variable correlation values from Nottingham study compared with values reported in the original Woringer study.

		Knowledge	Perceived Risk	Perceived Benefit	Healthy Intentions	IMD2010 Quintile	BMI/W2Hr	Qrisk2/ INTERHEART
Knowledge	Correlation Coefficient		-.124/ **.013**	-.148/ **-.021**	-.106/ **-.039**	-.002/ .085	-.225/ -.084	-.007/ -.018
	Sig 2 tailed		.236/ .722	.175/ .645	.319/ .400	.986/ .066	.021/ .082	.941/ .714
	N		93/462	86/462	91/462	99/466	105/433	104/436
Perceived Risk	Correlation Coefficient			-.195/ -.112	-.188/ -0.36	.239/ .039	.389/ .182	.220/ .356
	Sig 2 tailed			.080/ .016	.088/ .441	.025/ .397	.000/ .000	.036/ .000
	N			82/462	84/462	87/466	92/433	91/436
Perceived Benefits	Correlation Coefficient				.533/ .383	-.287/ .071	-.068/ .000	-.118/ -.164
	Sig 2 tailed				.000/ .000	.009/ .127	.538/ .997	.284/ .001
	N				83/462	81/466	85/433	84/436
Healthy Intentions	Correlation Coefficient					-.261/ .098	.084/ .044	-.072/ -.079
	Sig 2 tailed					.016/ .034	.430/ .365	.504/ .100
	N					85/466	90/462	89/436

Correlations

Correlations

Correlations

			knowledge score	Risk score	Benefit score	Diet score	Smoke score	total_score
Spearman's rho	knowledge score	Correlation Coefficient	1.000	.118**	.103*	.078	-.079	.006
		Sig. (2-tailed)	.	.009	.023	.086	.082	.896
		N	483	483	483	483	483	440
	Risk score	Correlation Coefficient	.118**	1.000	-.003	.057	.107*	.371**
		Sig. (2-tailed)	.009	.	.950	.212	.019	.000
		N	483	483	483	483	483	440
	Benefit score	Correlation Coefficient	.103*	-.003	1.000	.538**	.009	-.236**
		Sig. (2-tailed)	.023	.950	.	.000	.851	.000
		N	483	483	483	483	483	440
	Diet score	Correlation Coefficient	.078	.057	.538**	1.000	-.022	-.143**
		Sig. (2-tailed)	.086	.212	.000	.	.635	.003
		N	483	483	483	483	483	440
	Smoke score	Correlation Coefficient	-.079	.107*	.009	-.022	1.000	.240**
		Sig. (2-tailed)	.082	.019	.851	.635	.	.000
		N	483	483	483	483	483	440
	total_score	Correlation Coefficient	.006	.371**	-.236**	-.143**	.240**	1.000
		Sig. (2-tailed)	.896	.000	.000	.003	.000	.
		N	440	440	440	440	440	440

** Correlation is significant at the 0.01 level (2-tailed).

* Correlation is significant at the 0.05 level (2-tailed).

For peer review only

Appendix 5. Figures and factor results tables

Without smoking items

Non-missing samples: 420

Bartlett's Test of Sphericity (X2 = 4235.007, p-value < 0.001)

The overall KMO is 0.82, which is within the recommended range (0.8 to 1).

EFA results

- The root mean square of the residuals (RMSR) is 0.05
- Tucker Lewis Index of factoring reliability = 0.77
- RMSEA index = 0.121 and the 90 % confidence intervals are 0.113 0.129
- BIC = 165.35

Scree plot

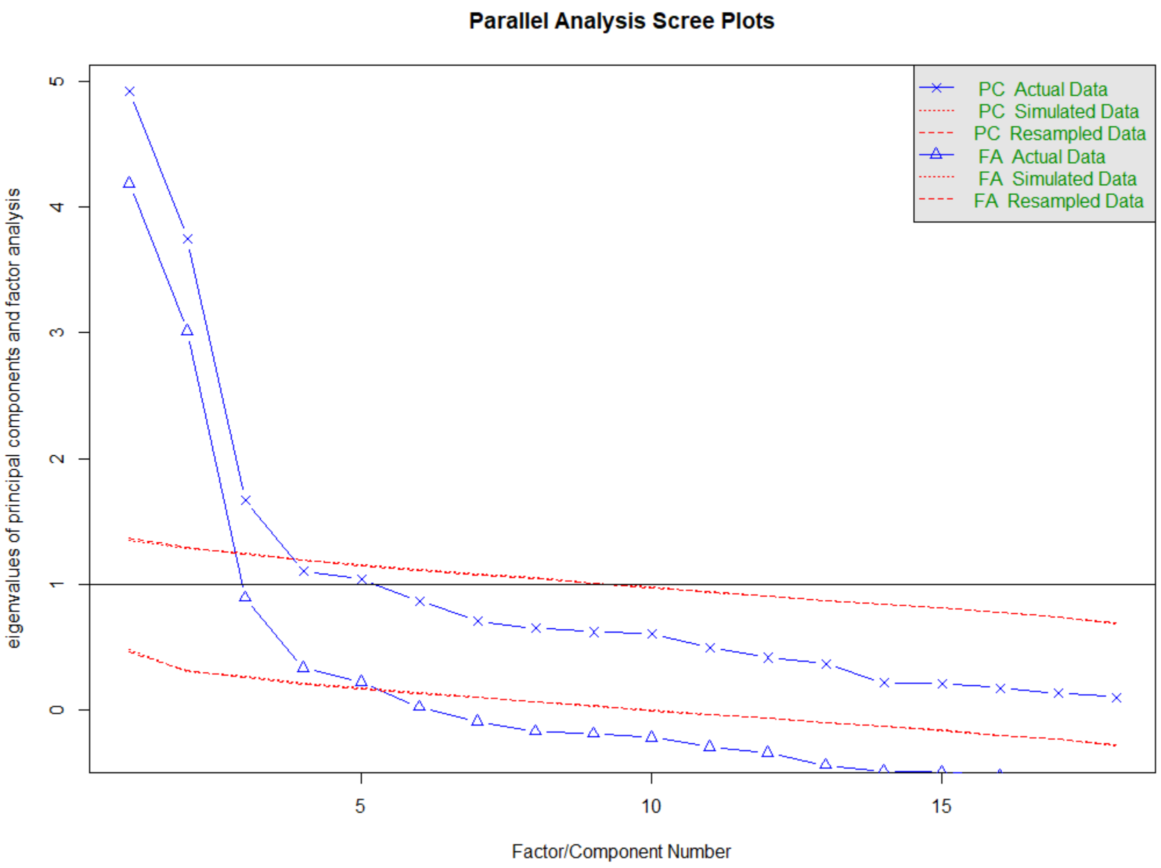

Figure 1. 18-item ABCD Questionnaire results (without smoking items)

Table A1 (a). Factor loadings of the exploratory factor analysis of the risk scale without the smoking items

Items	Factor2	Factor1	Factor3	communality	uniqueness
I feel I will suffer from a heart attack or stroke sometime during my life	0.86	0.02	-0.03	0.74	0.26
It is likely that I will suffer from a heart attack or stroke in the future	0.91	0.05	0.00	0.82	0.18
It is likely that I will have a heart attack or stroke sometime during my life	0.88	0.01	0.01	0.77	0.23
There is a good chance I will experience a heart attack or stroke in the next 10 years	0.73	-0.07	0.01	0.55	0.45
My chances of suffering from a heart attack or stroke in the next 10 years are great	0.65	-0.10	0.01	0.44	0.56
It is likely I will have a heart attack or stroke because of my past and/or present behaviors	0.56	-0.03	-0.01	0.32	0.68
I am not worried that I might have a heart attack or stroke (Reverse coded)	0.28	-0.11	0.10	0.10	0.90
I am concerned about the likelihood of having a heart attack or stroke in the near future	0.40	-0.02	0.11	0.16	0.84
I am thinking about exercising at least 2.5 hours a week	-0.02	0.87	-0.06	0.73	0.27
I intend or want to exercise at least 2.5 hours a week	-0.01	0.91	-0.04	0.80	0.20
When I exercise for at least 2.5 hours a week I am doing something good for the health of my heart	0.02	0.69	0.10	0.53	0.47
I am confident that I can maintain a healthy weight by exercising at least 2.5 hours a week	-0.05	0.45	0.19	0.31	0.69
I am not thinking about exercising for 2.5 hours a week (Reverse coded)	0.04	0.56	0.05	0.34	0.66
When I eat five portions of fruit and vegetables a day I am doing something good for the health of my heart	0.02	0.37	0.35	0.36	0.64
Increasing my exercise to at least 2.5 hours a week will decrease my chances of having a heart attack or stroke	0.02	0.39	0.27	0.30	0.70
I am confident that I can eat at least five portions of fruit and vegetables a day within the next two months	-0.04	0.07	0.64	0.46	0.54
I am thinking about eating at least five portions of fruit and vegetables a day	0.01	-0.01	0.93	0.85	0.15
I am not thinking about eating at least five portions of fruit and vegetables a day (Reverse coded)	-0.01	-0.03	0.78	0.60	0.40

Table A1 (b): Summary of factor loadings and variance distribution of the risk scale without the smoking items

Measures	Factor 2	Factor 1	Factor 3
SS loadings	3.86	3.04	2.28
Proportion Var	0.21	0.17	0.13

For peer review only

Cumulative Var	0.21	0.38	0.51
Proportion Explained	0.42	0.33	0.25
Cumulative Proportion	0.42	0.75	1.00

With smoking items

Non-missing samples: 88

The overall KMO is 0.78, which is slightly below the recommended range (0.8 to 1).

The Bartlet's test of Sphericity is significant (X2 = 1223.459, p-value < 0.001), indicating the sample adequacy for factor analysis.

EFA results

- The root mean square of the residuals (RMSR) is 0.06
- Tucker Lewis Index of factoring reliability = 0.69
- RMSEA index = 0.129 and the 90 % confidence intervals are 0.124 and 0.136
- BIC = 440.9

Scree plot

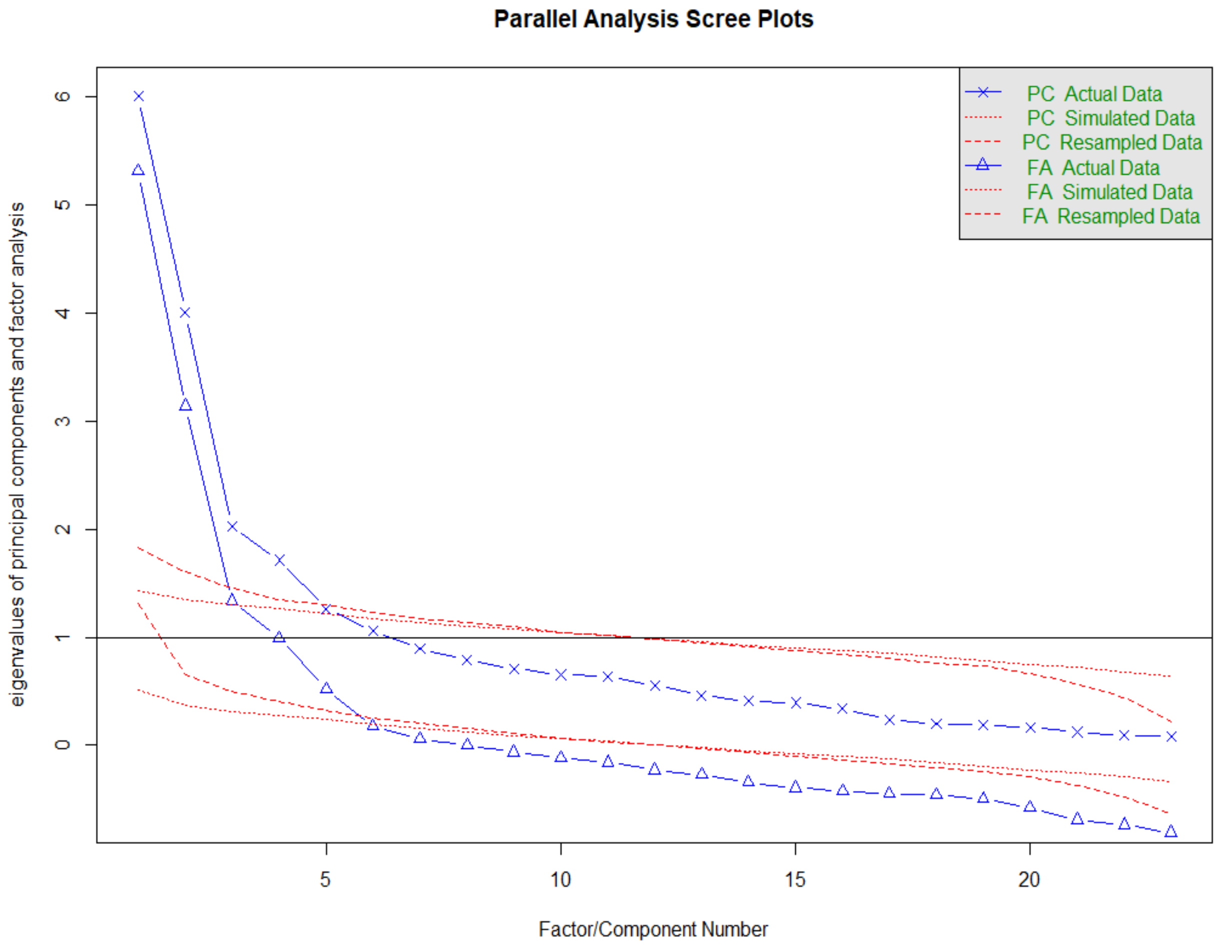

Figure 2. Modified ABCD Questionnaire 23 items with smoking.

Table A2 (a). Factor loadings of the exploratory factor analysis of the risk scale with the smoking items

Items	Factor2	Factor3	Factor1	Factor4	Communality	Uniqueness
I feel I will suffer from a heart attack or stroke sometime during my life	0.86	-0.1	0.05	-0.02	0.76	0.24
It is likely that I will suffer from a heart attack or stroke in the future	0.91	0.06	0.02	-0.01	0.82	0.18
It is likely that I will have a heart attack or stroke sometime during my life	0.88	0.02	0	0	0.77	0.23
There is a good chance I will experience a heart attack or stroke in the next 10 years	0.72	0	-0.09	0.01	0.54	0.46
My chances of suffering from a heart attack or stroke in the next 10 years are great	0.64	-0.03	-0.1	0.01	0.45	0.55
It is likely I will have a heart attack or stroke because of my past and/or present behaviors	0.57	-0.07	0	0	0.33	0.67
I am not worried that I might have a heart attack or stroke (Reverse coded)	0.28	0.02	-0.14	0.1	0.1	0.9
I am concerned about the likelihood of having a heart attack or stroke in the near future	0.41	0.19	-0.12	0.08	0.19	0.81
I am thinking about exercising at least 2.5 hours a week	-0.03	-0.05	0.88	-0.02	0.73	0.27
I intend or want to exercise at least 2.5 hours a week	-0.02	0.05	0.87	-0.02	0.79	0.21
When I exercise for at least 2.5 hours a week I am doing something good for the health of my heart	0.03	0.17	0.62	0.09	0.55	0.45
I am confident that I can maintain a healthy weight by exercising at least 2.5 hours a week	-0.05	0.09	0.42	0.18	0.32	0.68
I am not thinking about exercising for 2.5 hours a week (Reverse coded)	0.02	0	0.53	0.09	0.33	0.67
When I eat five portions of fruit and vegetables a day I am doing something good for the health of my heart	0.04	0.07	0.35	0.35	0.36	0.64
Increasing my exercise to at least 2.5 hours a week will decrease my chances of having a heart attack or stroke	0.04	0.12	0.37	0.24	0.32	0.68
I am confident that I can eat at least five portions of fruit and vegetables a day within the next two months	-0.04	-0.05	0.12	0.64	0.45	0.55
I am thinking about eating at least five portions of fruit and vegetables a day	0.01	0	0.02	0.89	0.8	0.2
I am not thinking about eating at least five portions of fruit and vegetables a day (Reverse coded)	-0.01	0	-0.06	0.83	0.66	0.34
I am thinking of stopping smoking within two months	0.06	0.78	0.12	-0.06	0.67	0.33

For peer review only

I have reduced or stopped smoking	-0.03	0.83	0.02	-0.01	0.71	0.29
I intend or want to stop smoking	-0.05	0.9	-0.02	-0.01	0.8	0.2
If I stop smoking it will reduce my chances of having a heart attack or stroke	0.16	0.58	0.09	0.08	0.43	0.57
I am not thinking about stopping smoking	-0.12	0.56	-0.2	0.17	0.35	0.65

Table A2 (b): Summary of factor loadings and variance distribution of the risk scale with the smoking items

Measures	Factor 2	Factor 3	Factor 1	Factor 4
SS loadings	3.90	3.00	2.97	2.33
Proportion Var	0.17	0.13	0.13	0.10
Cumulative Var	0.17	0.30	0.43	0.53
Proportion Explained	0.32	0.25	0.24	0.19
Cumulative Proportion	0.32	0.57	0.81	1.00

For peer review only

Modified scale (20-items including the smoking items)

Non-missing samples: 89

The overall KMO is 0.79, which is slightly below the recommended range (0.8 to 1).

The Bartlet's test of Sphericity is significant (X2 = 915.41, p-value < 0.001), indicating the sample adequacy for factor analysis.

EFA results

- The root mean square of the residuals (RMSR) is 0.06
- Tucker Lewis Index of factoring reliability = 0.72
- RMSEA index = 0.118 and the 90 % confidence intervals are 0.111 and 0.126
- BIC = 153.72

Scree plot

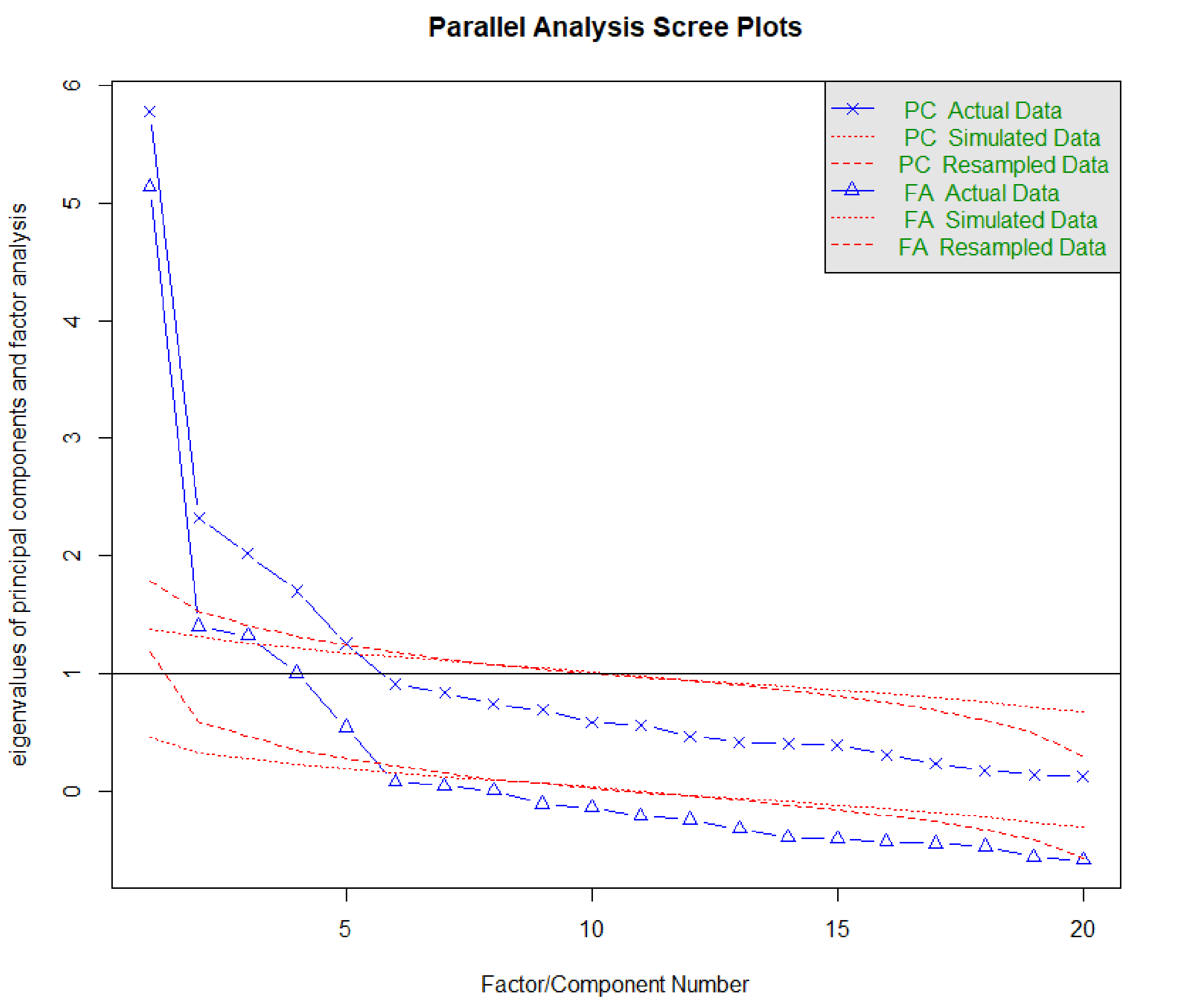

Figure 3. Modified ABCD Questionnaire 20 items with smoking.

Table A3 (a). Factor loadings of the exploratory factor analysis of the modified risk scale (20 items including the smoking items)

Items	Factor3	Factor1	Factor4	Factor2	Communality	Uniqueness
I feel I will suffer from a heart attack or stroke sometime during my life	-0.08	0.04	-0.03	0.76	0.60	0.40
There is a good chance I will experience a heart attack or stroke in the next 10 years	0.02	-0.08	-0.01	0.68	0.48	0.52
It is likely I will have a heart attack or stroke because of my past and/or present behaviors	-0.04	0.01	-0.01	0.61	0.38	0.62
I am not worried that I might have a heart attack or stroke (Reverse coded)	0.04	-0.13	0.10	0.35	0.14	0.86
I am concerned about the likelihood of having a heart attack or stroke in the near future	0.22	-0.11	0.07	0.45	0.23	0.77
I am thinking about exercising at least 2.5 hours a week	-0.06	0.88	-0.02	-0.04	0.74	0.26
I intend or want to exercise at least 2.5 hours a week	0.05	0.87	-0.02	-0.02	0.79	0.21
When I exercise for at least 2.5 hours a week I am doing something good for the health of my heart	0.17	0.62	0.09	0.04	0.55	0.45
I am confident that I can maintain a healthy weight by exercising at least 2.5 hours a week	0.09	0.42	0.18	-0.06	0.32	0.68
I am not thinking about exercising for 2.5 hours a week (Reverse coded)	0.01	0.53	0.09	0.03	0.32	0.68
When I eat five portions of fruit and vegetables a day I am doing something good for the health of my heart	0.08	0.35	0.35	0.07	0.37	0.63
Increasing my exercise to at least 2.5 hours a week will decrease my chances of having a heart attack or stroke	0.13	0.37	0.24	0.06	0.32	0.68
I am confident that I can eat at least five portions of fruit and vegetables a day within the next two months	-0.06	0.12	0.64	-0.05	0.46	0.54
I am thinking about eating at least five portions of fruit and vegetables a day	0.00	0.02	0.89	0.01	0.80	0.20
I am not thinking about eating at least five portions of fruit and vegetables a day (Reverse coded)	0.00	-0.06	0.83	-0.01	0.67	0.33
I am thinking of stopping smoking within two months	0.78	0.12	-0.06	0.04	0.66	0.34
I have reduced or stopped smoking	0.83	0.02	-0.01	-0.03	0.70	0.30
I intend or want to stop smoking	0.89	-0.02	-0.01	-0.07	0.80	0.20
If I stop smoking it will reduce my chances of having a heart attack or stroke	0.59	0.10	0.07	0.18	0.43	0.57
I am not thinking about stopping smoking	0.56	-0.20	0.17	-0.10	0.34	0.66

For peer review only

Table A3 (b): Summary of factor loadings and variance distribution of the modified risk scale (20 items including the smoking items)

Measures	Factor3	Factor1	Factor4	Factor2
SS loadings	3.00	2.96	2.33	1.80
Proportion Var	0.15	0.15	0.12	0.09
Cumulative Var	0.15	0.30	0.41	0.50
Proportion Explained	0.30	0.29	0.23	0.18
Cumulative Proportion	0.30	0.59	0.82	1.00

For peer review only

Appendix 6. Item Analysis of published ABCD Risk Questionnaire sub-scales plus 5 unpublished items relating to smoking.

**Perceived Risk of Heart Attack/ Stroke**<br>**8 Items**<br>**Cronbach's Alpha .861 (0.84,0.88) 95% CI**<br>**Omega 0.85 (0.83, 0.88) 95% CI**	**Inter-item correlation**	**Corrected Item-total correlation**	**Cronbach's alpha if item deleted**
It is likely that I will suffer from a heart attack or stroke in the future	**.832**	.756	.826
It is likely that I will have a heart attack or stroke some time during my life	**.869**	.777	.824
I feel I will suffer a heart attack or stroke some time during my life	**.616**	.784	.824
There is a good chance I will experience a heart attack or stroke in the next 10 years	**.729**	.722	.832
I am not worried that I might have a heart attack or stroke	.403	.624	.843
My chances of suffering a heart attack or stroke in the next 10 years are great	.245	.544	.852
It is likely that I will have a heart attack or stroke because of my past/present behaviours	.266	.319	**.876**
I am concerned about the likelihood of having a heart attack or stroke in the near future	.259	.387	**.870**
**Perceived Benefits and Intentions to Change**<br>**7 items**<br>**Cronbach's Alpha .801**<br>**Omega 0.82 (0.78, 0.85) 95% CI**	**Inter-item correlation**	**Corrected Item-total correlation**	**Cronbach's alpha if item deleted**
I am thinking about exercising at least 2.5 hours a week	**.727**	**.605**	.760
I intend or want to exercise at least 2.5 hours a week	.442	**.651**	.752
When I exercise for at least 2.5 hours a week I am doing something good for the health of my heart	.426	.593	.769
I am confident that I can maintain a healthy weight by exercising at least 2.5 hours a week within the next 2 months	.294	.452	.790

I am not thinking about exercising at least 2.5 hours a week	.264	.508	.781
When I eat at least 5 portions of fruit and vegetables a day I am doing something good for the health of my heart	.483	.483	.783
Increasing my exercise to at least 2.5 hours a week will decrease my chances of having a heart attack or stroke	.326	.474	.786
**Healthy Eating Intentions 3 items Cronbach's Alpha .787 (95% CI Omega 0.84 (0.81, 0.88) 95% CI**	**Inter-item correlation**	**Corrected Item-total correlation**	**Cronbach's alpha if item deleted**
I am confident that I can eat at least 5 portions of fruit and vegetables a day within the next 2 months	.555	.533	**.812**
I am thinking about eating at least 5 portions of fruit and vegetables a day	**.683**	**.732**	.596
I am not thinking about eating at least 5 portions of fruit and vegetables a day	.424	**.624**	.713
**Perceived Benefits and Intentions to Stop Smoking 5 Items Cronbach's Alpha .943 95% CI Omega 0.86 (0.81, 0.91) 95% CI**	**Inter-item correlation**	**Corrected item-total correlation**	**Cronbach's alpha if item deleted**
I am thinking of stopping smoking within the next 2 months	**.654**	.848	.932
I have reduced or stopped smoking	**.694**	.751	**.949**
I intend or want to stop smoking	**.829**	.906	.919
If I stop smoking it will reduce my chances of having a heart attack or stroke	**.834**	.886	.922
I am not thinking about stopping smoking	**.789**	.872	.925

Appendix 7. Modified ABCD Risk Questionnaire

Mark Bowyer, Hamid Hassen

Scale	Items	Coding
**Perceived Risk of Heart Attack or Stroke**	1. It is likely that I will have a heart attack or stroke sometime in my life	4= Strongly disagree, 3= Disagree, 2= Agree, 1= Strongly Agree; N/A= 0
	2. There is a good chance I will experience a heart attack or stroke in the next 10 years	4= Strongly disagree, 3= Disagree, 2= Agree, 1= Strongly Agree; N/A= 0
	3. It is (more) likely I will have a heart attack or stroke because of my past and/or present behaviours	4= Strongly disagree, 3= Disagree, 2= Agree, 1= Strongly Agree; N/A= 0
	4. I am not worried that I might have a heart attack or stroke	**REVERSE CODED** 4= Strongly disagree, 3= Disagree, 2= Agree, 1= Strongly Agree; N/A= 0
	5. I am concerned about the likelihood of having a heart attack or stroke in the near future	4= Strongly disagree, 3= Disagree, 2= Agree, 1= Strongly Agree; N/A= 0
**Perceived Benefits and Intentions to Exercise**	6. I am thinking about exercising at least 2.5 hours a week	4= Strongly disagree, 3= Disagree, 2= Agree, 1= Strongly Agree; N/A= 0
	7. I intend or want to exercise at least 2.5 hours a week	4= Strongly disagree, 3= Disagree, 2= Agree, 1= Strongly Agree; N/A= 0
	8. When I exercise for at least 2.5 hours a week I am doing something good for the health of my heart	4= Strongly disagree, 3= Disagree, 2= Agree, 1= Strongly Agree; N/A= 0
	9. I am confident that I can maintain a healthy weight by exercising at least 2.5 hours a week	4= Strongly disagree, 3= Disagree, 2= Agree, 1= Strongly Agree; N/A= 0
	10. I am not thinking about exercising for 2.5 hours a week	**REVERSE CODED** 4= Strongly disagree, 3= Disagree, 2= Agree, 1= Strongly Agree; N/A= 0
	11. Increasing my exercise to at least 2.5 hours a week will decrease my chances of having a heart attack or stroke	4= Strongly disagree, 3= Disagree, 2= Agree, 1= Strongly Agree; N/A= 0

For peer review only

**Perceived Benefit and Healthy Eating Intentions**	12. I am confident that I can eat at least five portions of fruit and vegetables a day within the next two months	4= Strongly disagree, 3= Disagree, 2= Agree, 1= Strongly Agree; N/A= 0
	13. I am thinking about eating at least five portions of fruit and vegetables a day	4= Strongly disagree, 3= Disagree, 2= Agree, 1= Strongly Agree; N/A= 0
	14. I am not thinking about eating at least five portions of fruit and vegetables a day	**REVERSE CODED** 4= Strongly disagree, 3= Disagree, 2= Agree, 1= Strongly Agree; N/A= 0
	15. When I eat five portions of fruit and vegetables a day I am doing something good for the health of my heart	4= Strongly disagree, 3= Disagree, 2= Agree, 1= Strongly Agree; N/A= 0
**Benefits and Intentions to Stop Smoking**	16. I am thinking of stopping smoking within two months	4= Strongly disagree, 3= Disagree, 2= Agree, 1= Strongly Agree; N/A= 0
	17. I have reduced or stopped smoking	4= Strongly disagree, 3= Disagree, 2= Agree, 1= Strongly Agree; N/A= 0
	18. I intend or want to stop smoking	4= Strongly disagree, 3= Disagree, 2= Agree, 1= Strongly Agree; N/A= 0
	19. If I stop smoking it will reduce my chances of having a heart attack or stroke	4= Strongly disagree, 3= Disagree, 2= Agree, 1= Strongly Agree; N/A= 0
	20. I am not thinking about stopping smoking	**REVERSE CODED** 4= Strongly disagree, 3= Disagree, 2= Agree, 1= Strongly Agree; N/A= 0

For peer review only

Reporting checklist for cross sectional study.

Based on the STROBE cross sectional guidelines.

Instructions to authors

Complete this checklist by entering the page numbers from your manuscript where readers will find each of the items listed below.

Your article may not currently address all the items on the checklist. Please modify your text to include the missing information. If you are certain that an item does not apply, please write "n/a" and provide a short explanation.

Upload your completed checklist as an extra file when you submit to a journal.

In your methods section, say that you used the STROBE cross sectionalreporting guidelines, and cite them as:

von Elm E, Altman DG, Egger M, Pocock SJ, Gotzsche PC, Vandenbroucke JP. The Strengthening the Reporting of Observational Studies in Epidemiology (STROBE) Statement: guidelines for reporting observational studies.

		Reporting Item	Page Number
**Title and abstract**			
Title	#1a	Indicate the study's design with a commonly used term in the title or the abstract	1
Abstract	#1b	Provide in the abstract an informative and balanced summary of what was done and what was found	1
**Introduction**			
Background / rationale	#2	Explain the scientific background and rationale for the investigation being reported	3
Objectives	#3	State specific objectives, including any prespecified hypotheses	3
**Methods**			
Study design	#4	Present key elements of study design early in the	4

For peer review only

		paper	
Setting	#5	Describe the setting, locations, and relevant dates, including periods of recruitment, exposure, follow-up, and data collection	4
Eligibility criteria	#6a	Give the eligibility criteria, and the sources and methods of selection of participants.	4
	#7	Clearly define all outcomes, exposures, predictors, potential confounders, and effect modifiers. Give diagnostic criteria, if applicable	6
Data sources / measurement	#8	For each variable of interest give sources of data and details of methods of assessment (measurement). Describe comparability of assessment methods if there is more than one group. Give information separately for for exposed and unexposed groups if applicable.	6
Bias	#9	Describe any efforts to address potential sources of bias	7
Study size	#10	Explain how the study size was arrived at	7
Quantitative variables	#11	Explain how quantitative variables were handled in the analyses. If applicable, describe which groupings were chosen, and why	7
Statistical methods	#12a	Describe all statistical methods, including those used to control for confounding	7
Statistical methods	#12b	Describe any methods used to examine subgroups and interactions	7
Statistical methods	#12c	Explain how missing data were addressed	7
Statistical methods	#12d	If applicable, describe analytical methods taking account of sampling strategy	7
Statistical methods	#12e	Describe any sensitivity analyses	7
**Results**			
Participants	#13a	Report numbers of individuals at each stage of study—	7

For peer review only

		eg numbers potentially eligible, examined for eligibility, confirmed eligible, included in the study, completing follow-up, and analysed. Give information separately for for exposed and unexposed groups if applicable.	
Participants	#13b	Give reasons for non-participation at each stage	7
Participants	#13c	Consider use of a flow diagram	n/a No drop-out
Descriptive data	#14a	Give characteristics of study participants (eg demographic, clinical, social) and information on exposures and potential confounders. Give information separately for exposed and unexposed groups if applicable.	7
Descriptive data	#14b	Indicate number of participants with missing data for each variable of interest	7
Outcome data	#15	Report numbers of outcome events or summary measures. Give information separately for exposed and unexposed groups if applicable.	7
Main results	#16a	Give unadjusted estimates and, if applicable, confounder-adjusted estimates and their precision (eg, 95% confidence interval). Make clear which confounders were adjusted for and why they were included	8
Main results	#16b	Report category boundaries when continuous variables were categorized	n/a Continuous variables not measured
Main results	#16c	If relevant, consider translating estimates of relative risk into absolute risk for a meaningful time period	n/a No measurement of risk
Other analyses	#17	Report other analyses done—e.g., analyses of subgroups and interactions, and sensitivity analyses	10
**Discussion**			
Key results	#18	Summarise key results with reference to study objectives	12
Limitations	#19	Discuss limitations of the study, taking into account	12

For peer review only

		sources of potential bias or imprecision. Discuss both direction and magnitude of any potential bias.	
Interpretation	#20	Give a cautious overall interpretation considering objectives, limitations, multiplicity of analyses, results from similar studies, and other relevant evidence.	12
Generalisability	#21	Discuss the generalisability (external validity) of the study results	13
**Other Information**			
Funding	#22	Give the source of funding and the role of the funders for the present study and, if applicable, for the original study on which the present article is based	1

Notes:

- 13c: n/a No drop-out
- 16b: n/a Continuous variables not measured
- 16c: n/a No measurement of risk The STROBE checklist is distributed under the terms of the Creative Commons Attribution License CC-BY. This checklist was completed on 08. June 2021 using https://www.goodreports.org/, a tool made by the EQUATOR Network in collaboration with Penelope.ai

For peer review only

BMJ Open

Psychometric evaluation of the 'Attitudes and Beliefs about Cardiovascular Disease (ABCD) Risk Questionnaire' with validation of a previously untested 'Intentions and Beliefs around Smoking' sub-scale.

Journal:	*BMJ Open*
Manuscript ID	bmjopen-2021-054532.R3
Article Type:	Original research
Date Submitted by the Author:	02-Sep-2022
Complete List of Authors:	Bowyer, Mark; Nottingham Trent University, Institute of Health and Allied Professions, School of Social Sciences<br>Hassen, Hamid; University of Antwerp, Family Medicine and Population Health, Faculty of Medicine and Health Services<br>Bastiaens, Hilde; University of Antwerp Faculty of Medicine and Health Sciences, Family Medicine and Population Health<br>Gibson, Linda ; Nottingham Trent University, Institute of Health and Allied Professions, School of Social Sciences
**Primary Subject Heading**:	Public health
Secondary Subject Heading:	Cardiovascular medicine, Smoking and tobacco
Keywords:	PUBLIC HEALTH, STATISTICS & RESEARCH METHODS, PREVENTIVE MEDICINE

SCHOLARONE™
Manuscripts

I, the Submitting Author has the right to grant and does grant on behalf of all authors of the Work (as defined in the below author licence), an exclusive licence and/or a non-exclusive licence for contributions from authors who are: i) UK Crown employees; ii) where BMJ has agreed a CC-BY licence shall apply, and/or iii) in accordance with the terms applicable for US Federal Government officers or employees acting as part of their official duties; on a worldwide, perpetual, irrevocable, royalty-free basis to BMJ Publishing Group Ltd ("BMJ") its licensees and where the relevant Journal is co-owned by BMJ to the co-owners of the Journal, to publish the Work in this journal and any other BMJ products and to exploit all rights, as set out in our licence.

The Submitting Author accepts and understands that any supply made under these terms is made by BMJ to the Submitting Author unless you are acting as an employee on behalf of your employer or a postgraduate student of an affiliated institution which is paying any applicable article publishing charge ("APC") for Open Access articles. Where the Submitting Author wishes to make the Work available on an Open Access basis (and intends to pay the relevant APC), the terms of reuse of such Open Access shall be governed by a Creative Commons licence – details of these licences and which Creative Commons licence will apply to this Work are set out in our licence referred to above.

Other than as permitted in any relevant BMJ Author's Self Archiving Policies, I confirm this Work has not been accepted for publication elsewhere, is not being considered for publication elsewhere and does not duplicate material already published. I confirm all authors consent to publication of this Work and authorise the granting of this licence.

For peer review only

TITLE PAGE

Psychometric evaluation of the 'Attitudes and Beliefs about Cardiovascular Disease (ABCD) Risk Questionnaire' with validation of a previously untested 'Intentions and Beliefs around Smoking' sub-scale.

Mark Bowyer, Nottingham Trent University (Corresponding Author)

mark.bowyer@ntu.ac.uk (**ORCID** 0000-0002-1474-5711)

Nottingham Trent University School of Social Sciences

Chaucer Building, Burton Street

Nottingham NG1 4BT

Tel: (+1) 7786 993405 Fax: (+1)115 8485574

Hamid Yimam Hassen, University of Antwerp, Belgium

hamid.hassen@uantwerpen.be (**ORCID** 0000-0001-6485-4193)

Dr Hilde Bastiaens (Participating Investigator)
Associate professor
Dept Family Medicine and Population Health
Faculty of Medicine and Health Sciences
University of Antwerp

Tel: 0032 (0)3 265.29.10 Fax: 0032 (0)3 265.25.26 Hilde.bastiaens@uantwerpen.be

Dr Linda Gibson (Participating Investigator)

Professor in Public Health, Institute of Health & Allied Professions

Nottingham Trent University Linda.gibson@ntu.ac.uk

Key words

Cardiovascular Diseases

- Cardiovascular risk factors
- Instrumentation

Psychometrics

- Surveys and questionnaires
- Instrumentation

Primary prevention

- Instrumentation

Word count 4092

For peer review only - http://bmjopen.bmj.com/site/about/guidelines.xhtml

Psychometric evaluation of the 'Attitudes and Beliefs about Cardiovascular Disease (ABCD) Risk Questionnaire' with validation of a previously untested 'Intentions and Beliefs around Smoking' sub-scale.

ABSTRACT

Objectives:

To provide evidence of validity, reliability and generalisability of results obtained using the Attitudes and Beliefs about Cardiovascular Disease (ABCD) Risk Questionnaire with a sample of the English population surveyed within the 'SPICES' Horizon 2020 project (Nottingham study site), and to specifically evaluate the psychometric and factor properties of an as-yet untested 5 item sub-scale relating to smoking behaviours.

Design and setting:

Community and workplace-based cross-sectional study in Nottingham, UK.

Participants:

466 English adults fitting inclusion criteria (aged 18+, without known history of CVD, not pregnant, able to provide informed consent) participated in the study.

Methods:

We re-validated the ABCD questionnaire on a sample of the general population in Nottingham to confirm the psychometric properties. Furthermore, we introduced 5 items related to smoking which were dropped in the original study due to inadequate valid samples.

Primary and secondary outcome measures:

1. Psychometric and factor performance of untested 5 item 'smoking behaviours' sub-scale
2. Psychometric and factorial properties in combination with the remaining 18 items across 3 sub-scales

Results:

Analyses of the data largely confirmed the validity, reliability, and factor structure of the original ABCD Risk Questionnaire. Sufficient participants in our study provided data against an additional five smoking related items to confirm their validity as a sub-scale and to advocate for their inclusion in future applications of the scale. EFA and CFA calculations support some minor changes to the remaining sub-scales which may further improve psychometric performance and therefore generalisability of the instrument.

Conclusions:

An amended version of the ABCD Risk Questionnaire would provide public health researchers and practitioners with a brief, easy to use, reliable and valid survey tool. The amended tool may assist public health practitioners and researchers to survey patient or public intentions and beliefs around three key areas of individually modifiable risk (Physical Activity, Diet, Smoking).

For peer review only - http://bmjopen.bmj.com/site/about/guidelines.xhtml

Trial registration:

ISRCTN68334579 https://doi.org/10.1186/ISRCTN68334579
Heart health without a doctor: an implementation study of CVD prevention and behaviour change interventions in community settings

Ethical approval

Ethical approval for the 'SPICES' Nottingham study protocol (incorporating the ABCD Risk Questionnaire) was secured from the Nottingham Trent University College of Business, Law and Social Sciences on the 20th February 2019. Participants were required to provide informed consent (Appendix 1).

Article summary

Strengths and Limitations of this study

- Large sample (n=466) of English adults from the Nottingham UK population
- Sufficient case data to validate additional sub-scale related to attitudes and intentions of smokers
- Criterion validity not explored
- Full assessment of the utility of ABCD Risk Questionnaire in health promotion and CVD prevention not explored; further studies may be required to position the tool in clinical and public health practice.
- The planned pre-post intervention measurement and analysis was not possible due to COVID-19 interruption of fieldwork.

Original protocol (Appendix 2)

Funding statement

This work was supported by the European Commission Horizon 2020 Non-communicable diseases and the challenge of healthy ageing Grant agreement 733356 'SPICES'.

Competing interests statement

None declared

Patient and public involvement

Patients and/or the public were not involved in the design, or conduct, or reporting, or dissemination plans of this research.

Patient consent for publication (data sharing agreement)

Not required (participant information and informed consent attached Appendix 1)

Provenance and peer review

Not commissioned.

Data availability statement

Data are available on reasonable request

For peer review only - http://bmjopen.bmj.com/site/about/guidelines.xhtml

Keywords

Cardiovascular diseases- Cardiovascular risk factors

Cardiovascular diseases- Instrumentation

Psychometrics- Instrumentation

Surveys and questionnaires- Instrumentation

Primary prevention- Instrumentation

Author contributions

Following ICMJE recommendations, Mark Bowyer and Hamid Hassen assert authorship based on the following 4 criteria:

Substantial contributions to the conception or design of the work; or the acquisition, analysis, or interpretation of data for the work; AND

Drafting the work or revising it critically for important intellectual content; AND

Final approval of the version to be published; AND

Agreement to be accountable for all aspects of the work in ensuring that questions related to the accuracy or integrity of any part of the work are appropriately investigated and resolved.

Professor Linda Gibson and Professor Hilde Bastiaens assert Participating Investigator status having served as scientific advisors, critically reviewed the study proposal, and participated in writing or technical editing of the manuscript.

Acknowledgements

The authors would like to acknowledge the cooperation of Rolls-Royce plc Hucknall Site; Nottingham City Council Adult Care in providing access to employees. Crabtree Farm Community Centre, Middle Street Resource Centre, Self-Help UK, in facilitating access to members, users and premises.

INTRODUCTION

Scientific Background and Rationale

In the UK, Cardiovascular Disease (CVD) is responsible for over 130,000 deaths per annum.[1] CVD morbidity is also the biggest contributor to the inequalities in Healthy Life Expectancy between members of the wealthiest neighbourhoods and the most deprived.[2] In 2009 the NHS Health Check [3]was established and more recently (2019) the CVD Prevent initiative to implement 'upstream' interventions for the prevention of CVD morbidity.[4] Both of these initiatives seek to

For peer review only - http://bmjopen.bmj.com/site/about/guidelines.xhtml

improve early case-finding to prevent avoidable strokes and heart attacks. Both recognise the importance of supported lifestyle change in conjunction with drug therapies.

Lifestyle or behavioural change requires a degree of individual agency and commitment which drug therapies do not. Unhealthy lifestyle behaviours are linked to culture and habit, environment, emotions, and confidence which can all moderate an individual's readiness to change and the commitment required to sustain those changes over time.[5] Understanding the attitudes and beliefs that people hold towards diet, exercise and smoking, as well as their perception of their own risk could assist primary care and public health professionals in providing relevant and effective behavioural advice and social prescribing options. To support evaluations of the NHS Health Check programme, in 2017 a questionnaire was developed to evaluate patients' awareness of cardiovascular disease risk at University College London.[6] This ABCD Risk Questionnaire attempts to provide a short survey drawing from the dominant theoretical models of behaviour change (Trans-Theoretical Model, Health Beliefs Model),[7] covering diet, smoking, exercise and alcohol behaviours, and incorporating a conceptual spread of perceived risk from immediate to lifetime. Whilst a range of validated CVD risk questionnaires exist,[8] and it is common to ask patients to self-report their physical activity, dietary and smoking behaviours through questionnaires and diaries, the ABCD Risk Questionnaire usefully investigates the knowledge, perceptions, beliefs and attitudes that govern these behaviours. To confirm the reliability and generalisability of the ABCD Risk Questionnaire, it was necessary to replicate the study methods with a new, larger independent data-set.[9]

Specific Objectives

In this study we re-validated the tool on a sample of the general population in Nottingham to confirm the psychometric properties. Furthermore, we introduced 5 items related to smoking which were dropped in the original study due to inadequate case numbers.

To the best of our knowledge, this is the first study which has incorporated items relating to attitudes and intentions towards stopping smoking into the published version of the ABCD Risk Questionnaire and collected sufficient data to submit them to analysis of validity, reliability and factor structure.

In the original ABCD study, over the course of three stages of validity testing (content, face, reliability) items relating to alcohol use and smoking were rejected, leaving four final sub-scales: Knowledge of CVD Risks; Perceived Risk of Heart Attack/ Stroke; Perceived Benefits and Intentions to Change; and Healthy Eating Intentions. During Exploratory Factor Analysis (EFA) none of the items relating to alcohol use achieved strong enough loadings to be included in the final scale, and items related to smoking could not be included due to the high proportion of missing data in the experimental sample. The authors of the study note this limitation '*the questionnaire does not encompass all aspects of CVD risk observed in the general population*' and that '*future studies examining populations at increased CVD risk can look into incorporating smoking and alcohol into the ABCD Risk Questionnaire to learn about these individuals' preconceptions and attendance of follow-up care*'.[10]

The present study

Nottingham is one of five global sites of the EU Horizon 2020 'SPICES' [11] CVD prevention implementation study which began in 2017. SPICES investigates contextual and health system barriers to the scaling up of successful behaviour change interventions for improved cardiovascular health in low, middle and high income European countries. The most recent data (2016) indicate

For peer review only - http://bmjopen.bmj.com/site/about/guidelines.xhtml

that "The prevalence of CVD recorded in Nottingham City GP Practices is significantly less that the national (England) average and in comparable areas, despite the CVD mortality rate being significantly higher than average; this partly reflects the differing age structures of the populations, but also indicates significant under-detection/diagnosis"[12]

The SPICES Nottingham population survey carried out in 2019-20 utilised the ABCD Risk Questionnaire alongside the non-clinical INTERHEART CVD risk prediction instrument.[13] The SPICES study team chose to re-introduce 5 pre-written items relating to 'Intentions and Readiness to Stop Smoking' from the 65 item University College London (UCL) item pool into the questionnaire due to the high prevalence of smoking in the Nottingham population compared to England averages,[14] and its importance as a CVD risk.[15] This created a 31 item questionnaire. 4 items relating to Alcohol intake from the same item pool were also considered for inclusion but omitted on two grounds: alcohol related CVD risk was not a specific focus of the 'SPICES' study; concerns about the time-burden on participants of including the additional items which can be a barrier to participation.

In so doing, NTU researchers attempted to *'replicate the factor analytic process on an independent, larger sample to confirm the generalisability of (the original) findings'* as requested by the authors of the original study.[16] At the same time, we anticipated securing sufficient responses against the reintroduced 5 item 'smoking' sub-scale to analyse its reliability and validity as an integral part of future versions of the Questionnaire.

METHODS

Incorporating the ABCD Risk Questionnaire into the SPICES Nottingham baseline survey provided cross-sectional study data across a broad sample of adult participants. The data-set generated was therefore suitable for psychometric validation of the original and modified versions of the ABCD questionnaire. Surveys were administered in-person by researchers in the field during attendance at community venues and workplaces. Administration of the survey took approximately ten minutes including provision of consent, and confidential communication of results another ten minutes on average. Participation was entirely voluntary.

Patient and public involvement

Patients and/or the public were not involved in the design, or conduct, or reporting, or 31 dissemination plans of this research.

Participants

Participants were recruited from across the Nottingham conurbation between April 2019 and March 2020 as part of the SPICES Nottingham baseline survey.[17] A purposive sampling method was employed based on community engagement. This strategy had two components:

1. engagement of citizens in neighbourhoods through existing community groups, organisations and venues, and
2. engagement of employees in the workplace through large city-based employers.

Community groups were targeted on the basis of the demographic of their membership to ensure that neighbourhoods of differing mean household income, those who are not in employment or of working age, and those from different ethnicities were included. In this way 327 participants were recruited.

For peer review only - http://bmjopen.bmj.com/site/about/guidelines.xhtml

Employers were targeted on the basis of workforce size, and policies relating to workforce well-being. Nottingham City Council Adult Care teams and the Rolls-Royce plc Hucknall site both responded positively and between them provided 156 participants. NTU researchers administered the SPICES Nottingham baseline survey individually within the community or workplace setting and personalised feedback about CVD risks was provided confidentially once the survey had been completed.

Criteria for inclusion included being aged 18+, resident in Nottinghamshire, not previously diagnosed with a heart condition, not pregnant, and able to provide informed consent.

Materials

The SPICES baseline survey incorporated the ABCD risk questionnaire into a digitised survey instrument created in the Research Electronic Data Capture (REDCap) database system,[18] a secure web application for building and managing online surveys and databases, and the online survey responses were uploaded automatically. No participant data was stored on local devices. Both the ABCD Risk Questionnaire (Table 1) and the non-laboratory INTERHEART questionnaire were included unchanged from their published versions apart from an additional 5 items pertaining to smoking behaviour (Table 2).[19]

Table 1. Published ABCD Risk Questionnaire

Scale	Items
**Knowledge**<br><br>True/False/Don't Know<br><br>Correct score =1<br>Incorrect/ Don't know score = 0<br><br>Higher sum score= more knowledgeable/ more correct about having a heart attack or stroke	1. One of the main causes of heart attack and stroke is stress
	2. Walking and gardening are considered types of exercise that can lower the risk of having a heart attack or stroke
	3. Moderately intense activity of 2.5 hours a week will reduce your chances of having a heart attack or stroke
	4. People who have diabetes are at higher risk of heart attack or stroke
	5. Managing your stress levels will help you to manage your blood pressure
	6. Drinking high levels of alcohol can increase your cholesterol and triglyceride levels
	7. HDL refers to 'good' cholesterol, and LDL refers to 'bad' cholesterol
	8. A family history of heart disease is not a risk factor for high blood pressure
**Perceived Risk of Heart Attack or Stroke**<br><br>4= Strongly disagree, 3= Disagree, 2= Agree, 1= Strongly Agree; N/A= 0<br><br>Higher sum score = higher perception of risk of having a heart attack or stroke	9. I feel I will suffer from a heart attack or stroke sometime during my life
	10. It is likely that I will suffer from a heart attack or stroke in the future
	11. It is likely that I will have a heart attack or stroke some time during my life
	12. There is a good chance I will experience a heart attack or stroke in the next 10 years
	13. My chances of suffering from a heart attack or stroke in the next 10 years are great
	14. It is likely I will have a heart attack or stroke because of my past and/or present behaviours

For peer review only - http://bmjopen.bmj.com/site/about/guidelines.xhtml

	15. I am not worried that I might have a heart attack or stroke (**Reverse coded**)
	16. I am concerned about the likelihood of having a heart attack or stroke in the near future
**Perceived Benefits and Intentions to Change**<br><br>4= Strongly disagree, 3= Disagree, 2= Agree, 1= Strongly Agree; N/A= 0<br><br>Higher average score = Higher perceived benefits of diet and exercise and higher perceived readiness for change in regards to exercise and behaviour	17. I am thinking about exercising at least 2.5 hours a week
	18. I intend or want to exercise at least 2.5 hours a week
	19. When I exercise for at least 2.5 hours a week I am doing something good for the health of my heart
	20. I am confident that I can maintain a healthy weight by exercising at least 2.5 hours a week
	21. I am not thinking about exercising for 2.5 hours a week **(Reverse coded)**
	22. When I eat five portions of fruit and vegetables a day I am doing something good for the health of my heart
	23. Increasing my exercise to at least 2.5 hours a week will decrease my chances of having a heart attack or stroke
**Healthy Eating Intentions**<br><br>4= Strongly disagree, 3= Disagree, 2= Agree, 1= Strongly Agree; N/A= 0<br><br>Higher average score = Higher perceived readiness for change with regard to healthy dietary behaviour	24. I am confident that I can eat at least five portions of fruit and vegetables a day within the next two months
	25. I am thinking about eating at least five portions of fruit and vegetables a day
	26. I am not thinking about eating at least five portions of fruit and vegetables a day **(Reverse coded)**

The surveys were administered in the field by a team of trained researchers recruited from the NTU student body and directly supervised by the SPICES Nottingham coordinator. The surveys were accessed using dedicated tablet computers. Items were reproduced word for word and in the same sequence as the original ABCD Risk Questionnaire with the additional 5 smoking items inserted after all 26 original items. The five smoking related items were developed by the authors of the original study through a process of literature review (construct validity), expert panel review (content validity), and modification by focus group (face validity). [20] These five smoking sub-scale items were included in the 65 item pool developed in the original study but omitted from their analysis due to a high proportion of missing responses.[21]

Table 2. Additional 'smoking' sub-scale

**Benefits and Intentions to Stop Smoking**<br><br>4= Strongly disagree, 3= Disagree, 2= Agree, 1= Strongly Agree; N/A= 0<br><br>Higher average score = Higher	27. I am thinking of stopping smoking within two months
	28. I have reduced or stopped smoking
	29. I intend or want to stop smoking
	30. If I stop smoking it will reduce my chances of having a heart attack or stroke
	31. I am not thinking about stopping smoking

For peer review only - http://bmjopen.bmj.com/site/about/guidelines.xhtml

perceived readiness for change with regard to healthy dietary behaviour	

Validating the sample

The baseline survey dataset was extracted from REDCap for analysis. Sample was checked for representativeness of the Nottingham population across parameters of age, gender, household income and known rates of physical activity and smoking.

Data analysis

We took the published 26-item ABCD Risk Questionnaire, introduced 5 further items relating to smoking behaviours, and administered it alongside a validated CVD risk assessment instrument (INTERHEART) to 486 individuals in Nottingham over a period of 12 months. Item, scale, and factor reliabilities were remeasured to generate a comparison to the results reported in the original study. Correlation was tested between and amongst ABCD sub-scale scores and selected INTERHEART variables, closely matching the methods applied in the original study (Appendix 3) and results were compared accordingly. After removing incomplete responses, 466 valid cases were entered for analysis, four times the sample size of the original study.

Item and sub-scale reliabilities were tested using inter-item correlations, corrected item-total correlations and Cronbach's Alpha. [22] We performed an exploratory factor analysis (EFA) to evaluate the dimensionality of items of the original and modified risk scale with and without the smoking items. The EFA was performed using the maximum likelihood extraction and varimax rotation method. [23] Sample and data adequacy was assessed using Kaiser-Meyer-Olkin (KMO) test and Bartlett's test of sphericity was performed to compare an observed correlation matrix to the identity matrix.[24] The adequate number of factors was determined using a scree plot. To further test the consistency of factors, we tested using Confirmatory Factor Analysis (CFA). We evaluated the model fit of the CFA using; the X2 test, the Tucker-Lewis and Comparative Fit Indexes and the root mean square error of approximation (RMSEA).[25] The analysis was performed using a free statistical software R version 4.0.2. UK postcodes were collected for all participants which allowed them to be sorted into income deciles using Office for National Statistics Index of Multiple Deprivation (IMD) public datasets, allowing correlations to be analysed. Following the methods used in the original study, case data from the 'Knowledge' sub-scale (8 items) were omitted from the analysis since they utilise a separate response format.[26]

We used the STROBE cross sectional checklist when writing our report.[27]

RESULTS

Participants

Participation was voluntary, and self-selection may have been influenced by sensitivities around disclosure of health status and lifestyle habits forming a barrier to those with co-morbidities and socially 'questionable' behaviours (heavy smoking, high alcohol intake).

For peer review only - http://bmjopen.bmj.com/site/about/guidelines.xhtml

The sample cohort has a 49:51 percent gender split, normal distribution of age ranges (18-92), and a distribution of Socio-Economic Status (SES) which reflects known data about neighbourhood income in Nottingham. Nottingham is the 11th most deprived district in England with higher unemployment, lower education and skills, and shorter life expectancy than the national averages. [28] Using the Index of Multiple Deprivation a relative measure of deprivation across seven domains, Health and Disability is the domain on which the city's scores are lowest compared to the rest of England. Nevertheless, the mean INTERHEART predicted risk score for all 466 participants was 10.32 which closely matches the global reported mean for the instrument.[29]

Smoking sub-scale

The percentage of smokers in our sample was 15.5%. The number of smokers in our sample was therefore higher than the 2019 England average (13.9%), and lower than the Nottingham city population average (20.6%) based on the ONS Annual Population Survey.[30] ONS notes that smoking prevalence estimates by local authority can fluctuate due to smaller sample sizes. Our SPICES Nottingham sample cohort also includes some participants from neighbouring Local Authorities with different recorded rates of smoking.

The five items in the smoking subscale are measured on the same four-point response scale as the 18 items submitted for Factor Analysis in the original published ABCD Risk Questionnaire (Strongly agree, agree, disagree, strongly disagree, and not applicable).

With the original 18 items this 'Not Applicable' response option was not used by any of the SPICES Nottingham study participants. By contrast, within their responses to the items in the 'smoking' subscale, 'Not Applicable' was the modal answer. Participants chose the 'N/A' response option whenever they reported being a non-smoker. This mirrors the behaviour of the original 110 NHS Health Check attendees who formed the pilot sample cohort for the original study, leaving an insufficient proportion of smokers in the sample to assess validity and reliability of smoking sub-scale items. In the present study, 88 cases were found where participants reported smoking behaviours and this was sufficient to enter them into analysis.

Sub-scale Alpha values, Cronbach's Alpha if item deleted calculated for all items, inter-item correlations and corrected item-total correlations were all calculated, mirroring the analysis reported in the original study (Appendix 4).

Interitem correlations calculated for these five items produced a range between 0.654 and 0.834. All of these five 'smoking' items therefore correlate with one another more strongly than recommended (<.6) and were considered for rejection. However, we found each item to be qualitatively different, and that the differences were conceptually clear and well expressed in the item wording so that no participant could be expected to confuse one with any other, and they were retained.

Discrimination was confirmed using item-total correlations. These fell between the range 0.751 and 0.906 meaning that all five 'smoking' sub-scale items are comfortably above the standard cut-off for acceptability of 0.3.

EFA was carried out twice, firstly with all cases, and then again with 88 confirmed smoking cases. The first operation ensured that factor loadings were not skewed by the lower number of cases reporting smoking behaviours, the second ensured that factor loadings for the remaining sub-scales where more case data was available were not skewed by outliers.

Exploratory Factor Analysis:

For peer review only - http://bmjopen.bmj.com/site/about/guidelines.xhtml

We conducted EFA on the original 18-item risk perception questionnaire and the modified 23-item (with smoking items). For the original 18-item, a total of 420 observations were included in the analysis, which was sufficient for factor analysis as indicated with KMO of 0.82, which is within the recommended range (0.8 to 1). The Bartlett's Test of Sphericity was significant (X2 = 4235.007, p-value < 0.001) indicating the data is adequate for factor analysis. As a result, a three-factor solution emerged based on the Scree plot (figure 1), accounting 57.4% of the total variance. Factor loading patterns in the present analysis slightly varied from the original subscales. The domains in the original subscales were risk perception, benefit finding and healthy eating intentions. In our analysis, Item 14 (‘*When I eat at least 5 portions of fruit and vegetables a day I am doing something good for the health of my heart’*) showed a better loading to healthy eating intention, which was loaded to benefit finding in the original study (Appendix 5).

For the modified 23-item (including the smoking sub-scale), 88 samples were valid and included in the analysis. The KMO was 0.78, which was slightly below the recommended range, but Bartlett’s Test of Sphericity was significant (X2 = 1223.459, p-value < 0.001), indicating adequacy for factor analysis. The analysis showed that the smoking items loaded to another latent construct resulting in four factors in total (figure 2).

Confirmatory Factor Analysis of the published ABCD Risk Questionnaire

A Confirmatory Factor Analysis was undertaken using the SPICES Nottingham dataset to investigate further. Conducting CFA allowed us to construct the sub-scales of the published ABCD Risk Questionnaire in a three-factor measurement model and test its fit against relevant indices. Original 18 item survey comprising three sub-scales (Perceived Risk of Heart Attack/Stroke 8 items; Perceived Benefits and Intentions to Change 7 items; Healthy Eating Intentions 3 items) were used to create measurement model in SPSS Amos 25. The model was then updated to include an additional 5 item sub-scale relating to smoking behaviours.

Editing the measurement model

The CFA measurement model was then reconstructed removing items which had confused participants and generated high inter-item correlations, and additionally re-assigning an item relating to dietary behaviour into the dietary behaviour sub-scale (Table 3). This resulted in a four-factor model (Perceived Risk of Heart Attack/ Stroke’ 6 items; ‘Perceived Benefits and Intentions to Exercise’ 6 items; ‘Healthy Eating Intentions’ 4 items, Perceived Benefits and Intentions to Reduce Smoking’ 5 items). Analysis properties were set to Estimation: Maximum Likelihood. A scree-plot of this amended four-factor version of the questionnaire was also plotted (Figure 3).

For peer review only - http://bmjopen.bmj.com/site/about/guidelines.xhtml

Table 3. CFA fit indices for the original and modified ABCD Questionnaire measurement models

Original 18 item ABCD

In the original study of 2017, 18 items were entered into factor analysis. This Confirmatory Factor Analysis tests the fit of these original items to their structure using the larger Nottingham SPICES dataset.

CMIN	P	CMIN/DF	TLI	CFI	RMSEA	RMR
714.941	.000	5.416	.826	.850	.097	.049

Original 18 item ABCD with 5 Smoking items added

In the original study of 2017, items relating to smoking behaviours were developed but could not be included in the published scale due to insufficient data. In the Nottingham SPICES study sufficient observations were made to test these smoking items.

CMIN	P	CMIN/DF	TLI	CFI	RMSEA	RMR
994.931	.000	4.442	.865	.881	.086	.049

Edited 20 item ABCD with Smoking sub-scale

As discussed above, independent item analysis and Exploratory factor Analysis using the independent SPICES Nottingham dataset revealed issues with the continued inclusion of some of the original 'perception of risk' sub-scale items, and the allocation of an item relating to dietary behaviours in the physical activity behaviours sub-scale. The published ABCD questionnaire was edited to remove or re-assign the problematic items and retested using Confirmatory Factor Analysis.

CMIN	P	CMIN/DF	TLI	CFI	RMSEA	RMR
638.973	.000	3.896	.881	.897	.079	.052

Modified 20 item ABCD with Smoking sub-scale

The measurement model created for the Confirmatory Factor Analysis was modified so that items within each ABCD sub-scale were set to co-vary with one another.

CMIN	P	CMIN/DF	TLI	CFI	RMSEA	RMR
385.312	.000	2.439	.941	.951	.056	.046

For peer review only

Similarly, in the 23-item factor analysis, item 14 was loaded to the healthy eating intention. The model fit indices showed a slight improvement as indicated in table 3.

Based on factor loading, inter-item correlations, and face validity results, we also tested a slightly shorter version of the questionnaire, 20-items including five smoking items and the result shows that the model fit improved (CFI=0.941; TLI=0.951; RMSEA=0.056, SRMR=0.046).

The three published factors achieved a poor fit in CFA (Table 3). Including the five smoking related items which had performed strongly in EFA as their own latent factor improved overall model fit slightly, but not to an acceptable level.

Modification of the measurement model

Reviewing modification indices and expected parameter changes for factor loadings and measurement intercepts we observed an extreme covariance value (116.812) and parameter change (.209) between two of the risk perception items ('there is a good chance that I will experience a heart attack or stroke in the next 10 years' and 'my chances of suffering a heart attack or stroke in the next 10 years are great') which had caused confusion for participants in our study.

Removing one of these two items (item #13), and the two other duplicative items (items #9 & #10) from the 'perceived risk of heart attack or stroke' sub-scale retains the conceptual spread of risk embodied by the items (lifetime, 10 year, near future, behaviour related). Moving the diet related item (#22) which appears in the 'perceived benefits and intentions to change' over to the 'healthy eating intentions' sub-scale might allow greater clarity for researchers analysing results from the questionnaire. Co-varying items within sub-scales that generated values above 20 (a high cut-off due to large sample used) resulted in acceptable or good fit across all sub-scales. Each of the three behaviour related sub-scales now contain items drawn from HBM, TTM and SE models providing a sound conceptual basis for comparison. Using EFA to check these results shows the modified sub-scale structure performs better than the published version (Figure 3).

Table 4. Amended ABCD Risk Questionnaire

Scale	Items	Coding
**Knowledge**	1. One of the main causes of heart attack and stroke is stress	Correct answers:<br><br>Q1 - T<br><br>Q2 – T<br><br>Q3 – T<br><br>Q4 – T<br><br>Q5 – T<br><br>Q6 – T<br><br>Q7 – T<br><br>Q8 – F<br><br>T = True<br>F = False<br><br>Correct score = 1,<br><br>Incorrect or Don't Know: score = 0.
	2. Walking and gardening are considered types of exercise that can lower the risk of having a heart attack or stroke	
	3. Moderately intense activity of 2.5 hours a week is enough to reduce your chances of having a heart attack or stroke	
	4. People who have diabetes are at higher risk of having a heart attack or stroke	
	5. Managing your stress levels will help you to manage your blood pressure	
	6. Drinking high levels of alcohol can increase your cholesterol and triglyceride levels	
	7. HDL refers to 'good' cholesterol, and LDL refers to 'bad' cholesterol	
	8. A family history of heart disease is not a risk factor for high blood pressure	

For peer review only

**Perceived Risk of Heart Attack or Stroke**	9. It is likely that I will have a heart attack or stroke sometime in my life	4= Strongly disagree, 3= Disagree, 2= Agree, 1= Strongly Agree; N/A= 0
	10. There is a good chance I will experience a heart attack or stroke in the next 10 years	4= Strongly disagree, 3= Disagree, 2= Agree, 1= Strongly Agree; N/A= 0
	11. It is more likely I will have a heart attack or stroke because of my past and/or present behaviours	4= Strongly disagree, 3= Disagree, 2= Agree, 1= Strongly Agree; N/A= 0
	12. I am not worried that I might have a heart attack or stroke	**REVERSE CODED** 4= Strongly disagree, 3= Disagree, 2= Agree, 1= Strongly Agree; N/A= 0
	13. I am concerned about the likelihood of having a heart attack or stroke in the near future	4= Strongly disagree, 3= Disagree, 2= Agree, 1= Strongly Agree; N/A= 0
**Perceived Benefits and Intentions to Exercise**	14. I am thinking about exercising at least 2.5 hours a week	4= Strongly disagree, 3= Disagree, 2= Agree, 1= Strongly Agree; N/A= 0
	15. I intend or want to exercise at least 2.5 hours a week	4= Strongly disagree, 3= Disagree, 2= Agree, 1= Strongly Agree; N/A= 0
	16. When I exercise for at least 2.5 hours a week I am doing something good for the health of my heart	4= Strongly disagree, 3= Disagree, 2= Agree, 1= Strongly Agree; N/A= 0
	17. I am confident that I can maintain a healthy weight by exercising at least 2.5 hours a week	4= Strongly disagree, 3= Disagree, 2= Agree, 1= Strongly Agree; N/A= 0
	18. I am not thinking about exercising for 2.5 hours a week	**REVERSE CODED** 4= Strongly disagree, 3= Disagree, 2= Agree, 1= Strongly Agree; N/A= 0
	19. Increasing my exercise to at least 2.5 hours a week will decrease my chances of having a heart attack or stroke	4= Strongly disagree, 3= Disagree, 2= Agree, 1= Strongly Agree; N/A= 0
**Perceived Benefit and Healthy Eating Intentions**	20. I am confident that I can eat at least five portions of fruit and vegetables a day within the next two months	4= Strongly disagree, 3= Disagree, 2= Agree, 1= Strongly Agree; N/A= 0
	21. I am thinking about eating at least five portions of fruit and vegetables a day	4= Strongly disagree, 3= Disagree, 2= Agree, 1= Strongly Agree; N/A= 0

For peer review only - http://bmjopen.bmj.com/site/about/guidelines.xhtml

	22. I am not thinking about eating at least five portions of fruit and vegetables a day	**REVERSE CODED** 4= Strongly disagree, 3= Disagree, 2= Agree, 1= Strongly Agree; N/A= 0
	23. When I eat five portions of fruit and vegetables a day I am doing something good for the health of my heart	4= Strongly disagree, 3= Disagree, 2= Agree, 1= Strongly Agree; N/A= 0
**Benefits and Intentions to Stop Smoking**	24. I am thinking of stopping smoking within two months	4= Strongly disagree, 3= Disagree, 2= Agree, 1= Strongly Agree; N/A= 0
	25. I have reduced or stopped smoking	4= Strongly disagree, 3= Disagree, 2= Agree, 1= Strongly Agree; N/A= 0
	26. I intend or want to stop smoking	4= Strongly disagree, 3= Disagree, 2= Agree, 1= Strongly Agree; N/A= 0
	27. If I stop smoking it will reduce my chances of having a heart attack or stroke	4= Strongly disagree, 3= Disagree, 2= Agree, 1= Strongly Agree; N/A= 0
	28. I am not thinking about stopping smoking	**REVERSE CODED** 4= Strongly disagree, 3= Disagree, 2= Agree, 1= Strongly Agree; N/A= 0

Other results

Analysing results from ABCD sub-scales recorded within our sample indicated that mean knowledge of CVD risk factors was 79% and recognition of the benefits of changing behaviour was 85%, but this barely correlated against objectively measured risk (-.164, sig .001 n=436).

DISCUSSION

Inadequate knowledge and/or a gap between perceived and actual CVD risk in the population could be an obstacle to better health outcomes. Improving an individual's CVD knowledge and risk perception may be important in improving a healthy lifestyle. Measuring CVD knowledge and risk perception may be a method to initiate a healthy lifestyle intervention as well as to monitor and evaluate the impact of interventions. Following this rationale, Woringer and colleagues developed the ABCD Risk questionnaire in order to measure CVD knowledge and risk perception. In this study, we re-validated the tool on a sample of the general population in Nottingham to confirm the psychometric properties.

The 88 participants in this study who reported smoking is a low number for pilot testing of psychometric scales but it does exceed a 10:1 ratio of cases to variables making it reasonable to proceed to analysis.

Based on EFA and CFA, we confirmed a three-factor structure, which closely matched the results reported in the original study, but differed in certain important respects. Item 14 ('*When I eat at*

For peer review only - http://bmjopen.bmj.com/site/about/guidelines.xhtml

least 5 portions of fruit and vegetables a day I am doing something good for the health of my heart") showed a better loading to the 'healthy eating intentions' sub-scale, in contrast to the factor loading in the original study, which placed this item in 'perceived benefits and intentions to change'. This is the only item which loaded onto a different sub-scale when using the Nottingham dataset, all others continued to load onto their original factors although many of these loaded weakly and failed to meet usual thresholds for validity (Appendix 5). The larger numbers of participants in our dataset (466 compared to 110) provides statistical confidence in the new results, and we therefore modelled this revised allocation of items and factors alongside the original factor allocations in the subsequent Confirmatory Factor Analysis. The revised measurement model with item 14 allocated to 'Healthy Eating Intentions' indicated a better fit in CFA results.

These results suggest that the additional five smoking items perform acceptably and should be incorporated into future applications of the ABCD Risk Questionnaire.

Limitations

Our purposive sampling strategy was successful in engaging a sufficient range and number of participants to reflect the population characteristics of Nottingham (Appendix 6) and therefore permit the generalisation of results to similar urban centres. Nottingham shares a similar socio-economic profile with a number of English conurbations sometimes referred to as 'core cities'. [31] There is a significant but weak negative correlation between household income and measured CVD risk in our sample (-.161, sig .001, n=486) but more data will be required to establish whether the ABCD Risk Questionnaire can expose differential patterns in attitude and belief about CVD risk in wealthier sample populations.

Psychometric performance based on reliability calculations and factorial analysis is not an end in itself. The resulting scale has to have some utility in the world and generate results which can add value to existing understanding of beliefs and attitudes to cardiovascular disease risk. The literature refers to a 'know-do' gap in health education which is framed as a knowledge translation challenge from research to practice. [32] Analysing results from the ABCD Risk Questionnaire, our findings indicate that this gap also exists within patients/ study participants who have recorded high levels of knowledge and motivation to moderate unhealthy behaviours but low levels of success in doing so. This suggests that health education may be failing to stimulate healthy changes in this population, and that other factors (addiction/dependence/social acceptance/lack of resources/time sensitivity) may be limiting the impact of health education even as knowledge of risks and remedies is high. The ABCD Risk Questionnaire enables a careful exploration of the relationships between knowledge, motivation, attitudes and beliefs in relation to CVD risks and their remedies which may in future be combined with investigation of these confounding factors to improve the effectiveness of future health promotion strategies.

Other observations

Researchers in the Nottingham SPICES team administering the questionnaire during fieldwork reported that three items within the 'Perception of Risk of Heart Attack/Stroke' sub-scale caused consistent difficulties for respondents due to apparent duplication and confusion over fine semantic differences. It was difficult for participants to see a semantic difference between statements 9, 10, 11, and 12, 13 respectively. For items 9, 10, and 11, if we agree that *suffer from* and *have* are synonymous, it is hard to differentiate between *in the future* and *some time during my life* because you would imagine that respondents will be thinking about the future in both cases.

For peer review only - http://bmjopen.bmj.com/site/about/guidelines.xhtml

For the questionnaire to be reliable across all sections of the population, including those with limited ability in English (whether native or non-native, first, second or additional language, etc.) who may find it particularly hard to differentiate with any confidence between different pairs/sets of statements with largely synonymous meanings, this confusion is a problem. Items 12 and 13 seem to differ mainly only in the possible interpretation of a difference of degree between *good* and *great.*

These face validity issues and their impact can be observed in the inter-item correlation results generated during item reliability analysis. In the original study, two items in the perception of risk sub-scale had been rejected due to correlations in excess of 0.6 leaving 8 items. Of these remaining 8 items half had inter-item correlations which exceeded 0.6 when tested against the Nottingham dataset. These were items 9, 10, 11, and 12 which generated inter-item correlation values of .832, .869, .616, and .729 respectively. Removing items 9, 10, and 13 does not reduce the conceptual range of the 'perception of risk' subscale which is framed temporally from immediate threat to lifetime risk, it simply removes the duplicate or confusing items. Testing this shortened scale with factor analysis strengthens both item and scale reliability and improves factor loadings (Appendix 5). We recommend that future versions of the English language ABCD Risk Questionnaire adopt these edits (Table 4/Appendix 7).

CONCLUSIONS

The published English language version of the ABCD Risk Questionnaire, with the removal of three problematic 'perception' items, the shift of one item from the 'perceived benefits and intentions to change' sub-scale into the 'healthy eating intentions' sub-scale, and the addition of a 5 item 'smoking' sub-scale performs sufficiently well in validity, reliability and factor analysis with an independent, larger sample to confirm the generalisability of its original published findings. This result supports continued use of the ABCD Risk Questionnaire in the field of CVD prevention research and practice. The inclusion of a smoking behaviours sub-scale is likely to increase its relevance where smoking behaviours still account for a large proportion of individually modifiable CVD risk in a target population. Although criterion validity has now been established for the 'Perception of risk of heart attack/stroke sub-scale' by two published studies, [33] the utility of the remaining sub-scales individually or in combination has been under-examined. Future studies should investigate the criterion validity of these sub-scales and the conceptual strength of the items and variables from which they have been composed in order to unambiguously position the resulting survey instrument and evaluate its utility in CVD prevention and treatment practices. Neither this study or the original published study of 2017 were able to conduct pre-post intervention measurements in their study design. Measuring using the ABCD survey before an intervention (such as the NHS Health Check) and then again at some time afterwards- in tandem with a validated CVD risk prediction scale (such as INTERHEART or Q Risk 2) would help to establish the ABCD Risk Questionnaire's sensitivity to change, and perhaps also its ability to discern between types of respondent.

REFERENCES

1 https://www.bhf.org.uk/bhf-cvd-statistics-uk-factsheet.pdf (Accessed 10/05/2021)
2 https://www.england.nhs.uk/ourwork/clinical-policy/cvd/ (Accessed 10/05/2021)
3 Robson J, Dostal I, Sheikh A*, et al* The NHS Health Check in England: an evaluation of the first 4 years *BMJ Open* 2016;**6:**e008840. doi: 10.1136/bmjopen-2015-008840

For peer review only - http://bmjopen.bmj.com/site/about/guidelines.xhtml

4 Kearney M Prevention and treatment of CVD: a new priority for the NHS *Heart* 2019;**105:**1924.
5 Michie, S., van Stralen, M.M. & West, R. The behaviour change wheel: A new method for characterising and designing behaviour change interventions. *Implementation Sci* **6,** 42 (2011). https://doi.org/10.1186/1748-5908-6-42
6 Woringer M, Nielsen JJ, Zibarras L*, et al*
Development of a questionnaire to evaluate patients' awareness of cardiovascular disease risk in England's National Health Service Health Check preventive cardiovascular programme *BMJ Open* 2017;**7:**e014413. doi: 10.1136/bmjopen-2016-014413
7 Health Behavior: Theory, Research, and Practice edited by Karen Glanz, Barbara K. Rimer, K. Viswanath
8 Gorenoi, V. and Hagen, A., 2015. Overview of risk-estimation tools for primary prevention of cardiovascular diseases in European populations. *Central European journal of public health*, *23*(2), p.91.
9 Baker, M. 1,500 scientists lift the lid on reproducibility. *Nature* **533**, 452–454 (2016). https://doi.org/10.1038/533452a
10 Woringer M, Nielsen JJ, Zibarras L*, et al* IBID
https://www.nottinghaminsight.org.uk/themes/health-and-wellbeing/joint-strategic-needs-assessment/adults/cardiovascular-disease-2016/ (accessed 29/11/21)
11 Nahar, P., van Marwijk, H., Gibson, L. *et al.* A protocol paper: community engagement interventions for cardiovascular disease prevention in socially disadvantaged populations in the UK: an implementation research study. *glob health res policy* **5,** 12 (2020). https://doi.org/10.1186/s41256-020-0131-1
12 https://www.nottinghaminsight.org.uk/themes/health-and-wellbeing/joint-strategic-needs-assessment/adults/smoking-and-tobacco-control-2019/ (accessed 29/11/21)
13 Yusuf S , Hawken S , Ounpuu S , *et al* . Effect of potentially modifiable risk factors associated with myocardial infarction in 52 countries (the INTERHEART study): case-control study. Lancet 2004;**364**:937–52.doi:10.1016/S0140-6736(04)17018-9
14 https://www.nottinghaminsight.org.uk/themes/health-and-wellbeing/joint-strategic-needs-assessment/adults/smoking-and-tobacco-control-2019/ (Accessed 10/05/21)
15 John A Ambrose, Rajat S Barua, The pathophysiology of cigarette smoking and cardiovascular disease: An update, Journal of the American College of Cardiology, Volume 43, Issue 10, 2004, Pages 1731-1737, ISSN 0735-1097, https://doi.org/10.1016/j.jacc.2003.12.047.
16 Woringer M, Nielsen JJ, Zibarras L*, et al* IBID
17 Nahar, P., van Marwijk, H., Gibson, L. *et al.* IBID
18 Harris, P.A., et al., Research electronic data capture (REDCap)—A metadata-driven methodology and workflow process for providing translational research informatics support. Journal of Biomedical Informatics, 2009. **42**(2): p. 377-381 DOI: https://doi.org/10.1016/j.jbi.2008.08.010.
19 Woringer M, Nielsen JJ, Zibarras L*, et al* IBID
20 Woringer M, Nielsen JJ, Zibarras L*, et al* IBID
21 Woringer M, Nielsen JJ, Zibarras L*, et al* IBID
22 Cronbach, L.J. Coefficient alpha and the internal structure of tests. *Psychometrika* **16,** 297–334 (1951). https://doi.org/10.1007/BF02310555
23 Ferguson, E. and Cox, T. (1993), Exploratory Factor Analysis: A Users'Guide. International Journal of Selection and Assessment, 1: 84-94. https://doi.org/10.1111/j.1468-2389.1993.tb00092.x
24 Kaiser, H.F. The varimax criterion for analytic rotation in factor analysis. Psychometrika 23, 187–200 (1958). https://doi.org/10.1007/BF02289233
25 Bartlett, M. S. (1951). The effect of standardization on a Chi-square approximation in factor analysis. Biometrika, 38(3/4), 337-344
26 Hu Lt, Bentler PM. Cutoff criteria for fit indexes in covariance structure analysis: Conventional criteria versus new alternatives. Structural Equation Modeling: A Multidisciplinary Journal. 1999;6(1):1-55. doi: 10.1080/10705519909540118.
27 von Elm E, Altman DG, Egger M, Pocock SJ, Gotzsche PC, Vandenbroucke JP. The Strengthening the Reporting of Observational Studies in Epidemiology (STROBE) Statement: guidelines for reporting observational studies.
28 Ministry of Housing, Communities & Local Government. The English indices of deprivation 2019 statistical release https://www.gov.uk/government/statistics/english-indices-of-deprivation-2019 (accessed 10/05/2021)
29 Yusuf S , Hawken S , Ounpuu S , *et al* **IBID**
30 UK Government Office for National Statistics 2019
https://www.ons.gov.uk/peoplepopulationandcommunity/healthandsocialcare/healthandlifeexpectancies/bulletins/adultsomkinghabitsgreatbritain/2018 (Accessed 10/05/2021)

For peer review only - http://bmjopen.bmj.com/site/about/guidelines.xhtml

31 https://www.corecities.com/about-us/what-core-cities-uk (Accessed 22/08/22)
32 Ward PR, Thompson J, Barber R, et al. Critical perspectives on 'consumer involvement' in health research: Epistemological dissonance and the know-do gap. Journal of Sociology. 2010;46(1):63-82. doi:10.1177/1440783309351771
33 Woringer M, Nielsen JJ, Zibarras L, *et al* IBID & Martos T, Csabai M, Bagyura Z, *et al* Cardiovascular disease risk perception in a Hungarian community sample: psychometric evaluation of the ABCD Risk Perception Questionnaire *BMJ Open* 2020;**10:**e036028. doi: 10.1136/bmjopen-2019-036028

Figure legends

Figure 1. Scree plot of factor eigenvalues (original published 18 items) Nottingham dataset

Figure 2. Scree plot of factor eigenvalues (original published 18 items plus 5 smoking items) Nottingham dataset

Figure 3. Scree plot of factor eigenvalues (recommended amended ABCD) Nottingham dataset

For peer review only

For peer review only - http://bmjopen.bmj.com/site/about/guidelines.xhtml

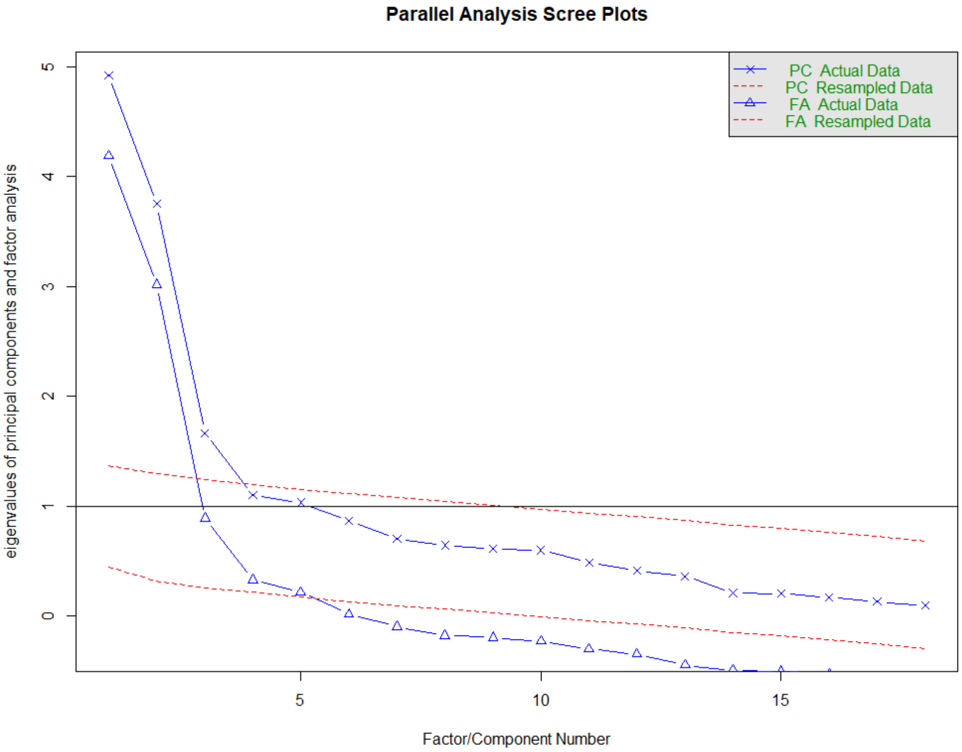

Figure 1. Scree plot of factor eigenvalues (original published 18 items)

266x211mm (96 x 96 DPI)

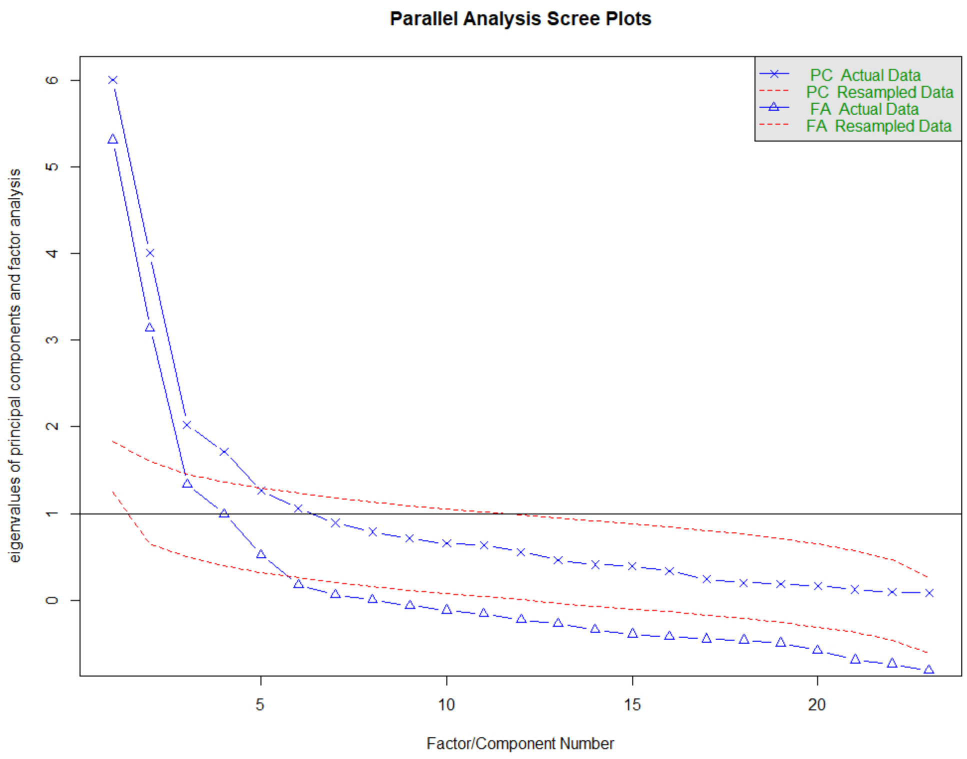

Figure 2. Scree plot of factor eigenvalues (original published 18 items plus 5 smoking items)

266x211mm (96 x 96 DPI)

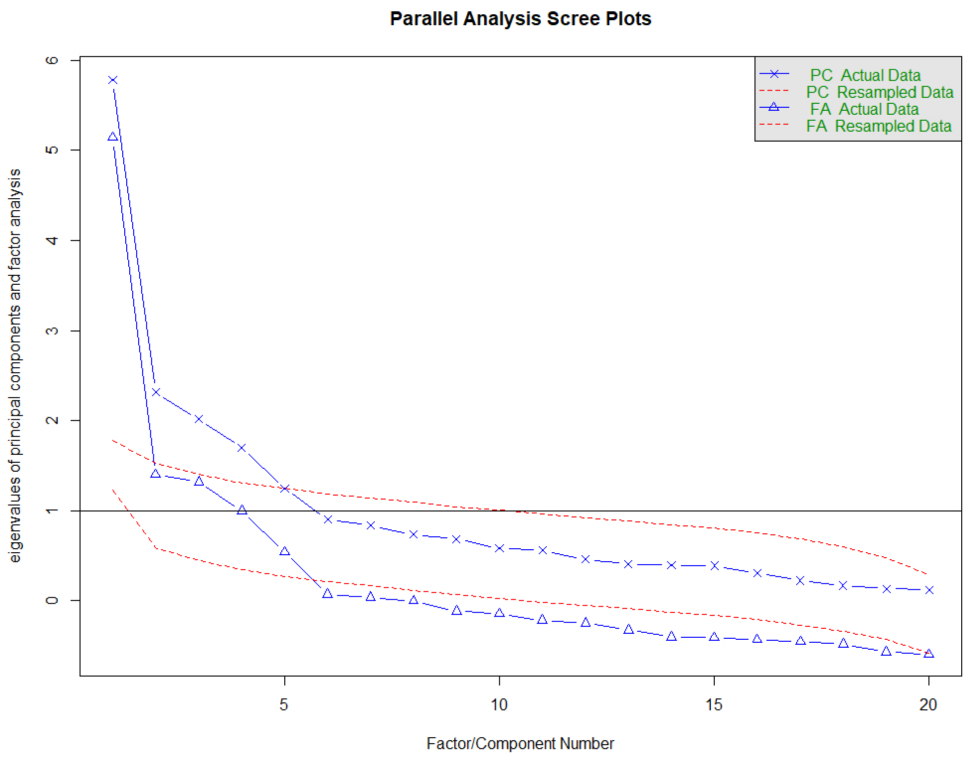

Figure 3. Scree plot of factor eigenvalues (recommended amended ABCD)

266x211mm (96 x 96 DPI)

Horizon 2020
European Union funding
for Research & Innovation

NOTTINGHAM
TRENT UNIVERSITY

‘SPICES’ Heart Diseases Prevention Research

Introduction to SPICES research

Nottingham Trent University is part of an international research team investigating ways to build good practice in the prevention of Heart Diseases. Researchers and doctors have a lot of evidence about what causes heart diseases and what prevents them. Heart Diseases are now the biggest cause of death globally, and one of the leading causes of disability, so the more people know what the doctors know, the better they can protect themselves and maintain a good quality of life.

The research project is called 'SPICES' and here in Nottingham we are going to see if working with people in the community instead of at the doctor's surgery, we can spread the message quicker and further.

If you choose to take part we will ask you to complete a simple survey. From the we will be able see how well you are looking after your heart in terms of your lifestyle. Then there will be three possible options:

If the data you provide suggests you may need to make some lifestyle changes we will recommend that you make an appointment to see your doctor. As researchers we cannot give any medical advice, but it would be inappropriate for us to ignore any signs of an unhealthy lifestyle that could give rise to heart problems.

If the data you provide suggests you have a healthy lifestyle, then this is positive news and we'll talk to you about how you might be able to help the project in other ways.

If you are somewhere in the middle we will show you some simple ways to reduce your risk and stay healthier for longer.

N.B. In all cases, the data you provided is for research purposes only and a decision about your health cannot be made on the basis of questionnaires only.   Whilst we advise you to see a doctor if figures are high, lower figures should not be taken to indicate a healthy heart, and the results should not be used to replace medical assessments and the taking of medical advice about other health monitoring strategies. The dividing of participants into three groups is for research purposes only and is not a medical intervention.

If you're interested please complete our survey (It might take about 10 minutes, and you will need a tape measure for one of the questions).

Our researchers will then get in touch with you about ways that we can support you to make your heart healthier.   Any information we collect will be kept securely and not shared outside of the research team. Your name and personal details will not be used in any reports, and all our records will be destroyed at the end of the project in line with the relevant GDPR legislation. Additionally you may withdraw your data at any time up to but no later than December 31st 2020 by contacting Mark Bowyer, SPICES Coordinator, Nottingham Trent University 0115 8485574 mark.bowyer@ntu.ac.uk

OK? Let's start with your agreement to take part.

For peer review only

Horizon 2020
European Union funding
for Research & Innovation

NOTTINGHAM TRENT UNIVERSITY

CONSENT FORM

'SPICES' Heart Diseases Prevention Research

You are making a decision to take part. By ticking ALL statements and signing your name below you will indicate that you have read the information provided above and decided to participate.

If you choose to discontinue participation in this study, you may withdraw at any time without judgement, or effect on your status.

CONSENT STATEMENT		Please tick if you agree
**1.**	I have received, read and understood the SPICES participant information sheet	
**2.**	I am aware that I can withdraw my participation at any time without prejudice, judgement or effect on my status in relation to Nottingham Trent University or its research partners	
**3.**	I understand that information I provide during my participation can be deleted at my request up to but no later than December 31st 2020	
**4.**	I agree to be contacted by SPICES researchers using the details that I have supplied below	
**5.**	I understand that the collection of data is not part of medical assessment or diagnosis and cannot be relied upon to reach conclusions as to the state of my health	
**5.**	I understand that any information I provide as part of the SPICES research will be managed in accordance with the EU General Data Protection Regulation (GDPR) framework (see SPICES participant information sheet)	
**6.**	I agree to take part in this research project	

Name:

Preferred contact details:

D.O.B.

Gender:

Postcode:

Signature:

Date:

……………………………………………………………………….

Staff signature:

Date:

For peer review only

A Protocol Paper: Community engagement interventions for Cardiovascular Disorders prevention in socially disadvantaged populations in the UK: An implementation research study
Final 15072019

Target Journal: Journal of Global Health Research and Policy
https://ghrp.biomedcentral.com/?gclid=Cj0KCQiA68bhBRCKARIsABYUGifuKd-xktjcmV7tn3r7G-IEqS5rAb6QmiEl6P9dXGBdNRDhsIPVzA0aAiJWEALw_wcB

Papreen Nahar[1], Harm van Marwijk[1], Linda Gibson[2], Geofrey Musinguzi[3], Sibyl Anthierens[4], Elizabeth Ford [1], Stephen A Bremner [1], Mark Bower [2], Jean Yves Le Reste[5], Tholene Sodi [6], Hilde Bastiaens[4]

Corresponding author: Dr Papreen Nahar, Department of Primary Care and Public Health, Brighton and Sussex Medical School, UK. The University of Sussex. E-mail: P.Nahar@bsms.ac.uk

Abstract:

Cardiovascular disorders (CVD) are the single greatest cause of mortality worldwide. In the UK, the National Health Service (NHS) has launched an initiative of health checks over and above current care to tackle CVD. However, the uptake of Health Checks is poor in disadvantaged communities. This protocol paper sets out a UK-based study aiming to co-produce a community delivered CVD risk assessment and coaching intervention to support community members to reduce their risk of CVD.

The overall aim of the project is to implement a tailored-to-context community engagement (CE) intervention on awareness of CVD risks in vulnerable populations in high, middle and low-income countries. This paper describes the protocol for the UK sites in Sussex and Nottingham. The specific objectives of the study are to enhance stakeholder' engagement; to implement lifestyle interventions for cardiovascular primary prevention, in disadvantaged populations and motivate uptake of NHS health checks.

This study takes a mixed methods approach, combining qualitative and quantitative methods in three phases of evaluation, including pre-, during- and post-implementation. To ensure contextual appropriateness the SPICES project will organize a multi-component community-engagement intervention implementation. For the qualitative component, the pre-implementation phase will involve a contextual assessment and stakeholder mapping, exploring potentials for CVD risk profiling strategies and led by trained Community Health Volunteers (CHV) to identify accessibility and acceptability. The during-implementation phase will involve healthy lifestyle counselling provided by CHVs and evaluation of the outcome to identify fidelity and scalability. The post-implementation phase will involve developing sustainable community-based strategies for CVD risk reduction. All three components will include a process evaluation. The theory of the socio-ecological framework will be applied to analyse the community engagement approach.
A stepped wedge quantitative evaluation of the roll out will focus on implementation outcomes such as uptake and engagement and changes in risk profiles. The quantitative component includes pre and post-intervention surveys.

The research project will ultimately develop a sustainable community engagement-based strategy for the primary prevention of CVD, to support or enhance the performance of NHS health care.

For peer review only - http://bmjopen.bmj.com/site/about/guidelines.xhtml

Key words: Implementation research, Cardiovascular disorders prevention, community engagement.

Introduction:

Cardiovascular disorders (CVD) are the single greatest cause of mortality worldwide each year, estimated to contribute to 31% of all deaths globally (1). Tackling CVD is an international priority and there have been many global initiatives such as the "Global Hearts" programme, a package launched by the World Health Organisation (WHO) and partners, to enhance the prevention and control of CVD. Some risk factors for CVD are non-modifiable, such as age, ethnicity and family history (2). Some other risk factors for CVD are modifiable, such as smoking, a lack of physical activity, being overweight, lower consumption of fruit and vegetables, high blood pressure, diabetes and high cholesterol (2). These risk factors can be changed through lifestyle or behavioural modifications. There is evidence of a social gradient in the prevalence of CVD, which points to associations between social and financial deprivation, vulnerability and risk factors for CVD. (3).

In 2015, CVD was the leading cause of mortality in the context of all chronic diseases, accounting for 27% and 25% of deaths in men and women respectively, in the UK(2). Coronary heart disease (CHD) and stroke were the main CVDs responsible for this mortality of men and women across all ages. As per British Heart Foundation report in 2017 CVD has a huge financial burden with annual associated healthcare costs estimated to be £9 billion annually in the UK (2). The UK has a standardised CVD death rate of 265.1 per 100,000 (2).

In the UK, the National Health Service (NHS) has launched the Health Check initiative aimed to prevent CVD. It is a national risk assessment and management program, free to adults aged 40 to 74 living in England, who do not currently have any vascular disorders and are not being treated for certain risk factors such as diabetes (4). It aims to assess the 10-year risk of CV events and disorders. Risk is assessed using QRISK2 (5), a tool which involves collection of the following information: age, gender, ethnicity, smoking status, family history of CHD, body mass index (BMI), cholesterol test, systolic and diastolic blood pressure, levels of physical activity, and alcohol consumption. Attendees receive a low (<10 % chance of event in 10 years), medium (>10 % but <20 %), or high (>20 %) 10-year cardiovascular (QRISK2) score. Above the 10% cut-off, attendees are offered a discussion with a qualified person, such as a nurse, about lifestyle and motivation to change, which may include goal setting and plans for follow up. Patients may also be offered medication for cholesterol and blood pressure. The NHS Health Check is recommended to be undertaken every five years.

Modelling predicted that the NHS Health Check could prevent 1,600 heart attacks and strokes each year if implemented as intended (6). Whilst evidence suggests that the Health Check programme has the potential to reduce CVD events and has therefore been rolled out nationally across the UK, its implementation has been poor, especially in some of the most disadvantaged groups at highest risk of developing CVD. In 2014, Public Health England (PHE) issued a call for action to increase the uptake rate of NHS Health Checks to 75% (7) and to increase awareness of risk and engagement with existing resources. Yet, as of 2017, current uptake remains far from this target with current predictions suggesting only 40% of the eligible population will receive one (8), due to the fact that uptake is low (48%) even when Health Checks are offered. (8) (9)

Data from some regions with very large ethnic minority community and socio-economically challenged populations showed that only 45% of patients who were invited for the check attended and subsequently received some form of counselling when they needed it. Authors have discussed how higher uptake in deprived communities would reduce the possibility of exacerbation of inequalities (10). Difficulty with accessing general practices, especially among socially vulnerable groups, has been highlighted as a common barrier to

For peer review only - http://bmjopen.bmj.com/site/about/guidelines.xhtml

attendance at Health Checks (11). A community-based engagement approach, which takes the CVD risking profiling and affiliated advice processes outside of the formal healthcare facility setting, has the potential to improve access to Health Checks and could be an effective and scalable way for improving the implementation and uptake of Health Checks. Community engagement (CE) has been conceptualised as “the process of working collaboratively with and through groups of people affiliated by geographic proximity, special interest, or similar situations, to address issues affecting the well-being of those people” (12). A review of community engagement interventions found them to be effective in improving health behaviours (such as physical activity), health consequences and psychological outcomes (i.e. self-efficacy and perceived social support) (13). Community-based intervention programmes have been implemented to increase the uptake of cancer screening programmes. The programmes have been found to be effective in increasing outcomes such as recognition, receipt and maintenance of screening behaviours (14). The CE approach offers the opportunity for task-shifting and owning the programme, whereby trained non-healthcare-professionals can perform CVD risk profiling assessments to individuals who might not otherwise be captured by the formal care pathway.

There is evidence that CVD risk assessments can be successfully delivered by Community Health Workers (CHWs), outside or inside the healthcare system. An observational study conducted in Bangladesh, Guatemala, Mexico and South Africa has demonstrated that CHWs who are inhabitants of their local communities and were fluent in the community’s predominant language, can perform community-based screenings to predict CVD risk as effectively as physicians and nurses when using the non-laboratory-based Gaziano CVD risk scoring tool (15). CHWs were trained for 1-2 weeks, and results showed a 96.8% agreement between risk scores assigned by CHWs and healthcare professionals. However, a question remains whether the model taken in the global South could be transferrable to the global North, but it is at least plausible that a community-based engagement approach will be effective for increasing the uptake of CVD risk assessment, particularly in disadvantaged communities of the global North. There are examples in the global North on community engagement in health (16), and indeed the voluntary or ‘third sector’ have been considered key partners in the delivery of health promotion initiatives in the community (17).

Authors have argued that because of the current economic constraints with the formal healthcare system, the focus should be upon supplementing a service delivery model with an alternative community development model (18). The key aspect is supplementing formal service delivery by utilizing communities’ ‘social capital’. The term ‘social capital’ describes the various resources that people may have through their relationships in families, communities and other social networks. Social capital bonds people together and helps them make links beyond their immediate friends and neighbours (19).

For this compassionate community approach to work, contextual appropriateness and cultural sensitivity of an intervention is crucial (20). Following this argument, the SPICES project in two areas of England, East Sussex and Nottingham, will co-produce a multi-component community-engagement intervention focussed on delivering a Health Check-style CVD risk screening, with appropriate health coaching and follow-up, in a community setting (21) and delivered by community volunteers. The intervention will be trialled and evaluated using a mixed methods approach using both qualitative and quantitative methods. The specific objectives of the project are:

To evaluate with stakeholders the potential for a community engagement-based CVD primary prevention programme to support or enhance the NHS Health Check Programme.

To co-produce with the communities an evidence-informed community-engagement intervention on CVD risk, based on the NHS Health Check model, tailored to the context in disadvantaged communities in East Sussex and Nottingham.

For peer review only - http://bmjopen.bmj.com/site/about/guidelines.xhtml

To implement the intervention in the local communities where it was co-produced, and:
-assess its effectiveness versus routine care.
-assess the fidelity, feasibility, acceptability, uptake and scalability of the implementation.
-carry out a process evaluation of the intervention and its implementation

This project is part of the SPICES (Scaling-up Packages of Interventions for Cardiovascular disease prevention in selected sites in Europe and Sub-Saharan Africa) project (22). This is a Horizon 2020 project financed by the European Commission that aims to address the CVD burden. The overall objective is to implement and evaluate a comprehensive cardiovascular disease (CVD) prevention and care program at the community level in five countries (Belgium, France, Uganda, UK, South Africa), to identify and compare barriers and facilitators for implementation across study contexts and to develop a learning community.

Methods:

Theoretical Model

SPICES is underpinned by the Consolidated Framework for Advancing Implementation Research (23), and Reach, Effectiveness, Adoption, Implementation, and Maintenance (sustainability) framework /RE-AIM models (24). We also recognize as a global health project the need for the use of the socio-ecological framework (25). As mentioned above, this model allows an understanding of the multifaceted and interactive effects of personal, social and environmental factors that determine behaviour; and for identifying behavioural and organisational leverage points and intermediaries for health promotion within organisations and communities.

Study Design

A mixed-methods research methodology will be applied strategically combining qualitative and quantitative methods at both sites. This approach will allow us to model the iterative nature of coproduction and implementation research without compromising the rigour of the study (26; 27). The study will take place in three phases:
- Pre-intervention; when stakeholder mapping and local adaptation will be carried out
- Intervention roll out, recruitment and evaluation
- Post-intervention evaluations and feedback (28)- Process evaluation will be conducted in all three phases.

Stage 1: To explore the implementation context and co-produce the intervention.

To explore the context where the implementation will take place we will carry out several mappings. These will give us the context for recruitment and implementation co-design.
They are as follows:
(a) Mapping the potential stakeholders: Mapping of the stakeholders will be done to find out who are the key stakeholders, where they come from, and what they are looking for in relationship to the study objectives(29). To engage the community, it is essential to map the community stakeholders (civil society organisations) as they are the gatekeepers of the community. Three levels of stakeholder mapping will be carried out, namely at macro, meso and micro levels.
Macro-level: stakeholders will be identified via the existing link of PI of the project in the community through meetings with local public health or other relevant departments and CSOs and using online information. Interviews with this category of stakeholders will provide insights into implementation sustainability.
Meso-level: a strategic community volunteer organisation mapping will be carried out to find out the relevant organisations, through which individual volunteers will be selected. This will

For peer review only - http://bmjopen.bmj.com/site/about/guidelines.xhtml

be done in three ways; using online searches, personal contacts and snowballing. In-depth interviews will be conducted to co-design a sustainable intervention implementation.

Micro-level: an exploration will be done with volunteers and end-user groups to co-design an acceptable and feasible intervention implementation.

(b) Mapping the context: social mapping will be carried out to explore the lifestyle context of the community via observations.

(c) Training of volunteers by professional health trainers and researchers following current NICE Public health guideline [PH6] 'Behaviour change: general approaches' (30)

(d) CVD risk profiling by trained community health volunteers (CHV).

CHVs will be the persons who have been involved in health-related volunteering for example volunteers who worked in cancer prevention, health check, healthy lifestyle etc programme. They will be involved in the screening of the CVD risk population and implement the designed intervention.

Expected Intervention

The final elements of the intervention will be co-produced within each community setting, following the mapping exercises outlined above. As outlined in the CFAIR (23), interventions are usually composed of a core component which is essential and indispensable, and an adaptable periphery, which can and should be tailored to the specific setting and users.

Core Components: Following identification of moderate to high risk for CVD, the intervention will consist of non-clinical (non-NHS) individual or group support sessions within the community, focus on motivating behaviour change. Each participant will be supported by trained SPICES researchers or community health workers to identify behaviour change goals, produce action plans to achieve them, and problem solve in cases of unexpected outcomes. All SPICES Interventions are theoretically grounded in the theory of behaviour change and deploy the strongest evidenced Behaviour Change Techniques (BCTs) from the literature.

1. Goal Setting
2. Action Planning
3. Problem Solving
4. Motivational Interviewing
5. Feedback on progress towards goals
6. Feedback on the health impact

The use of these six BCTs are focussed in SPICES on five Target Behaviours:

1. Reduce/cease smoking
2. Increase moderate physical activity
3. Reduce fat, salt, the sugar content of the diet
4. Increase fibre, oily fish, fruit and vegetable content of the diet
5. Reduce sedentary hours

Community Adaptation: The exact elements of the support sessions will be tailored to individuals and their community context, will be determined during iterative co-design with community representatives, and will be drawn from the following (31; 32):

Step-I - Goal setting

Every participant should receive specific healthy lifestyle counselling/feedback based on their individual item InterHE ART assessment scores (the moderate group). The feedback will be based on a review of international guidelines conducted as formative work for the SPICES project intervention (33). SPICES behaviour change support sessions will be based on the best-evidenced approaches to healthy lifestyle modification and community context and preferences.

For peer review only - http://bmjopen.bmj.com/site/about/guidelines.xhtml

Two further screening questionnaires may be used with individuals to assess the benefit of possibly behaviour change;

- International Physical Activity Questionnaire (IPAQ, see appendix) is an internationally validated instrument to capture information about weekly physical activity habits, behaviours and routines.
- The Dietary Approaches to Stop Hypertension Questionnaire DASH-Q is a self-reporting lifestyle questionnaire (see appendix) to capture information about weekly dietary habits, routines and behaviours, based around 'Dietary Approach to Stopping Hypertension' (34).
- Current behaviours audit: Using food and physical activity diaries prepared by and provided to participants by the SPICES research team, participants will be encouraged to complete an audit of one week of current dietary and physical activity behaviours, habits and routines to establish a baseline from which goals for change and improvement can be set in negotiation with SPICES CHVs
- The ABCD self-reporting questionnaire (see appendix) to assess participant perception of personal heart health risk.
- The EQ-5D-5L internationally validated Quality of Life self-reporting questionnaire (see appendix).

Step-II - Action Planning by the participants

Participants will be asked to create an action plan with appropriate goal setting for two behaviours (diet and exercise habits) in relation to when, where and how they will undertake, for example, physical activity (based on the item stems used by Luszczynska & Schwarzer (35); when the physical activity will be performed, where it will be performed, how often it will be performed. The way goals are reached and plans recorded will be co-designed with key stakeholders.

Step III - Problem-solving

CHVs will help participants to analyse any factors which may influence their ability to achieve the goals and to generate strategies which could help them overcome these barriers.

CHVs will use Motivational Interviewing techniques about health, social and environmental, and emotional barriers and consequences. Culturally and context-sensitive information will be provided (both verbally and in the form of leaflets) about the importance of eating healthily, being physically active, and not smoking for positive outcomes on physical and mental health.

Trial of Intervention

This will be an open-label, non-controlled trial, examining fidelity, feasibility, acceptability, uptake and scalability of the intervention.

Eligible Population

Economically disadvantaged, lower socio-economic status (SES) postcodes, will be identified using the overall Index of Multiple Deprivation (36a); Participants' SES will be determined by their postcode of residence. Any resident aged 18 or above living in the study postcode areas will be eligible to take part in the baseline assessment for the study.

Study Sample Size

The sample size calculation for the quantitative study used statistical modelling for a stepped wedge design, randomising community centres over time with the InterRHEART score as the outcome (90% power for 5% significance, effect size (Cohen's D)=0.25, intracluster correlation coefficient of 0.05, control clusters crossing to intervention in 4 steps, participant autocorrelation=0.7 and cluster autocorrelation=0.9), which requires a total of at least 144 persons. This needs approximately 200-300 people across the two sites as we expect a high level of attrition (as much as 50%). At least 1500 community members will need to be screened to achieve this recruitment (37).

For peer review only - http://bmjopen.bmj.com/site/about/guidelines.xhtml

Recruitment of Community Health Volunteers and Trial Participants

Community Health Volunteers (CHVs) will be recruited to perform CVD risk profiling assessments through a combination of 'doorstep outreach' and 'intermediary organisation recruitment' approaches in East Sussex and through existing community and neighbourhood groups with the assistance of partners such as Self-Help UK, the Renewal Trust, Nottingham CVS and others in Nottingham.

For recruitment of trial participants, we will use similar community networks, and endeavour to use quota sampling, in that we will seek to ensure the inclusion of high, low and median income neighbourhood residents, citizens from the South Asian and African diasporas; and will encourage participants to refer others to the researchers who may be able to potentially contribute or participate in the study.

Baseline Screening of CVD Risk

Participants will fill in the validated InterHEART score to determine suitability for the trial. The non-laboratory-based InterHEART scoring tool requires minimal resources which is practical for use within the community. There is also evidence to suggest that the InterHEART can reliably predict the incidence of CVD and death in low, middle, and high-income countries for a mean follow-up of 4.1 years (38). Risk is expressed as a score from the InterHEART: 0-9 (Low risk), 10-15 (moderate risk), and 16-48 (high risk). The InterHEART scoring tool will be translated onto a mHealth platform so that the trained CHVs can easily administer them during community engagement and contact, and online data will directly reach the University repository in real time from the respondents' device.

Participants who score moderate or high risk in the baseline assessment will be invited to participate in the intervention. The moderate risk (amber) score population will be selected for participation in the intervention (=score of 10 or higher), and will fill out the self-completion survey InterHEART scoring every three months. The InterHEART scoring tool will be translated onto a mHealth platform so that the trained CHVs can easily administer them during community engagement and contact, and online data will directly reach the University repository in real time from the respondents' device (39).

Clinical Outcome and Follow-Up

The primary outcome will be the change in the risk score among people who complete the community delivered CVD risk assessment and coaching. Secondary outcomes will be gathered from participants identified as 'high risk'. Numbers of participants who a) self-referred (defined as having contacted their GP surgery requesting for a formal check-up) and b) completed the NHS Health Checks

Data collected during the trial of intervention will comprise:

- Self-reported lifestyle (modifiable and non-modifiable) risk factors gathered through survey instruments and interviews.
- Observed/measured data on all participants' age, gender, ethnicity, postcode, hip to waist ratio, gathered by trained volunteers.
- Quantitative analysis of changes in behavioural intention, target behaviours, and measurable CVD risk.

Outcomes will be assessed at three months post-intervention.

Post-intervention Qualitative Evaluation and Feedback

In the post-intervention phase, a qualitative evaluation will be carried out during which

The following implementation parameters will be assessed:

For peer review only - http://bmjopen.bmj.com/site/about/guidelines.xhtml

1. The impact on awareness of CVD risks and mitigating measures, amongst disadvantaged populations of a community-based, non-clinical, CVD risk scoring tool and education.
2. The impact of the community based non-clinical CVD risk scoring tool and education on motivational healthy lifestyle among disadvantaged populations.
3. The facilitators and barriers to the adoption of a community-based CVD prevention implementation programme, by target populations.
4. The perspectives of participants regarding their experience and meaning of the intervention.

These will be explored with a subset of intervention participants using focus groups or/and in-depth interview and community mapping. Participants for the qualitative component will include adult volunteers, public health stakeholders and people within the community. The community volunteers will be selected via community organisations and public health stakeholders will be selected from the same area of the research site. Community participants for the qualitative component will be selected via the community volunteers. This post-intervention qualitative study will include randomly selected trial participants.

We will be flexible in terms of the number of participants for the qualitative component. The number will be determined through the principle of saturation and diversity. However, from each site, we will aim to include at least 12 respondents and a maximum of 30 respondents from different categories (40; 41).

Process evaluation of the intervention

To assess the fidelity of the conclusions concerning the project's effectiveness, ongoing assessment, monitoring, and enhancement is important. If significant results are found, but fidelity was not assessed, it cannot be determined if the effectiveness is attributable to unintentionally added or omitted components. Bellg and colleagues (42) propose that considerations of fidelity should permeate all stages of the study: design of the study, provision of training, delivery of the intervention, receipt of the intervention, and re-enactment of skills. As a result, we will carry out a process evaluation of the project. This will be done through Process Documentation of all the stages of this project including community volunteers mapping, Healthy lifestyle counselling, action planning and problem-solving.

Thirsk and Clark (43) argue how health-care interventions need to be understood in ways that are responsive to the complexities and intricacies of programs, people and places. They emphasise the understanding of the comprehensive experience of the persons who are delivering and receiving the intervention. Process Evaluation is a tool that can capture the intervention experience. We will be following the model designed by Moore et al (44):

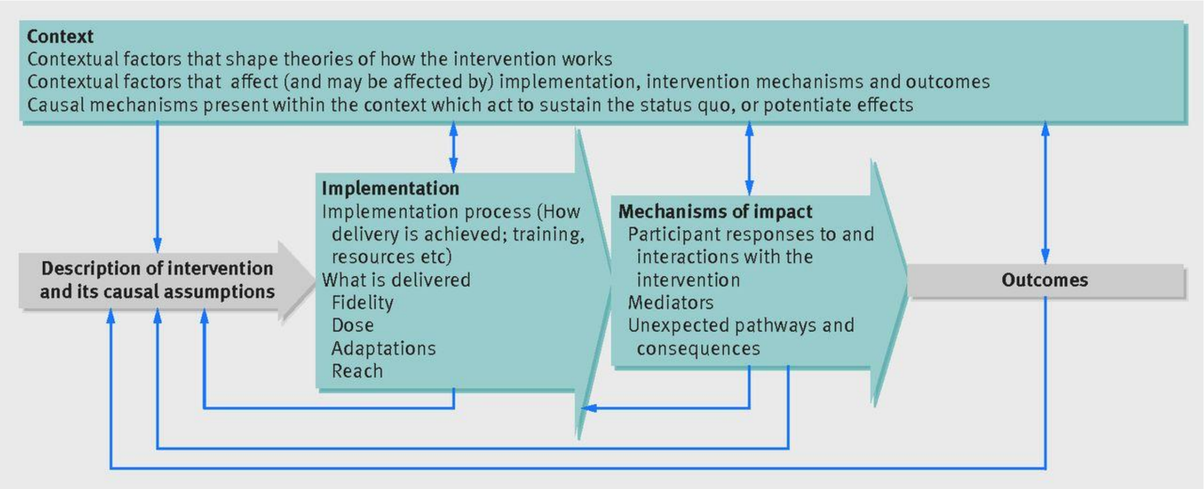

For peer review only - http://bmjopen.bmj.com/site/about/guidelines.xhtml

Data Analysis:
Quantitative data will be analysed using Stata version 15 or later. Descriptive statistics will summarise outcomes before and after clusters cross over to the intervention (45. Normally distributed variables will be summarised by means and standard deviations, skewed continuous variables by medians and interquartile ranges, categorical variables by frequencies and percentages. We will estimate the treatment effect using a cross-classified linear mixed effects model. A statistical analysis plan will be agreed and signed off prior to final analysis commencing. Thematic analysis of qualitative data will be carried out using a constant comparison method of analysis, which will gather and generate ideas and categories through inductive processes. The computer package NVivo will be used for primary analysis (46). Memo writing will be carried out to describe details of the interview setting and interaction of respondent and interviewer that may not be captured in audio transcriptions. This thematic analysis has deductive and inductive elements, lending itself to multidisciplinary health research (47). The analysis framework will incorporate the key theoretical constructs and respond to the context of policy and practice to include a range of deductive themes. Further themes will be induced from the interview data.

An appropriate balance of integration between empirical data and interpretation will be ensured. The investigators will extract the meaning of the empirical data and interpret them whilst acknowledging the complexity of the phenomena of CVD risk reduction in the context of community engagement (48). This method holds links to the original data and the output allows comprehensive and transparent data analysis.

Conclusion:
Given that despite the rolling out of the NHS Health Checks programme over and above current care across the UK has not been implemented as well as it could have been, especially in some of the most disadvantaged groups prone to developing CVD, the project aims to scale-up packages of interventions for cardiovascular prevention particularly to these vulnerable populations. This interdisciplinary project includes public health, social and behavioural science approaches. The main focus aspect of this project is the deinstitutionalization of health care by operating outside of formal healthcare settings. The project will emphasise on the power of citizens, combining their efforts to generate cultures of care which complement or even compensate for the inadequacies of formal systems thus sustainable. The research project will ultimately develop a community engagement-based CVD primary prevention programme to support or enhance the performance of the NHS health care.

Funding statement:
This protocol is a contextual plan for the SPICES project in the UK. The SPICES project received funding from the European Commission through the Horizon 2020 Research and Innovation Action Grant Agreement No 733356 to implement and evaluate a comprehensive CVD prevention programme in five settings: a rural & semi-urban community in a low-income country (Uganda), middle income (South Africa) and vulnerable groups in three high-income countries (Belgium, France and United Kingdom). The funder had no role in the design, decision to publish, or preparation of the manuscript.

Availability of data and materials:
A protocol should not contain any data; it sets out the research questions and how they will be addressed.

For peer review only - http://bmjopen.bmj.com/site/about/guidelines.xhtml

Ethics approval and consent to participate:
This protocol has received two ethics approval from the University of Sussex, The **BSMS** Research Governance and Ethics Committee (RGEC (ER/BSMS9E3G/1)), and from Nottingham Trent University (no. TBA). All participants will be requested to consent before enrolment into the study. All participant information will be kept confidential and accessible only to the key investigative team. All published data will be anonymised and can be accessed based on a written request to the Principal Investigator.

Competing interests:
Authors declare that they have no competing interests.

Authors' contributions:
PN has written the first draft and received feedback from HvM and SA on it. PN prepared the second draft and it received feedback from LG. The third draft received feedback from all the authors. All authors read and approved the final contextual protocol (4th version).

References:

1. World Health Organisation; WHO. *Global Health Observatory (GHO) data*. 2017. Retrieved on 18th May 2019, retrieved from https://www.who.int/gho/mortality_burden_disease/causes_death/top_10/en

2. British Heart Foundation report BHF. https://www.bhf.org.uk/informationsupport/publications/policy-documents/annual-report-2017.

3. Amuzu A, Carson C, Watt HC, Lawlor DA, Ebrahim S. Influence of area and individual life course deprivation on health behaviours: findings from the British Women's Heart and Health Study. Eur J Cardiovasc Prev Rehabil. 2009;16(2): 169-73. doi: 10.1097/HJR.0b013e328325d64d.

4.Public Health England (2013). NHS Health Check implementation review and action plan Summary, https://assets.publishing.service.gov.uk/government/uploads/system/uploads/attachment_data/file/224536/NHS_Health_Check_implementation_review_and_action_plan_summary_web.pdf 5.

5. QRISK2. 2017. https://qrisk.org/2017/

6. Public Health, England. NHS Health Checks 'prevented thousands of heart attacks'. 2016. https://www.nhs.uk/news/heart-and-lungs/nhs-health-checks-prevented-thousands-of-heart-attacks/

7. Public Health England. Factsheet: Implementation of the NHS Health Check programme. 2014. https://www.england.nhs.uk/wp-content/uploads/2014/02/pm-fs-3-1.pdf

8. Waterall Jamie. PHE. Getting Serious About CVD Prevention. Reducing Variation & Optimising Care. 2018. https://www.healthcheck.nhs.uk/seecmsfile/?id=195

For peer review only - http://bmjopen.bmj.com/site/about/guidelines.xhtml

9. Dalton AR, Bottle A, Okoro C, Majeed A, Millett C. Uptake of the NHS Health Checks programme in a deprived, culturally diverse setting: cross-sectional study. J Public Health (Oxf). 2011; 33(3):422-9. doi: 10.1093/pubmed/fdr034.

10. Robson J, Dostal I, Sheikh A, et al. The NHS Health Check in England: an evaluation of the first 4 years. 2015. BMJ Open. 2015;6: e008840. doi:10.1136/ bmjopen-2015

11. Harte Emma, Calum MacLure, Adam Martin, Catherine L Saunders, Catherine Meads, Fiona M Walter, Simon J Griffin, Jonathan Mant and Juliet A Usher-Smith (2018), Reasons why people do not attend NHS Health Checks: a systematic review and qualitative synthesis, British Journal of General Practice. 2018;68 (666): e28-e35. DOI: https://doi.org/10.3399/bjgp17X693929

12. CTSA. Principles of Community Engagement, Second Edition, NIH Publication No. 11-7782. 2011. https://.atsdr.cdc.gov/communityengagement/pdf/PCE_Report_508_FINAL.pdf

13. O'Mara-Eves A., G Brunton, D McDaid, S Oliver, J Kavanagh, F Jamal, T Matosevic, A Harden, and J Thomas. Community engagement to reduce inequalities in health: a systematic review, meta-analysis and economic analysis, Public Health Research. 2013;1:4

14. Bird JA; McPhee SJ, Ha NT, Le B, Davis T, Jenkins CN. Opening pathways to cancer screening for Vietnamese-American women: lay health workers hold a key. Prev Med. 1998;27(6):821-9.

15. Gaziano Thomas A, Shafi ka Abrahams-Gessel, Catalina A Denman, Carlos Mendoza Montano, Masuma Khanam, Thandi Puoane, Naomi S Levitt (2015). An assessment of community health workers' ability to screen for cardiovascular disease risk with a simple, non invasive risk assessment instrument in Bangladesh, Guatemala, Mexico, and South Africa: an observational study. Lancet Glob Health. 2015; 3:e556–63. http://dx.doi.org/10.1016/ S2214-109X(15)00142-4

16. Wood, J. The University as a Public Good: Active citizenship and university community engagement. International Journal of Progressive Education. 2012; 8 (3), 15-31. Retrieved from http://dergipark.org.tr/ijpe/issue/26312/277299

17. Kings Fund. 2011. Retrieved from, https://www.kingsfund.org.uk/sites/default/files/Voluntary-and-community-sector-in-health-implications-NHS-reforms-The-Kings-Fund-june-2011_0.pdf

18. Abel, J., Bowra, J., Walter, T. et al. Compassionate Community networks: Supporting home dying. BMJ Supportive and Palliative Care. 2011;1,129-133. http://dx.doi.org/10.1136/bmjspcare-2011-000068

19. Baum FE and Ziersch AM. Social capital. Journal of Epidemiology & Community Health. 2003; 57: 320-323.

20. Sallnow L. Libby, Heather Richardson, Scott Murray, Allan Kellehear. Understanding the impact of a new public health approach to end of life care: a qualitative study of a community-

For peer review only - http://bmjopen.bmj.com/site/about/guidelines.xhtml

led intervention. The Lancet. 2017; 389: Special issue 788. DOI:https://doi.org/10.1016/S0140-6736(17)30484-1

21. Boyd, Stephen McKernon, Bernie Mullin, Andrew Old. Improving healthcare through the use of co-design. The New Zeeland Medical Journal. 2012; 125:(1357).

22. Musinguzi Geofrey, Rhoda K. Wanyenze1, Rawlance Ndejjo, Isaac Ssinabulya, Harm van Marwijk, Isaac Ddumba, Hilde Bastiaens and Fred Nuwaha. An implementation science study to enhance cardiovascular disease prevention in Mukono and Buikwe districts in Uganda: a stepped-wedge design. Health Service research. 2019; 19:253 doi.org/10.1186/s12913-019-4095-0

23. Damschroder Laura J, David C Aron, Rosalind E Keith, Susan R Kirsh, Jeffery A Alexander and Julie C Lowery. Fostering implementation of health services research findings into practice: a Consolidated Framework for Advancing Implementation Science. Implementation Science. 2009; 4:50 doi:10.1186/1748-5908-4-50

24. Glasgow RE, McKay HG, Piette JD, Reynolds KD: The RE-AIM framework for evaluating interventions: what can it tell us about approaches to chronic illness management? Patient Educ Couns. 2001;44:119-127.

25. Theobald, S., Brandes, N., Gyapong, M., et al. Implementation research: new imperatives and opportunities in global health, Lancet. 2018;392: 2214–28

26. Peters DH, Adam T, Alonge O, et al. Implementation research: what it is and how to do it. BMJ. 2013; 347: f6753.

27. Brown CH, Curran G, Palinkas LA, et al. (2017). An overview of research and evaluation designs for dissemination and implementation. Annual Review of Public Health. 2017; 38:1–22

28. Greene, J. C. Mixed Methods in Social Inquiry. 2007. Jossey-Bass. San Francisco.

29. BSR. Stakeholder Mapping (2011). www.bsr.org/reports/BSR_Stakeholder_Engagement_Stakeholder_Mapping.final.pdf

30. NICE Public health guideline [PH6] 'Behaviour change: general approaches' (https://www.nice.org.uk/guidance/ph6/resources/behaviour-change-general-approaches-pdf-55457515717).

31. Michie Susan, Michelle Richardson, Marie Johnston, et al. (2013). The Behaviour Change Technique Taxonomy (v1) of 93 Hierarchically Clustered Techniques: Building an International Consensus for the Reporting of Behavior Change Interventions. Annals of Behavioural Medicine. 2013. 46;(1):81–95, https://doi.org/10.1007/s12160-013 9486-6

32. Michie, Susan, Maartje M. Van Stralen, and Robert West. "The Behaviour Change Wheel: A New Method for Characterising and Designing Behaviour Change Interventions. Implementation Science. 2011;6,1:42.

For peer review only - http://bmjopen.bmj.com/site/about/guidelines.xhtml

33. Yusuf Salim, Steven Hawken, Stephanie Ôunpuu, et al., on behalf of the INTERHEART Study Investigators. Effect of potentially modifiable risk factors associated with myocardial infarction in 52 countries (the INTERHEART study): a case-control study. Lancet. 2004; 364: 937–52

34. Sacks Frank M., Laura P. Svetkey, M.D., William M. Vollmer et al. Effects on Blood Pressure of Reduced Dietary Sodium and the Dietary Approaches to Stop Hypertension (DASH) Diet.
N Engl J Med. 2001; 344:3-10, DOI:10.1056/NEJM200101043440101

35. Luszczynska Aleksandra, Urte Scholz & Ralf Schwarzer. The General Self-Efficacy Scale: Multicultural Validation Studies, The Journal of Psychology. 2005; 139:5, 439-457, doi:10.3200/JRLP.139.5.439-457

36.. Kontopantelis E, Mamas MA, Van Marwijk H, Ryan AM, Buchan IE, Ashcroft DM, et al. Geographical epidemiology of health and overall deprivation in England, its changes and persistence from 2004 to 2015: A longitudinal spatial population study. 2018; 72 (2):140–7.

37. Hooper R, Teerenstra S, de Hoop E & Eldridge S. Sample size calculation for the stepped wedge and other longitudinal cluster randomised trials. Statist. Med. 2016; 35 4718–4728

38. Yusuf Salim, Sumathy Rangarajan, Koon Teo, Shofiqul Islam, et al. Cardiovascular. Risk and Events in 17 Low-, Middle-, and High-Income Countries. N Engl J Med. 2014; 371:818-827, DOI: 10.1056/NEJMoa1311890

39. Williams Natalie Joseph, Amy Lloyd, Adrian Edwards, Lynne Stobbart, David Tomson, Sheila Macphail, Carole Dodd, Kate Brain, Glyn Elwyn, Richard Thomson. Implementing shared decision making in the NHS: lessons from the MAGIC programme. BMJ. 2017a;357:j1744 doi: https://doi.org/10.1136/bmj.j1744

40. Guest, G., Bunce, A., & Johnson, L. How Many Interviews Are Enough?: An Experiment with Data Saturation and Variability. Field Methods. 2006; 18 (1): 59–82. https://doi.org/10.1177/1525822X05279903

41. Malterud K, Siersma VD, Guassora AD. Sample Size in Qualitative Interview Studies: Guided by Information Power. Qual Health Res. 2015; 1–8.

42. Bellg, A. J., Borrelli, B., Resnick, B., Hecht, J., Minicucci, D. S., Ory, M., et al. Treatment Fidelity Workgroup of the NIH Behavior Change Consortium. (2004). Enhancing Treatment Fidelity in Health Behavior Change Studies: Best Practices and Recommendations From the NIH Behavior Change Consortium. Health Psychology. 2004; 23(5):443-451. http://dx.doi.org/10.1037/0278 6133.23.5.443

43. Thirsk Lorraine M. and Alexander M. Clark. Using Qualitative Research for Complex Interventions: The Contributions of Hermeneutics. International journal of qualitative methods. 2017; 16:1-10. doi.org/10.1177/1609406917721068

For peer review only - http://bmjopen.bmj.com/site/about/guidelines.xhtml

44. Moore Laurence, Alicia O'Cathain, Tannaze Tinati, Daniel Wight, Janis Baird (2015). Process evaluation of complex interventions: Medical Research Council guidance. The BMJ. 2015;350:h1258. doi: 10.1136/bmj.h1258

45. StataCorp. 2017. *Stata Statistical Software: Release 15*. College Station, TX: StataCorp LLC

46. Ritchie Jane, Jane Lewis, Carol McNaughton Nicholls, Rachel Ormston. Qualitative Research Practice: A Guide for Social Science Students and Researcher; 2013. Sage Publications.

47. Gale NK, Heath G, Cameron E, Rashid S, Redwood S. Using the framework method for the analysis of qualitative data in multi-disciplinary health research. BMC Med Res Methodol. 2013;(13):117. doi.org/10.1186/1471-2288-13-117

48. Green J, Thorogood N. Qualitative methods for health research. 2005. London: Sage Publications Ltd.

Authors Information:

1. Papreen Nahar. Department of Primary Care and Public Health, Brighton and Sussex Medical School. University of Sussex, UK.
1. Harm van Marwijk. Department of Primary Care and Public Health, Brighton and Sussex Medical School. The University of Sussex. UK
2. Linda Gibson: School of Social Sciences. Nottingham Trent University, UK
3. Musinguzi Geofrey. Department of Disease Control and Environmental Health, School of Public Health, College of Health Sciences. Makerere University, Uganda
4. Sibyl Anthierens. Department of Primary and Interdisciplinary Care, University of Antwerp, Belgium
1. Elizabeth Ford. Department of Primary Care and Public Health Brighton and Sussex Medical School. University of Sussex, UK
1. Stephen A Bremner. Department of Primary Care and Public Health Brighton and Sussex Medical School. University of Sussex, UK
2. Mark Bower. School of Social Sciences, Nottingham Trent University, UK
5. JY Reste. Faculté de médecine et des sciences de la santé, Université de Bretagne Occidentale, Brest, France
6. Sodi Tholene. Department of Psychology. University of Limpopo, South Africa
4. Hilde Bastiaens. Department of Primary and Interdisciplinary care. University of Antwerp, Belgium

For peer review only - http://bmjopen.bmj.com/site/about/guidelines.xhtml

Appendix 3

ABCD subscale and selected INTERHEART variable correlation values from Nottingham study compared with values reported in the original Woringer study.

		Knowledge	Perceived Risk	Perceived Benefit	Healthy Intentions	IMD2010 Quintile	BMI/W2Hr	Qrisk2/ INTERHEART
Knowledge	Correlation Coefficient		-.124/ **.013**	-.148/ **-.021**	-.106/ **-.039**	-.002/ .085	-.225/ -.084	-.007/ -.018
	Sig 2 tailed		.236/ .722	.175/ .645	.319/ .400	.986/ .066	.021/ .082	.941/ .714
	N		93/462	86/462	91/462	99/466	105/433	104/436
Perceived Risk	Correlation Coefficient			-.195/ -.112	-.188/ -0.36	.239/ .039	.389/ .182	.220/ .356
	Sig 2 tailed			.080/ .016	.088/ .441	.025/ .397	.000/ .000	.036/ .000
	N			82/462	84/462	87/466	92/433	91/436
Perceived Benefits	Correlation Coefficient				.533/ .383	-.287/ .071	-.068/ .000	-.118/ -.164
	Sig 2 tailed				.000/ .000	.009/ .127	.538/ .997	.284/ .001
	N				83/462	81/466	85/433	84/436
Healthy Intentions	Correlation Coefficient					-.261/ .098	.084/ .044	-.072/ -.079
	Sig 2 tailed					.016/ .034	.430/ .365	.504/ .100
	N					85/466	90/462	89/436

Correlations

Correlations

Correlations

			knowledge score	Risk score	Benefit score	Diet score	Smoke score	total_score
Spearman's rho	knowledge score	Correlation Coefficient	1.000	.118**	.103*	.078	-.079	.006
		Sig. (2-tailed)	.	.009	.023	.086	.082	.896
		N	483	483	483	483	483	440
	Risk score	Correlation Coefficient	.118**	1.000	-.003	.057	.107*	.371**
		Sig. (2-tailed)	.009	.	.950	.212	.019	.000
		N	483	483	483	483	483	440
	Benefit score	Correlation Coefficient	.103*	-.003	1.000	.538**	.009	-.236**
		Sig. (2-tailed)	.023	.950	.	.000	.851	.000
		N	483	483	483	483	483	440
	Diet score	Correlation Coefficient	.078	.057	.538**	1.000	-.022	-.143**
		Sig. (2-tailed)	.086	.212	.000	.	.635	.003
		N	483	483	483	483	483	440
	Smoke score	Correlation Coefficient	-.079	.107*	.009	-.022	1.000	.240**
		Sig. (2-tailed)	.082	.019	.851	.635	.	.000
		N	483	483	483	483	483	440
	total_score	Correlation Coefficient	.006	.371**	-.236**	-.143**	.240**	1.000
		Sig. (2-tailed)	.896	.000	.000	.003	.000	.
		N	440	440	440	440	440	440

** Correlation is significant at the 0.01 level (2-tailed).

* Correlation is significant at the 0.05 level (2-tailed).

For peer review only

Appendix 4.

Item Analysis of published ABCD Risk Questionnaire sub-scales plus 5 unpublished items relating to smoking compared to Item Analysis of recommended edited ABCD Risk Questionnaire sub-scales plus 5 unpublished items relating to smoking.

Table 1. Item Analysis of published ABCD Risk Questionnaire sub-scales plus 5 unpublished items relating to smoking

**Perceived Risk of Heart Attack/ Stroke<br>8 Items<br>Cronbach's Alpha .861<br>(0.84,0.88) 95% CI**	**Inter-item correlation**	**Corrected Item-total correlation**	**Cronbach's alpha if item deleted**
It is likely that I will suffer from a heart attack or stroke in the future	**.832**	.756	.826
It is likely that I will have a heart attack or stroke some time during my life	**.869**	.777	.824
I feel I will suffer a heart attack or stroke some time during my life	**.616**	.784	.824
There is a good chance I will experience a heart attack or stroke in the next 10 years	**.729**	.722	.832
I am not worried that I might have a heart attack or stroke	.403	.624	.843
My chances of suffering a heart attack or stroke in the next 10 years are great	.245	.544	.852
It is likely that I will have a heart attack or stroke because of my past/present behaviours	.266	.319	**.876**
I am concerned about the likelihood of having a heart attack or stroke in the near future	.259	.387	**.870**
**Perceived Benefits and Intentions to Change<br>7 items<br>Cronbach's Alpha .801**	**Inter-item correlation**	**Corrected Item-total correlation**	**Cronbach's alpha if item deleted**
I am thinking about exercising at least 2.5 hours a week	**.727**	**.605**	.760
I intend or want to exercise at least 2.5 hours a week	.442	**.651**	.752
When I exercise for at least 2.5 hours a week I am doing something good for the health of my heart	.426	.593	.769
I am confident that I can maintain a healthy weight by exercising at	.294	.452	.790

least 2.5 hours a week within the next 2 months			
I am not thinking about exercising at least 2.5 hours a week	.264	.508	.781
When I eat at least 5 portions of fruit and vegetables a day I am doing something good for the health of my heart	.483	.483	.783
Increasing my exercise to at least 2.5 hours a week will decrease my chances of having a heart attack or stroke	.326	.474	.786
**Healthy Eating Intentions 3 items Cronbach's Alpha .787 (95% CI**	**Inter-item correlation**	**Corrected Item-total correlation**	**Cronbach's alpha if item deleted**
I am confident that I can eat at least 5 portions of fruit and vegetables a day within the next 2 months	.555	.533	**.812**
I am thinking about eating at least 5 portions of fruit and vegetables a day	**.683**	**.732**	.596
I am not thinking about eating at least 5 portions of fruit and vegetables a day	.424	**.624**	.713
**Perceived Benefits and Intentions to Stop Smoking 5 Items Cronbach's Alpha .943 95% CI**	**Inter-item correlation**	**Corrected item-total correlation**	**Cronbach's alpha if item deleted**
I am thinking of stopping smoking within the next 2 months	**.654**	.848	.932
I have reduced or stopped smoking	**.694**	.751	**.949**
I intend or want to stop smoking	**.829**	.906	.919
If I stop smoking it will reduce my chances of having a heart attack or stroke	**.834**	.886	.922
I am not thinking about stopping smoking	**.789**	.872	.925

For peer review only

Table 2. Item Analysis of edited ABCD Risk Questionnaire sub-scales plus 5 unpublished items relating to smoking.

**Perceived Risk of Heart Attack/ Stroke<br>5 Items<br>Cronbach's Alpha .86 (0.84,0.88) 95% CI<br>Omega 0.85 (0.83, 0.88) 95% CI**	**Inter-item correlation**	**Corrected Item-total correlation**	**Cronbach's alpha if item deleted**
It is likely that I will have a heart attack or stroke some time during my life	**.869**	.777	.824
There is a good chance I will experience a heart attack or stroke in the next 10 years	**.729**	.722	.832
I am not worried that I might have a heart attack or stroke	.403	.624	.843
It is likely that I will have a heart attack or stroke because of my past/present behaviours	.266	.319	**.876**
I am concerned about the likelihood of having a heart attack or stroke in the near future	.259	.387	**.870**
**Perceived Benefits and Intentions to Change<br>6 items<br>Cronbach's Alpha .84 (.81-.86) 95% CI<br>Omega 0.82 (0.78, 0.85) 95% CI**	**Inter-item correlation**	**Corrected Item-total correlation**	**Cronbach's alpha if item deleted**
I am thinking about exercising at least 2.5 hours a week	**.727**	**.605**	.760
I intend or want to exercise at least 2.5 hours a week	.442	**.651**	.752
When I exercise for at least 2.5 hours a week I am doing something good for the health of my heart	.426	.593	.769
I am confident that I can maintain a healthy weight by exercising at least 2.5 hours a week within the next 2 months	.294	.452	.790
I am not thinking about exercising at least 2.5 hours a week	.264	.508	.781
Increasing my exercise to at least 2.5 hours a week will decrease my chances of having a heart attack or stroke	.326	.474	.786
**Healthy Eating Intentions<br>4 items**	**Inter-item correlation**	**Corrected Item-total correlation**	**Cronbach's alpha if item deleted**

**Cronbach's Alpha .84 (.81-.86) 95% CI**<br>**Omega 0.84 (0.81, 0.88) 95% CI**			
I am confident that I can eat at least 5 portions of fruit and vegetables a day within the next 2 months	.555	.533	**.812**
I am thinking about eating at least 5 portions of fruit and vegetables a day	**.683**	**.732**	.596
I am not thinking about eating at least 5 portions of fruit and vegetables a day	.424	**.624**	.713
When I eat at least 5 portions of fruit and vegetables a day I am doing something good for the health of my heart	.483	.483	.783
**Smoking Intentions**<br>**5 items**<br>**Cronbach's Alpha .85 (.83-.87) 95% CI**<br>**Omega 0.84 (0.81, 0.91) 95% CI**	**Inter-item correlation**	**Corrected Item-total correlation**	**Cronbach's alpha if item deleted**
I am thinking of stopping smoking within the next 2 months	**.654**	.848	.932
I have reduced or stopped smoking	**.694**	.751	**.949**
I intend or want to stop smoking	**.829**	.906	.919
If I stop smoking it will reduce my chances of having a heart attack or stroke	**.834**	.886	.922
I am not thinking about stopping smoking	**.789**	.872	.925

For peer review only

Appendix 5. Figures and factor results tables

Without smoking items

Non-missing samples: 420

Bartlett's Test of Sphericity (X2 = 4235.007, p-value < 0.001)

The overall KMO is 0.82, which is within the recommended range (0.8 to 1).

EFA results

- The root mean square of the residuals (RMSR) is 0.05
- Tucker Lewis Index of factoring reliability = 0.77
- RMSEA index = 0.121 and the 90 % confidence intervals are 0.113 0.129
- BIC = 165.35

Scree plot

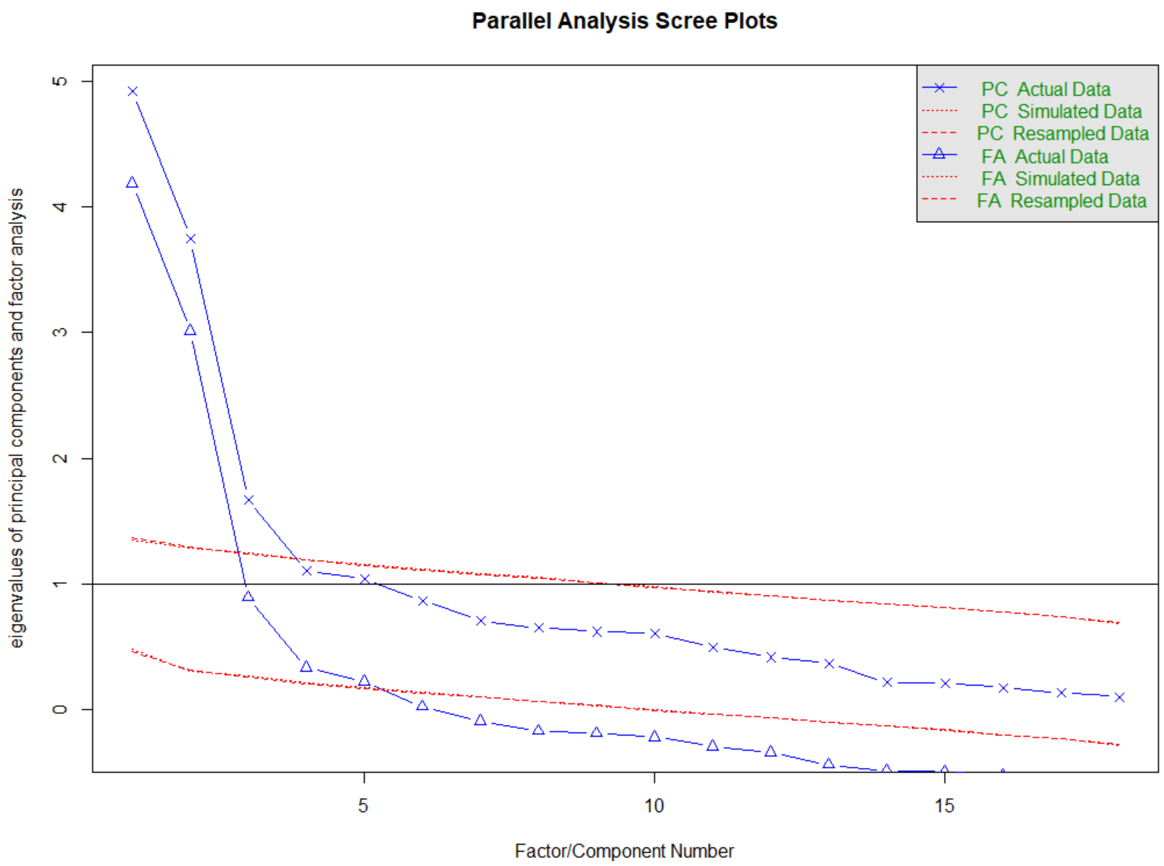

Figure 1. 18-item ABCD Questionnaire results (without smoking items)

Table A1 (a). Factor loadings of the exploratory factor analysis of the risk scale without the smoking items

Items	Factor2	Factor1	Factor3	communality	uniqueness
I feel I will suffer from a heart attack or stroke sometime during my life	0.86	0.02	-0.03	0.74	0.26
It is likely that I will suffer from a heart attack or stroke in the future	0.91	0.05	0.00	0.82	0.18
It is likely that I will have a heart attack or stroke sometime during my life	0.88	0.01	0.01	0.77	0.23
There is a good chance I will experience a heart attack or stroke in the next 10 years	0.73	-0.07	0.01	0.55	0.45
My chances of suffering from a heart attack or stroke in the next 10 years are great	0.65	-0.10	0.01	0.44	0.56
It is likely I will have a heart attack or stroke because of my past and/or present behaviors	0.56	-0.03	-0.01	0.32	0.68
I am not worried that I might have a heart attack or stroke (Reverse coded)	0.28	-0.11	0.10	0.10	0.90
I am concerned about the likelihood of having a heart attack or stroke in the near future	0.40	-0.02	0.11	0.16	0.84
I am thinking about exercising at least 2.5 hours a week	-0.02	0.87	-0.06	0.73	0.27
I intend or want to exercise at least 2.5 hours a week	-0.01	0.91	-0.04	0.80	0.20
When I exercise for at least 2.5 hours a week I am doing something good for the health of my heart	0.02	0.69	0.10	0.53	0.47
I am confident that I can maintain a healthy weight by exercising at least 2.5 hours a week	-0.05	0.45	0.19	0.31	0.69
I am not thinking about exercising for 2.5 hours a week (Reverse coded)	0.04	0.56	0.05	0.34	0.66
When I eat five portions of fruit and vegetables a day I am doing something good for the health of my heart	0.02	0.37	0.35	0.36	0.64
Increasing my exercise to at least 2.5 hours a week will decrease my chances of having a heart attack or stroke	0.02	0.39	0.27	0.30	0.70
I am confident that I can eat at least five portions of fruit and vegetables a day within the next two months	-0.04	0.07	0.64	0.46	0.54
I am thinking about eating at least five portions of fruit and vegetables a day	0.01	-0.01	0.93	0.85	0.15
I am not thinking about eating at least five portions of fruit and vegetables a day (Reverse coded)	-0.01	-0.03	0.78	0.60	0.40

Table A1 (b): Summary of factor loadings and variance distribution of the risk scale without the smoking items

Measures	Factor 2	Factor 1	Factor 3
SS loadings	3.86	3.04	2.28
Proportion Var	0.21	0.17	0.13

For peer review only

Cumulative Var	0.21	0.38	0.51
Proportion Explained	0.42	0.33	0.25
Cumulative Proportion	0.42	0.75	1.00

With smoking items

Non-missing samples: 88

The overall KMO is 0.78, which is slightly below the recommended range (0.8 to 1).

The Bartlet's test of Sphericity is significant (X2 = 1223.459, p-value < 0.001), indicating the sample adequacy for factor analysis.

EFA results

- The root mean square of the residuals (RMSR) is 0.06
- Tucker Lewis Index of factoring reliability = 0.69
- RMSEA index = 0.129 and the 90 % confidence intervals are 0.124 and 0.136
- BIC = 440.9

Scree plot

For peer

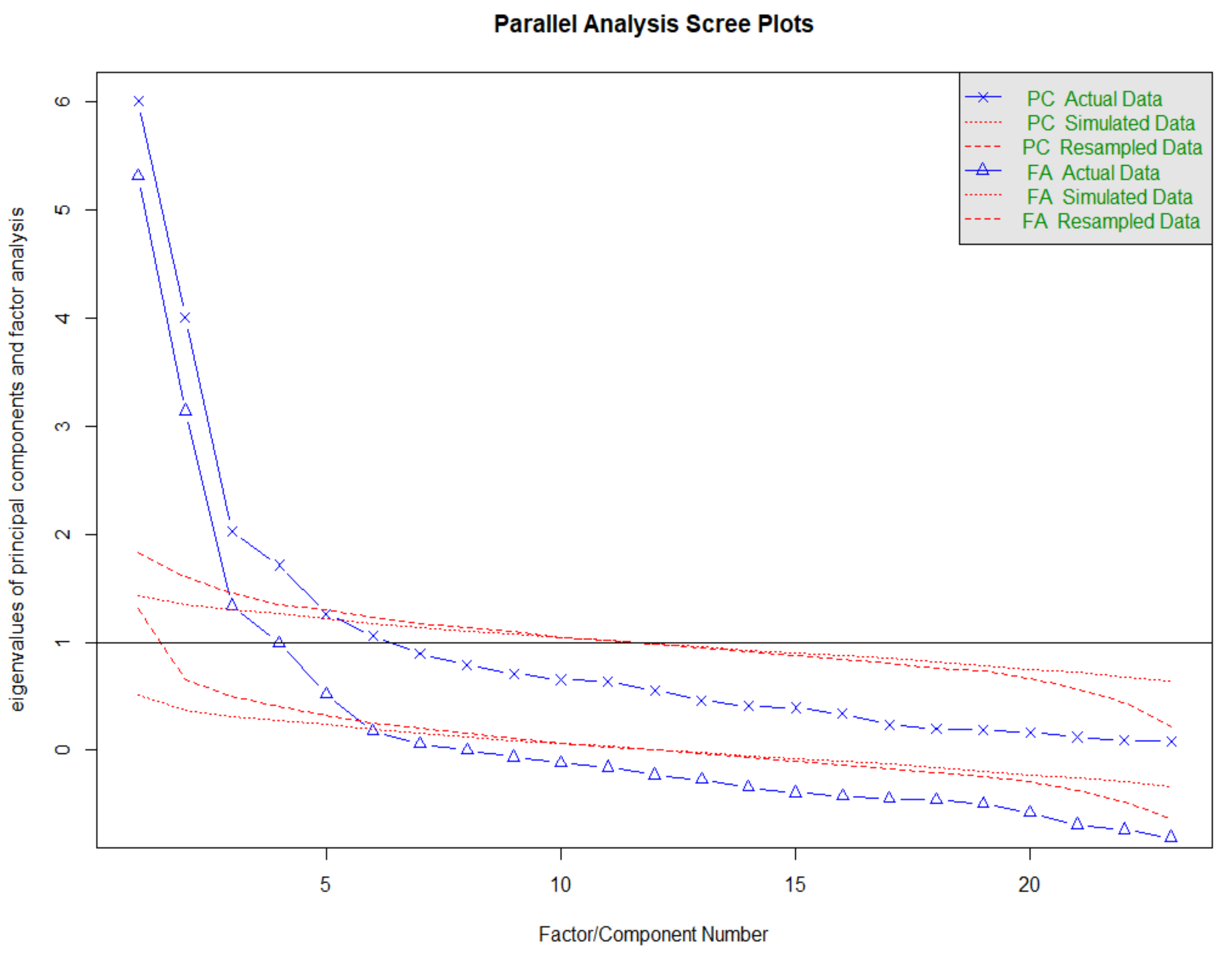

Figure 2. Modified ABCD Questionnaire 23 items with smoking.

Table A2 (a). Factor loadings of the exploratory factor analysis of the risk scale with the smoking items

Items	Factor2	Factor3	Factor1	Factor4	Communality	Uniqueness
I feel I will suffer from a heart attack or stroke sometime during my life	0.86	-0.1	0.05	-0.02	0.76	0.24
It is likely that I will suffer from a heart attack or stroke in the future	0.91	0.06	0.02	-0.01	0.82	0.18
It is likely that I will have a heart attack or stroke sometime during my life	0.88	0.02	0	0	0.77	0.23
There is a good chance I will experience a heart attack or stroke in the next 10 years	0.72	0	-0.09	0.01	0.54	0.46
My chances of suffering from a heart attack or stroke in the next 10 years are great	0.64	-0.03	-0.1	0.01	0.45	0.55
It is likely I will have a heart attack or stroke because of my past and/or present behaviors	0.57	-0.07	0	0	0.33	0.67
I am not worried that I might have a heart attack or stroke (Reverse coded)	0.28	0.02	-0.14	0.1	0.1	0.9
I am concerned about the likelihood of having a heart attack or stroke in the near future	0.41	0.19	-0.12	0.08	0.19	0.81
I am thinking about exercising at least 2.5 hours a week	-0.03	-0.05	0.88	-0.02	0.73	0.27
I intend or want to exercise at least 2.5 hours a week	-0.02	0.05	0.87	-0.02	0.79	0.21
When I exercise for at least 2.5 hours a week I am doing something good for the health of my heart	0.03	0.17	0.62	0.09	0.55	0.45
I am confident that I can maintain a healthy weight by exercising at least 2.5 hours a week	-0.05	0.09	0.42	0.18	0.32	0.68
I am not thinking about exercising for 2.5 hours a week (Reverse coded)	0.02	0	0.53	0.09	0.33	0.67
When I eat five portions of fruit and vegetables a day I am doing something good for the health of my heart	0.04	0.07	0.35	0.35	0.36	0.64
Increasing my exercise to at least 2.5 hours a week will decrease my chances of having a heart attack or stroke	0.04	0.12	0.37	0.24	0.32	0.68
I am confident that I can eat at least five portions of fruit and vegetables a day within the next two months	-0.04	-0.05	0.12	0.64	0.45	0.55
I am thinking about eating at least five portions of fruit and vegetables a day	0.01	0	0.02	0.89	0.8	0.2
I am not thinking about eating at least five portions of fruit and vegetables a day (Reverse coded)	-0.01	0	-0.06	0.83	0.66	0.34
I am thinking of stopping smoking within two months	0.06	0.78	0.12	-0.06	0.67	0.33

For peer review only

I have reduced or stopped smoking	-0.03	0.83	0.02	-0.01	0.71	0.29
I intend or want to stop smoking	-0.05	0.9	-0.02	-0.01	0.8	0.2
If I stop smoking it will reduce my chances of having a heart attack or stroke	0.16	0.58	0.09	0.08	0.43	0.57
I am not thinking about stopping smoking	-0.12	0.56	-0.2	0.17	0.35	0.65

Table A2 (b): Summary of factor loadings and variance distribution of the risk scale with the smoking items

Measures	Factor 2	Factor 3	Factor 1	Factor 4
SS loadings	3.90	3.00	2.97	2.33
Proportion Var	0.17	0.13	0.13	0.10
Cumulative Var	0.17	0.30	0.43	0.53
Proportion Explained	0.32	0.25	0.24	0.19
Cumulative Proportion	0.32	0.57	0.81	1.00

For peer review only

Modified scale (20-items including the smoking items)

Non-missing samples: 89

The overall KMO is 0.79, which is slightly below the recommended range (0.8 to 1).

The Bartlet's test of Sphericity is significant (X2 = 915.41, p-value < 0.001), indicating the sample adequacy for factor analysis.

EFA results

- The root mean square of the residuals (RMSR) is 0.06
- Tucker Lewis Index of factoring reliability = 0.72
- RMSEA index = 0.118 and the 90 % confidence intervals are 0.111 and 0.126
- BIC = 153.72

Scree plot

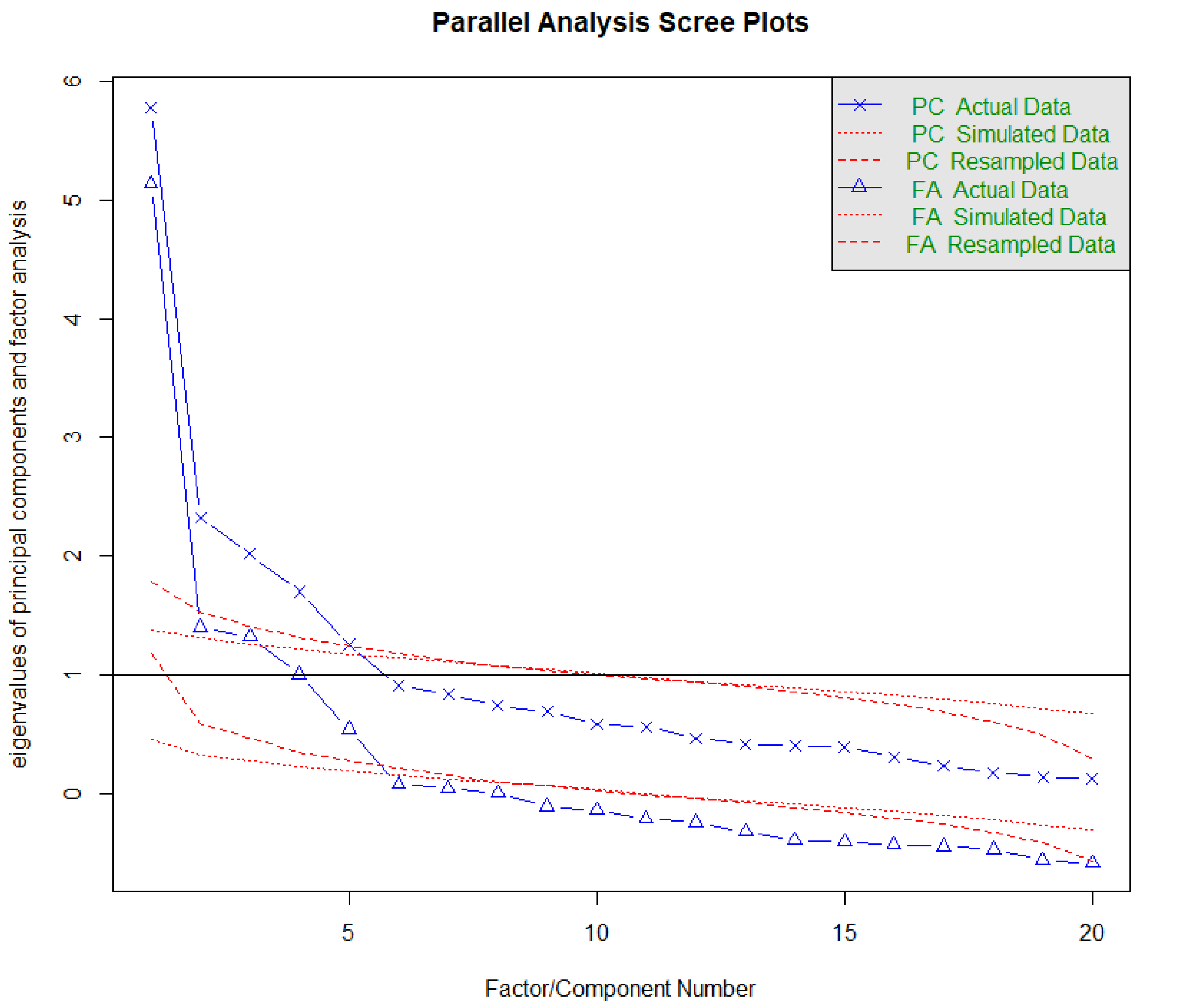

Figure 3. Modified ABCD Questionnaire 20 items with smoking.

w only

Table A3 (a). Factor loadings of the exploratory factor analysis of the modified risk scale (20 items including the smoking items)

Items	Factor3	Factor1	Factor4	Factor2	Communality	Uniqueness
I feel I will suffer from a heart attack or stroke sometime during my life	-0.08	0.04	-0.03	0.76	0.60	0.40
There is a good chance I will experience a heart attack or stroke in the next 10 years	0.02	-0.08	-0.01	0.68	0.48	0.52
It is likely I will have a heart attack or stroke because of my past and/or present behaviors	-0.04	0.01	-0.01	0.61	0.38	0.62
I am not worried that I might have a heart attack or stroke (Reverse coded)	0.04	-0.13	0.10	0.35	0.14	0.86
I am concerned about the likelihood of having a heart attack or stroke in the near future	0.22	-0.11	0.07	0.45	0.23	0.77
I am thinking about exercising at least 2.5 hours a week	-0.06	0.88	-0.02	-0.04	0.74	0.26
I intend or want to exercise at least 2.5 hours a week	0.05	0.87	-0.02	-0.02	0.79	0.21
When I exercise for at least 2.5 hours a week I am doing something good for the health of my heart	0.17	0.62	0.09	0.04	0.55	0.45
I am confident that I can maintain a healthy weight by exercising at least 2.5 hours a week	0.09	0.42	0.18	-0.06	0.32	0.68
I am not thinking about exercising for 2.5 hours a week (Reverse coded)	0.01	0.53	0.09	0.03	0.32	0.68
When I eat five portions of fruit and vegetables a day I am doing something good for the health of my heart	0.08	0.35	0.35	0.07	0.37	0.63
Increasing my exercise to at least 2.5 hours a week will decrease my chances of having a heart attack or stroke	0.13	0.37	0.24	0.06	0.32	0.68
I am confident that I can eat at least five portions of fruit and vegetables a day within the next two months	-0.06	0.12	0.64	-0.05	0.46	0.54
I am thinking about eating at least five portions of fruit and vegetables a day	0.00	0.02	0.89	0.01	0.80	0.20
I am not thinking about eating at least five portions of fruit and vegetables a day (Reverse coded)	0.00	-0.06	0.83	-0.01	0.67	0.33
I am thinking of stopping smoking within two months	0.78	0.12	-0.06	0.04	0.66	0.34
I have reduced or stopped smoking	0.83	0.02	-0.01	-0.03	0.70	0.30
I intend or want to stop smoking	0.89	-0.02	-0.01	-0.07	0.80	0.20
If I stop smoking it will reduce my chances of having a heart attack or stroke	0.59	0.10	0.07	0.18	0.43	0.57
I am not thinking about stopping smoking	0.56	-0.20	0.17	-0.10	0.34	0.66

For peer review only

Table A3 (b): Summary of factor loadings and variance distribution of the modified risk scale (20 items including the smoking items)

**Measures**	**Factor3**	**Factor1**	**Factor4**	**Factor2**
SS loadings	3.00	2.96	2.33	1.80
Proportion Var	0.15	0.15	0.12	0.09
Cumulative Var	0.15	0.30	0.41	0.50
Proportion Explained	0.30	0.29	0.23	0.18
Cumulative Proportion	0.30	0.59	0.82	1.00

For peer review only

Appendix 6. Characteristics of the sample population

**Population Characteristics**		**N**	**% total**
**Gender**	Male	218	49.8
	Female	220	50.2
**Age Group**	18-30	78	17.8
	30-39	80	18.3
	40-49	82	18.7
	50-59	99	22.6
	60-74	78	17.8
	74+	53	12.1
**Deprivation**	IMD1- least deprived	84	17.98
	IMD2	55	11.77
	IMD3	83	17.77
	IMD4	89	19.05
	IMD5- most deprived	156	33.4

For peer review only

Appendix 7. Modified ABCD Risk Questionnaire

Mark Bowyer, Hamid Hassen

Scale	Items	Coding
**Perceived Risk of Heart Attack or Stroke**	1. It is likely that I will have a heart attack or stroke sometime in my life	4= Strongly disagree, 3= Disagree, 2= Agree, 1= Strongly Agree; N/A= 0
	2. There is a good chance I will experience a heart attack or stroke in the next 10 years	4= Strongly disagree, 3= Disagree, 2= Agree, 1= Strongly Agree; N/A= 0
	3. It is (more) likely I will have a heart attack or stroke because of my past and/or present behaviours	4= Strongly disagree, 3= Disagree, 2= Agree, 1= Strongly Agree; N/A= 0
	4. I am not worried that I might have a heart attack or stroke	**REVERSE CODED** 4= Strongly disagree, 3= Disagree, 2= Agree, 1= Strongly Agree; N/A= 0
	5. I am concerned about the likelihood of having a heart attack or stroke in the near future	4= Strongly disagree, 3= Disagree, 2= Agree, 1= Strongly Agree; N/A= 0
**Perceived Benefits and Intentions to Exercise**	6. I am thinking about exercising at least 2.5 hours a week	4= Strongly disagree, 3= Disagree, 2= Agree, 1= Strongly Agree; N/A= 0
	7. I intend or want to exercise at least 2.5 hours a week	4= Strongly disagree, 3= Disagree, 2= Agree, 1= Strongly Agree; N/A= 0
	8. When I exercise for at least 2.5 hours a week I am doing something good for the health of my heart	4= Strongly disagree, 3= Disagree, 2= Agree, 1= Strongly Agree; N/A= 0
	9. I am confident that I can maintain a healthy weight by exercising at least 2.5 hours a week	4= Strongly disagree, 3= Disagree, 2= Agree, 1= Strongly Agree; N/A= 0
	10. I am not thinking about exercising for 2.5 hours a week	**REVERSE CODED** 4= Strongly disagree, 3= Disagree, 2= Agree, 1= Strongly Agree; N/A= 0
	11. Increasing my exercise to at least 2.5 hours a week will decrease my chances of having a heart attack or stroke	4= Strongly disagree, 3= Disagree, 2= Agree, 1= Strongly Agree; N/A= 0

For peer review only

**Perceived Benefit and Healthy Eating Intentions**	12. I am confident that I can eat at least five portions of fruit and vegetables a day within the next two months	4= Strongly disagree, 3= Disagree, 2= Agree, 1= Strongly Agree; N/A= 0
	13. I am thinking about eating at least five portions of fruit and vegetables a day	4= Strongly disagree, 3= Disagree, 2= Agree, 1= Strongly Agree; N/A= 0
	14. I am not thinking about eating at least five portions of fruit and vegetables a day	**REVERSE CODED** 4= Strongly disagree, 3= Disagree, 2= Agree, 1= Strongly Agree; N/A= 0
	15. When I eat five portions of fruit and vegetables a day I am doing something good for the health of my heart	4= Strongly disagree, 3= Disagree, 2= Agree, 1= Strongly Agree; N/A= 0
**Benefits and Intentions to Stop Smoking**	16. I am thinking of stopping smoking within two months	4= Strongly disagree, 3= Disagree, 2= Agree, 1= Strongly Agree; N/A= 0
	17. I have reduced or stopped smoking	4= Strongly disagree, 3= Disagree, 2= Agree, 1= Strongly Agree; N/A= 0
	18. I intend or want to stop smoking	4= Strongly disagree, 3= Disagree, 2= Agree, 1= Strongly Agree; N/A= 0
	19. If I stop smoking it will reduce my chances of having a heart attack or stroke	4= Strongly disagree, 3= Disagree, 2= Agree, 1= Strongly Agree; N/A= 0
	20. I am not thinking about stopping smoking	**REVERSE CODED** 4= Strongly disagree, 3= Disagree, 2= Agree, 1= Strongly Agree; N/A= 0

For peer review only

Reporting checklist for cross sectional study.

Based on the STROBE cross sectional guidelines.

Instructions to authors

Complete this checklist by entering the page numbers from your manuscript where readers will find each of the items listed below.

Your article may not currently address all the items on the checklist. Please modify your text to include the missing information. If you are certain that an item does not apply, please write "n/a" and provide a short explanation.

Upload your completed checklist as an extra file when you submit to a journal.

In your methods section, say that you used the STROBE cross sectionalreporting guidelines, and cite them as:

von Elm E, Altman DG, Egger M, Pocock SJ, Gotzsche PC, Vandenbroucke JP. The Strengthening the Reporting of Observational Studies in Epidemiology (STROBE) Statement: guidelines for reporting observational studies.

		Reporting Item	Page Number
**Title and abstract**			
Title	#1a	Indicate the study's design with a commonly used term in the title or the abstract	1
Abstract	#1b	Provide in the abstract an informative and balanced summary of what was done and what was found	1
**Introduction**			
Background / rationale	#2	Explain the scientific background and rationale for the investigation being reported	3
Objectives	#3	State specific objectives, including any prespecified hypotheses	3
**Methods**			
Study design	#4	Present key elements of study design early in the	4

For peer review only

		paper	
Setting	#5	Describe the setting, locations, and relevant dates, including periods of recruitment, exposure, follow-up, and data collection	4
Eligibility criteria	#6a	Give the eligibility criteria, and the sources and methods of selection of participants.	4
	#7	Clearly define all outcomes, exposures, predictors, potential confounders, and effect modifiers. Give diagnostic criteria, if applicable	6
Data sources / measurement	#8	For each variable of interest give sources of data and details of methods of assessment (measurement). Describe comparability of assessment methods if there is more than one group. Give information separately for for exposed and unexposed groups if applicable.	6
Bias	#9	Describe any efforts to address potential sources of bias	7
Study size	#10	Explain how the study size was arrived at	7
Quantitative variables	#11	Explain how quantitative variables were handled in the analyses. If applicable, describe which groupings were chosen, and why	7
Statistical methods	#12a	Describe all statistical methods, including those used to control for confounding	7
Statistical methods	#12b	Describe any methods used to examine subgroups and interactions	7
Statistical methods	#12c	Explain how missing data were addressed	7
Statistical methods	#12d	If applicable, describe analytical methods taking account of sampling strategy	7
Statistical methods	#12e	Describe any sensitivity analyses	7
**Results**			
Participants	#13a	Report numbers of individuals at each stage of study—	7

For peer review only

		eg numbers potentially eligible, examined for eligibility, confirmed eligible, included in the study, completing follow-up, and analysed. Give information separately for for exposed and unexposed groups if applicable.	
Participants	#13b	Give reasons for non-participation at each stage	7
Participants	#13c	Consider use of a flow diagram	n/a No drop-out
Descriptive data	#14a	Give characteristics of study participants (eg demographic, clinical, social) and information on exposures and potential confounders. Give information separately for exposed and unexposed groups if applicable.	7
Descriptive data	#14b	Indicate number of participants with missing data for each variable of interest	7
Outcome data	#15	Report numbers of outcome events or summary measures. Give information separately for exposed and unexposed groups if applicable.	7
Main results	#16a	Give unadjusted estimates and, if applicable, confounder-adjusted estimates and their precision (eg, 95% confidence interval). Make clear which confounders were adjusted for and why they were included	8
Main results	#16b	Report category boundaries when continuous variables were categorized	n/a Continuous variables not measured
Main results	#16c	If relevant, consider translating estimates of relative risk into absolute risk for a meaningful time period	n/a No measurement of risk
Other analyses	#17	Report other analyses done—e.g., analyses of subgroups and interactions, and sensitivity analyses	10
**Discussion**			
Key results	#18	Summarise key results with reference to study objectives	12
Limitations	#19	Discuss limitations of the study, taking into account	12

For peer review only

		sources of potential bias or imprecision. Discuss both direction and magnitude of any potential bias.	
Interpretation	#20	Give a cautious overall interpretation considering objectives, limitations, multiplicity of analyses, results from similar studies, and other relevant evidence.	12
Generalisability	#21	Discuss the generalisability (external validity) of the study results	13
**Other Information**			
Funding	#22	Give the source of funding and the role of the funders for the present study and, if applicable, for the original study on which the present article is based	1

Notes:

- 13c: n/a No drop-out
- 16b: n/a Continuous variables not measured
- 16c: n/a No measurement of risk The STROBE checklist is distributed under the terms of the Creative Commons Attribution License CC-BY. This checklist was completed on 08. June 2021 using https://www.goodreports.org/, a tool made by the EQUATOR Network in collaboration with Penelope.ai

For peer review only

BMJ Open

Psychometric evaluation of the 'Attitudes and Beliefs about Cardiovascular Disease (ABCD) Risk Questionnaire' with validation of a previously untested 'Intentions and Beliefs around Smoking' sub-scale.

Journal:	*BMJ Open*
Manuscript ID	bmjopen-2021-054532.R4
Article Type:	Original research
Date Submitted by the Author:	07-Oct-2022
Complete List of Authors:	Bowyer, Mark; Nottingham Trent University, Institute of Health and Allied Professions, School of Social Sciences<br>Hassen, Hamid; University of Antwerp, Family Medicine and Population Health, Faculty of Medicine and Health Services<br>Bastiaens, Hilde; University of Antwerp Faculty of Medicine and Health Sciences, Family Medicine and Population Health<br>Gibson, Linda ; Nottingham Trent University, Institute of Health and Allied Professions, School of Social Sciences
**Primary Subject Heading**:	Public health
Secondary Subject Heading:	Cardiovascular medicine, Smoking and tobacco
Keywords:	PUBLIC HEALTH, STATISTICS & RESEARCH METHODS, PREVENTIVE MEDICINE

SCHOLARONE™
Manuscripts

BMJ

I, the Submitting Author has the right to grant and does grant on behalf of all authors of the Work (as defined in the below author licence), an exclusive licence and/or a non-exclusive licence for contributions from authors who are: i) UK Crown employees; ii) where BMJ has agreed a CC-BY licence shall apply, and/or iii) in accordance with the terms applicable for US Federal Government officers or employees acting as part of their official duties; on a worldwide, perpetual, irrevocable, royalty-free basis to BMJ Publishing Group Ltd ("BMJ") its licensees and where the relevant Journal is co-owned by BMJ to the co-owners of the Journal, to publish the Work in this journal and any other BMJ products and to exploit all rights, as set out in our licence.

The Submitting Author accepts and understands that any supply made under these terms is made by BMJ to the Submitting Author unless you are acting as an employee on behalf of your employer or a postgraduate student of an affiliated institution which is paying any applicable article publishing charge ("APC") for Open Access articles. Where the Submitting Author wishes to make the Work available on an Open Access basis (and intends to pay the relevant APC), the terms of reuse of such Open Access shall be governed by a Creative Commons licence – details of these licences and which Creative Commons licence will apply to this Work are set out in our licence referred to above.

Other than as permitted in any relevant BMJ Author's Self Archiving Policies, I confirm this Work has not been accepted for publication elsewhere, is not being considered for publication elsewhere and does not duplicate material already published. I confirm all authors consent to publication of this Work and authorise the granting of this licence.

For peer review only

TITLE PAGE

Psychometric evaluation of the 'Attitudes and Beliefs about Cardiovascular Disease (ABCD) Risk Questionnaire' with validation of a previously untested 'Intentions and Beliefs around Smoking' sub-scale.

Mark Bowyer, Nottingham Trent University (Corresponding Author)

mark.bowyer@ntu.ac.uk (**ORCID** 0000-0002-1474-5711)

Nottingham Trent University School of Social Sciences

Chaucer Building, Burton Street

Nottingham NG1 4BT

Tel: (+1) 7786 993405 Fax: (+1)115 8485574

Hamid Yimam Hassen, University of Antwerp, Belgium

hamid.hassen@uantwerpen.be (**ORCID** 0000-0001-6485-4193)

Dr Hilde Bastiaens (Participating Investigator)
Associate professor
Dept Family Medicine and Population Health
Faculty of Medicine and Health Sciences
University of Antwerp

Tel: 0032 (0)3 265.29.10 Fax: 0032 (0)3 265.25.26 Hilde.bastiaens@uantwerpen.be

Dr Linda Gibson (Participating Investigator)

Professor in Public Health, Institute of Health & Allied Professions

Nottingham Trent University Linda.gibson@ntu.ac.uk

Key words

Cardiovascular Diseases

- Cardiovascular risk factors
- Instrumentation

Psychometrics

- Surveys and questionnaires
- Instrumentation

Primary prevention

- Instrumentation

Word count 4421

For peer review only

Psychometric evaluation of the 'Attitudes and Beliefs about Cardiovascular Disease (ABCD) Risk Questionnaire' with validation of a previously untested 'Intentions and Beliefs around Smoking' sub-scale.

ABSTRACT

Objectives:

To provide evidence of validity, reliability and generalisability of results obtained using the Attitudes and Beliefs about Cardiovascular Disease (ABCD) Risk Questionnaire with a sample of the English population surveyed within the 'SPICES' Horizon 2020 project (Nottingham study site), and to specifically evaluate the psychometric and factor properties of an as-yet untested 5 item sub-scale relating to smoking behaviours.

Design and setting:

Community and workplace-based cross-sectional study in Nottingham, UK.

Participants:

466 English adults fitting inclusion criteria (aged 18+, without known history of CVD, not pregnant, able to provide informed consent) participated in the study.

Methods:

We re-validated the ABCD questionnaire on a sample of the general population in Nottingham to confirm the psychometric properties. Furthermore, we introduced 5 items related to smoking which were dropped in the original study due to inadequate valid samples.

Primary and secondary outcome measures:

1. Psychometric and factor performance of untested 5 item 'smoking behaviours' sub-scale
2. Psychometric and factorial properties in combination with the remaining 18 items across 3 sub-scales

Results:

Analyses of the data largely confirmed the validity, reliability, and factor structure of the original ABCD Risk Questionnaire. Sufficient participants in our study provided data against an additional five smoking related items to confirm their validity as a sub-scale and to advocate for their inclusion in future applications of the scale. EFA and CFA calculations support some minor changes to the remaining sub-scales which may further improve psychometric performance and therefore generalisability of the instrument.

Conclusions:

An amended version of the ABCD Risk Questionnaire would provide public health researchers and practitioners with a brief, easy to use, reliable and valid survey tool. The amended tool may assist public health practitioners and researchers to survey patient or public intentions and beliefs around three key areas of individually modifiable risk (Physical Activity, Diet, Smoking).

For peer review only - http://bmjopen.bmj.com/site/about/guidelines.xhtml

Trial registration:

ISRCTN68334579 https://doi.org/10.1186/ISRCTN68334579
Heart health without a doctor: an implementation study of CVD prevention and behaviour change interventions in community settings

Ethical approval

Ethical approval for the 'SPICES' Nottingham study protocol (incorporating the ABCD Risk Questionnaire) was secured from the Nottingham Trent University College of Business, Law and Social Sciences on the 20th February 2019. Participants were required to provide informed consent (Appendix 1).

Article summary

Strengths and Limitations of this study

- Large sample (n=466) of English adults from the Nottingham UK population
- Sufficient case data to validate additional sub-scale related to attitudes and intentions of smokers
- Criterion validity not explored
- Full assessment of the utility of ABCD Risk Questionnaire in health promotion and CVD prevention not explored; further studies may be required to position the tool in clinical and public health practice.
- The planned pre-post intervention measurement and analysis was not possible due to COVID-19 interruption of fieldwork.

Original protocol (Appendix 2)

Funding statement

This work was supported by the European Commission Horizon 2020 Non-communicable diseases and the challenge of healthy ageing Grant agreement 733356 'SPICES'.

Competing interests statement

None declared

Patient and public involvement

Patients and/or the public were not involved in the design, or conduct, or reporting, or dissemination plans of this research.

Patient consent for publication (data sharing agreement)

Not required (participant information and informed consent attached Appendix 1)

Provenance and peer review

Not commissioned.

Data availability statement

Data are available on reasonable request

For peer review only - http://bmjopen.bmj.com/site/about/guidelines.xhtml

Keywords

Cardiovascular diseases- Cardiovascular risk factors

Cardiovascular diseases- Instrumentation

Psychometrics- Instrumentation

Surveys and questionnaires- Instrumentation

Primary prevention- Instrumentation

Author contributions

Following ICMJE recommendations, Mark Bowyer and Hamid Hassen assert authorship based on the following 4 criteria:

Substantial contributions to the conception or design of the work; or the acquisition, analysis, or interpretation of data for the work; AND

Drafting the work or revising it critically for important intellectual content; AND

Final approval of the version to be published; AND

Agreement to be accountable for all aspects of the work in ensuring that questions related to the accuracy or integrity of any part of the work are appropriately investigated and resolved.

Professor Linda Gibson and Professor Hilde Bastiaens assert Participating Investigator status having served as scientific advisors, critically reviewed the study proposal, and participated in writing or technical editing of the manuscript.

Acknowledgements

The authors would like to acknowledge the cooperation of Rolls-Royce plc Hucknall Site; Nottingham City Council Adult Care in providing access to employees. Crabtree Farm Community Centre, Middle Street Resource Centre, Self-Help UK, in facilitating access to members, users and premises.

INTRODUCTION

Scientific Background and Rationale

In the UK, Cardiovascular Disease (CVD) is responsible for over 130,000 deaths per annum.[1] CVD morbidity is also the biggest contributor to the inequalities in Healthy Life Expectancy between members of the wealthiest neighbourhoods and the most deprived.[2] In 2009 the NHS Health Check [3]was established and more recently (2019) the CVD Prevent initiative to implement ‘upstream’ interventions for the prevention of CVD morbidity.[4] Both of these initiatives seek to

For peer review only

improve early case-finding to prevent avoidable strokes and heart attacks. Both recognise the importance of supported lifestyle change in conjunction with drug therapies.

Lifestyle or behavioural change requires a degree of individual agency and commitment which drug therapies do not. Unhealthy lifestyle behaviours are linked to culture and habit, environment, emotions, and confidence which can all moderate an individual's readiness to change and the commitment required to sustain those changes over time.[5] Understanding the attitudes and beliefs that people hold towards diet, exercise and smoking, as well as their perception of their own risk could assist primary care and public health professionals in providing relevant and effective behavioural advice and social prescribing options. To support evaluations of the NHS Health Check programme, in 2017 a questionnaire was developed to evaluate patients' awareness of cardiovascular disease risk at University College London.[6] This ABCD Risk Questionnaire attempts to provide a short survey drawing from the dominant theoretical models of behaviour change (Trans-Theoretical Model, Health Beliefs Model),[7] covering diet, smoking, exercise and alcohol behaviours, and incorporating a conceptual spread of perceived risk from immediate to lifetime. Whilst a range of validated CVD risk questionnaires exist,[8] and it is common to ask patients to self-report their physical activity, dietary and smoking behaviours through questionnaires and diaries, the ABCD Risk Questionnaire usefully investigates the knowledge, perceptions, beliefs and attitudes that govern these behaviours. 'The literature suggests that in order to lower measurement errors, larger sample sizes and respondent: item ratios are necessary, and that replication is required if the sample size is <300. [9] In the original study, item analysis was carried out on a sample of 110. The necessity to reproduce results was recognised by the authors of the original study:

> "Additional studies should be conducted with larger samples to confirm the reliability and validity of the questionnaire. It would be useful to replicate the factor analytic process on an independent, larger sample to confirm the generalisability of these findings." .[10]

Specific Objectives

In this study we re-validated the tool on a sample of the general population in Nottingham to confirm the psychometric properties. Furthermore, we introduced 5 items related to smoking which were dropped in the original study due to inadequate case numbers.

To the best of our knowledge, this is the first study which has incorporated items relating to attitudes and intentions towards stopping smoking into the published version of the ABCD Risk Questionnaire and collected sufficient data to submit them to analysis of validity, reliability and factor structure.

In the original ABCD study, over the course of three stages of validity testing (content, face, reliability) items relating to alcohol use and smoking were rejected, leaving four final sub-scales: Knowledge of CVD Risks; Perceived Risk of Heart Attack/ Stroke; Perceived Benefits and Intentions to Change; and Healthy Eating Intentions. During Exploratory Factor Analysis (EFA) none of the items relating to alcohol use achieved strong enough loadings to be included in the final scale, and items related to smoking could not be included due to the high proportion of missing data in the experimental sample. The authors of the study note this limitation '*the questionnaire does not encompass all aspects of CVD risk observed in the general population*' and that '*future studies examining populations at increased CVD risk can look into incorporating smoking and alcohol into the ABCD Risk Questionnaire to learn about these individuals' preconceptions and attendance of follow-up care*'.[11]

For peer review only - http://bmjopen.bmj.com/site/about/guidelines.xhtml

The present study

Nottingham is one of five global sites of the EU Horizon 2020 'SPICES' [12] CVD prevention implementation study which began in 2017. SPICES investigates contextual and health system barriers to the scaling up of successful behaviour change interventions for improved cardiovascular health in low, middle and high income European countries. The most recent data (2016) indicate that "The prevalence of CVD recorded in Nottingham City GP Practices is significantly less that the national (England) average and in comparable areas, despite the CVD mortality rate being significantly higher than average; this partly reflects the differing age structures of the populations, but also indicates significant under-detection/diagnosis"[13]

The SPICES Nottingham population survey carried out in 2019-20 utilised the ABCD Risk Questionnaire alongside the non-clinical INTERHEART CVD risk prediction instrument.[14] The SPICES study team chose to re-introduce 5 pre-written items relating to 'Intentions and Readiness to Stop Smoking' from the 65 item University College London (UCL) item pool into the questionnaire due to the high prevalence of smoking in the Nottingham population compared to England averages,[15] and its importance as a CVD risk.[16] This created a 31 item questionnaire. 4 items relating to Alcohol intake from the same item pool were also considered for inclusion but omitted on two grounds: alcohol related CVD risk was not a specific focus of the 'SPICES' study; concerns about the time-burden on participants of including the additional items which can be a barrier to participation.

METHODS

Incorporating the ABCD Risk Questionnaire into the SPICES Nottingham baseline survey provided cross-sectional study data across a broad sample of adult participants. The data-set generated was therefore suitable for psychometric validation of the original and modified versions of the ABCD questionnaire. Surveys were administered in-person by researchers in the field during attendance at community venues and workplaces. Administration of the survey took approximately ten minutes including provision of consent, and confidential communication of results another ten minutes on average. Participation was entirely voluntary.

Participants

Participants were recruited from across the Nottingham conurbation between April 2019 and March 2020 as part of the SPICES Nottingham baseline survey.[17] A purposive sampling method was employed based on community and workplace engagement. This strategy had two components:

1. engagement of citizens in neighbourhoods through existing community groups, organisations and venues, and
2. engagement of employees in the workplace through large city-based employers.

Community groups were targeted on the basis of the demographic of their membership to ensure that neighbourhoods of differing mean household income, those who are not in employment or of working age, and those from different ethnicities were included. In this way 327 participants were recruited.

Employers were targeted on the basis of workforce size, and policies relating to workforce well-being. Nottingham City Council Adult Care teams and the Rolls-Royce plc Hucknall site both responded positively and between them provided 156 participants. NTU researchers administered the SPICES Nottingham baseline survey individually within the community or workplace setting and

For peer review only - http://bmjopen.bmj.com/site/about/guidelines.xhtml

personalised feedback about CVD risks was provided confidentially once the survey had been completed.

Criteria for inclusion included being aged 18+, resident in Nottinghamshire, not previously diagnosed with a heart condition, not pregnant, and able to provide informed consent.

Materials

The SPICES baseline survey incorporated the ABCD risk questionnaire into a digitised survey instrument created in the Research Electronic Data Capture (REDCap) database system,[18] a secure web application for building and managing online surveys and databases, and the online survey responses were uploaded automatically. No participant data was stored on local devices. Both the ABCD Risk Questionnaire (Table 1) and the non-laboratory INTERHEART questionnaire were included unchanged from their published versions apart from an additional 5 items pertaining to smoking behaviour (Table 2).[19]

Table 1. Published ABCD Risk Questionnaire

Scale	Items
**Knowledge**<br><br>True/False/Don't Know<br><br>Correct score =1<br>Incorrect/ Don't know score = 0<br><br>Higher sum score= more knowledgeable/ more correct about having a heart attack or stroke	1. One of the main causes of heart attack and stroke is stress
	2. Walking and gardening are considered types of exercise that can lower the risk of having a heart attack or stroke
	3. Moderately intense activity of 2.5 hours a week will reduce your chances of having a heart attack or stroke
	4. People who have diabetes are at higher risk of heart attack or stroke
	5. Managing your stress levels will help you to manage your blood pressure
	6. Drinking high levels of alcohol can increase your cholesterol and triglyceride levels
	7. HDL refers to 'good' cholesterol, and LDL refers to 'bad' cholesterol
	8. A family history of heart disease is not a risk factor for high blood pressure
**Perceived Risk of Heart Attack or Stroke**<br><br>4= Strongly disagree, 3= Disagree, 2= Agree, 1= Strongly Agree; N/A= 0<br><br>Higher sum score = higher perception of risk of having a heart attack or stroke	9. I feel I will suffer from a heart attack or stroke sometime during my life
	10. It is likely that I will suffer from a heart attack or stroke in the future
	11. It is likely that I will have a heart attack or stroke some time during my life
	12. There is a good chance I will experience a heart attack or stroke in the next 10 years
	13. My chances of suffering from a heart attack or stroke in the next 10 years are great
	14. It is likely I will have a heart attack or stroke because of my past and/or present behaviours
	15. I am not worried that I might have a heart attack or stroke (**Reverse coded**)
	16. I am concerned about the likelihood of having a heart attack or stroke in the near future

For peer review only - http://bmjopen.bmj.com/site/about/guidelines.xhtml

**Perceived Benefits and Intentions to Change**<br><br>4= Strongly disagree, 3= Disagree, 2= Agree, 1= Strongly Agree; N/A= 0<br><br>Higher average score = Higher perceived benefits of diet and exercise and higher perceived readiness for change in regards to exercise and behaviour	17. I am thinking about exercising at least 2.5 hours a week
	18. I intend or want to exercise at least 2.5 hours a week
	19. When I exercise for at least 2.5 hours a week I am doing something good for the health of my heart
	20. I am confident that I can maintain a healthy weight by exercising at least 2.5 hours a week
	21. I am not thinking about exercising for 2.5 hours a week **(Reverse coded)**
	22. When I eat five portions of fruit and vegetables a day I am doing something good for the health of my heart
	23. Increasing my exercise to at least 2.5 hours a week will decrease my chances of having a heart attack or stroke
**Healthy Eating Intentions**<br><br>4= Strongly disagree, 3= Disagree, 2= Agree, 1= Strongly Agree; N/A= 0<br><br>Higher average score = Higher perceived readiness for change with regard to healthy dietary behaviour	24. I am confident that I can eat at least five portions of fruit and vegetables a day within the next two months
	25. I am thinking about eating at least five portions of fruit and vegetables a day
	26. I am not thinking about eating at least five portions of fruit and vegetables a day **(Reverse coded)**

The surveys were administered in the field by a team of trained researchers recruited from the NTU student body and directly supervised by the SPICES Nottingham coordinator. The surveys were accessed using dedicated tablet computers. Items were reproduced word for word and in the same sequence as the original ABCD Risk Questionnaire with the additional 5 smoking items inserted after all 26 original items. The five smoking related items were developed by the authors of the original study through a process of literature review (construct validity), expert panel review (content validity), and modification by focus group (face validity). [20] These five smoking sub-scale items were included in the 65 item pool developed in the original study but omitted from their analysis due to a high proportion of missing responses.[21]

Table 2. Additional 'smoking' sub-scale

**Benefits and Intentions to Stop Smoking**<br><br>4= Strongly disagree, 3= Disagree, 2= Agree, 1= Strongly Agree; N/A= 0<br><br>Higher average score = Higher perceived readiness for change with regard to healthy dietary behaviour	27. I am thinking of stopping smoking within two months
	28. I have reduced or stopped smoking
	29. I intend or want to stop smoking
	30. If I stop smoking it will reduce my chances of having a heart attack or stroke
	31. I am not thinking about stopping smoking

For peer review only

For peer review only - http://bmjopen.bmj.com/site/about/guidelines.xhtml

Validating the sample

The baseline survey dataset was extracted from REDCap for analysis. Sample was checked for representativeness of the Nottingham population across parameters of age, gender, household income and known rates of physical activity and smoking.

Data analysis

We took the published 26-item ABCD Risk Questionnaire, introduced 5 further items relating to smoking behaviours, and administered it alongside a validated CVD risk assessment instrument (INTERHEART) to 486 individuals in Nottingham over a period of 12 months. Item, scale, and factor reliabilities were remeasured to generate a comparison to the results reported in the original study. Correlation was tested between and amongst ABCD sub-scale scores and selected INTERHEART variables, closely matching the methods applied in the original study (Appendix 3) and results were compared accordingly. After removing incomplete responses, 466 valid cases were entered for analysis, four times the sample size of the original study.

Item and sub-scale reliabilities were tested using inter-item correlations, corrected item-total correlations and Cronbach's Alpha. [22] We performed an exploratory factor analysis (EFA) to evaluate the dimensionality of items of the original and modified risk scale with and without the smoking items. The EFA was performed using the maximum likelihood extraction and varimax rotation method. [23] Sample and data adequacy was assessed using Kaiser-Meyer-Olkin (KMO) test and Bartlett's test of sphericity was performed to compare an observed correlation matrix to the identity matrix.[24] The adequate number of factors was determined using a scree plot (Appendix 4). To further test the consistency of factors, we tested using Confirmatory Factor Analysis (CFA). We evaluated the model fit of the CFA using; the X2 test, the Tucker-Lewis and Comparative Fit Indexes and the root mean square error of approximation (RMSEA).[25] The analysis was performed using a free statistical software R version 4.0.2. UK postcodes were collected for all participants which allowed them to be sorted into income deciles using Office for National Statistics Index of Multiple Deprivation (IMD) public datasets, allowing correlations to be analysed. Following the methods used in the original study, case data from the 'Knowledge' sub-scale (8 items) were omitted from the analysis since they utilise a separate response format.[26]

We used the STROBE cross sectional checklist when writing our report.[27]

RESULTS

Participants

Participation was voluntary, and self-selection may have been influenced by sensitivities around disclosure of health status and lifestyle habits forming a barrier to those with co-morbidities and socially 'questionable' behaviours (heavy smoking, high alcohol intake).

The sample cohort has a 49:51 percent gender split, normal distribution of age ranges (18-92), and a distribution of Socio-Economic Status (SES) which reflects known data about neighbourhood income in Nottingham. Nottingham is the 11th most deprived district in England with higher unemployment, lower education and skills, and shorter life expectancy than the national averages. [28] Using the Index of Multiple Deprivation a relative measure of deprivation across seven domains, Health and Disability is the domain on which the city's scores are lowest compared to the rest of England.

For peer review only - http://bmjopen.bmj.com/site/about/guidelines.xhtml

Nevertheless, the mean INTERHEART predicted risk score for all 466 participants was 10.32 which closely matches the global reported mean for the instrument.[29]

Smoking sub-scale

The percentage of smokers in our sample was 15.5%. The proportion of smokers in our sample was therefore higher than the 2019 England average (13.9%), and lower than the Nottingham city population average (20.6%) based on the ONS Annual Population Survey.[30] ONS notes that smoking prevalence estimates by local authority can fluctuate due to smaller sample sizes. Our SPICES Nottingham sample cohort also includes some participants from neighbouring Local Authorities with different recorded rates of smoking.

The five items in the smoking subscale are measured on the same four-point response scale as the 18 items submitted for Factor Analysis in the original published ABCD Risk Questionnaire (Strongly agree, agree, disagree, strongly disagree, and not applicable).

With the original 18 items this 'Not Applicable' response option was not used by any of the SPICES Nottingham study participants. By contrast, within their responses to the items in the 'smoking' subscale, 'Not Applicable' was the modal answer. Participants chose the 'N/A' response option whenever they reported being a non-smoker. This mirrors the behaviour of the original 110 NHS Health Check attendees who formed the pilot sample cohort for the original study, leaving an insufficient number of smokers in the sample to assess validity and reliability of smoking sub-scale items. To reduce measurement error in item and factorial analysis, it is recommended to over-determine the ratio of variables to items/factors by utilising larger sample sizes. No hard rule exists, but at least 10 respondents for each scale item is usually recommended. [31] In the original study, there were insufficient smokers in the sample to achieve this ratio and consequently the smoking sub-scale items were omitted from the analysis. In the present study, 88 smokers were recorded within the sample and we were therefore able to proceed with item and factorial analysis of the five smoking sub-scale items.

Sub-scale Alpha values, Cronbach's Alpha if item deleted calculated for all items, inter-item correlations and corrected item-total correlations were all calculated, mirroring the analysis reported in the original study (Appendix 5).

Interitem correlations calculated for these five items produced a range between 0.654 and 0.834. All of these five 'smoking' items therefore correlate with one another more strongly than recommended (<.6) and were considered for rejection. However, we found each item to be qualitatively different, and that the differences were conceptually clear and well expressed in the item wording so that no participant could be expected to confuse one with any other, and they were retained.

Discrimination was confirmed using item-total correlations. These fell between the range 0.751 and 0.906 meaning that all five 'smoking' sub-scale items are comfortably above the standard cut-off for acceptability of 0.3.

EFA was carried out twice, firstly with all cases, and then again with 88 confirmed smoking cases. The first operation ensured that factor loadings were not skewed by the lower number of cases reporting smoking behaviours, the second ensured that factor loadings for the remaining sub-scales where more case data was available were not skewed by outliers.

Exploratory Factor Analysis:

For peer review only - http://bmjopen.bmj.com/site/about/guidelines.xhtml

We conducted EFA on the original 18-item risk perception questionnaire and the modified 23-item (with smoking items). For the original 18-item, a total of 420 observations were included in the analysis, which was sufficient for factor analysis as indicated with KMO of 0.82, which is within the recommended range (0.8 to 1). The Bartlett's Test of Sphericity was significant (X2 = 4235.007, p-value < 0.001) indicating the data is adequate for factor analysis. As a result, a three-factor solution emerged based on the Scree plot (figure 1), accounting 57.4% of the total variance. Factor loading patterns in the present analysis slightly varied from the original subscales. The domains in the original subscales were risk perception, benefit finding and healthy eating intentions. In our analysis, Item 14 (‘*When I eat at least 5 portions of fruit and vegetables a day I am doing something good for the health of my heart’*) showed a better loading to healthy eating intention, which was loaded to benefit finding in the original study (Appendix 5).

For the modified 23-item (including the smoking sub-scale), 88 samples were valid and included in the analysis. The KMO was 0.78, which was slightly below the recommended range, but Bartlett’s Test of Sphericity was significant (X2 = 1223.459, p-value < 0.001), indicating adequacy for factor analysis. The analysis showed that the smoking items loaded to another latent construct resulting in four factors in total (figure 2).

Confirmatory Factor Analysis of the published ABCD Risk Questionnaire

A Confirmatory Factor Analysis was undertaken using the SPICES Nottingham dataset to investigate further. Conducting CFA allowed us to construct the sub-scales of the published ABCD Risk Questionnaire in a three-factor measurement model and test its fit against relevant indices. Original 18 item survey comprising three sub-scales (Perceived Risk of Heart Attack/Stroke 8 items; Perceived Benefits and Intentions to Change 7 items; Healthy Eating Intentions 3 items) were used to create measurement model in SPSS Amos 25. The model was then updated to include an additional 5 item sub-scale relating to smoking behaviours.

Editing the measurement model

The CFA measurement model was then reconstructed removing items which had confused participants and generated high inter-item correlations, and additionally re-assigning an item relating to dietary behaviour into the dietary behaviour sub-scale (Table 3). This resulted in a four-factor model (Perceived Risk of Heart Attack/ Stroke’ 6 items; ‘Perceived Benefits and Intentions to Exercise’ 6 items; ‘Healthy Eating Intentions’ 4 items, Perceived Benefits and Intentions to Reduce Smoking’ 5 items). Analysis properties were set to Estimation: Maximum Likelihood. A scree-plot of this amended four-factor version of the questionnaire was also plotted (Figure 3).

For peer review only - http://bmjopen.bmj.com/site/about/guidelines.xhtml

Table 3. CFA fit indices for the original and modified ABCD Questionnaire measurement models

**Original 18 item ABCD** In the original study of 2017, 18 items were entered into factor analysis. This Confirmatory Factor Analysis tests the fit of these original items to their structure using the larger Nottingham SPICES dataset.						
CMIN	P	CMIN/DF	TLI	CFI	RMSEA	RMR
714.941	.000	5.416	.826	.850	.097	.049
**Original 18 item ABCD with 5 Smoking items added** In the original study of 2017, items relating to smoking behaviours were developed but could not be included in the published scale due to insufficient data. In the Nottingham SPICES study sufficient observations were made to test these smoking items.						
CMIN	P	CMIN/DF	TLI	CFI	RMSEA	RMR
994.931	.000	4.442	.865	.881	.086	.049
**Edited 20 item ABCD with Smoking sub-scale** As discussed above, independent item analysis and Exploratory factor Analysis using the independent SPICES Nottingham dataset revealed issues with the continued inclusion of some of the original 'perception of risk' sub-scale items, and the allocation of an item relating to dietary behaviours in the physical activity behaviours sub-scale. The published ABCD questionnaire was edited to remove or re-assign the problematic items and retested using Confirmatory Factor Analysis.						
CMIN	P	CMIN/DF	TLI	CFI	RMSEA	RMR
638.973	.000	3.896	.881	.897	.079	.052
**Modified 20 item ABCD with Smoking sub-scale** The measurement model created for the Confirmatory Factor Analysis was modified so that items within each ABCD sub-scale were set to co-vary with one another.						
CMIN	P	CMIN/DF	TLI	CFI	RMSEA	RMR
385.312	.000	2.439	.941	.951	.056	.046

For peer review only

Similarly, in the 23-item factor analysis, item 14 was loaded to the healthy eating intention. The model fit indices showed a slight improvement as indicated in table 3.

Based on factor loading, inter-item correlations, and face validity results, we also tested a slightly shorter version of the questionnaire, 20-items including five smoking items and the result shows that the model fit improved (CFI=0.941; TLI=0.951; RMSEA=0.056, SRMR=0.046).

The three published factors achieved a poor fit in CFA (Table 3). Including the five smoking related items which had performed strongly in EFA as their own latent factor improved overall model fit slightly, but not to an acceptable level.

Modification of the measurement model

For peer review only - http://bmjopen.bmj.com/site/about/guidelines.xhtml

Reviewing modification indices and expected parameter changes for factor loadings and measurement intercepts we observed an extreme covariance value (116.812) and parameter change (.209) between two of the risk perception items ('there is a good chance that I will experience a heart attack or stroke in the next 10 years' and 'my chances of suffering a heart attack or stroke in the next 10 years are great') which had caused confusion for participants in our study.

Removing one of these two items (item #13), and the two other duplicative items (items #9 & #10) from the 'perceived risk of heart attack or stroke' sub-scale retains the conceptual spread of risk embodied by the items (lifetime, 10 year, near future, behaviour related). Moving the diet related item (#22) which appears in the 'perceived benefits and intentions to change' over to the 'healthy eating intentions' sub-scale might allow greater clarity for researchers analysing results from the questionnaire. Co-varying items within sub-scales that generated values above 20 (a high cut-off due to large sample used) resulted in acceptable or good fit across all sub-scales. Each of the three behaviour related sub-scales now contain items drawn from HBM, TTM and SE models providing a sound conceptual basis for comparison. Using EFA to check these results shows the modified sub-scale structure performs better than the published version (Figure 3).

For peer review only

Table 4. Amended ABCD Risk Questionnaire

Scale	Items	Coding
**Knowledge**	1. One of the main causes of heart attack and stroke is stress	Correct answers:<br>Q1 - T<br>Q2 – T<br>Q3 – T<br>Q4 – T<br>Q5 – T<br>Q6 – T<br>Q7 – T<br>Q8 – F<br>T = True<br>F = False<br>Correct score = 1,<br>Incorrect or Don't Know: score = 0.
	2. Walking and gardening are considered types of exercise that can lower the risk of having a heart attack or stroke	
	3. Moderately intense activity of 2.5 hours a week is enough to reduce your chances of having a heart attack or stroke	
	4. People who have diabetes are at higher risk of having a heart attack or stroke	
	5. Managing your stress levels will help you to manage your blood pressure	
	6. Drinking high levels of alcohol can increase your cholesterol and triglyceride levels	
	7. HDL refers to 'good' cholesterol, and LDL refers to 'bad' cholesterol	
	8. A family history of heart disease is not a risk factor for high blood pressure	

For peer review only - http://bmjopen.bmj.com/site/about/guidelines.xhtml

**Perceived Risk of Heart Attack or Stroke**	9. It is likely that I will have a heart attack or stroke sometime in my life	4= Strongly disagree, 3= Disagree, 2= Agree, 1= Strongly Agree; N/A= 0
	10. There is a good chance I will experience a heart attack or stroke in the next 10 years	4= Strongly disagree, 3= Disagree, 2= Agree, 1= Strongly Agree; N/A= 0
	11. It is more likely I will have a heart attack or stroke because of my past and/or present behaviours	4= Strongly disagree, 3= Disagree, 2= Agree, 1= Strongly Agree; N/A= 0
	12. I am not worried that I might have a heart attack or stroke	**REVERSE CODED** 4= Strongly disagree, 3= Disagree, 2= Agree, 1= Strongly Agree; N/A= 0
	13. I am concerned about the likelihood of having a heart attack or stroke in the near future	4= Strongly disagree, 3= Disagree, 2= Agree, 1= Strongly Agree; N/A= 0
**Perceived Benefits and Intentions to Exercise**	14. I am thinking about exercising at least 2.5 hours a week	4= Strongly disagree, 3= Disagree, 2= Agree, 1= Strongly Agree; N/A= 0
	15. I intend or want to exercise at least 2.5 hours a week	4= Strongly disagree, 3= Disagree, 2= Agree, 1= Strongly Agree; N/A= 0
	16. When I exercise for at least 2.5 hours a week I am doing something good for the health of my heart	4= Strongly disagree, 3= Disagree, 2= Agree, 1= Strongly Agree; N/A= 0
	17. I am confident that I can maintain a healthy weight by exercising at least 2.5 hours a week	4= Strongly disagree, 3= Disagree, 2= Agree, 1= Strongly Agree; N/A= 0
	18. I am not thinking about exercising for 2.5 hours a week	**REVERSE CODED** 4= Strongly disagree, 3= Disagree, 2= Agree, 1= Strongly Agree; N/A= 0
	19. Increasing my exercise to at least 2.5 hours a week will decrease my chances of having a heart attack or stroke	4= Strongly disagree, 3= Disagree, 2= Agree, 1= Strongly Agree; N/A= 0
**Perceived Benefit and Healthy Eating Intentions**	20. I am confident that I can eat at least five portions of fruit and vegetables a day within the next two months	4= Strongly disagree, 3= Disagree, 2= Agree, 1= Strongly Agree; N/A= 0
	21. I am thinking about eating at least five portions of fruit and vegetables a day	4= Strongly disagree, 3= Disagree, 2= Agree, 1= Strongly Agree; N/A= 0

For peer review only - http://bmjopen.bmj.com/site/about/guidelines.xhtml

For peer review only

	22. I am not thinking about eating at least five portions of fruit and vegetables a day	**REVERSE CODED** 4= Strongly disagree, 3= Disagree, 2= Agree, 1= Strongly Agree; N/A= 0
	23. When I eat five portions of fruit and vegetables a day I am doing something good for the health of my heart	4= Strongly disagree, 3= Disagree, 2= Agree, 1= Strongly Agree; N/A= 0
**Benefits and Intentions to Stop Smoking**	24. I am thinking of stopping smoking within two months	4= Strongly disagree, 3= Disagree, 2= Agree, 1= Strongly Agree; N/A= 0
	25. I have reduced or stopped smoking	4= Strongly disagree, 3= Disagree, 2= Agree, 1= Strongly Agree; N/A= 0
	26. I intend or want to stop smoking	4= Strongly disagree, 3= Disagree, 2= Agree, 1= Strongly Agree; N/A= 0
	27. If I stop smoking it will reduce my chances of having a heart attack or stroke	4= Strongly disagree, 3= Disagree, 2= Agree, 1= Strongly Agree; N/A= 0
	28. I am not thinking about stopping smoking	**REVERSE CODED** 4= Strongly disagree, 3= Disagree, 2= Agree, 1= Strongly Agree; N/A= 0

Other results

Analysing results from ABCD sub-scales recorded within our sample indicated that mean knowledge of CVD risk factors was 79% and recognition of the benefits of changing behaviour was 85%, but this barely correlated against objectively measured risk (-.164, sig .001 n=436).

DISCUSSION

Inadequate knowledge and/or a gap between perceived and actual CVD risk in the population could be an obstacle to better health outcomes. Improving an individual's CVD knowledge and risk perception may be important in improving a healthy lifestyle. Measuring CVD knowledge and risk perception may be a method to initiate a healthy lifestyle intervention as well as to monitor and evaluate the impact of interventions. Following this rationale, Woringer and colleagues developed the ABCD Risk questionnaire in order to measure CVD knowledge and risk perception. In this study, we re-validated the tool on a sample of the general population in Nottingham to confirm the psychometric properties.

The 88 participants in this study who reported smoking is a low number for pilot testing of psychometric scales but it does exceed a 10:1 ratio of cases to variables making it reasonable to proceed to analysis.

Based on EFA and CFA, we confirmed a three-factor structure, which closely matched the results reported in the original study, but differed in certain important respects. Item 14 ('*When I eat at*

For peer review only - http://bmjopen.bmj.com/site/about/guidelines.xhtml

For peer review only

least 5 portions of fruit and vegetables a day I am doing something good for the health of my heart") showed a better loading to the 'healthy eating intentions' sub-scale, in contrast to the factor loading in the original study, which placed this item in 'perceived benefits and intentions to change'. This is the only item which loaded onto a different sub-scale when using the Nottingham dataset, all others continued to load onto their original factors although many of these loaded weakly and failed to meet usual thresholds for validity (Appendix 5). The larger numbers of participants in our dataset (466 compared to 110) provides statistical confidence in the new results, and we therefore modelled this revised allocation of items and factors alongside the original factor allocations in the subsequent Confirmatory Factor Analysis. The revised measurement model with item 14 allocated to 'Healthy Eating Intentions' indicated a better fit in CFA results.

These results suggest that the additional five smoking items perform acceptably and should be incorporated into future applications of the ABCD Risk Questionnaire.

Limitations

Our purposive sampling strategy was non-probabilistic but the resulting sample distribution reflects the population characteristics of Nottingham (Appendix 6) and therefore permits the generalisation of results to similar urban centres. Because random sampling was not employed, it is not possible to generalise the findings further to a wider population.

Psychometric performance based on reliability calculations and factorial analysis is not an end in itself. The resulting scale has to have some utility in the world and generate results which can add value to existing understanding of beliefs and attitudes to cardiovascular disease risk. The literature refers to a 'know-do' gap in health education which is framed as a knowledge translation challenge from research to practice. [32] Analysing results from the ABCD Risk Questionnaire, our findings indicate that this gap also exists within patients/ study participants who have recorded high levels of knowledge and motivation to moderate unhealthy behaviours but low levels of success in doing so. This suggests that health education may be failing to stimulate healthy changes in this population, and that other factors (addiction/dependence/social acceptance/lack of resources/time sensitivity) may be limiting the impact of health education even as knowledge of risks and remedies is high. The ABCD Risk Questionnaire enables a careful exploration of the relationships between knowledge, motivation, attitudes and beliefs in relation to CVD risks and their remedies which may in future be combined with investigation of these confounding factors to improve the effectiveness of future health promotion strategies.

Other observations

Researchers in the Nottingham SPICES team administering the questionnaire during fieldwork reported that three items within the 'Perception of Risk of Heart Attack/Stroke' sub-scale caused consistent difficulties for respondents due to apparent duplication and confusion over fine semantic differences. It was difficult for participants to see a semantic difference between statements 9, 10, 11, and 12, 13 respectively. For items 9, 10, and 11, if we agree that *suffer from* and *have* are synonymous, it is hard to differentiate between *in the future* and *some time during my life* because you would imagine that respondents will be thinking about the future in both cases.

For the questionnaire to be reliable across all sections of the population, including those with limited ability in English (whether native or non-native, first, second or additional language, etc.) who may find it particularly hard to differentiate with any confidence between different pairs/sets of statements with largely synonymous meanings, this confusion is a problem. Items 12 and 13 seem to differ mainly only in the possible interpretation of a difference of degree between *good* and *great.*

For peer review only - http://bmjopen.bmj.com/site/about/guidelines.xhtml

These face validity issues and their impact can be observed in the inter-item correlation results generated during item reliability analysis. In the original study, two items in the perception of risk sub-scale had been rejected due to correlations in excess of 0.6 leaving 8 items. Of these remaining 8 items half had inter-item correlations which exceeded 0.6 when tested against the Nottingham dataset. These were items 9, 10, 11, and 12 which generated inter-item correlation values of .832, .869, .616, and .729 respectively. Removing items 9, 10, and 13 does not reduce the conceptual range of the 'perception of risk' subscale which is framed temporally from immediate threat to lifetime risk, it simply removes the duplicate or confusing items. Testing this shortened scale with factor analysis strengthens both item and scale reliability and improves factor loadings (Appendix 5). We recommend that future versions of the English language ABCD Risk Questionnaire adopt these edits (Table 4/Appendix 7).

CONCLUSIONS

The published English language version of the ABCD Risk Questionnaire, with the removal of three problematic 'perception' items, the shift of one item from the 'perceived benefits and intentions to change' sub-scale into the 'healthy eating intentions' sub-scale, and the addition of a 5 item 'smoking' sub-scale performs sufficiently well in validity, reliability and factor analysis with an independent, larger sample to confirm the generalisability of its original published findings. This result supports continued use of the ABCD Risk Questionnaire in the field of CVD prevention research and practice. The inclusion of a smoking behaviours sub-scale is likely to increase its relevance where smoking behaviours still account for a large proportion of individually modifiable CVD risk in a target population. Although criterion validity has now been established for the 'Perception of risk of heart attack/stroke sub-scale' by two published studies, [33] the utility of the remaining sub-scales individually or in combination has been under-examined. Future studies should investigate the criterion validity of these sub-scales and the conceptual strength of the items and variables from which they have been composed in order to unambiguously position the resulting survey instrument and evaluate its utility in CVD prevention and treatment practices. Neither this study or the original published study of 2017 was able to conduct pre-post intervention measurements in their study design. Measuring using the ABCD survey before an intervention (such as the NHS Health Check) and then again at some time afterwards- in tandem with a validated CVD risk prediction scale (such as INTERHEART or Q Risk 2) would help to establish the ABCD Risk Questionnaire's sensitivity to change, and perhaps also its ability to discern between types of respondent.

REFERENCES

1 https://www.bhf.org.uk/bhf-cvd-statistics-uk-factsheet.pdf (Accessed 10/05/2021)
2 https://www.england.nhs.uk/ourwork/clinical-policy/cvd/ (Accessed 10/05/2021)
3 Robson J, Dostal I, Sheikh A, *et al* The NHS Health Check in England: an evaluation of the first 4 years *BMJ Open* 2016;**6:**e008840. doi: 10.1136/bmjopen-2015-008840
4 Kearney M Prevention and treatment of CVD: a new priority for the NHS *Heart* 2019;**105:**1924.
5 Michie, S., van Stralen, M.M. & West, R. The behaviour change wheel: A new method for characterising and designing behaviour change interventions. *Implementation Sci* **6,** 42 (2011). https://doi.org/10.1186/1748-5908-6-42
6 Woringer M, Nielsen JJ, Zibarras L, *et al*

For peer review only - http://bmjopen.bmj.com/site/about/guidelines.xhtml

Development of a questionnaire to evaluate patients' awareness of cardiovascular disease risk in England's National Health Service Health Check preventive cardiovascular programme *BMJ Open* 2017;**7:**e014413. doi: 10.1136/bmjopen-2016-014413

7 Health Behavior: Theory, Research, and Practice edited by Karen Glanz, Barbara K. Rimer, K. Viswanath

8 Gorenoi, V. and Hagen, A., 2015. Overview of risk-estimation tools for primary prevention of cardiovascular diseases in European populations. *Central European journal of public health*, *23*(2), p.91.

9 Guadagnoli E, Velicer WF. Relation of sample size to the stability of component patterns. *Am Psychol Assoc.* (1988) 103:265–75. 10.1037/0033-2909.103.2.265

10 Woringer M, Nielsen JJ, Zibarras L*, et al* IBID

11 https://www.nottinghaminsight.org.uk/themes/health-and-wellbeing/joint-strategic-needs-assessment/adults/cardiovascular-disease-2016/ (accessed 29/11/21)

11 Woringer M, Nielsen JJ, Zibarras L*, et al* IBID

12 Nahar, P., van Marwijk, H., Gibson, L. *et al.* A protocol paper: community engagement interventions for cardiovascular disease prevention in socially disadvantaged populations in the UK: an implementation research study. *glob health res policy* **5,** 12 (2020). https://doi.org/10.1186/s41256-020-0131-1

13 https://www.nottinghaminsight.org.uk/themes/health-and-wellbeing/joint-strategic-needs-assessment/adults/smoking-and-tobacco-control-2019/ (accessed 29/11/21)

14 Yusuf S , Hawken S , Ounpuu S , *et al* . Effect of potentially modifiable risk factors associated with myocardial infarction in 52 countries (the INTERHEART study): case-control study. Lancet 2004;**364**:937–52.doi:10.1016/S0140-6736(04)17018-9

15 https://www.nottinghaminsight.org.uk/themes/health-and-wellbeing/joint-strategic-needs-assessment/adults/smoking-and-tobacco-control-2019/ (Accessed 10/05/21)

16 John A Ambrose, Rajat S Barua, The pathophysiology of cigarette smoking and cardiovascular disease: An update, Journal of the American College of Cardiology, Volume 43, Issue 10, 2004, Pages 1731-1737, ISSN 0735-1097, https://doi.org/10.1016/j.jacc.2003.12.047.

16 Woringer M, Nielsen JJ, Zibarras L*, et al* IBID

17 Nahar, P., van Marwijk, H., Gibson, L. *et al.* IBID

18 Harris, P.A., et al., Research electronic data capture (REDCap)—A metadata-driven methodology and workflow process for providing translational research informatics support. Journal of Biomedical Informatics, 2009. **42**(2): p. 377-381 DOI: https://doi.org/10.1016/j.jbi.2008.08.010.

19 Woringer M, Nielsen JJ, Zibarras L*, et al* IBID

20 Woringer M, Nielsen JJ, Zibarras L*, et al* IBID

21 Woringer M, Nielsen JJ, Zibarras L*, et al* IBID

22 Cronbach, L.J. Coefficient alpha and the internal structure of tests. *Psychometrika* **16,** 297–334 (1951). https://doi.org/10.1007/BF02310555

23 Ferguson, E. and Cox, T. (1993), Exploratory Factor Analysis: A Users'Guide. International Journal of Selection and Assessment, 1: 84-94. https://doi.org/10.1111/j.1468-2389.1993.tb00092.x

24 Kaiser, H.F. The varimax criterion for analytic rotation in factor analysis. Psychometrika 23, 187–200 (1958). https://doi.org/10.1007/BF02289233

25 Bartlett, M. S. (1951). The effect of standardization on a Chi-square approximation in factor analysis. Biometrika, 38(3/4), 337-344

26 Hu Lt, Bentler PM. Cutoff criteria for fit indexes in covariance structure analysis: Conventional criteria versus new alternatives. Structural Equation Modeling: A Multidisciplinary Journal. 1999;6(1):1-55. doi: 10.1080/10705519909540118.

27 von Elm E, Altman DG, Egger M, Pocock SJ, Gotzsche PC, Vandenbroucke JP. The Strengthening the Reporting of Observational Studies in Epidemiology (STROBE) Statement: guidelines for reporting observational studies.

28 Ministry of Housing, Communities & Local Government. The English indices of deprivation 2019 statistical release https://www.gov.uk/government/statistics/english-indices-of-deprivation-2019 (accessed 10/05/2021)

29 Yusuf S , Hawken S , Ounpuu S , *et al* **IBID**

30 UK Government Office for National Statistics 2019 https://www.ons.gov.uk/peoplepopulationandcommunity/healthandsocialcare/healthandlifeexpectancies/bulletins/adultsomkinghabitsgreatbritain/2018 (Accessed 10/05/2021)

31 Nunnally JC. *Pyschometric Theory*. New York, NY: McGraw-Hill; (1978).

32 Ward PR, Thompson J, Barber R, et al. Critical perspectives on 'consumer involvement' in health research: Epistemological dissonance and the know-do gap. Journal of Sociology. 2010;46(1):63-82. doi:10.1177/1440783309351771

For peer review only - http://bmjopen.bmj.com/site/about/guidelines.xhtml

33 Woringer M, Nielsen JJ, Zibarras L, *et al* IBID & Martos T, Csabai M, Bagyura Z, *et al* Cardiovascular disease risk perception in a Hungarian community sample: psychometric evaluation of the ABCD Risk Perception Questionnaire *BMJ Open* 2020;**10:**e036028. doi: 10.1136/bmjopen-2019-036028

Figure legends

Figure 1. Scree plot of factor eigenvalues (original published 18 items) Nottingham dataset

Figure 2. Scree plot of factor eigenvalues (original published 18 items plus 5 smoking items) Nottingham dataset

Figure 3. Scree plot of factor eigenvalues (recommended amended ABCD) Nottingham dataset

For peer review only

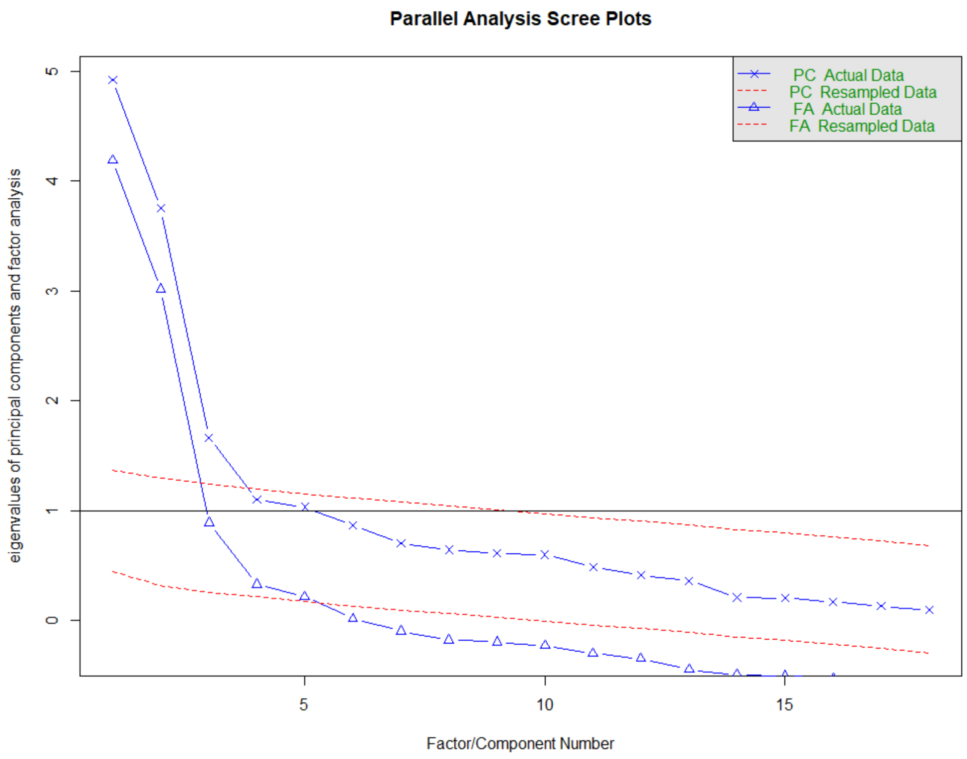

Figure 1. Scree plot of factor eigenvalues (original published 18 items)

266x211mm (96 x 96 DPI)

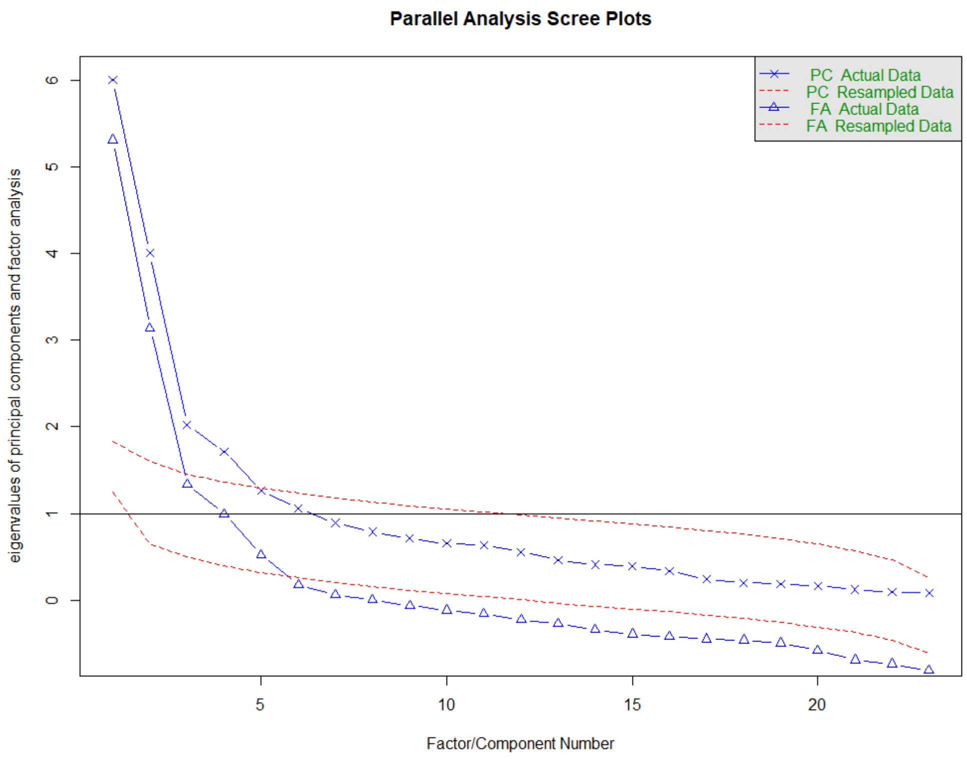

Figure 2. Scree plot of factor eigenvalues (original published 18 items plus 5 smoking items)

266x211mm (96 x 96 DPI)

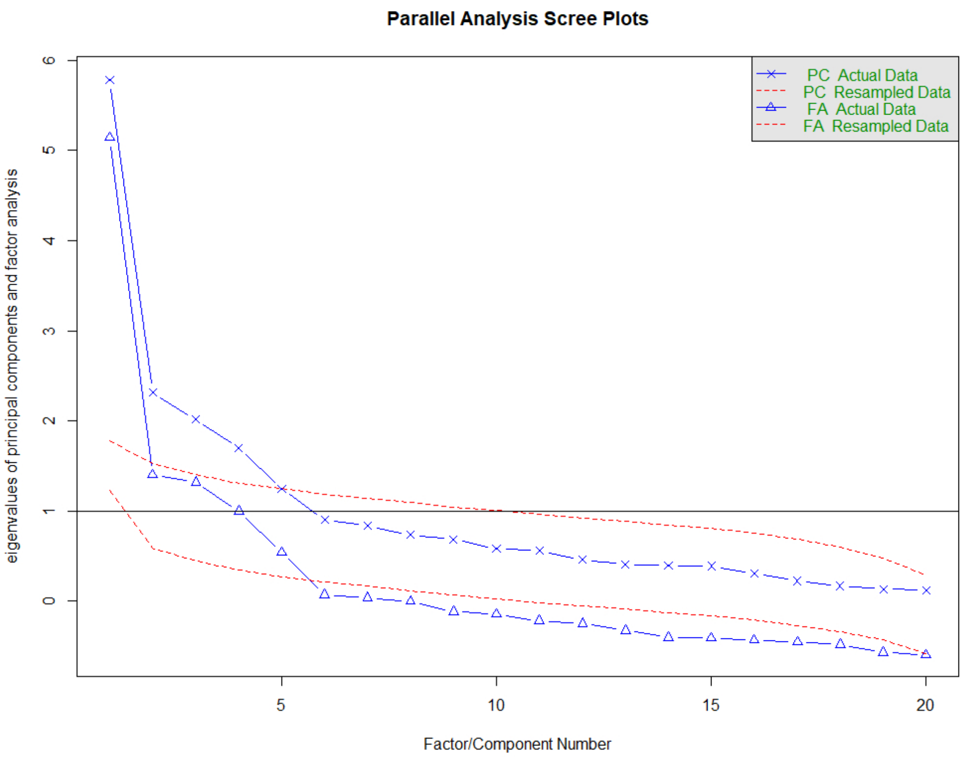

Figure 3. Scree plot of factor eigenvalues (recommended amended ABCD)

266x211mm (96 x 96 DPI)

Horizon 2020
European Union funding
for Research & Innovation

NOTTINGHAM
TRENT UNIVERSITY

'SPICES' Heart Diseases Prevention Research

Introduction to SPICES research

Nottingham Trent University is part of an international research team investigating ways to build good practice in the prevention of Heart Diseases. Researchers and doctors have a lot of evidence about what causes heart diseases and what prevents them. Heart Diseases are now the biggest cause of death globally, and one of the leading causes of disability, so the more people know what the doctors know, the better they can protect themselves and maintain a good quality of life.

The research project is called 'SPICES' and here in Nottingham we are going to see if working with people in the community instead of at the doctor's surgery, we can spread the message quicker and further.

If you choose to take part we will ask you to complete a simple survey. From the we will be able see how well you are looking after your heart in terms of your lifestyle. Then there will be three possible options:

If the data you provide suggests you may need to make some lifestyle changes we will recommend that you make an appointment to see your doctor. As researchers we cannot give any medical advice, but it would be inappropriate for us to ignore any signs of an unhealthy lifestyle that could give rise to heart problems.

If the data you provide suggests you have a healthy lifestyle, then this is positive news and we'll talk to you about how you might be able to help the project in other ways.

If you are somewhere in the middle we will show you some simple ways to reduce your risk and stay healthier for longer.

N.B. In all cases, the data you provided is for research purposes only and a decision about your health cannot be made on the basis of questionnaires only.   Whilst we advise you to see a doctor if figures are high, lower figures should not be taken to indicate a healthy heart, and the results should not be used to replace medical assessments and the taking of medical advice about other health monitoring strategies. The dividing of participants into three groups is for research purposes only and is not a medical intervention.

If you're interested please complete our survey (It might take about 10 minutes, and you will need a tape measure for one of the questions).

Our researchers will then get in touch with you about ways that we can support you to make your heart healthier.   Any information we collect will be kept securely and not shared outside of the research team. Your name and personal details will not be used in any reports, and all our records will be destroyed at the end of the project in line with the relevant GDPR legislation. Additionally you may withdraw your data at any time up to but no later than December 31st 2020 by contacting Mark Bowyer, SPICES Coordinator, Nottingham Trent University 0115 8485574 mark.bowyer@ntu.ac.uk

OK? Let's start with your agreement to take part.

For peer review only

Horizon 2020
European Union funding
for Research & Innovation

NOTTINGHAM
TRENT UNIVERSITY

CONSENT FORM

'SPICES' Heart Diseases Prevention Research

You are making a decision to take part. By ticking ALL statements and signing your name below you will indicate that you have read the information provided above and decided to participate.

If you choose to discontinue participation in this study, you may withdraw at any time without judgement, or effect on your status.

**CONSENT STATEMENT**		**Please tick if you agree**
**1.**	I have received, read and understood the SPICES participant information sheet	
**2.**	I am aware that I can withdraw my participation at any time without prejudice, judgement or effect on my status in relation to Nottingham Trent University or its research partners	
**3.**	I understand that information I provide during my participation can be deleted at my request up to but no later than December 31st 2020	
**4.**	I agree to be contacted by SPICES researchers using the details that I have supplied below	
**5.**	I understand that the collection of data is not part of medical assessment or diagnosis and cannot be relied upon to reach conclusions as to the state of my health	
**5.**	I understand that any information I provide as part of the SPICES research will be managed in accordance with the EU General Data Protection Regulation (GDPR) framework (see SPICES participant information sheet)	
**6.**	I agree to take part in this research project	

Name:

Preferred contact details:

D.O.B.

Gender:

Postcode:

Signature:

Date:

………………………………………………………………………….

Staff signature:

Date:

For peer review only

A Protocol Paper: Community engagement interventions for Cardiovascular Disorders prevention in socially disadvantaged populations in the UK: An implementation research study
Final 15072019

Target Journal: Journal of Global Health Research and Policy
https://ghrp.biomedcentral.com/?gclid=Cj0KCQiA68bhBRCKARIsABYUGifuKd-xktjcmV7tn3r7G-IEqS5rAb6QmiEl6P9dXGBdNRDhsIPVzA0aAiJWEALw_wcB

Papreen Nahar[1], Harm van Marwijk[1], Linda Gibson[2], Geofrey Musinguzi[3], Sibyl Anthierens[4], Elizabeth Ford [1], Stephen A Bremner [1], Mark Bower [2], Jean Yves Le Reste[5], Tholene Sodi [6], Hilde Bastiaens[4]

Corresponding author: Dr Papreen Nahar, Department of Primary Care and Public Health, Brighton and Sussex Medical School, UK. The University of Sussex. E-mail: P.Nahar@bsms.ac.uk

For peer review only

Abstract:

Cardiovascular disorders (CVD) are the single greatest cause of mortality worldwide. In the UK, the National Health Service (NHS) has launched an initiative of health checks over and above current care to tackle CVD. However, the uptake of Health Checks is poor in disadvantaged communities. This protocol paper sets out a UK-based study aiming to co-produce a community delivered CVD risk assessment and coaching intervention to support community members to reduce their risk of CVD.

The overall aim of the project is to implement a tailored-to-context community engagement (CE) intervention on awareness of CVD risks in vulnerable populations in high, middle and low-income countries. This paper describes the protocol for the UK sites in Sussex and Nottingham. The specific objectives of the study are to enhance stakeholder' engagement; to implement lifestyle interventions for cardiovascular primary prevention, in disadvantaged populations and motivate uptake of NHS health checks.

This study takes a mixed methods approach, combining qualitative and quantitative methods in three phases of evaluation, including pre-, during- and post-implementation. To ensure contextual appropriateness the SPICES project will organize a multi-component community-engagement intervention implementation. For the qualitative component, the pre-implementation phase will involve a contextual assessment and stakeholder mapping, exploring potentials for CVD risk profiling strategies and led by trained Community Health Volunteers (CHV) to identify accessibility and acceptability. The during-implementation phase will involve healthy lifestyle counselling provided by CHVs and evaluation of the outcome to identify fidelity and scalability. The post-implementation phase will involve developing sustainable community-based strategies for CVD risk reduction. All three components will include a process evaluation. The theory of the socio-ecological framework will be applied to analyse the community engagement approach.

A stepped wedge quantitative evaluation of the roll out will focus on implementation outcomes such as uptake and engagement and changes in risk profiles. The quantitative component includes pre and post-intervention surveys.

The research project will ultimately develop a sustainable community engagement-based strategy for the primary prevention of CVD, to support or enhance the performance of NHS health care.

For peer review only - http://bmjopen.bmj.com/site/about/guidelines.xhtml

Key words: Implementation research, Cardiovascular disorders prevention, community engagement.

Introduction:

Cardiovascular disorders (CVD) are the single greatest cause of mortality worldwide each year, estimated to contribute to 31% of all deaths globally (1). Tackling CVD is an international priority and there have been many global initiatives such as the "Global Hearts" programme, a package launched by the World Health Organisation (WHO) and partners, to enhance the prevention and control of CVD. Some risk factors for CVD are non-modifiable, such as age, ethnicity and family history (2). Some other risk factors for CVD are modifiable, such as smoking, a lack of physical activity, being overweight, lower consumption of fruit and vegetables, high blood pressure, diabetes and high cholesterol (2). These risk factors can be changed through lifestyle or behavioural modifications. There is evidence of a social gradient in the prevalence of CVD, which points to associations between social and financial deprivation, vulnerability and risk factors for CVD. (3).

In 2015, CVD was the leading cause of mortality in the context of all chronic diseases, accounting for 27% and 25% of deaths in men and women respectively, in the UK(2). Coronary heart disease (CHD) and stroke were the main CVDs responsible for this mortality of men and women across all ages. As per British Heart Foundation report in 2017 CVD has a huge financial burden with annual associated healthcare costs estimated to be £9 billion annually in the UK (2). The UK has a standardised CVD death rate of 265.1 per 100,000 (2).

In the UK, the National Health Service (NHS) has launched the Health Check initiative aimed to prevent CVD. It is a national risk assessment and management program, free to adults aged 40 to 74 living in England, who do not currently have any vascular disorders and are not being treated for certain risk factors such as diabetes (4). It aims to assess the 10-year risk of CV events and disorders. Risk is assessed using QRISK2 (5), a tool which involves collection of the following information: age, gender, ethnicity, smoking status, family history of CHD, body mass index (BMI), cholesterol test, systolic and diastolic blood pressure, levels of physical activity, and alcohol consumption. Attendees receive a low (<10 % chance of event in 10 years), medium (>10 % but <20 %), or high (>20 %) 10-year cardiovascular (QRISK2) score. Above the 10% cut-off, attendees are offered a discussion with a qualified person, such as a nurse, about lifestyle and motivation to change, which may include goal setting and plans for follow up. Patients may also be offered medication for cholesterol and blood pressure. The NHS Health Check is recommended to be undertaken every five years.

Modelling predicted that the NHS Health Check could prevent 1,600 heart attacks and strokes each year if implemented as intended (6). Whilst evidence suggests that the Health Check programme has the potential to reduce CVD events and has therefore been rolled out nationally across the UK, its implementation has been poor, especially in some of the most disadvantaged groups at highest risk of developing CVD. In 2014, Public Health England (PHE) issued a call for action to increase the uptake rate of NHS Health Checks to 75% (7) and to increase awareness of risk and engagement with existing resources. Yet, as of 2017, current uptake remains far from this target with current predictions suggesting only 40% of the eligible population will receive one (8), due to the fact that uptake is low (48%) even when Health Checks are offered. (8) (9)

Data from some regions with very large ethnic minority community and socio-economically challenged populations showed that only 45% of patients who were invited for the check attended and subsequently received some form of counselling when they needed it. Authors have discussed how higher uptake in deprived communities would reduce the possibility of exacerbation of inequalities (10). Difficulty with accessing general practices, especially among socially vulnerable groups, has been highlighted as a common barrier to

For peer review only - http://bmjopen.bmj.com/site/about/guidelines.xhtml

attendance at Health Checks (11). A community-based engagement approach, which takes the CVD risking profiling and affiliated advice processes outside of the formal healthcare facility setting, has the potential to improve access to Health Checks and could be an effective and scalable way for improving the implementation and uptake of Health Checks. Community engagement (CE) has been conceptualised as "the process of working collaboratively with and through groups of people affiliated by geographic proximity, special interest, or similar situations, to address issues affecting the well-being of those people" (12). A review of community engagement interventions found them to be effective in improving health behaviours (such as physical activity), health consequences and psychological outcomes (i.e. self-efficacy and perceived social support) (13). Community-based intervention programmes have been implemented to increase the uptake of cancer screening programmes. The programmes have been found to be effective in increasing outcomes such as recognition, receipt and maintenance of screening behaviours (14). The CE approach offers the opportunity for task-shifting and owning the programme, whereby trained non-healthcare-professionals can perform CVD risk profiling assessments to individuals who might not otherwise be captured by the formal care pathway.

There is evidence that CVD risk assessments can be successfully delivered by Community Health Workers (CHWs), outside or inside the healthcare system. An observational study conducted in Bangladesh, Guatemala, Mexico and South Africa has demonstrated that CHWs who are inhabitants of their local communities and were fluent in the community's predominant language, can perform community-based screenings to predict CVD risk as effectively as physicians and nurses when using the non-laboratory-based Gaziano CVD risk scoring tool (15). CHWs were trained for 1-2 weeks, and results showed a 96.8% agreement between risk scores assigned by CHWs and healthcare professionals. However, a question remains whether the model taken in the global South could be transferrable to the global North, but it is at least plausible that a community-based engagement approach will be effective for increasing the uptake of CVD risk assessment, particularly in disadvantaged communities of the global North. There are examples in the global North on community engagement in health (16), and indeed the voluntary or 'third sector' have been considered key partners in the delivery of health promotion initiatives in the community (17).

Authors have argued that because of the current economic constraints with the formal healthcare system, the focus should be upon supplementing a service delivery model with an alternative community development model (18). The key aspect is supplementing formal service delivery by utilizing communities' 'social capital'. The term 'social capital' describes the various resources that people may have through their relationships in families, communities and other social networks. Social capital bonds people together and helps them make links beyond their immediate friends and neighbours (19).

For this compassionate community approach to work, contextual appropriateness and cultural sensitivity of an intervention is crucial (20). Following this argument, the SPICES project in two areas of England, East Sussex and Nottingham, will co-produce a multi-component community-engagement intervention focussed on delivering a Health Check-style CVD risk screening, with appropriate health coaching and follow-up, in a community setting (21) and delivered by community volunteers. The intervention will be trialled and evaluated using a mixed methods approach using both qualitative and quantitative methods. The specific objectives of the project are:

To evaluate with stakeholders the potential for a community engagement-based CVD primary prevention programme to support or enhance the NHS Health Check Programme.

To co-produce with the communities an evidence-informed community-engagement intervention on CVD risk, based on the NHS Health Check model, tailored to the context in disadvantaged communities in East Sussex and Nottingham.

For peer review only - http://bmjopen.bmj.com/site/about/guidelines.xhtml

To implement the intervention in the local communities where it was co-produced, and:
-assess its effectiveness versus routine care.
-assess the fidelity, feasibility, acceptability, uptake and scalability of the implementation.
-carry out a process evaluation of the intervention and its implementation

This project is part of the SPICES (Scaling-up Packages of Interventions for Cardiovascular disease prevention in selected sites in Europe and Sub-Saharan Africa) project (22). This is a Horizon 2020 project financed by the European Commission that aims to address the CVD burden. The overall objective is to implement and evaluate a comprehensive cardiovascular disease (CVD) prevention and care program at the community level in five countries (Belgium, France, Uganda, UK, South Africa), to identify and compare barriers and facilitators for implementation across study contexts and to develop a learning community.

Methods:

Theoretical Model

SPICES is underpinned by the Consolidated Framework for Advancing Implementation Research (23), and Reach, Effectiveness, Adoption, Implementation, and Maintenance (sustainability) framework /RE-AIM models (24). We also recognize as a global health project the need for the use of the socio-ecological framework (25). As mentioned above, this model allows an understanding of the multifaceted and interactive effects of personal, social and environmental factors that determine behaviour; and for identifying behavioural and organisational leverage points and intermediaries for health promotion within organisations and communities.

Study Design

A mixed-methods research methodology will be applied strategically combining qualitative and quantitative methods at both sites. This approach will allow us to model the iterative nature of coproduction and implementation research without compromising the rigour of the study (26; 27). The study will take place in three phases:
- Pre-intervention; when stakeholder mapping and local adaptation will be carried out
- Intervention roll out, recruitment and evaluation
- Post-intervention evaluations and feedback (28)- Process evaluation will be conducted in all three phases.

Stage 1: To explore the implementation context and co-produce the intervention.

To explore the context where the implementation will take place we will carry out several mappings. These will give us the context for recruitment and implementation co-design.
They are as follows:
(a) Mapping the potential stakeholders: Mapping of the stakeholders will be done to find out who are the key stakeholders, where they come from, and what they are looking for in relationship to the study objectives(29). To engage the community, it is essential to map the community stakeholders (civil society organisations) as they are the gatekeepers of the community. Three levels of stakeholder mapping will be carried out, namely at macro, meso and micro levels.
Macro-level: stakeholders will be identified via the existing link of PI of the project in the community through meetings with local public health or other relevant departments and CSOs and using online information. Interviews with this category of stakeholders will provide insights into implementation sustainability.
Meso-level: a strategic community volunteer organisation mapping will be carried out to find out the relevant organisations, through which individual volunteers will be selected. This will

For peer review only - http://bmjopen.bmj.com/site/about/guidelines.xhtml

be done in three ways; using online searches, personal contacts and snowballing. In-depth interviews will be conducted to co-design a sustainable intervention implementation.
Micro-level: an exploration will be done with volunteers and end-user groups to co-design an acceptable and feasible intervention implementation.
(b) Mapping the context: social mapping will be carried out to explore the lifestyle context of the community via observations.
(c) Training of volunteers by professional health trainers and researchers following current NICE Public health guideline [PH6] 'Behaviour change: general approaches' (30)
(d) CVD risk profiling by trained community health volunteers (CHV).
CHVs will be the persons who have been involved in health-related volunteering for example volunteers who worked in cancer prevention, health check, healthy lifestyle etc programme. They will be involved in the screening of the CVD risk population and implement the designed intervention.

Expected Intervention

The final elements of the intervention will be co-produced within each community setting, following the mapping exercises outlined above. As outlined in the CFAIR (23), interventions are usually composed of a core component which is essential and indispensable, and an adaptable periphery, which can and should be tailored to the specific setting and users.

Core Components: Following identification of moderate to high risk for CVD, the intervention will consist of non-clinical (non-NHS) individual or group support sessions within the community, focus on motivating behaviour change. Each participant will be supported by trained SPICES researchers or community health workers to identify behaviour change goals, produce action plans to achieve them, and problem solve in cases of unexpected outcomes. All SPICES Interventions are theoretically grounded in the theory of behaviour change and deploy the strongest evidenced Behaviour Change Techniques (BCTs) from the literature.

1. Goal Setting
2. Action Planning
3. Problem Solving
4. Motivational Interviewing
5. Feedback on progress towards goals
6. Feedback on the health impact

The use of these six BCTs are focussed in SPICES on five Target Behaviours:

1. Reduce/cease smoking
2. Increase moderate physical activity
3. Reduce fat, salt, the sugar content of the diet
4. Increase fibre, oily fish, fruit and vegetable content of the diet
5. Reduce sedentary hours

Community Adaptation: The exact elements of the support sessions will be tailored to individuals and their community context, will be determined during iterative co-design with community representatives, and will be drawn from the following (31; 32):

Step-I - Goal setting

Every participant should receive specific healthy lifestyle counselling/feedback based on their individual item InterHE ART assessment scores (the moderate group). The feedback will be based on a review of international guidelines conducted as formative work for the SPICES project intervention (33). SPICES behaviour change support sessions will be based on the best-evidenced approaches to healthy lifestyle modification and community context and preferences.

For peer review only - http://bmjopen.bmj.com/site/about/guidelines.xhtml

Two further screening questionnaires may be used with individuals to assess the benefit of possibly behaviour change;

- International Physical Activity Questionnaire (IPAQ, see appendix) is an internationally validated instrument to capture information about weekly physical activity habits, behaviours and routines.
- The Dietary Approaches to Stop Hypertension Questionnaire DASH-Q is a self-reporting lifestyle questionnaire (see appendix) to capture information about weekly dietary habits, routines and behaviours, based around ‘Dietary Approach to Stopping Hypertension’ (34).
- Current behaviours audit: Using food and physical activity diaries prepared by and provided to participants by the SPICES research team, participants will be encouraged to complete an audit of one week of current dietary and physical activity behaviours, habits and routines to establish a baseline from which goals for change and improvement can be set in negotiation with SPICES CHVs
- The ABCD self-reporting questionnaire (see appendix) to assess participant perception of personal heart health risk.
- The EQ-5D-5L internationally validated Quality of Life self-reporting questionnaire (see appendix).

Step-II - Action Planning by the participants

Participants will be asked to create an action plan with appropriate goal setting for two behaviours (diet and exercise habits) in relation to when, where and how they will undertake, for example, physical activity (based on the item stems used by Luszczynska & Schwarzer (35); when the physical activity will be performed, where it will be performed, how often it will be performed. The way goals are reached and plans recorded will be co-designed with key stakeholders.

Step III - Problem-solving

CHVs will help participants to analyse any factors which may influence their ability to achieve the goals and to generate strategies which could help them overcome these barriers.

CHVs will use Motivational Interviewing techniques about health, social and environmental, and emotional barriers and consequences. Culturally and context-sensitive information will be provided (both verbally and in the form of leaflets) about the importance of eating healthily, being physically active, and not smoking for positive outcomes on physical and mental health.

Trial of Intervention

This will be an open-label, non-controlled trial, examining fidelity, feasibility, acceptability, uptake and scalability of the intervention.

Eligible Population

Economically disadvantaged, lower socio-economic status (SES) postcodes, will be identified using the overall Index of Multiple Deprivation (36a); Participants’ SES will be determined by their postcode of residence. Any resident aged 18 or above living in the study postcode areas will be eligible to take part in the baseline assessment for the study.

Study Sample Size

The sample size calculation for the quantitative study used statistical modelling for a stepped wedge design, randomising community centres over time with the InterRHEART score as the outcome (90% power for 5% significance, effect size (Cohen’s D)=0.25, intracluster correlation coefficient of 0.05, control clusters crossing to intervention in 4 steps, participant autocorrelation=0.7 and cluster autocorrelation=0.9), which requires a total of at least 144 persons. This needs approximately 200-300 people across the two sites as we expect a high level of attrition (as much as 50%). At least 1500 community members will need to be screened to achieve this recruitment (37).

For peer review only - http://bmjopen.bmj.com/site/about/guidelines.xhtml

Recruitment of Community Health Volunteers and Trial Participants
Community Health Volunteers (CHVs) will be recruited to perform CVD risk profiling assessments through a combination of 'doorstep outreach' and 'intermediary organisation recruitment' approaches in East Sussex and through existing community and neighbourhood groups with the assistance of partners such as Self-Help UK, the Renewal Trust, Nottingham CVS and others in Nottingham.
For recruitment of trial participants, we will use similar community networks, and endeavour to use quota sampling, in that we will seek to ensure the inclusion of high, low and median income neighbourhood residents, citizens from the South Asian and African diasporas; and will encourage participants to refer others to the researchers who may be able to potentially contribute or participate in the study.

Baseline Screening of CVD Risk
Participants will fill in the validated InterHEART score to determine suitability for the trial. The non-laboratory-based InterHEART scoring tool requires minimal resources which is practical for use within the community. There is also evidence to suggest that the InterHEART can reliably predict the incidence of CVD and death in low, middle, and high-income countries for a mean follow-up of 4.1 years (38). Risk is expressed as a score from the InterHEART: 0-9 (Low risk), 10-15 (moderate risk), and 16-48 (high risk). The InterHEART scoring tool will be translated onto a mHealth platform so that the trained CHVs can easily administer them during community engagement and contact, and online data will directly reach the University repository in real time from the respondents' device.

Participants who score moderate or high risk in the baseline assessment will be invited to participate in the intervention. The moderate risk (amber) score population will be selected for participation in the intervention (=score of 10 or higher), and will fill out the self-completion survey InterHEART scoring every three months. The InterHEART scoring tool will be translated onto a mHealth platform so that the trained CHVs can easily administer them during community engagement and contact, and online data will directly reach the University repository in real time from the respondents' device (39).

Clinical Outcome and Follow-Up
The primary outcome will be the change in the risk score among people who complete the community delivered CVD risk assessment and coaching. Secondary outcomes will be gathered from participants identified as 'high risk'. Numbers of participants who a) self-referred (defined as having contacted their GP surgery requesting for a formal check-up) and b) completed the NHS Health Checks
Data collected during the trial of intervention will comprise:

- Self-reported lifestyle (modifiable and non-modifiable) risk factors gathered through survey instruments and interviews.
- Observed/measured data on all participants' age, gender, ethnicity, postcode, hip to waist ratio, gathered by trained volunteers.
- Quantitative analysis of changes in behavioural intention, target behaviours, and measurable CVD risk.

Outcomes will be assessed at three months post-intervention.

Post-intervention Qualitative Evaluation and Feedback
In the post-intervention phase, a qualitative evaluation will be carried out during which

The following implementation parameters will be assessed:

For peer review only - http://bmjopen.bmj.com/site/about/guidelines.xhtml

1. The impact on awareness of CVD risks and mitigating measures, amongst disadvantaged populations of a community-based, non-clinical, CVD risk scoring tool and education.
2. The impact of the community based non-clinical CVD risk scoring tool and education on motivational healthy lifestyle among disadvantaged populations.
3. The facilitators and barriers to the adoption of a community-based CVD prevention implementation programme, by target populations.
4. The perspectives of participants regarding their experience and meaning of the intervention.

These will be explored with a subset of intervention participants using focus groups or/and in-depth interview and community mapping. Participants for the qualitative component will include adult volunteers, public health stakeholders and people within the community. The community volunteers will be selected via community organisations and public health stakeholders will be selected from the same area of the research site. Community participants for the qualitative component will be selected via the community volunteers. This post-intervention qualitative study will include randomly selected trial participants.

We will be flexible in terms of the number of participants for the qualitative component. The number will be determined through the principle of saturation and diversity. However, from each site, we will aim to include at least 12 respondents and a maximum of 30 respondents from different categories (40; 41).

Process evaluation of the intervention

To assess the fidelity of the conclusions concerning the project's effectiveness, ongoing assessment, monitoring, and enhancement is important. If significant results are found, but fidelity was not assessed, it cannot be determined if the effectiveness is attributable to unintentionally added or omitted components. Bellg and colleagues (42) propose that considerations of fidelity should permeate all stages of the study: design of the study, provision of training, delivery of the intervention, receipt of the intervention, and re-enactment of skills. As a result, we will carry out a process evaluation of the project. This will be done through Process Documentation of all the stages of this project including community volunteers mapping, Healthy lifestyle counselling, action planning and problem-solving.

Thirsk and Clark (43) argue how health-care interventions need to be understood in ways that are responsive to the complexities and intricacies of programs, people and places. They emphasise the understanding of the comprehensive experience of the persons who are delivering and receiving the intervention. Process Evaluation is a tool that can capture the intervention experience. We will be following the model designed by Moore et al (44):

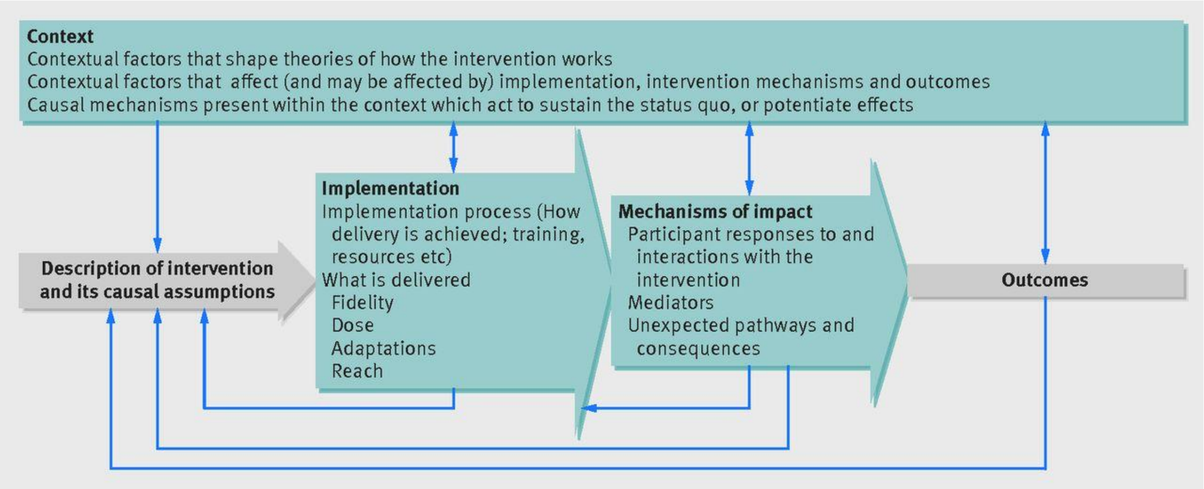

For peer review only - http://bmjopen.bmj.com/site/about/guidelines.xhtml

Data Analysis:

Quantitative data will be analysed using Stata version 15 or later. Descriptive statistics will summarise outcomes before and after clusters cross over to the intervention (45. Normally distributed variables will be summarised by means and standard deviations, skewed continuous variables by medians and interquartile ranges, categorical variables by frequencies and percentages. We will estimate the treatment effect using a cross-classified linear mixed effects model. A statistical analysis plan will be agreed and signed off prior to final analysis commencing. Thematic analysis of qualitative data will be carried out using a constant comparison method of analysis, which will gather and generate ideas and categories through inductive processes. The computer package NVivo will be used for primary analysis (46). Memo writing will be carried out to describe details of the interview setting and interaction of respondent and interviewer that may not be captured in audio transcriptions. This thematic analysis has deductive and inductive elements, lending itself to multidisciplinary health research (47). The analysis framework will incorporate the key theoretical constructs and respond to the context of policy and practice to include a range of deductive themes. Further themes will be induced from the interview data.

An appropriate balance of integration between empirical data and interpretation will be ensured. The investigators will extract the meaning of the empirical data and interpret them whilst acknowledging the complexity of the phenomena of CVD risk reduction in the context of community engagement (48). This method holds links to the original data and the output allows comprehensive and transparent data analysis.

Conclusion:

Given that despite the rolling out of the NHS Health Checks programme over and above current care across the UK has not been implemented as well as it could have been, especially in some of the most disadvantaged groups prone to developing CVD, the project aims to scale-up packages of interventions for cardiovascular prevention particularly to these vulnerable populations. This interdisciplinary project includes public health, social and behavioural science approaches. The main focus aspect of this project is the deinstitutionalization of health care by operating outside of formal healthcare settings. The project will emphasise on the power of citizens, combining their efforts to generate cultures of care which complement or even compensate for the inadequacies of formal systems thus sustainable. The research project will ultimately develop a community engagement-based CVD primary prevention programme to support or enhance the performance of the NHS health care.

Funding statement:

This protocol is a contextual plan for the SPICES project in the UK. The SPICES project received funding from the European Commission through the Horizon 2020 Research and Innovation Action Grant Agreement No 733356 to implement and evaluate a comprehensive CVD prevention programme in five settings: a rural & semi-urban community in a low-income country (Uganda), middle income (South Africa) and vulnerable groups in three high-income countries (Belgium, France and United Kingdom). The funder had no role in the design, decision to publish, or preparation of the manuscript.

Availability of data and materials:

A protocol should not contain any data; it sets out the research questions and how they will be addressed.

For peer review only - http://bmjopen.bmj.com/site/about/guidelines.xhtml

Ethics approval and consent to participate:
This protocol has received two ethics approval from the University of Sussex, The **BSMS** Research Governance and Ethics Committee (RGEC (ER/BSMS9E3G/1)), and from Nottingham Trent University (no. TBA). All participants will be requested to consent before enrolment into the study. All participant information will be kept confidential and accessible only to the key investigative team. All published data will be anonymised and can be accessed based on a written request to the Principal Investigator.

Competing interests:
Authors declare that they have no competing interests.

Authors' contributions:
PN has written the first draft and received feedback from HvM and SA on it. PN prepared the second draft and it received feedback from LG. The third draft received feedback from all the authors. All authors read and approved the final contextual protocol (4th version).

References:

1. World Health Organisation; WHO. *Global Health Observatory (GHO) data*. 2017. Retrieved on 18th May 2019, retrieved from https://www.who.int/gho/mortality_burden_disease/causes_death/top_10/en

2. British Heart Foundation report BHF. https://www.bhf.org.uk/informationsupport/publications/policy-documents/annual-report-2017.

3. Amuzu A, Carson C, Watt HC, Lawlor DA, Ebrahim S. Influence of area and individual life course deprivation on health behaviours: findings from the British Women's Heart and Health Study. Eur J Cardiovasc Prev Rehabil. 2009;16(2): 169-73. doi: 10.1097/HJR.0b013e328325d64d.

4.Public Health England (2013). NHS Health Check implementation review and action plan Summary, https://assets.publishing.service.gov.uk/government/uploads/system/uploads/attachment_data/file/224536/NHS_Health_Check_implementation_review_and_action_plan_summary_web.pdf 5.

5. QRISK2. 2017. https://qrisk.org/2017/

6. Public Health, England. NHS Health Checks 'prevented thousands of heart attacks'. 2016. https://www.nhs.uk/news/heart-and-lungs/nhs-health-checks-prevented-thousands-of-heart-attacks/

7. Public Health England. Factsheet: Implementation of the NHS Health Check programme. 2014. https://www.england.nhs.uk/wp-content/uploads/2014/02/pm-fs-3-1.pdf

8. Waterall Jamie. PHE. Getting Serious About CVD Prevention. Reducing Variation & Optimising Care. 2018. https://www.healthcheck.nhs.uk/seecmsfile/?id=195

For peer review only - http://bmjopen.bmj.com/site/about/guidelines.xhtml

9. Dalton AR, Bottle A, Okoro C, Majeed A, Millett C. Uptake of the NHS Health Checks programme in a deprived, culturally diverse setting: cross-sectional study. J Public Health (Oxf). 2011; 33(3):422-9. doi: 10.1093/pubmed/fdr034.

10. Robson J, Dostal I, Sheikh A, et al. The NHS Health Check in England: an evaluation of the first 4 years. 2015. BMJ Open. 2015;6: e008840. doi:10.1136/ bmjopen-2015

11. Harte Emma, Calum MacLure, Adam Martin, Catherine L Saunders, Catherine Meads, Fiona M Walter, Simon J Griffin, Jonathan Mant and Juliet A Usher-Smith (2018), Reasons why people do not attend NHS Health Checks: a systematic review and qualitative synthesis, British Journal of General Practice. 2018;68 (666): e28-e35. DOI: https://doi.org/10.3399/bjgp17X693929

12. CTSA. Principles of Community Engagement, Second Edition, NIH Publication No. 11-7782. 2011. https://.atsdr.cdc.gov/communityengagement/pdf/PCE_Report_508_FINAL.pdf

13. O'Mara-Eves A., G Brunton, D McDaid, S Oliver, J Kavanagh, F Jamal, T Matosevic, A Harden, and J Thomas. Community engagement to reduce inequalities in health: a systematic review, meta-analysis and economic analysis, Public Health Research. 2013;1:4

14. Bird JA; McPhee SJ, Ha NT, Le B, Davis T, Jenkins CN. Opening pathways to cancer screening for Vietnamese-American women: lay health workers hold a key. Prev Med. 1998;27(6):821-9.

15. Gaziano Thomas A, Shafi ka Abrahams-Gessel, Catalina A Denman, Carlos Mendoza Montano, Masuma Khanam, Thandi Puoane, Naomi S Levitt (2015). An assessment of community health workers' ability to screen for cardiovascular disease risk with a simple, non invasive risk assessment instrument in Bangladesh, Guatemala, Mexico, and South Africa: an observational study. Lancet Glob Health. 2015; 3:e556–63. http://dx.doi.org/10.1016/ S2214-109X(15)00142-4

16. Wood, J. The University as a Public Good: Active citizenship and university community engagement. International Journal of Progressive Education. 2012; 8 (3), 15-31. Retrieved from http://dergipark.org.tr/ijpe/issue/26312/277299

17. Kings Fund. 2011. Retrieved from, https://www.kingsfund.org.uk/sites/default/files/Voluntary-and-community-sector-in-health-implications-NHS-reforms-The-Kings-Fund-june-2011_0.pdf

18. Abel, J., Bowra, J., Walter, T. et al. Compassionate Community networks: Supporting home dying. BMJ Supportive and Palliative Care. 2011;1,129-133. http://dx.doi.org/10.1136/bmjspcare-2011-000068

19. Baum FE and Ziersch AM. Social capital. Journal of Epidemiology & Community Health. 2003; 57: 320-323.

20. Sallnow L. Libby, Heather Richardson, Scott Murray, Allan Kellehear. Understanding the impact of a new public health approach to end of life care: a qualitative study of a community-

For peer review only - http://bmjopen.bmj.com/site/about/guidelines.xhtml

led intervention. The Lancet. 2017; 389: Special issue 788. DOI:https://doi.org/10.1016/S0140-6736(17)30484-1

21. Boyd, Stephen McKernon, Bernie Mullin, Andrew Old. Improving healthcare through the use of co-design. The New Zeeland Medical Journal. 2012; 125:(1357).

22. Musinguzi Geofrey, Rhoda K. Wanyenze1, Rawlance Ndejjo, Isaac Ssinabulya, Harm van Marwijk, Isaac Ddumba, Hilde Bastiaens and Fred Nuwaha. An implementation science study to enhance cardiovascular disease prevention in Mukono and Buikwe districts in Uganda: a stepped-wedge design. Health Service research. 2019; 19:253 doi.org/10.1186/s12913-019-4095-0

23. Damschroder Laura J, David C Aron, Rosalind E Keith, Susan R Kirsh, Jeffery A Alexander and Julie C Lowery. Fostering implementation of health services research findings into practice: a Consolidated Framework for Advancing Implementation Science. Implementation Science. 2009; 4:50 doi:10.1186/1748-5908-4-50

24. Glasgow RE, McKay HG, Piette JD, Reynolds KD: The RE-AIM framework for evaluating interventions: what can it tell us about approaches to chronic illness management? Patient Educ Couns. 2001;44:119-127.

25. Theobald, S., Brandes, N., Gyapong, M., et al. Implementation research: new imperatives and opportunities in global health, Lancet. 2018;392: 2214–28

26. Peters DH, Adam T, Alonge O, et al. Implementation research: what it is and how to do it. BMJ. 2013; 347: f6753.

27. Brown CH, Curran G, Palinkas LA, et al. (2017). An overview of research and evaluation designs for dissemination and implementation. Annual Review of Public Health. 2017; 38:1–22

28. Greene, J. C. Mixed Methods in Social Inquiry. 2007. Jossey-Bass. San Francisco.

29. BSR. Stakeholder Mapping (2011). www.bsr.org/reports/BSR_Stakeholder_Engagement_Stakeholder_Mapping.final.pdf

30. NICE Public health guideline [PH6] 'Behaviour change: general approaches' (https://www.nice.org.uk/guidance/ph6/resources/behaviour-change-general-approaches-pdf-55457515717).

31. Michie Susan, Michelle Richardson, Marie Johnston, et al. (2013). The Behaviour Change Technique Taxonomy (v1) of 93 Hierarchically Clustered Techniques: Building an International Consensus for the Reporting of Behavior Change Interventions. Annals of Behavioural Medicine. 2013. 46;(1):81–95, https://doi.org/10.1007/s12160-013 9486-6

32. Michie, Susan, Maartje M. Van Stralen, and Robert West. "The Behaviour Change Wheel: A New Method for Characterising and Designing Behaviour Change Interventions. Implementation Science. 2011;6,1:42.

For peer review only - http://bmjopen.bmj.com/site/about/guidelines.xhtml

33. Yusuf Salim, Steven Hawken, Stephanie Ôunpuu, et al., on behalf of the INTERHEART Study Investigators. Effect of potentially modifiable risk factors associated with myocardial infarction in 52 countries (the INTERHEART study): a case-control study. Lancet. 2004; 364: 937–52

34. Sacks Frank M., Laura P. Svetkey, M.D., William M. Vollmer et al. Effects on Blood Pressure of Reduced Dietary Sodium and the Dietary Approaches to Stop Hypertension (DASH) Diet.
N Engl J Med. 2001; 344:3-10, DOI:10.1056/NEJM200101043440101

35. Luszczynska Aleksandra, Urte Scholz & Ralf Schwarzer. The General Self-Efficacy Scale: Multicultural Validation Studies, The Journal of Psychology. 2005; 139:5, 439-457, doi:10.3200/JRLP.139.5.439-457

36.. Kontopantelis E, Mamas MA, Van Marwijk H, Ryan AM, Buchan IE, Ashcroft DM, et al. Geographical epidemiology of health and overall deprivation in England, its changes and persistence from 2004 to 2015: A longitudinal spatial population study. 2018; 72 (2):140–7.

37. Hooper R, Teerenstra S, de Hoop E & Eldridge S. Sample size calculation for the stepped wedge and other longitudinal cluster randomised trials. Statist. Med. 2016; 35 4718–4728

38. Yusuf Salim, Sumathy Rangarajan, Koon Teo, Shofiqul Islam, et al. Cardiovascular. Risk and Events in 17 Low-, Middle-, and High-Income Countries. N Engl J Med. 2014; 371:818-827, DOI: 10.1056/NEJMoa1311890

39. Williams Natalie Joseph, Amy Lloyd, Adrian Edwards, Lynne Stobbart, David Tomson, Sheila Macphail, Carole Dodd, Kate Brain, Glyn Elwyn, Richard Thomson. Implementing shared decision making in the NHS: lessons from the MAGIC programme. BMJ. 2017a;357:j1744 doi: https://doi.org/10.1136/bmj.j1744

40. Guest, G., Bunce, A., & Johnson, L. How Many Interviews Are Enough?: An Experiment with Data Saturation and Variability. Field Methods. 2006; 18 (1): 59–82. https://doi.org/10.1177/1525822X05279903

41. Malterud K, Siersma VD, Guassora AD. Sample Size in Qualitative Interview Studies: Guided by Information Power. Qual Health Res. 2015; 1–8.

42. Bellg, A. J., Borrelli, B., Resnick, B., Hecht, J., Minicucci, D. S., Ory, M., et al. Treatment Fidelity Workgroup of the NIH Behavior Change Consortium. (2004). Enhancing Treatment Fidelity in Health Behavior Change Studies: Best Practices and Recommendations From the NIH Behavior Change Consortium. Health Psychology. 2004; 23(5):443-451. http://dx.doi.org/10.1037/0278 6133.23.5.443

43. Thirsk Lorraine M. and Alexander M. Clark. Using Qualitative Research for Complex Interventions: The Contributions of Hermeneutics. International journal of qualitative methods. 2017; 16:1-10. doi.org/10.1177/1609406917721068

For peer review only

44. Moore Laurence, Alicia O'Cathain, Tannaze Tinati, Daniel Wight, Janis Baird (2015). Process evaluation of complex interventions: Medical Research Council guidance. The BMJ. 2015;350:h1258. doi: 10.1136/bmj.h1258

45. StataCorp. 2017. *Stata Statistical Software: Release 15*. College Station, TX: StataCorp LLC

46. Ritchie Jane, Jane Lewis, Carol McNaughton Nicholls, Rachel Ormston. Qualitative Research Practice: A Guide for Social Science Students and Researcher; 2013. Sage Publications.

47. Gale NK, Heath G, Cameron E, Rashid S, Redwood S. Using the framework method for the analysis of qualitative data in multi-disciplinary health research. BMC Med Res Methodol. 2013;(13):117. doi.org/10.1186/1471-2288-13-117

48. Green J, Thorogood N. Qualitative methods for health research. 2005. London: Sage Publications Ltd.

Authors Information:

1. Papreen Nahar. Department of Primary Care and Public Health, Brighton and Sussex Medical School. University of Sussex, UK.
1. Harm van Marwijk. Department of Primary Care and Public Health, Brighton and Sussex Medical School. The University of Sussex. UK
2. Linda Gibson: School of Social Sciences. Nottingham Trent University, UK
3. Musinguzi Geofrey. Department of Disease Control and Environmental Health, School of Public Health, College of Health Sciences. Makerere University, Uganda
4. Sibyl Anthierens. Department of Primary and Interdisciplinary Care, University of Antwerp, Belgium
1. Elizabeth Ford. Department of Primary Care and Public Health Brighton and Sussex Medical School. University of Sussex, UK
1. Stephen A Bremner. Department of Primary Care and Public Health Brighton and Sussex Medical School. University of Sussex, UK
2. Mark Bower. School of Social Sciences, Nottingham Trent University, UK
5. JY Reste. Faculté de médecine et des sciences de la santé, Université de Bretagne Occidentale, Brest, France
6. Sodi Tholene. Department of Psychology. University of Limpopo, South Africa
4. Hilde Bastiaens. Department of Primary and Interdisciplinary care. University of Antwerp, Belgium

For peer review only

Appendix 3

ABCD subscale and selected INTERHEART variable correlation values from Nottingham study compared with values reported in the original Woringer study.

		Knowledge	Perceived Risk	Perceived Benefit	Healthy Intentions	IMD2010 Quintile	BMI/W2Hr	Qrisk2/ INTERHEART
Knowledge	Correlation Coefficient		-.124/**.013**	-.148/**-.021**	-.106/**-.039**	-.002/ .085	-.225/ -.084	-.007/ -.018
	Sig 2 tailed		.236/ .722	.175/ .645	.319/ .400	.986/ .066	.021/ .082	.941/ .714
	N		93/462	86/462	91/462	99/466	105/433	104/436
Perceived Risk	Correlation Coefficient			-.195/ -.112	-.188/ -0.36	.239/ .039	.389/ .182	.220/ .356
	Sig 2 tailed			.080/ .016	.088/ .441	.025/ .397	.000/ .000	.036/ .000
	N			82/462	84/462	87/466	92/433	91/436
Perceived Benefits	Correlation Coefficient				.533/ .383	-.287/ .071	-.068/ .000	-.118/ -.164
	Sig 2 tailed				.000/ .000	.009/ .127	.538/ .997	.284/ .001
	N				83/462	81/466	85/433	84/436
Healthy Intentions	Correlation Coefficient					-.261/ .098	.084/ .044	-.072/ -.079
	Sig 2 tailed					.016/ .034	.430/ .365	.504/ .100
	N					85/466	90/462	89/436

Correlations

Correlations

Correlations

			knowledge score	Risk score	Benefit score	Diet score	Smoke score	total_score
Spearman's rho	knowledge score	Correlation Coefficient	1.000	.118**	.103*	.078	-.079	.006
		Sig. (2-tailed)	.	.009	.023	.086	.082	.896
		N	483	483	483	483	483	440
	Risk score	Correlation Coefficient	.118**	1.000	-.003	.057	.107*	.371**
		Sig. (2-tailed)	.009	.	.950	.212	.019	.000
		N	483	483	483	483	483	440
	Benefit score	Correlation Coefficient	.103*	-.003	1.000	.538**	.009	-.236**
		Sig. (2-tailed)	.023	.950	.	.000	.851	.000
		N	483	483	483	483	483	440
	Diet score	Correlation Coefficient	.078	.057	.538**	1.000	-.022	-.143**
		Sig. (2-tailed)	.086	.212	.000	.	.635	.003
		N	483	483	483	483	483	440
	Smoke score	Correlation Coefficient	-.079	.107*	.009	-.022	1.000	.240**
		Sig. (2-tailed)	.082	.019	.851	.635	.	.000
		N	483	483	483	483	483	440
	total_score	Correlation Coefficient	.006	.371**	-.236**	-.143**	.240**	1.000
		Sig. (2-tailed)	.896	.000	.000	.003	.000	.
		N	440	440	440	440	440	440

** Correlation is significant at the 0.01 level (2-tailed).

* Correlation is significant at the 0.05 level (2-tailed).

For peer review only

Appendix 4. Figures and factor result tables

Without smoking items

Non-missing samples: 420

Bartlett's Test of Sphericity (X2 = 4235.007, p-value < 0.001)

The overall KMO is 0.82, which is within the recommended range (0.8 to 1).

EFA results

- The root mean square of the residuals (RMSR) is 0.05
- Tucker Lewis Index of factoring reliability = 0.77
- RMSEA index = 0.121 and the 90 % confidence intervals are 0.113 0.129
- BIC = 165.35

Scree plot

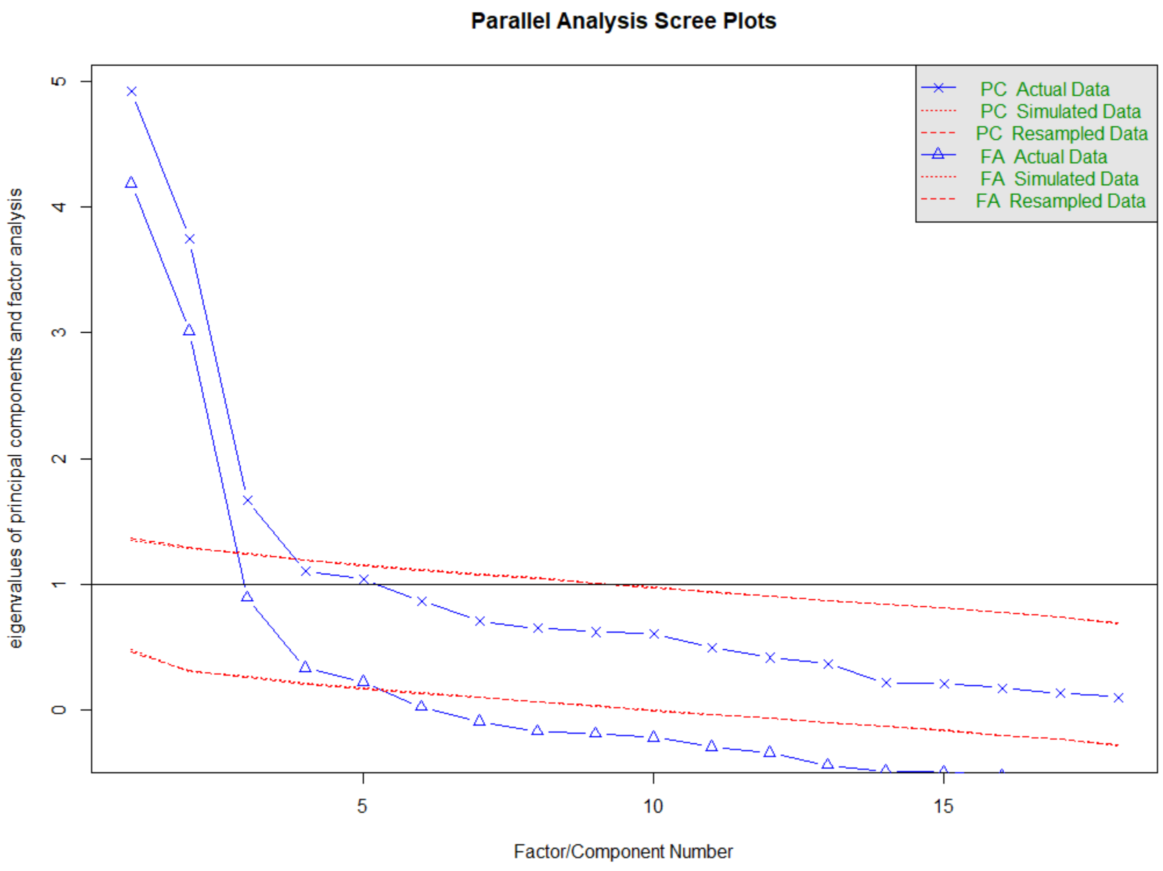

Figure 1. 18-item ABCD Questionnaire results (without smoking items)

Note: Scree plots are a line-plot of the eigenvalues of factors identified by the Principal Components Analysis (PCA) and Exploratory Factor Analysis (EFA). In this analysis, conducted using the independent Nottingham 'SPICES' study dataset, the blue lines indicate eigenvalues calculated for

each factor extracted from the observed data. Eigenvalues of 1 or greater are generally considered significant. The red lines represent eigenvalues generated by the PCA and EFA operations from a random data matrix of the same size as the original. Plotting both lines allow us to observe 1) the number of identified factors with eigenvalues exceeding 1, and 2) the point of inflection (the point at which the gap between resampled data and actual data tends to be minimum). The principle is to retain, at maximum, the number of factors with observed eigenvalues that are larger than those extracted from corresponding factors based on resampled/noise data.

Table A1 (a). Factor loadings of the exploratory factor analysis of the risk scale without the smoking items

Items	Factor2	Factor1	Factor3	communality	uniqueness
I feel I will suffer from a heart attack or stroke sometime during my life	0.86	0.02	-0.03	0.74	0.26
It is likely that I will suffer from a heart attack or stroke in the future	0.91	0.05	0.00	0.82	0.18
It is likely that I will have a heart attack or stroke sometime during my life	0.88	0.01	0.01	0.77	0.23
There is a good chance I will experience a heart attack or stroke in the next 10 years	0.73	-0.07	0.01	0.55	0.45
My chances of suffering from a heart attack or stroke in the next 10 years are great	0.65	-0.10	0.01	0.44	0.56
It is likely I will have a heart attack or stroke because of my past and/or present behaviors	0.56	-0.03	-0.01	0.32	0.68
I am not worried that I might have a heart attack or stroke (Reverse coded)	0.28	-0.11	0.10	0.10	0.90
I am concerned about the likelihood of having a heart attack or stroke in the near future	0.40	-0.02	0.11	0.16	0.84
I am thinking about exercising at least 2.5 hours a week	-0.02	0.87	-0.06	0.73	0.27
I intend or want to exercise at least 2.5 hours a week	-0.01	0.91	-0.04	0.80	0.20
When I exercise for at least 2.5 hours a week I am doing something good for the health of my heart	0.02	0.69	0.10	0.53	0.47
I am confident that I can maintain a healthy weight by exercising at least 2.5 hours a week	-0.05	0.45	0.19	0.31	0.69
I am not thinking about exercising for 2.5 hours a week (Reverse coded)	0.04	0.56	0.05	0.34	0.66
When I eat five portions of fruit and vegetables a day I am doing something good for the health of my heart	0.02	0.37	0.35	0.36	0.64
Increasing my exercise to at least 2.5 hours a week will decrease my chances of having a heart attack or stroke	0.02	0.39	0.27	0.30	0.70
I am confident that I can eat at least five portions of fruit and vegetables a day within the next two months	-0.04	0.07	0.64	0.46	0.54

For peer review only

I am thinking about eating at least five portions of fruit and vegetables a day	0.01	-0.01	0.93	0.85	0.15
I am not thinking about eating at least five portions of fruit and vegetables a day (Reverse coded)	-0.01	-0.03	0.78	0.60	0.40

Table A1 (b): Summary of factor loadings and variance distribution of the risk scale without the smoking items

Measures	Factor 2	Factor 1	Factor 3
SS loadings	3.86	3.04	2.28
Proportion Var	0.21	0.17	0.13
Cumulative Var	0.21	0.38	0.51
Proportion Explained	0.42	0.33	0.25
Cumulative Proportion	0.42	0.75	1.00

With smoking items

Non-missing samples: 88

The overall KMO is 0.78, which is slightly below the recommended range (0.8 to 1).

The Bartlet's test of Sphericity is significant (X2 = 1223.459, p-value < 0.001), indicating the sample adequacy for factor analysis.

EFA results

- The root mean square of the residuals (RMSR) is 0.06
- Tucker Lewis Index of factoring reliability = 0.69
- RMSEA index = 0.129 and the 90 % confidence intervals are 0.124 and 0.136
- BIC = 440.9

Scree plot

For peer review only

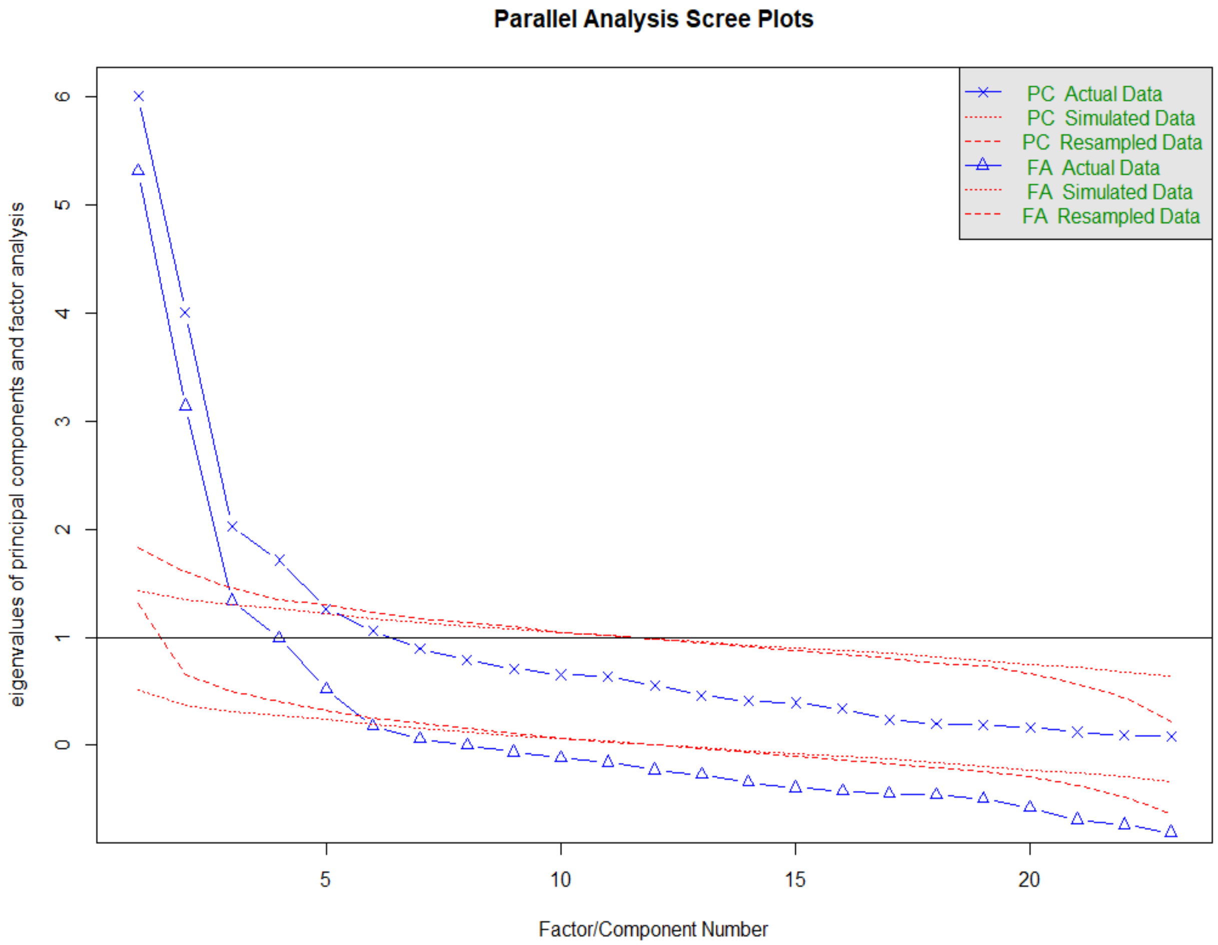

Figure 2. Modified ABCD Questionnaire 23 items with smoking.

Table A2 (a). Factor loadings of the exploratory factor analysis of the risk scale with the smoking items

Items	Factor2	Factor3	Factor1	Factor4	Communality	Uniqueness
I feel I will suffer from a heart attack or stroke sometime during my life	0.86	-0.1	0.05	-0.02	0.76	0.24
It is likely that I will suffer from a heart attack or stroke in the future	0.91	0.06	0.02	-0.01	0.82	0.18
It is likely that I will have a heart attack or stroke sometime during my life	0.88	0.02	0	0	0.77	0.23
There is a good chance I will experience a heart attack or stroke in the next 10 years	0.72	0	-0.09	0.01	0.54	0.46
My chances of suffering from a heart attack or stroke in the next 10 years are great	0.64	-0.03	-0.1	0.01	0.45	0.55
It is likely I will have a heart attack or stroke because of my past and/or present behaviors	0.57	-0.07	0	0	0.33	0.67

I am not worried that I might have a heart attack or stroke (Reverse coded)	0.28	0.02	-0.14	0.1	0.1	0.9
I am concerned about the likelihood of having a heart attack or stroke in the near future	0.41	0.19	-0.12	0.08	0.19	0.81
I am thinking about exercising at least 2.5 hours a week	-0.03	-0.05	0.88	-0.02	0.73	0.27
I intend or want to exercise at least 2.5 hours a week	-0.02	0.05	0.87	-0.02	0.79	0.21
When I exercise for at least 2.5 hours a week I am doing something good for the health of my heart	0.03	0.17	0.62	0.09	0.55	0.45
I am confident that I can maintain a healthy weight by exercising at least 2.5 hours a week	-0.05	0.09	0.42	0.18	0.32	0.68
I am not thinking about exercising for 2.5 hours a week (Reverse coded)	0.02	0	0.53	0.09	0.33	0.67
When I eat five portions of fruit and vegetables a day I am doing something good for the health of my heart	0.04	0.07	0.35	0.35	0.36	0.64
Increasing my exercise to at least 2.5 hours a week will decrease my chances of having a heart attack or stroke	0.04	0.12	0.37	0.24	0.32	0.68
I am confident that I can eat at least five portions of fruit and vegetables a day within the next two months	-0.04	-0.05	0.12	0.64	0.45	0.55
I am thinking about eating at least five portions of fruit and vegetables a day	0.01	0	0.02	0.89	0.8	0.2
I am not thinking about eating at least five portions of fruit and vegetables a day (Reverse coded)	-0.01	0	-0.06	0.83	0.66	0.34
I am thinking of stopping smoking within two months	0.06	0.78	0.12	-0.06	0.67	0.33
I have reduced or stopped smoking	-0.03	0.83	0.02	-0.01	0.71	0.29
I intend or want to stop smoking	-0.05	0.9	-0.02	-0.01	0.8	0.2
If I stop smoking it will reduce my chances of having a heart attack or stroke	0.16	0.58	0.09	0.08	0.43	0.57
I am not thinking about stopping smoking	-0.12	0.56	-0.2	0.17	0.35	0.65

Table A2 (b): Summary of factor loadings and variance distribution of the risk scale with the smoking items

**Measures**	**Factor 2**	**Factor 3**	**Factor 1**	**Factor 4**
SS loadings	3.90	3.00	2.97	2.33
Proportion Var	0.17	0.13	0.13	0.10
Cumulative Var	0.17	0.30	0.43	0.53
Proportion Explained	0.32	0.25	0.24	0.19
Cumulative Proportion	0.32	0.57	0.81	1.00

For peer review only

Modified scale (20-items including the smoking items)

Non-missing samples: 89

The overall KMO is 0.79, which is slightly below the recommended range (0.8 to 1).

The Bartlet's test of Sphericity is significant (X2 = 915.41, p-value < 0.001), indicating the sample adequacy for factor analysis.

EFA results

- The root mean square of the residuals (RMSR) is 0.06
- Tucker Lewis Index of factoring reliability = 0.72
- RMSEA index = 0.118 and the 90 % confidence intervals are 0.111 and 0.126
- BIC = 153.72

Scree plot

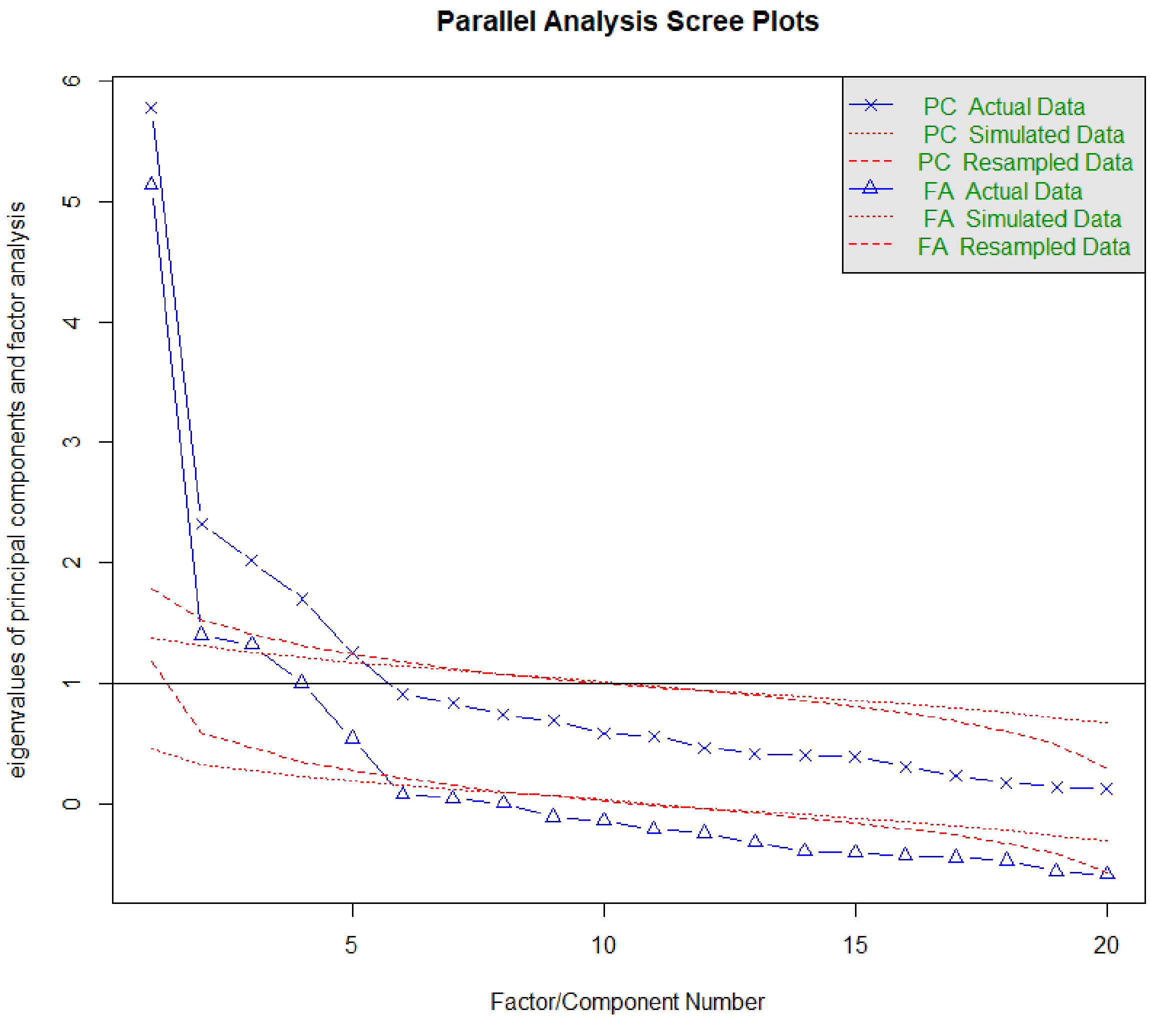

Figure 3. Modified ABCD Questionnaire 20 items with smoking.

Table A3 (a). Factor loadings of the exploratory factor analysis of the modified risk scale (20 items including the smoking items)

Items	Factor3	Factor1	Factor4	Factor2	Communality	Uniqueness
I feel I will suffer from a heart attack or stroke sometime during my life	-0.08	0.04	-0.03	0.76	0.60	0.40
There is a good chance I will experience a heart attack or stroke in the next 10 years	0.02	-0.08	-0.01	0.68	0.48	0.52
It is likely I will have a heart attack or stroke because of my past and/or present behaviors	-0.04	0.01	-0.01	0.61	0.38	0.62
I am not worried that I might have a heart attack or stroke (Reverse coded)	0.04	-0.13	0.10	0.35	0.14	0.86
I am concerned about the likelihood of having a heart attack or stroke in the near future	0.22	-0.11	0.07	0.45	0.23	0.77
I am thinking about exercising at least 2.5 hours a week	-0.06	0.88	-0.02	-0.04	0.74	0.26
I intend or want to exercise at least 2.5 hours a week	0.05	0.87	-0.02	-0.02	0.79	0.21
When I exercise for at least 2.5 hours a week I am doing something good for the health of my heart	0.17	0.62	0.09	0.04	0.55	0.45
I am confident that I can maintain a healthy weight by exercising at least 2.5 hours a week	0.09	0.42	0.18	-0.06	0.32	0.68
I am not thinking about exercising for 2.5 hours a week (Reverse coded)	0.01	0.53	0.09	0.03	0.32	0.68
When I eat five portions of fruit and vegetables a day I am doing something good for the health of my heart	0.08	0.35	0.35	0.07	0.37	0.63
Increasing my exercise to at least 2.5 hours a week will decrease my chances of having a heart attack or stroke	0.13	0.37	0.24	0.06	0.32	0.68

For peer review only

I am confident that I can eat at least five portions of fruit and vegetables a day within the next two months	-0.06	0.12	0.64	-0.05	0.46	0.54
I am thinking about eating at least five portions of fruit and vegetables a day	0.00	0.02	0.89	0.01	0.80	0.20
I am not thinking about eating at least five portions of fruit and vegetables a day (Reverse coded)	0.00	-0.06	0.83	-0.01	0.67	0.33
I am thinking of stopping smoking within two months	0.78	0.12	-0.06	0.04	0.66	0.34
I have reduced or stopped smoking	0.83	0.02	-0.01	-0.03	0.70	0.30
I intend or want to stop smoking	0.89	-0.02	-0.01	-0.07	0.80	0.20
If I stop smoking it will reduce my chances of having a heart attack or stroke	0.59	0.10	0.07	0.18	0.43	0.57
I am not thinking about stopping smoking	0.56	-0.20	0.17	-0.10	0.34	0.66

Table A3 (b): Summary of factor loadings and variance distribution of the modified risk scale (20 items including the smoking items)

Measures	Factor3	Factor1	Factor4	Factor2
SS loadings	3.00	2.96	2.33	1.80
Proportion Var	0.15	0.15	0.12	0.09
Cumulative Var	0.15	0.30	0.41	0.50
Proportion Explained	0.30	0.29	0.23	0.18
Cumulative Proportion	0.30	0.59	0.82	1.00

For peer review only

Appendix 5.

Item Analysis of published ABCD Risk Questionnaire sub-scales plus 5 unpublished items relating to smoking compared to Item Analysis of recommended edited ABCD Risk Questionnaire sub-scales plus 5 unpublished items relating to smoking.

Table 1. Item Analysis of published ABCD Risk Questionnaire sub-scales plus 5 unpublished items relating to smoking

**Perceived Risk of Heart Attack/ Stroke<br>8 Items<br>Cronbach's Alpha .861<br>(0.84,0.88) 95% CI**	**Inter-item correlation**	**Corrected Item-total correlation**	**Cronbach's alpha if item deleted**
It is likely that I will suffer from a heart attack or stroke in the future	**.832**	.756	.826
It is likely that I will have a heart attack or stroke some time during my life	**.869**	.777	.824
I feel I will suffer a heart attack or stroke some time during my life	**.616**	.784	.824
There is a good chance I will experience a heart attack or stroke in the next 10 years	**.729**	.722	.832
I am not worried that I might have a heart attack or stroke	.403	.624	.843
My chances of suffering a heart attack or stroke in the next 10 years are great	.245	.544	.852
It is likely that I will have a heart attack or stroke because of my past/present behaviours	.266	.319	**.876**
I am concerned about the likelihood of having a heart attack or stroke in the near future	.259	.387	**.870**
**Perceived Benefits and Intentions to Change<br>7 items<br>Cronbach's Alpha .801**	**Inter-item correlation**	**Corrected Item-total correlation**	**Cronbach's alpha if item deleted**
I am thinking about exercising at least 2.5 hours a week	**.727**	**.605**	.760
I intend or want to exercise at least 2.5 hours a week	.442	**.651**	.752
When I exercise for at least 2.5 hours a week I am doing something good for the health of my heart	.426	.593	.769
I am confident that I can maintain a healthy weight by exercising at	.294	.452	.790

least 2.5 hours a week within the next 2 months			
I am not thinking about exercising at least 2.5 hours a week	.264	.508	.781
When I eat at least 5 portions of fruit and vegetables a day I am doing something good for the health of my heart	.483	.483	.783
Increasing my exercise to at least 2.5 hours a week will decrease my chances of having a heart attack or stroke	.326	.474	.786
**Healthy Eating Intentions 3 items Cronbach's Alpha .787 (95% CI**	**Inter-item correlation**	**Corrected Item-total correlation**	**Cronbach's alpha if item deleted**
I am confident that I can eat at least 5 portions of fruit and vegetables a day within the next 2 months	.555	.533	**.812**
I am thinking about eating at least 5 portions of fruit and vegetables a day	**.683**	**.732**	.596
I am not thinking about eating at least 5 portions of fruit and vegetables a day	.424	**.624**	.713
**Perceived Benefits and Intentions to Stop Smoking 5 Items Cronbach's Alpha .943 95% CI**	**Inter-item correlation**	**Corrected item-total correlation**	**Cronbach's alpha if item deleted**
I am thinking of stopping smoking within the next 2 months	**.654**	.848	.932
I have reduced or stopped smoking	**.694**	.751	**.949**
I intend or want to stop smoking	**.829**	.906	.919
If I stop smoking it will reduce my chances of having a heart attack or stroke	**.834**	.886	.922
I am not thinking about stopping smoking	**.789**	.872	.925

For peer review only

Table 2. Item Analysis of edited ABCD Risk Questionnaire sub-scales plus 5 unpublished items relating to smoking.

**Perceived Risk of Heart Attack/ Stroke**<br>**5 Items**<br>**Cronbach's Alpha .86 (0.84,0.88) 95% CI**<br>**Omega 0.85 (0.83, 0.88) 95% CI**	**Inter-item correlation**	**Corrected Item-total correlation**	**Cronbach's alpha if item deleted**
It is likely that I will have a heart attack or stroke some time during my life	**.869**	.777	.824
There is a good chance I will experience a heart attack or stroke in the next 10 years	**.729**	.722	.832
I am not worried that I might have a heart attack or stroke	.403	.624	.843
It is likely that I will have a heart attack or stroke because of my past/present behaviours	.266	.319	**.876**
I am concerned about the likelihood of having a heart attack or stroke in the near future	.259	.387	**.870**
**Perceived Benefits and Intentions to Change**<br>**6 items**<br>**Cronbach's Alpha .84 (.81-.86) 95% CI**<br>**Omega 0.82 (0.78, 0.85) 95% CI**	**Inter-item correlation**	**Corrected Item-total correlation**	**Cronbach's alpha if item deleted**
I am thinking about exercising at least 2.5 hours a week	**.727**	**.605**	.760
I intend or want to exercise at least 2.5 hours a week	.442	**.651**	.752
When I exercise for at least 2.5 hours a week I am doing something good for the health of my heart	.426	.593	.769
I am confident that I can maintain a healthy weight by exercising at least 2.5 hours a week within the next 2 months	.294	.452	.790
I am not thinking about exercising at least 2.5 hours a week	.264	.508	.781
Increasing my exercise to at least 2.5 hours a week will decrease my chances of having a heart attack or stroke	.326	.474	.786
**Healthy Eating Intentions**<br>**4 items**	**Inter-item correlation**	**Corrected Item-total correlation**	**Cronbach's alpha if item deleted**

**Cronbach's Alpha .84 (.81-.86) 95% CI**<br>**Omega 0.84 (0.81, 0.88) 95% CI**			
I am confident that I can eat at least 5 portions of fruit and vegetables a day within the next 2 months	.555	.533	**.812**
I am thinking about eating at least 5 portions of fruit and vegetables a day	**.683**	**.732**	.596
I am not thinking about eating at least 5 portions of fruit and vegetables a day	.424	**.624**	.713
When I eat at least 5 portions of fruit and vegetables a day I am doing something good for the health of my heart	.483	.483	.783
**Smoking Intentions**<br>**5 items**<br>**Cronbach's Alpha .85 (.83-.87) 95% CI**<br>**Omega 0.84 (0.81, 0.91) 95% CI**	**Inter-item correlation**	**Corrected Item-total correlation**	**Cronbach's alpha if item deleted**
I am thinking of stopping smoking within the next 2 months	**.654**	.848	.932
I have reduced or stopped smoking	**.694**	.751	**.949**
I intend or want to stop smoking	**.829**	.906	.919
If I stop smoking it will reduce my chances of having a heart attack or stroke	**.834**	.886	.922
I am not thinking about stopping smoking	**.789**	.872	.925

For peer review only

Appendix 6. Characteristics of the sample population

**Population Characteristics**		**N**	**% total**
**Gender**	Male	218	49.8
	Female	220	50.2
**Age Group**	18-30	78	17.8
	30-39	80	18.3
	40-49	82	18.7
	50-59	99	22.6
	60-74	78	17.8
	74+	53	12.1
**Deprivation**	IMD1- least deprived	84	17.98
	IMD2	55	11.77
	IMD3	83	17.77
	IMD4	89	19.05
	IMD5- most deprived	156	33.4

For peer review only

Appendix 7. Modified ABCD Risk Questionnaire

Mark Bowyer, Hamid Hassen

Scale	Items	Coding
**Perceived Risk of Heart Attack or Stroke**	1. It is likely that I will have a heart attack or stroke sometime in my life	4= Strongly disagree, 3= Disagree, 2= Agree, 1= Strongly Agree; N/A= 0
	2. There is a good chance I will experience a heart attack or stroke in the next 10 years	4= Strongly disagree, 3= Disagree, 2= Agree, 1= Strongly Agree; N/A= 0
	3. It is (more) likely I will have a heart attack or stroke because of my past and/or present behaviours	4= Strongly disagree, 3= Disagree, 2= Agree, 1= Strongly Agree; N/A= 0
	4. I am not worried that I might have a heart attack or stroke	**REVERSE CODED** 4= Strongly disagree, 3= Disagree, 2= Agree, 1= Strongly Agree; N/A= 0
	5. I am concerned about the likelihood of having a heart attack or stroke in the near future	4= Strongly disagree, 3= Disagree, 2= Agree, 1= Strongly Agree; N/A= 0
**Perceived Benefits and Intentions to Exercise**	6. I am thinking about exercising at least 2.5 hours a week	4= Strongly disagree, 3= Disagree, 2= Agree, 1= Strongly Agree; N/A= 0
	7. I intend or want to exercise at least 2.5 hours a week	4= Strongly disagree, 3= Disagree, 2= Agree, 1= Strongly Agree; N/A= 0
	8. When I exercise for at least 2.5 hours a week I am doing something good for the health of my heart	4= Strongly disagree, 3= Disagree, 2= Agree, 1= Strongly Agree; N/A= 0
	9. I am confident that I can maintain a healthy weight by exercising at least 2.5 hours a week	4= Strongly disagree, 3= Disagree, 2= Agree, 1= Strongly Agree; N/A= 0
	10. I am not thinking about exercising for 2.5 hours a week	**REVERSE CODED** 4= Strongly disagree, 3= Disagree, 2= Agree, 1= Strongly Agree; N/A= 0
	11. Increasing my exercise to at least 2.5 hours a week will decrease my chances of having a heart attack or stroke	4= Strongly disagree, 3= Disagree, 2= Agree, 1= Strongly Agree; N/A= 0

For peer review only

**Perceived Benefit and Healthy Eating Intentions**	12. I am confident that I can eat at least five portions of fruit and vegetables a day within the next two months	4= Strongly disagree, 3= Disagree, 2= Agree, 1= Strongly Agree; N/A= 0
	13. I am thinking about eating at least five portions of fruit and vegetables a day	4= Strongly disagree, 3= Disagree, 2= Agree, 1= Strongly Agree; N/A= 0
	14. I am not thinking about eating at least five portions of fruit and vegetables a day	**REVERSE CODED** 4= Strongly disagree, 3= Disagree, 2= Agree, 1= Strongly Agree; N/A= 0
	15. When I eat five portions of fruit and vegetables a day I am doing something good for the health of my heart	4= Strongly disagree, 3= Disagree, 2= Agree, 1= Strongly Agree; N/A= 0
**Benefits and Intentions to Stop Smoking**	16. I am thinking of stopping smoking within two months	4= Strongly disagree, 3= Disagree, 2= Agree, 1= Strongly Agree; N/A= 0
	17. I have reduced or stopped smoking	4= Strongly disagree, 3= Disagree, 2= Agree, 1= Strongly Agree; N/A= 0
	18. I intend or want to stop smoking	4= Strongly disagree, 3= Disagree, 2= Agree, 1= Strongly Agree; N/A= 0
	19. If I stop smoking it will reduce my chances of having a heart attack or stroke	4= Strongly disagree, 3= Disagree, 2= Agree, 1= Strongly Agree; N/A= 0
	20. I am not thinking about stopping smoking	**REVERSE CODED** 4= Strongly disagree, 3= Disagree, 2= Agree, 1= Strongly Agree; N/A= 0

For peer review only

Reporting checklist for cross sectional study.

Based on the STROBE cross sectional guidelines.

Instructions to authors

Complete this checklist by entering the page numbers from your manuscript where readers will find each of the items listed below.

Your article may not currently address all the items on the checklist. Please modify your text to include the missing information. If you are certain that an item does not apply, please write "n/a" and provide a short explanation.

Upload your completed checklist as an extra file when you submit to a journal.

In your methods section, say that you used the STROBE cross sectionalreporting guidelines, and cite them as:

von Elm E, Altman DG, Egger M, Pocock SJ, Gotzsche PC, Vandenbroucke JP. The Strengthening the Reporting of Observational Studies in Epidemiology (STROBE) Statement: guidelines for reporting observational studies.

		Reporting Item	Page Number
**Title and abstract**			
Title	#1a	Indicate the study’s design with a commonly used term in the title or the abstract	1
Abstract	#1b	Provide in the abstract an informative and balanced summary of what was done and what was found	1
**Introduction**			
Background / rationale	#2	Explain the scientific background and rationale for the investigation being reported	3
Objectives	#3	State specific objectives, including any prespecified hypotheses	3
**Methods**			
Study design	#4	Present key elements of study design early in the	4

For peer review only

		paper	
Setting	#5	Describe the setting, locations, and relevant dates, including periods of recruitment, exposure, follow-up, and data collection	4
Eligibility criteria	#6a	Give the eligibility criteria, and the sources and methods of selection of participants.	4
	#7	Clearly define all outcomes, exposures, predictors, potential confounders, and effect modifiers. Give diagnostic criteria, if applicable	6
Data sources / measurement	#8	For each variable of interest give sources of data and details of methods of assessment (measurement). Describe comparability of assessment methods if there is more than one group. Give information separately for for exposed and unexposed groups if applicable.	6
Bias	#9	Describe any efforts to address potential sources of bias	7
Study size	#10	Explain how the study size was arrived at	7
Quantitative variables	#11	Explain how quantitative variables were handled in the analyses. If applicable, describe which groupings were chosen, and why	7
Statistical methods	#12a	Describe all statistical methods, including those used to control for confounding	7
Statistical methods	#12b	Describe any methods used to examine subgroups and interactions	7
Statistical methods	#12c	Explain how missing data were addressed	7
Statistical methods	#12d	If applicable, describe analytical methods taking account of sampling strategy	7
Statistical methods	#12e	Describe any sensitivity analyses	7
**Results**			
Participants	#13a	Report numbers of individuals at each stage of study—	7

For peer review only

		eg numbers potentially eligible, examined for eligibility, confirmed eligible, included in the study, completing follow-up, and analysed. Give information separately for for exposed and unexposed groups if applicable.	
Participants	#13b	Give reasons for non-participation at each stage	7
Participants	#13c	Consider use of a flow diagram	n/a No drop-out
Descriptive data	#14a	Give characteristics of study participants (eg demographic, clinical, social) and information on exposures and potential confounders. Give information separately for exposed and unexposed groups if applicable.	7
Descriptive data	#14b	Indicate number of participants with missing data for each variable of interest	7
Outcome data	#15	Report numbers of outcome events or summary measures. Give information separately for exposed and unexposed groups if applicable.	7
Main results	#16a	Give unadjusted estimates and, if applicable, confounder-adjusted estimates and their precision (eg, 95% confidence interval). Make clear which confounders were adjusted for and why they were included	8
Main results	#16b	Report category boundaries when continuous variables were categorized	n/a Continuous variables not measured
Main results	#16c	If relevant, consider translating estimates of relative risk into absolute risk for a meaningful time period	n/a No measurement of risk
Other analyses	#17	Report other analyses done—e.g., analyses of subgroups and interactions, and sensitivity analyses	10
**Discussion**			
Key results	#18	Summarise key results with reference to study objectives	12
Limitations	#19	Discuss limitations of the study, taking into account	12

For peer review only

		sources of potential bias or imprecision. Discuss both direction and magnitude of any potential bias.	
Interpretation	#20	Give a cautious overall interpretation considering objectives, limitations, multiplicity of analyses, results from similar studies, and other relevant evidence.	12
Generalisability	#21	Discuss the generalisability (external validity) of the study results	13
**Other Information**			
Funding	#22	Give the source of funding and the role of the funders for the present study and, if applicable, for the original study on which the present article is based	1

Notes:

- 13c: n/a No drop-out
- 16b: n/a Continuous variables not measured
- 16c: n/a No measurement of risk The STROBE checklist is distributed under the terms of the Creative Commons Attribution License CC-BY. This checklist was completed on 08. June 2021 using https://www.goodreports.org/, a tool made by the EQUATOR Network in collaboration with Penelope.ai

For peer review only
